# Diverse structures and antihepatoma effect of sesquiterpenoid dimers from *Artemisia eriopoda* by AKT/STAT signaling pathway

**DOI:** 10.1038/s41392-022-01267-6

**Published:** 2023-02-15

**Authors:** Xiaofeng He, Wenjing Ma, Jing Hu, Tianze Li, Changan Geng, Yunbao Ma, Mengfei Wang, Kexin Yang, Xuemei Zhang, Ji-Jun Chen

**Affiliations:** 1 State Key Laboratory of Phytochemistry and Plant Resources in West China, Kunming Institute of Botany, Chinese Academy of Sciences, Kunming, 650201 PR China; 2 University of Chinese Academy of Sciences, Beijing, 100049 PR China

**Keywords:** Medicinal chemistry, Drug development

**Dear Editor**,

Liver cancer as a common malignant tumor has become a major source of morbidity and mortality worldwide.^1^ Currently, four synthetic drugs and three monoclonal antibody drugs have been approved for the treatment of hepatocellular carcinoma (HCC) since 2007. Whereas, the disadvantages of the above drugs such as low response rates, severe side effects, and drug resistance hamper their therapeutic effects. Structurally and biologically diverse natural products are a major source in the search for novel antihepatoma agents. Sesquiterpenoid dimers are a kind of important components from the genus *Artemisia* and exhibit remarkably cytotoxic activity.^2^

*Artemisia eriopoda* has been used as a folk medicine to treat rheumatoid arthritis, edema, and thanatophidia.^3^ However, neither the sesquiterpenoid dimers nor the antihepatoma activity has been reported from *A. eriopoda*. Continuing research of the genus *Artemisia* revealed that the ethanol extract of *A. eriopoda* demonstrated cytotoxicity against HepG2, Huh7 and SK-Hep-1 cells (Supplementary Table 1). To clarify the antihepatoma constituents, 36 novel sesquiterpenoid dimers were isolated from the active fractions of *A. eriopoda* under the guidance of cytotoxicity bioassay by column chromatography (including silica gel, MCI gel CHP 20P, Rp C_18_, and Sephadex LH-20) and semi-preparative HPLC. Their structures were elucidated as artemeriopodins A1‒A3 (**1**‒**3**), B1‒B2 (**4**, **5**), C1‒C4 (**6**‒**9**), D (**10**), E (**11**), F1‒F15 (**12**‒**26**), G1‒G8 (**27**‒**34**), H (**35**), and I (**36**) by spectral data including HRESIMS, IR, UV, 1D and 2D NMR, and ECD calculations (Fig. 1a, Supplementary Figs. 2, 3, 5, and 6, Supplementary Tables 2‒11). Among them, seven compounds (**12**, **14**, **16**, **17**, **19**, **27**, **29**) were unambiguously confirmed by the single-crystal X-ray diffraction (Supplementary Fig. 4). These compounds were classified as nine types of sesquiterpenoid dimers involving Diels-Alder reaction, radical addition, and esterification, which suggested chemical diversities of sesquiterpenoid dimers in *A. eriopoda*.

**Fig. 1:**
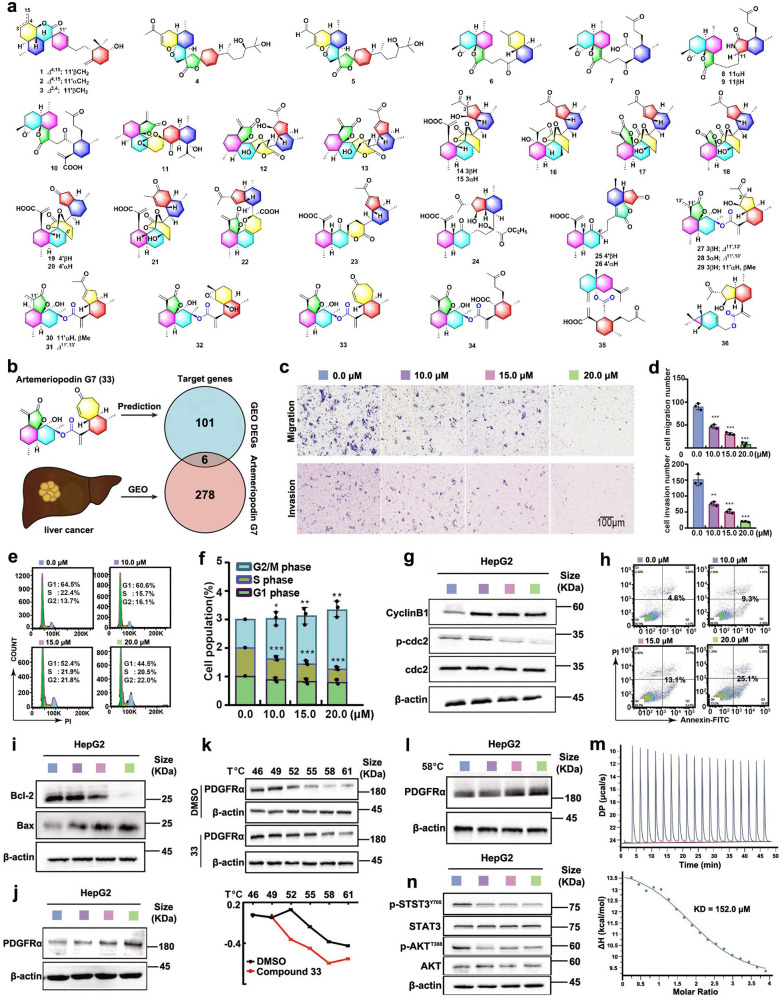
Structurally diverse sesquiterpenoid dimers from *Artemisia eriopoda* exert antihepatoma effect by AKT/STAT signaling pathway. **a** The structures of compounds **1**–**36**. **b** Schematic diagram of investigation on the target and mechanism analysis of artemeriopodin G7 (**33**). **c** Compound **33** regulated HepG2 cell migration and invasion examined by Transwell assay. **d** The quantification data for **c**. **e** HepG2 cells were treated with compound **33** (0.0, 10.0, 15.0, and 20.0 μM) for 12 h, and effect of compound **33** on the G2/M cell cycle transition was tested by PI staining and flow cytometry. **f** The quantification data for **e**. **g** Cell cycle related proteins CyclinB1, cdc2, and phosphorylated cdc2 were examined by Western blot. **h** HepG2 cells were treated with different concentrations (0.0, 10.0, 15.0, and 20.0 μM) of compound **33** for 48 h, flow cytometric analysis, and cell apoptosis quantification of HepG2 cells. **i** The apoptosis-related protein levels were treated with **33** for 48 h by Western blot. **j** The expression of PDGFRA proteins was examined in HepG2 cells by Western blot. **k** CETSA analysis of binding between compound **33** and PDGFRA protein. Protein levels were investigated at different temperatures under the treatment of **33** (20.0 μM) in HepG2 cells. **l** Protein levels were investigated at different concentrations of **33** (58 °C). **m** Isothermal titration calorimetry (ITC) enthalpogram of the interaction between **33** and PDGFRA. The titration curve was depicted as a function of the molar ratio between PDGFRA and the calculated concentration of **33** in the assay. **n** Total and phosphorylated forms of AKT/STAT proteins were examined by immunoblot with indicating antibodies in HepG2 cells. *P < 0.05, **P < 0.01, and ***P < 0.001, *n* = 3

The isolates except for **4** and **5** (limited amount) were evaluated for cytotoxicity against HepG2, Huh7, and SK-Hep-1 cell lines (Supplementary Table 12). Of them, compounds **13**, **16**, **31**, and **33** exhibited obvious cytotoxicity against HepG2 cells with IC_50_ values of 14.3, 12.2, 17.2, and 16.0 μM, which were equivalent to that of sorafenib (IC_50_, 11.0 μM); compounds **31** and **33** demonstrated cytotoxicity against Huh7 and SK-Hep-1 cells with IC_50_ values of 10.3 and 22.3, 18.3 and 19.0 μM, which were comparable to sorafenib (IC_50_, 12.3 and 18.1 μM). Interestingly, artemeriopodins G5 (**31**) and G7 (**33**) showed significant activity against three HepG2, Huh7, and SK-Hep-1 cells. Artemeriopodin G7 (**33**) featured an unprecedented 7/6 bicyclic scaffold, and was evaluated by CCK8 assay to compare with sorafenib on THLE-2 cells, suggesting that **33** showed a better safety on THLE-2 cells (IC_50_, 32.0 μM) than sorafenib (IC_50_, 16.7 μM) (Supplementary Fig. 12). From a comprehensive consideration, artemeriopodin G7 (**33**) was chosen for the further investigation.

The network pharmacology analysis predicted that HSD11B1, CYP2C9, CYP3A4, PDGFRA, CETP, and CCNA2 were potential targets of artemeriopodin G7 (**33**) (Fig. 1b, Supplementary Fig. 7b‒e, Supplementary Tables 13 and 14). Among them, HSD11B1, CYP2C9, CYP3A4, PDGFRA, and CETP were lowly expressed but CCNA2 was highly expressed in HCC tissues, which led to a poor clinical outcome and a worse prognosis in HCC patients (Supplementary Fig. 8a‒f). Next, GO and KEGG pathway enrichment analyses indicated that many signaling pathways were closely associated with **33** (Supplementary Fig. 9a, b, Supplementary Table 15). Then, a molecular docking manifested that **33** had high binding affinity with PDGFRA that was related to cell proliferation and metastasis (Supplementary Fig. 10),^4^ and binding energy was ‒6.9 kcal/mol, which were higher than the empirical threshold (−5.0 kcal/mol).^5^ The findings indicated that artemeriopodin G7 (**33**) might play an antihepatoma role through the expression of PDGFRA protein regulated by the AKT/STAT signaling pathway.

To verify the bioinformatics analysis, a series of experiments of artemeriopodin G7 (**33**) in HepG2 cells were carried out. Firstly, the Transwell assay indicated that **33** dose-dependently decreased cell migration and invasion in contrast to the control group (Fig. 1c, d). Then, the flow cytometry manifested that artemeriopodin G7 (**33**) significantly induced cell cycle arrest in G2/M phase in a dose-dependent manner (Fig. 1e, f), and the percentage of G2/M phase cells increased from 13.7% to 16.1% (10.0 μM), 21.8% (15.0 μM), and 22.0% (20.0 μM) by comparison to the control cells. The results of the key-related proteins with cell cycle indicated that artemeriopodin G7 (**33**) downregulated the expression of phosphorylated cdc2 and upregulated the expression of CyclinB1 with increasing concentrations of **33** (Fig. 1g). Furthermore, the flow cytometry manifested that **33** induced cell apoptosis (Fig. 1h, Supplementary Fig. 11b). The Western blot assay revealed that **33** induced cell apoptosis by inhibiting the expression of Bcl-2 and activating the expression of Bax (Fig. 1i). The results suggested that artemeriopodin G7 (**33**) inhibited the growth of HepG2 cells through suppressing tumor cell proliferation, migration, and inducing cell apoptosis and G2/M cell cycle arrest.

Furthermore, the Western blot assay manifested that artemeriopodin G7 (**33**) upregulated the expression of PDGFRA protein about 18.3%, 49.1%, and 57.8% at 10.0, 15.0 and 20.0 μM compared to control (Fig. 1j, Supplementary Fig. 13b). The binding between compound **33** and PDGFRA was determined by using the cellular thermal shift assay (CETSA). Treatment of HepG2 cells with **33** (20.0 μM) led to the significant thermal stabilization of PDGFRA by comparing with the control group (Fig. 1k). Meanwhile, **33** increased the stability of PDGFRA about 15.6%, 28.8%, and 48.1% at concentrations of 10.0, 15.0, and 20.0 μM (58 °C) (Fig. 1l, Supplementary Fig. 14c). Subsequently, the isothermal titration calorimetry (ITC) showed that the KD value between artemeriopodin G7 (**33**) and PDGFRA was 152.0 μM (Fig. 1m). Surface Plasmon Resonance (SPR) assay suggested that compound **33** bound to PDGFRA by a dose-dependent response with KD value of 90.1 μM (Supplementary Fig. 16). These results verified that compound **33** directly targeted on PDGFRA and were consistent with the predicted data, demonstrating that PDGFRA might be one of acting targets of **33**. The network pharmacology analysis indicated that **33** might play an antihepatoma role by the AKT/STAT signaling pathway. The immunoblot analysis revealed that compound **33** significantly inhibited the expression of phosphorylated AKT/STAT (Fig. 1n). Thus, artemeriopodin G7 (**33**) exerted antihepatoma effect through AKT/STAT.

In summary, 36 novel sesquiterpenoid dimers (**1**‒**36**) were isolated from *A. eriopoda* under the guidance of bioassay and elucidated by spectral data. Structurally, these compounds were classified into nine different types based on connecting modes of two monomeric sesquiterpenoids. Antihepatoma assay suggested that most of compounds were cytotoxic, and artemeriopodins G5 (**31**) and G7 (**33**) showed significant cytotoxicity. The network pharmacology analysis predicated that PDGFRA might be one of acting targets of **33**, and signaling pathway significantly enriched in AKT/STAT. Functional experiments verified that artemeriopodin G7 (**33**) could inhibit cell migration and invasion, induce G2/M cell cycle arrest and cell apoptosis, upregulate PDGFRA expression in HepG2 cells, and dramatically suppress the activity of AKT/STAT signaling pathway by downregulating the expression of phosphorylated AKT/STAT. Furthermore, CETSA, ITC, and SPR assays demonstrated **33** was bound with PDGFRA. This investigation first disclosed a series of novel sesquiterpenoid dimers with antihepatoma effects from *A. eriopoda* and the mechanism of artemeriopodin G7 (**33**), which provided important clues for searching therapeutic drugs and new antihepatoma candidates from natural sources.

## Supplementary information

SIGTRANS-07111R1-Supplementary Materials

SIGTRANS-07111R-Supplementary Material -Tables S13-S15

## Data Availability

All data and materials are presented in the main manuscript or supplementary materials and are available on request. The crystallographic data of compounds **12**, **14**, **16**, **17**, **19**, **27**, and **29** were deposited at the Cambridge Crystallographic Data Centre.

## References

[1] Ma L (2018). A cardiac glycoside HTF-1 isolated from Helleborus thibetanus Franch displays potent in vitro anti-cancer activity via caspase-9, MAPK and PI3K-Akt-mTOR pathways. Eur. J. Med. Chem..

[2] Su LH (2021). New guaiane-type sesquiterpenoid dimers from *Artemisia atrovirens* and their antihepatoma activity. Acta Pharm. Sin. B.

[3] Hu JF, Jia ZJ, Bai SP (1998). Two new polyacetylenes from *Artemisia eriopoda*. Planta Med..

[4] Oseini AM, Roberts LR (2009). PDGFRα: a new therapeutic target in the treatment of hepatocellular carcinoma?. Expert Opin. Ther. Targets.

[5] Wang C, Yan Q, Hu M, Qin MD, Feng ZQ (2016). Effect of AURKA gene expression knockdown on angiogenesis and tumorigenesis of human ovarian cancer cell lines. Target. Oncol..

