# Supplementary material for: Diverse structures and antihepatoma effect of sesquiterpenoid dimers from Artemisia eriopoda by AKT/STAT signaling pathway
Source: Signal Transduct Target Ther. 2023 Feb 15;8:64. doi: 10.1038/s41392-022-01267-6 (PMC9929101; doi:10.1038/s41392-022-01267-6)
Supplement: Supplementary file 1 — SIGTRANS-07111R1-Supplementary Materials [file 41392_2022_1267_MOESM1_ESM.doc]

**Supplementary Materials**

**Diverse structures and antihepatoma effect of sesquiterpenoid dimers from *Artemisia eriopoda* by AKT/STAT signaling pathway**

Xiaofeng He^a,†^, Wenjing Ma^a,b,†^, Jing Hu^a^, Tianze Li^a^, Changan Geng^a^, Yunbao Ma^a^, Mengfei Wang^a,b^, Kexin Yang^a,b^, Xuemei Zhang^a^, Ji-Jun Chen^a,b^,*

*^a^State Key Laboratory of Phytochemistry and Plant Resources in West China, Kunming Institute of Botany, Chinese Academy of Sciences, Kunming 650201, People's Republic of China*

*^b^University of Chinese Academy of Sciences, Beijing 100049, People's Republic of China*

*Corresponding author. Prof. Dr. Ji-Jun Chen, State Key Laboratory of Phytochemistry and Plant Resources in West China, Kunming Institute of Botany, Chinese Academy of Sciences, 132^#^ Lanhei Road, Kunming 650201, Yunnan, People's Republic of China.

Tel.: +86 871 65223265, Fax: +86 871 65227197, *E-mail address*: [chenjj@mail.kib.ac.cn](mailto:chenjj@mail.kib.ac.cn) (Ji-Jun Chen)

^†^These authors made equal contributions to this work.

**Supplementary information includes:**

Detailed results and discussion, including structural identification, cytotoxicity, network pharmacology, functional experiments, CETSA assays, Isothermal titration calorimetry (ITC), and Surface Plasmon Resonance (SPR) assay.

Materials and Methods
Supplementary Figures S1‒S17
Supplementary Tables S1‒S12
Supplementary References

The spectral data of compounds **1**‒**36**, including HRESIMS, IR, 1D and 2D NMR, and ECD.

**Detailed results and discussion**

***Cytotoxic activity***

The EtOH extract and EtOAc fraction (Fr. A) of *A. eriopoda* showed cytotoxicity against HepG2, Huh7, and SK-Hep-1 cell lines with inhibitory ratios of 59.4%, 58.9%, 53.5% and 97.6%, 81.7%, 79.5% (200 μg/mL) (Fig. S1). The most active Fr. A was further separated into six sub-fractions by silica gel column chromatography (CC), of which Frs. A-3‒A-5 displayed obvious cytotoxic activities with inhibitory ratios ranging from 52.9% to 94.9%. Especially, Frs. A-4 and A-5 possessed significantly cytotoxic activities with inhibitory ratios higher than 60% at 100 μg/mL, indicating more potent than other subfractions. Thus, Frs. A-4 and A-5 were chosen for the investigation to provide 36 compounds by CC (silica gel, MCI gel CHP 20P, and Sephadex LH-20) and semi-preparative HPLC. Their structures were elucidated based on the analyses of HRESIMS, 1D and 2D NMR, ECD spectra, and single-crystal X-ray diffraction.

***Structural identification***

Artemeriopodin A1 (**1**), colorless oil, possessed a molecular formula of C_30_H_44_O_3_ as determined by its HRESIMS data at *m/z* 453.3356 [M + H]^+^ (calcd. for C_30_H_45_O_3_, 453.3363), indicating nine degrees of unsaturation. The IR absorptions at 3447 and 1730 cm^−1^ suggested the presence of hydroxyl and carbonyl groups in compound **1**. The ^13^C NMR (DEPT) spectrum of **1** (Table S1) displayed 30 carbon resonances, corresponding to three methyls, 13 methylenes (including two olefinic ones at *δ*_C_ 104.9 and 108.6), eight methines (including one olefinic one at *δ*_C_ 117.0, two oxygenated ones at *δ*_C_ 76.3 and 77.2), and six quaternary carbons (including three olefinic ones at *δ*_C_ 137.6, 146.2 and 147.1, one carbonyl at *δ*_C_ 178.0). In the ^1^H NMR spectrum (Table S2), one secondary methyl at *δ*_H_ 0.91 (3H, d, *J* = 6.0 Hz, H-14) and two tertiary methyls at *δ*_H_ 1.03 (3H, s, H-13') and 0.74 (3H, s, H-14') were clearly observed. The proton signals at *δ*_H_ 3.42 (1H, dd, *J* = 9.6, 4.2 Hz), 4.59 (1H, s), 4.81 (1H, d, *J* = 1.2 Hz), 4.88 (1H, s), 4.97 (1H, d, *J* = 12.0 Hz), 5.04 (1H, d, *J* = 1.2 Hz), and 5.21 (1H, brd, *J* = 1.2 Hz) should be attributed to oxygenated or olefinic protons. Thus, a pentacyclic terpenoid skeleton was proposed for compound **1**.

The ^1^H-^1^H COSY correlations of H_2_-3/H_2_-2/H-1/H-10(H-6)/H_2_-9/H_2_-8/H-7/H-6/H-5, in combination with the HMBC correlations from H_3_-14 to C-1/C-9/C-10, from H_2_-15 to C-3/C-4/C-5, from H-10 to C-2/C-6/C-8, and from H_2_-13 to C-7/C-11/C-12 (Fig. S3), determined the existence of a cadinene sesquiterpenoid moiety (moiety A) in compound **1**. Meanwhile, the key HMBC correlations of H-5 with C-7/C-12 further established a lactone connecting C-5 and C-12. Similarly, the ^1^H-^1^H COSY relationships of H-2'/H_2_-3'/H_2_-4', H-6'/H_2_-7/H_2_-8', and H-10'/H_2_-11', along with the key HMBC correlations of H_3_-13' with C-1'/C-2'/C-6'/C-14', of H_3_-14' with C-1'/C-2'/C-6'/C-13', of H_2_-15' with C-4'/C-5'/C-6', and of H-10' with C-8'/C-9'/C-12', constructed a monocyclofarnesane-type sesquiterpenoid moiety (moiety B). The moieties A and B were assembled by C-11‒C-11' and C-13‒C-12' bonds to form a spiro-cyclohexane ring, which was confirmed by the ^1^H-^1^H COSY correlation of H_2_-13/H_2_-12' as well as the HMBC correlations from H_2_-11' to C-7/C-11/C-12/C-13 and from H_2_-13 to C-9'/C-11'. Thus, the planar structure of compound **1** was established as a cadinane-monocyclofarnesane hybridized skeleton.

The relative stereochemistry of compound **1** was assigned by the interpretation of the coupling constants and ROESY correlations. The large coupling constant between H-5 and H-6 (*J* = 12.0 Hz) suggested their *anti*-axial orientations. The key correlations of H-1 with H-5/H-7/H_3_-14, of H-6 with H-10/H-11'a, of H-7 with H-13a, of H-2' with H-6'/H_3_-13', and of H_3_-14' with H_2_-7' were obviously observed in the ROESY spectrum (Fig. S4). In addition, the ROESY correlation of H-10' with H_2_-8' indicated the *Z*-configuration of ∆^9',10'^-double bond. In order to determine the absolute configuration of **1**, ECD calculation was conducted using the time-dependent density functional theory (TD-DFT) method at the B3LYP/6-31+G (d,p) level. The calculated ECD curve matched well with the experimental one, and the absolute configuration of compound **1** was assigned as 1*R*,5*S*,6*S*,7*S*,10*S*,11*S*,2'*R*,6'*R* (Fig. S5).

Artemeriopodin A2 (**2**) had the same molecular formula of C_30_H_44_O_3_ with compound **1** based on their HRESIMS data (*m/z* 453.3359 [M + H]^+^, calcd. for C_30_H_45_O_3_, 453.3363). Detailed comparison of their 1D and 2D NMR spectra (Tables S1 and S2, Figs. S2 and S3) suggested that compounds **2** and **1** shared the same planar structures and relative configurations of the sesquiterpenoid moieties A and B, but different in the configuration at C-11 as deduced from the chemical shifts at C-11 (*δ*_C_ 44.6 *vs* 44.0), C-13 (*δ*_C_ 25.9 *vs* 26.3), C-9' (*δ*_C_ 134.4 *vs* 137.6), C-10' (*δ*_C_ 120.2 *vs* 117.0), C-11' (*δ*_C_ 38.8 *vs* 35.6), and C-12' (*δ*_C_ 21.5 *vs* 24.5). The above inference indicated that compound **2** was the epimer of **1** at C-11, which was verified by the ROESY correlations of H-6 with H_2_-13 and of H-7 with H_2_-11' in compound **2** rather than those of H-6 with H_2_-11' and of H-7 with H_2_-13 in compound **1**. Subsequently, the absolute stereochemistry of compound **2** was elucidated as 1*R*,5*S*,6*S*,7*S*, 10*S*,11R,2'*R*,6'*R* by comparing with the calculated ECD and the experimental curves (Fig. S5).

Artemeriopodin A3 (**3**) possessed the same molecular formula of C_30_H_44_O_3_ as compounds **1** and **2** based on their HRESIMS data (*m/z* 453.3357 [M + H]^+^). The NMR data of compound **3** resembled with those of **1**, and the major difference was that a terminal double bond between C-4 and C-15 in **1** was absent and replaced by a trisubstituent double bond [*δ*_H_ 5.37 (1H, m, H-3); *δ*_C_ 122.8 (C-3) and 132.0 (C-4)] in **3**. The HMBC correlations from a singlet methyl H_3_-15 to C-3/C-4/C-5 confirmed the formation of the trisubstituent double bond between C-3 and C-4. Compound **3** was assigned the same configuration as **1** by the similar coupling constant, as well as the consistent ROESY and ECD spectra. Thus, the absolute stereochemistry of compound **3** was determined as 1*R*,5*S*,6*S*,7*S*,10*S*,11*S*,2'*R*,6'*R*.

Artemeriopodin B1 (**4**), colorless oils, possessed a molecular formula of C_30_H_44_O_7_ based on its HRESIMS data (*m/z* 517.3157 [M + H]^+^, calcd. for C_30_H_45_O_7_, 517.3160). The ^13^C NMR displayed 30 carbon resonances, including five methyls, nine methylenes (including an oxygenated one at *δ*_C_ 77.5), nine methines (including an oxygenated one at *δ*_C_ 78.5, and three olefinic ones at *δ*_C_ 153.0, 134.9 and 130.2), and seven quaternary carbons (including two carbonyls at *δ*_C_ 196.3 and 176.9, an olefinic carbon at *δ*_C_ 119.0, and three oxygenated ones at *δ*_C_ 110.2, 81.9 and 73.2). Its ^1^H NMR spectrum showed signals assignable to five methyls [*δ*_H_ 2.22 (3H, s), 1.23 (3H, s), 1.18 (3H, s), 0.97 (3H, d, *J* = 6.4 Hz), 0.88 (3H, d, *J* = 7.2 Hz)], three olefinic methine protons [*δ*_H_ 7.38 (d, *J* = 1.6 Hz), 5.75 (dd, *J* = 10.4, 2.4 Hz), 5.71 (d, *J* = 10.4 Hz)]. An oxygenated methylene and an oxygenated methine were proposed by their characteristic carbons at *δ*_C_ 77.5 (C-13) and 78.5 (C-10'), as well as protons at *δ*_H_ 4.56 (1H, d, *J* = 9.6 Hz, H-13a), 3.88 (1H, d, *J* = 9.6 Hz, H-13b)], and 3.36 (1H, brd, *J* = 9.6 Hz, H-10').

The ^1^H-^1^H COSY correlations of H_2_-2/H-1/H-10/H_2_-9(H_3_-14)/H_2_-8/H-7 and the main HMBC correlations of H-5 with C-2/C-3/C-4/C-6, of H_3_-15 with C-3/C-4, of H_2_-2 with C-4/C-5/C-6/C-10, of H_3_-14 with C-1/C-9/C-10, of H-7 with C-6/C-11/C-12/C-13, and of H_2_-13 with C-6/C-7/C-12 (Fig. S3), led to the assignment of a rearranged *seco*-cadinane-type sesquiterpenoid scaffold [namely 5(4→3)-*abeo*-5,6-*seco*-cadinane] with a ketal at C-6 (moiety A). Besides, the other ^1^H-^1^H COSY correlations of H-1'/H-2'/H-3' and H_2_-5'/H_2_-6'/H-1'/H-7'(H_3_-14')/H_2_-8'/H_2_-9'/H-10', together with the HMBC correlations from H_2_-15' to C-3'/C-4'/C-5', from H_3_-14' to C-1'/C-7'/C-8', from H_3_-13' to C-10'/C-11'/C-12', and from H_3_-12' to C-10'/C-11'/C-13', constructed a bisabolane-type sesquiterpenoid scaffold (moiety B). The moieties A and B were linked by a spiro-lactone ring, which was supported by the HMBC correlations from H_2_-13 to C-15' and H_2_-15' with C-7/C-11/C-12/C-13, along with analysis of the chemical shifts of C-12 (*δ*_C_ 176.9) and C-4' (*δ*_C_ 81.9) as well as the remained one degree of unsaturation. Therefore, the planar structure of compound **4** with a congested 6/6/5/5/6 pentacyclic system was established.

The partial relative stereochemistry of compound **4** could be deduced from the ROESY correlations of H-1 with H-7/H_3_-14, of H-7 with H-3'/H_2_-15', and of H-1' with H_3_-14'. The *E*-configuration of the Δ^3,5^-double bond was explained by the ROESY correlation of H-5 with H_3_-15. Furthermore, the coupling constant between H-2' and H-3' (*J*_2',3'_ = 10.4 Hz) supported the *Z*-configuration of the Δ^2'^-double bond.

The stereochemistry of the bisabolane-type sesquiterpenoid with 3,10,11-triol was characterized by analyzing NMR spectrum^1,2^. Generally, if the chemical shifts of the olefinic carbons (C-2' and C-3') show difference ranging from Δ*δ* 3.1 to 5.2 ppm, the relative configurations of C-1' and C-4' are *trans* (1'*S*,4'*R*) or (1'*R*,4'*S*), while the difference between Δ*δ* 0.1 and 1.7 ppm indicates *cis* (1'*S*,4'*S*) or (1'*R*,4'*R*) configuration. The relative configuration of C-7' is related to C-1' and concluded by comparing their ^1^H and ^13^C NMR data. When the chemical shifts of C-14' and H-14' are lower than *δ*_C_ 16.0 and *δ*_H_ 0.90, the relative stereochemistry at C-1' and C-7' are 1'*S*,7'*R*(1'*R*,7'*S*)-form, and conversely, it is 1'*S*,7'*S*(1'*R*,7'*R*)-form. The stereochemistry of C-10' is resolved by interpreting proton splitting patterns of H-10'. If the splitting pattern of H-10' is doublet peak (d), the relative configuration of C-10' is *R*-form, while the doublet-doublet peak splitting (dd) indicates the *S*-form. From the above analyses and the ROESY correlations, the *trans* (1'*S^*^*,4'*S^*^*) configuration was assigned for compound **4** by the chemical shifts of C-2' (*δ* 130.2) and C-3' (*δ* 134.9). Meanwhile, considering the chemical shifts of C-14' (*δ*_C_ 15.7) and H-14' (*δ*_H_ 0.88) as well as the doublet-like splitting of H-10', the absolute configuration of C-7' and C-10' was determined to be 7'*R*,10'*R*. However, it is not clear whether the stereochemistry at C-6 is **4a** (1*S*,6*S*,7*S*,10*R*,11*R*,1'*S*,4'*S*,7'*R*,10'*R*) or **4b** (1*S*,6*R*,7*S*,10*R*,11*R*,1'*S*,4'*S*,7'*R*,10'*R*). Finally, the calculated ECD spectrum of (1*S*,6*S*,7*S*,10*R*,11*R*,1'*S*,4'*S*,7'*R*,10'*R*)-configuration (**4a**) was proved to be in good accordance with the experimental one (Fig. S5), affording the absolute configuration of **4**.

Artemeriopodin B2 (**5**), colorless oils, shared the same molecular formula of C_30_H_44_O_7_ as **4** from its HRESIMS data (*m/z* 517.3158 [M + H]^+^). The ^1^H and ^13^C NMR data of compound **5** was very similar to those of **4**, indicating that compound **5** was also a sesquiterpenoid dimer consisting of bisabolane-type and *seco*-cadinane-type. The major differences were that the methyl at C-4 in the moiety A of compound **4** was migrated to C-5 in **5**, and meanwhile, a formyl group appeared in compound **5**. The HMBC correlations of H-4 with C-2/C-3/C-5 and of H_3_-15 with C-3/C-5 demonstrated the above assignment. The similar ROESY correlations of H-1 with H-1 with H-7/H_3_-14, of H-7 with H-3'/H_2_-15', of H-1' with H_3_-14' revealed that compounds **5** and **4** shared the same relative configurations. Similar with **4**, the Δ^3,5^-double bond was *E*-form, which was manifested by the ROESY correlation of H-4 and H_3_-15. The coupling constant between H-2' and H-3' (*J*_2',3'_ = 10.2 Hz) allowed the *Z*-configuration of the Δ^2'^-double bond. Accordingly, the absolute configuration of compound **5** was elucidated as 1*S*,6*S*,7*S*,10*R*,11*R*,1'*S*,4'*S*,7'*R*,10'*R* through comparing the similarly experimental ECD spectra between **5** and **4**.

Artemeriopodin C1 (**6**) was assigned to have a molecular formula of C_29_H_40_O_4_ on the basis HRESIMS data at *m/z* 453.3001 [M + H]^+^ (calcd. for C_29_H_41_O_4_, 453.2999), indicating ten indices of hydrogen deficiency. The ^13^C NMR (DEPT) spectra of **6** (Table S1) showed 29 carbon signals, ascribing to four methyls, ten methylenes, eight methines (including an olefinic carbon at *δ*_C_ 119.9 and an oxygenated one at *δ*_C_ 58.9), and seven quaternary carbons (including a keto-carbonyl at *δ*_C_ 212.2, an ester carbonyl at *δ*_C_ 173.2, three olefinic carbons at *δ*_C_ 165.3, 136.3 and 122.9, and two oxygenated ones at *δ*_C_ 83.1 and 57.2). The ^1^H NMR spectrum (Table S3) displayed two singlet methyls at *δ*_H_ 1.57 (3H, s) and 1.37 (3H, s), two doublet methyls at *δ*_H_ 0.94 (3H, d, *J* = 6.6 Hz) and 0.88 (3H, d, *J* = 6.0 Hz), as well as an olefinic methine proton at *δ*_H_ 4.74 (1H, brs).

The proton spin systems of H-5/H-6/H-7/H_2_-8/H_2_-9/H-10/H-1/H_2_-2/H_2_-3 and H-6/H-1/H-10/H_3_-14 were obviously recognized from the ^1^H-^1^H COSY correlations of **6**. In the HMBC spectrum, the key correlations from H_3_-14 to C-1/C-9/C-10, from H_3_-15 to C-3/C-4/C-5, from H-5 to C-1/C-3/C-7, and from H-7 to C-1/C-11/C-13, established a 12-*nor*-cadinane sesquiterpenoid moiety (moiety A). Similarly, the ^1^H-^1^H COSY correlations of H_2_-3'/H_2_-2'/H-1'/H-10' and H_2_-8'/H_2_-9'/H-10'/H_3_-14', along with the key HMBC correlations of H_3_-14' with C-1'/C-9'/C-10', of H_3_-15' with C-3'/C-4'/C-5', of H-5' with C-1'/C-6'/C-7', of H_2_-8' with C-6'/C-7'/C-11', and of H_2_-13' with C-7'/C-11'/C-12', constructed a cadinane-type sesquiterpenoid moiety with an *α*,*β*-unsaturated lactone and an ethylene oxide unit (moiety B). The direct linkage of the above two moieties *via* C‒C single bond between C-13 and C-13' was proved by the ^1^H-^1^H COSY correlation of H_2_-13/H_2_-13' and the HMBC correlations of H_2_-13 with C-11' and of H_2_-13' with C-11. Thus, the planar structure of **6** was ascertained.

In the ROESY spectrum, the cross-peaks of H-1/H_3_-14, H-1/H-7, and H-6/H-10, disclosed the relative configuration of the moiety A. For the moiety B, the obvious correlations of H-1'/H_3_-14' and H-5'/H_3_-15' were observed in the ROESY spectrum. Taking the above analyses into consideration, two possible isomers, 1*S*,6*S*,7*R*,10*R*,1'*S*,4'*R*,5'*R*,6'*S*, 10'*R* or 1*S*,6*S*,7*R*,10*R*,1'*R*,4'*S*,5'*S*,6'*R*,10'*S*, were proposed for **6**. By the aid of ECD calculation, the absolute configuration of compound **6** was assigned as 1*S*,6*S*,7*R*,10*R*,1'*S*,4'*R*,5'*R*,6'*S*,10'*R*.

Artemeriopodin C2 (**7**) was determined to have a molecular formula of C_29_H_40_O_7_ (*m/z* 501.2853 [M + H]^+^). The ^1^H and ^13^C NMR data of compound **7** closely resembled those of **6**, and the major difference was that the double bond between C-4/C-5 in compound **6** (*δ*_H_ 4.74; *δ*_C_ 136.3, 119.9) disappeared in **7**, while two carbonyls (*δ*_C_ 209.3, 177.8) occurred, which was implied that compound **7** might be the oxi-cracking product of **6**. The HMBC correlations from H_3_-15 to C-3/C-4, from H-1 to C-5, and from H-7 to C-5 verified the above assignment. The nearly identical ROESY correlation and 1D NMR data indicated that compounds **7** and **6** had the same relative configuration in the moiety B. In the moiety A, the characteristic coupling constants of H-6 with H-1/H-7 (*J*_H-1/H-6_ = *J*_H-6/H-7_ = 11.4 Hz) and the key ROESY correlations of H-1 with H-7/H_3_-14 and of H-6 with H-10 disclosed the same orientations of H-1, H-7, and H_3_-14, but opposite to H-6. By the ECD calculation of two possible configurations, the absolute configuration of compound **7** was finally established as 1*S*,6*R*,7*R*,10*R*,1'*S*,4'*R*,5'*R*,6'*S*,10'*R*.

Artemeriopodin C3 (**8**) had a molecular formula of C_29_H_39_NO_5_ as determined from its HRESIMS data (*m/z* 482.2895 [M + H]^+^), responding 11 degrees of unsaturation. Detailed analysis of its 1D and 2D NMR spectra revealed that compound **8** contained the same cadinane sesquiterpenoid moiety with an *α*,*β*-unsaturated lactone and an ethylene oxide unit (moiety B) as **7**. The remained 14 carbons were established as a 12-*nor*-cadinane-type sesquiterpenoid moiety with an *α*,*β*-unsaturated-γ-lactam ring (moiety A), which was supported by the ^1^H-^1^H COSY correlations of H_2_-3/H_2_-2/H-1/H-10//H_3_-14, H_2_-8/H_2_-9/H-10, and H-11/H_2_-13, together with the HMBC correlations from H_3_-15 to C-3/C-4, from H_3_-14 to C-1/C-9/C-10, from H-1 to C-6/C-7, from H_2_-8 to C-6/C-7/C-11, and from H-11 to C-5/C-6/C-7. The relative configuration of compound **8** in the moiety B was consistent with those of **6** and **7**, which was corroborated by the closely similar NMR data and the ROESY correlation of H-1'/H-7' and H-1'/H_3_-14'. The key ROESY correlations between H-1 and H_3_-14, between H-8a with H-10/H-13b, between H-11 with H-8b ascertained the relative configuration of the moiety A in compound **8**. Thus, the absolute stereochemistry of compound **8** was elucidated as 1*S*,10*R*,11*R*,1'*S*,4'*R*,5'*R*,6'*S*,10'*R* by the high match between the experimental and calculated ECD spectra.

Artemeriopodin C4 (**9**) had the same molecular formula of C_29_H_39_NO_5_ with compound **8** deduced from its HRESIMS data (*m/z* 482.2882 [M + H]^+^). Comparison of the NMR spectra revealed that compounds **9** and **8** were a pair of epimers with the main difference of the chemical shifts at C-5 (*δ*_C_ 164.3 *vs* 174.6) and C-11 (*δ*_C_ 58.2 *vs* 57.9) in the ^13^C NMR spectrum. Analyses of their 2D NMR spectra indicated that the planar structure of compound **9** was identical with that of compound **8**. Therefore, the differences between their NMR data should be derived from configurational variations. In the ROESY spectrum, the correlations of H-1/H_3_-14, H-8a/H-10, H-1'/H-7', and H-1'/H_3_-14' implied that the relative stereochemistry of compound **9** at chiral centers C-1, C-10, C-1', C-4', C-5', C-6', and C-10' was consistent with those of **8**. However, the ROESY correlation of H-8a/H-11 was observed in compound **9**, instead of the correlation of H-11/H-8b and H-8a/H-13b observed in compound **8**, which explained the different stereochemistry between both structures. Accordingly, the absolute configuration of compound **9** was assigned as 1*S*,10*R*,11*S*,1'*S*,4'*R*,5'*R*,6'*S*,10'*R* by comparing the calculated ECD curves with the experimental one.

Artemeriopodin D (**10**) possessed a molecular formula of C_30_H_40_O_7_ based on the HRESIMS data (*m/z* 513.2844 [M + H]^+^, calcd. for C_30_H_41_O_7_, 513.2847). Detailed inspection of the 1D and 2D NMR spectra of compounds **10** with **6**–**9** revealed that they shared the common cadinene sesquiterpenoid moiety with the *α*,*β*-unsaturated-*γ*-lactonic ring and ethylene oxide (moiety B). The remained 15 carbon resonances were clarified as two methyls [*δ*_C_ 19.6 (C-14), 30.0 (C-15); *δ*_H_ 0.95 (3H, d, *J* = 6.0 Hz, H-14), 2.15 (3H, s, H-15)], an *α*,*β*-carbonyl [*δ*_C_ 128.2 (C-13), 141.8 (C-11), 169.8 (C-12); *δ*_H_ 6.29 (1H, s, H-13a), 5.65 (1H, s, H-13b)], two saturated keto-carbonyls [*δ*_C_ 209.0 (C-4), 208.3 (C-5)), four methylenes, and four methines. A 4,5-*seco*-cadinane sesquiterpenoid scaffold (moiety A) was deduced by the ^1^H-^1^H COSY correlations of H_2_-3/H_2_-2/H-1/H-6/H-7/H_2_-8/H_2_-9/H-10/H-1(H_3_-14) and the HMBC correlations from H_3_-14 to C-1/C-9/C-10, from H_3_-15 to C-3/C-4, from H-6 to C-5/C-11, from H-7 to C-1/C-5/C-11/C-12/C12, and from H_2_-13 to C-7/C-11/C-12. The moiety A was directly linked to moiety B by C-C single bond between C-5 and C-13', which was confirmed by the HMBC correlations of H_2_-13' with C-5/C-6. The characteristic coupling constants of H-6 with H-1/H-7 (*J*_H-1/H-6_ = *J*_H-6/H-7_ = 10.8 Hz) indicated their axial orientations. In the ROESY spectrum, the key correlations of H-1 with H-7/H_3_-14, of H-6 with H-10, of H-1' with H_3_-14', and of H-5' with H_3_-15', determined the relative configuration of compound **10**. By comparison of its experimental and calculated ECD spectra, the absolute configuration of compound **10** was elucidated as 1*S*,6*R*,7*R*,10*R*,1'*S*,4'*R*,5'*R*,6'*S*,10'*R*.

Artemeriopodin E (**11**) had a molecular formula of C_30_H_42_O_6_ deduced from the HRESIMS at *m/z* 521.2865 [M + Na]^+^ (calcd. for C_30_H_42_O_6_Na, 521.2874) with ten degrees of unsaturation. The ^13^C NMR data exhibited 30 carbon resonances, corresponding to four methyls, eleven methylenes (including an olefinic one at *δ*_C_ 114.0), seven methines, and eight quaternary carbons (including five oxygenated ones at *δ*_C_ 60.7, 74.1, 80.5, 84.5 and 87.0), an olefinic one at *δ*_C_ 139.4, and two carbonyls at *δ*_C_ 170.6, 210.8). Its ^1^H NMR spectrum (Table S4) clearly suggested four secondary methyls [*δ*_H_ 0.76 (3H, d, *J* = 6.0 Hz), 0.86 (3H, d, *J* = 6.6 Hz), 0.98 (3H, d, *J* = 6.6 Hz), 1.00 (3H, d, *J* = 6.6 Hz)], and two olefinic protons [*δ*_H_ 5.35 (1H, d, *J* = 3.6 Hz), 6.15 (1H, d, *J* = 3.6 Hz)]. The above spectral evidence indicated that **11** might be a highly oxygenated sesquiterpenoid dimer.

A cadinane-type sesquiterpenoid scaffold (moiety A) existed in compound **11**, which was supported by the ^1^H-^1^H COSY relationships of H_2_-3/H_2_-2/H-1/H-6(H-10), H_2_-8/H_2_-9/H-10/H_3_-14, and H_3_-12/H-11/H_3_-13, and the key HMBC correlations from H_3_-14 to C-1/C-9/C-10, from H-1 to C-5/C-7, from H_2_-15 to C-3/C-4/C-5, from H-6 to C-5/C-7, from H_2_-8 to C-6/C-7, from H_3_-12 to C-7/C-11/C-13, and from H_3_-13 to C-7/C-11/C-12 (Fig. S3). Likewise, the another cadinane sesquiterpenoid moiety (moiety B) was assigned by the ^1^H-^1^H COSY correlations of H-7'/H_2_-8'/H_2_-9'/H-10'/H-1'(H_3_-14')/H_2_-2'/H_2_-3' and the HMBC correlations of H_2_-13' with C-7'/C-11'/C-12', of H_3_-14' with C-1'/C-9'/C-10', of H-1' with C-5'/C-6'/C-7', and of H_2_-15' with C-3'/C-4'/C-5'. The moiety B possessed a five-membered lactone ring, verified by the down-fielded chemical shifts at C-6' and C-12'. In addition, the ^1^H-^1^H COSY relationship of H_2_-15/H_2_-15' and the HMBC correlations of H_2_-15' with C-4/C-5' proposed the direct connection of the moieties A and B through C-15 and C-15' single bond. However, the molecular formula based on this 2D structure was incompatible with the HRESIMS data, indicating the presence of two ring according to the index of hydrogen deficiency. The chemical shifts of C-4 (*δ*_C_ 80.5), C-4' (*δ*_C_ 60.7), and C-5' (*δ*_C_ 87.0), and the HRESIMS data of compound **11** obviously proposed the presence of 4,5'-epoxy and 4',5'-epoxy structural moieties. Therefore, the planar structure of compound **11** was defined as a sesquiterpenoid dimer featuring a novel 6/6/6/3/6/6/5 heptacyclic fused ring system.

The assignment of the relative configuration for compound **11** was accomplished based on the coupling constant and ROESY spectrum. The small coupling constant between H-1 and H-6 allowed their *cis*-configurations. The ROESY correlations of H-1/H_3_-14, H-6/H-15b, H-1'/H-7', H-1'/H_3_-14', and H-15'a/H-3'a were determined the relative configuration. Based on the above relative configuration, the two possible isomers, **11a** (1*S*,4*R*,6*S*,7*R*,10*R*,1'*S*,4'*R*, 5'*R*,6'*S*,7'*S*,10'*R*) or **11b** (1*S*,4*R*,6*S*,7*R*,10*R*, 1'*S*,4'*S*,5'*S*,6'*S*,7'*S*,10'*R*) were proposed for **11**. The absolute stereochemistry was defined as 1*S*,4*R*,6*S*,7*R*,10*R*,1'*S*,4'*R*,5'*R*,6'*S*,7'*S*,10'*R* by comparison of calculated and experimental ECD spectra.

Artemeriopodin F1 (**12**) was obtained as white monoclinic crystals. Its molecular formula was deduced to be C_30_H_40_O_8_ by HRESIMS data (*m/z* 527.2650 [M ‒ H]^+^, calcd. for C_30_H_39_O_8_, 527.2650), indicating eleven degrees of unsaturation. Its ^13^C NMR spectrum showed 30 carbon resonances, corresponding to three methyls, ten methylenes (including an olefinic one at *δ*_C_ 118.9), nine methines (including an oxygenated carbon at *δ*_C_ 78.3), and eight quaternary carbons (including one keto-carbonyl at *δ*_C_ 210.7, two ester carbonyls at *δ*_C_ 174.0 and 170.9, an olefinic one at *δ*_C_ 142.8, and three oxygenated ones at *δ*_C_ 82.4, 86.2 and 97.3). The ^1^H NMR spectrum (Table S4) contained three methyls [*δ*_H_ 0.85 (3H, d, *J* = 6.0 Hz), 0.98 (3H, d, *J* = 6.6 Hz), 2.17 (3H, s)], an oxygenated methine proton at *δ*_H_ 4.37 (1H, ddd, *J* = 11.4, 7.8, 4.2 Hz), and olefinic methylene proton at *δ*_H_ 6.13 (1H, d, *J* = 1.8 Hz) and 5.54 (1H, d, *J* = 1.2 Hz). The above information suggested compound **12** to be a highly oxygenated sesquiterpenoid dimer.

The ^1^H-^1^H COSY correlations of H-1/H_2_-2/H-3/H-5/H-6/H-7/H_2_-8/H_2_-9/H-10/H_3_-14 and H-6/H-1, along with the HMBC correlations from H_3_-14 to C-1/C-9/C-10, from H_3_-15 to C-3/C-4, from H-5 to C-4/C-7, from 5-OH to C-5/C-6, from H-7 to C-11/C-12/C-13, and H_2_-13 to C-7/C-11/C-12, established a rearranged cadinene-type sesquiterpenoid moiety (moiety A). Similarly, the coupling relationship of H_2_-3'/H_2_-2'/H-1'/H-10'/H_2_-9'(H_3_-14')/H_2_-8'/H-7' in the ^1^H-^1^H COSY spectrum, as well as the key HMBC correlations from H_3_-14' to C-1'/C-9'/C-10', from H-1' to C-5'/C-6'/C-7', from H_2_-15' to C-3'/C-4'/C-5', from H_2_-3' to C-4'/C-5', from 5'-OH to C-5'/C-6', from H-7' to C-5'/C-6'/C-11'/C-12'/C-13', and from H_2_-13' to C-7'/C-11'/C-12', completed a cadinane-type sesquiterpenoid moiety with a five-membered lactone (moiety B). The linkage of the above two sesquiterpenoid moieties through a rare fused 5,7-dioxabicyclo[2.2.2]octan-6-one ring system was confirmed by the ^1^H-^1^H COSY correlation of H_2_-13/H_2_-15' and the HMBC correlation of H_2_-13 with C-4', combined with the HRESIMS data, the degree of unsaturation, and the chemical shifts at C-4', C-5', and C-11. Accordingly, the gross structure of **12** was established.

The relative configuration of compound **12** was deduced by the coupling constants and ROESY correlation. The large coupling constants of H-1/H-6 and H-6/H-7 (*J*_H-1/H-6_ = *J*_H-6/H-7_ = 10.8 Hz) indicated the *anti*-axial orientation of H-6 with H-1 and H-7. The ROESY correlations between H-1 with H-5/H-7/H_3_-14, of H-6 with H-3/H-10/5-OH, of H-1' with H_3_-14', and of H-7' with 5'-OH (Fig. S4), explained the partially relative configurations. However, the above-mentioned evidence did not sufficiently confirm the stereochemistry of compound **12**. Thus, a single X-ray crystallographic analysis (Fig. S4) was performed, which not only verified the deduced planar structure of compound **12** but also determined its absolute stereochemistry as 1*S*,3*R*,5*R*,6*R*,7*R*,10*R*,11*S*,1'*S*,4'*S*,5'*S*,6'*S*,7'*S*,10'*R* [Flack parameter = 0.02(4)].

Artemeriopodin F2 (**13**) was determined to have a molecular formula of C_30_H_38_O_7_ (*m/z* 511.2699 [M + H]^+^) from the HRESIMS data, less a H_2_O than compound **12**. Comparison of the NMR data of compounds **13** and **12** (Tables 1 and 4) revealed that they shared very similar structures. The major differences between them were that the two methines at C-3 and C-5 of **12** (*δ*_H_ 2.74, 4.37; *δ*_C_ 58.1, 78.3) were absent and replaced by a pair of olefinic carbons (*δ*_H_ 6.66; *δ*_C_ 144.6, 147.0) in **13**, which was indicated that compound **13** should be a dehydrated product of **12**. This inference was agreement with its molecular formula and further supported by the HMBC correlations of H-5 with C-1/C-4/C-7. Detailed analyses of the ROSEY spectrum (Fig. S3) verified that compound **13** maintained the identical relative stereochemistry with **12** at all chiral centers except for C-3 and C-5. Subsequently, the absolute configuration of compound **13** was determined as 1*S*,6*S*,7*R*,10*R*,11*S*,1'*S*,4'*S*,5'*S*,6'*S*, 7'*S*,10'*R* by the high match between the experimental and calculated ECD spectra (Fig. S5).

Artemeriopodin F3 (**14**), white orthorhombic crystals, was deduced to have a molecular formula of C_30_H_42_O_7_ as determined by its HRESIMS data at *m/z* 515.2999 [M + H]^+^ (calcd. for C_30_H_43_O_7_, 515.3003). The ^1^H and ^13^C NMR data of compound **14** (Tables S4 and S5) closely resembled those of arteannoide A, a cadinane-type sesquiterpenoid dimer reported from *A. annua*^3^, and the major differences were that the double bond between C-3 (*δ*_C_ 144.5) and C-5 (*δ*_H_ 7.13; *δ*_C_ 150.7) in arteannoide A was replaced by an oxygenated methine [*δ*_H_ 4.23 (1H, dd, *J* = 9.0, 6.6 Hz, H-5); *δ*_C_ 75.6 (C-5)] and methine [*δ*_H_ 2.99 (1H, ddd, *J* = 12.0, 6.0, 3.0 Hz, H-3); *δ*_C_ 56.5 (C-3)] in **14**. The ^1^H-^1^H COSY correlation of H-3/H-5/H-6 and the HMBC correlations from H-5 to C-4/C-7 verified the above assignment and the C-5 location of the hydroxyl group. The characteristic coupling constants between H-6 with H-1/H-5/H-7 (*J*_H-1/H-6_ = *J*_H-5/H-6_ = *J*_H-6/H-7_ = 10.2 Hz) implied the *anti*-axial orientations of H-6 with H-1, H-5, and H-7. Similarly, the axial orientations of H-1', H-6', and H-7' were supported by the coupling constants of *J*_H-1'/H-6'_ (10.2 Hz) and *J*_H-6'/H-7'_ (10.2 Hz). In the ROESY spectrum, the key correlations between H-1 and H-5/H-7/H_3_-14, between H-6 and H-3/H-10/H-13a, between H-1' and H-7'/H_3_-14', as well as between H-6' and H-4'/H-10', assigned the relative configuration of compound **14**. The structure of **14** was confirmed using a single crystal X-ray diffraction experiment with Cu K*α* radiation [Flack parameter = 0.02(4)], and the absolute configuration was elucidated as 1*S*,3*R*,5*R*,6*R*,7*R*,10*R*,11*R*,1'*S*,4'*S*,5'*S*,6'*R*,7'*R*,10'*R*.

Artemeriopodin F4 (**15**) had the same molecular formula of C_30_H_42_O_7_ with compound **14** by their HRESIMS data (*m/z* 515.2999 [M + H]^+^). Detailed inspection of the 1D and 2D NMR data of compounds **14** and **15** revealed that they shared the same planar structure, and the only NMR difference occurred at H-3/C-3 and the concurrent chemical shift changes surrounding C-3 position, which implied they were a pair of C-3 epimers. Just like compound **14**, the large coupling constants between H-6 and H-1/H-5/H-7 (*J*_H-1/H-6_ = *J*_H-5/H-6_ = *J*_H-6/H-7_ = 10.8 Hz) illustrated the *anti*-axial orientations of H-6 with H-1, H-5, and H-7 in **15**. The ROESY correlations of H-1 with H-3/H-5/H-7/H_3_-14, of H-6 with H-10/H-13a, of H-1' with H-7'/H_3_-14', and of H-6' with H-4'/H-10', interpreted the relative configuration of compound **15**, and verified that compound **15** was the epimer of **14** at C-3 position. The absolute configuration of **15** was determined as 1*S*,3*S*,5*R*,6*R*,7*R*,10*R*,11*R*,1'*S*,4'*S*,5'*S*,6'*R*,7'*R*,10'*R* by the high agreement between the experimental and calculated ECD spectra.

Artemeriopodin F5 (**16**), white orthorhombic crystals, was proposed a molecular formula of C_30_H_44_O_5_ by HRESIMS (*m/z* 483.3145 [M ‒ H]^‒^). The resemblance of the NMR data of compound **16** (Tables S5 and S6) with those of arteannoide A suggested their similar structures^3^. The obvious difference was that the *α*,*β*-unsaturated carboxylic acid at C-7' in arteannoide A was replaced by an isopropyl group [*δ*_H_ 1.87 (1H, m), 1.02 (3H, d, *J* = 6.0 Hz), 0.83 (3H, d, *J* = 6.6 Hz); *δ*_C_ 34.8, 17.1, 16.8] in compound **16**, and an additional hydroxyl group occurred at C-7' [*δ*_C_ 75.3]. This deduction was verified by the spin-coupled fragments of H_3_-12'/H-11/H_3_-13' in the ^1^H-^1^H COSY spectrum and the correlations from H_3_-13' to C-7'/C-11'/C-12' and from H_3_-12' to C-7'/C-11'/C-13' in the HMBC spectrum. The coupling constants of H-1/H-6 (*J* = 10.8 Hz) and H-6/H-7 (*J* = 10.8 Hz) proposed that H-1 and H-7 were the homolateral axial-orientations, but opposite to H-6. The small coupling constant of H-1'/H-6' suggested their *cis*-configurations. In the ROESY spectrum, the key correlations of H-1 with H-7/H_3_-14 and of H-6 with H-10, permitted the *α*-orientations of H-1, H-7, and H_3_-14, as well as the *β*-orientation of H-6. Similarly, H-1', H-4', H-6', and H_3_-14' were positioned in the *α*-orientations by the ROESY correlations of H-1'/H_3_-14' and H-4'/H-6'. Thus, the relative configurations of partially chiral centers within moieties A and B were established. However, the NOESY experiment could not provide any useful information for relative configurational determination at C-11, C-5', and C-7'. It is delightful that a qualified crystal suitable for a single-crystal X-ray diffraction experiment was obtained from a mixture solvent system (MeOH-H_2_O). As a result, the relative and absolute stereochemistry of compound **16** was unambiguously defined as 1*S*,6*S*,7*R*,10*R*,11*R*,1'*S*,4'*S*,5'*S*,6'*S*,7'*R*,10'*R* by single crystal X-ray diffraction [Flack parameter = 0.04(4)].

Artemeriopodin F6 (**17**), white monoclinic crystals, manifested a molecular formula of C_30_H_38_O_7_ by its HRESIMS data (*m/z* 533.2505 [M + Na]^+^, calcd. for C_30_H_38_O_7_Na, 533.2510). The ^1^H and ^13^C NMR data of compound **17** resembled those of arteannoide A,^3^ and the major differences were that two oxygenated quaternary carbons at C-4' (*δ*_C_ 69.4) and C-6' (*δ*_C_ 85.8) in compound **17** appeared instead of two methines [*δ*_H_ 1.71 (H-4'), 2.28 (H-6'); *δ*_C_ 40.3 (C-4'), 47.4(C-6')] in arteannoide A. The above deduction was confirmed by the HMBC correlations of H_2_-8'/H-10' with C-6' and of H_2_-13/H_2_-15' with C-4'. Meanwhile, a five-membered lactone ring was proposed between C-6' and C-11' by considering a remained degree of unsaturation in **17** and the downfielded chemical shift of C-6' (*δ*_C_ 85.8). The partial relative configurations of compound **17** could be deduced by the ROESY correlations of H-1/H-7, H-1/H_3_-14, H-6/H-10, H-1'/H-7', and H-1'/H_3_-14'. Fortunately, a signal crystal was obtained from mixture solvent of MeOH-H_2_O and performed to the Cu K*α* radiation. The absolute configurations of compound **17** was then established as 1*S*,6*S*,7*R*,10*R*,11*R*,1'*S*,4'*R*, 5'*S*,6'*S*,7'*S*,10'*R* [Flack parameter = 0.04(4)].

Artemeriopodin F7 (**18**) was gave a molecular formula of C_29_H_38_O_7_ based on the HRESIMS data (*m/z* 499.2701 [M + H]^+^). The resemblance between the NMR data of compounds **18** and **17** indicated both similar structures, but obvious changes existed in the five-membered lactone ring of the moiety B (Tables 5 and 6). The *α*,*β*-unsaturated ester group [*δ*_C_ 140.0 (C-11'), 121.5 (C-13'), 168.4 (C-12'); *δ*_H_ 6.17 (1H, d, *J* = 1.2 Hz, H-13'a), 5.43 (1H, s, H-13'b)] in compound **17** was absent, while a keto-carbonyl (*δ*_C_ 211.4, C-11') and an oxygenated methylene [*δ*_C_ 69.4 (C-13'); *δ*_H_ 4.19 (2H, d, *J* = 2.4 Hz, H-13')] appeared in compound **18**, which was demonstrated by the HMBC correlations from H_2_-13' to C-6'/C-7'/C-11' and from H_2_-8' to C-11'. Hence, compound **18** was identified as a sesquiterpenoid dimer consisting of a 12'-*nor*-cadinane and a 5(4→3)-*abeo*-cadinane to forming the 6,8-dioxabicyclo[3.2.l]octan-7-one. The large coupling constants of H-1/H-6 (*J* = 11.4 Hz) and H-6/H-7 (*J* = 11.4 Hz), along with the ROESY correlations of H-1/H-7, H-1/H_3_-14, H-6/H-10, H-1'/H-7', H-1'/H_3_-14', and 4'-OH/H_2_-13' indicated that the relative configuration of compound **18** maintained a good agreement with that of **17**. By comparing the experimental and theoretically calculated ECD spectra (Fig. S5), the absolute configuration of compound **18** was certified as 1*S*,6*S*,7*R*,10*R*, 11*R*,1'*S*,4'*R*,5'*S*,6'*S*,7'*R*,10'*R*.

Artemeriopodin F8 (**19**) was deduced to have a molecular formula of C_28_H_36_O_6_ from its HRESIMS (*m/z* 469.2591 [M + H]^+^). Detailed interpretation of the 1D and 2D NMR data between compounds **19** and **14** revealed that the major differences were present in the rearranged cadinene-type sesquiterpenoid moiety (moiety A). The ^1^H-^1^H COSY correlation of H-7/H_2_-8/H_2_-9/H-10/H-1(H_3_-14)/H_2_-2 as well as the key HMBC correlations from H-5 to C-1/C-2/C-7, from H_2_-2 to C-3/C-5/C-6, and from H-7/H_2_-13 to C-11/C-12 constructed a rearranged 4,15-*dinor*-cadinene sesquiterpenoid scaffold (moiety A) in **19**. Compounds **19** and **14** maintained the same stereochemistry at C-1, C-7, C-10, C-11, C-1', C-4', C-5', C-6', C-7', and C-10', which was verified by the cross-peak of H-1 with H-7/H_3_-14, between H-1' with H-7'/H_3_-14', and between H-6' with H-4'/H-10' in the ROESY spectrum. Therefore, its absolute configuration was established as 1*S*,7*R*,10*R*,11*R*,1'*S*,4'*S*,5'*S*,6'*R*,7'*R*, 10'*R* by a single-crystal X-ray diffraction experiment [Flack parameter = 0.19(10)], which was further demonstrated by calculated ECD spectrum.

Artemeriopodin F9 (**20**) was determined to have the same molecular formula of C_28_H_36_O_6_ as compound **19** based on their HRESIMS. Comparison of the 1D and 2D NMR data supported compounds **20** and **19** shared the same planar structure (Tables S5 and S6, Fig. S2). The characteristic coupling constant of H-6' and H-7' (10.2 Hz) allowed their *anti*-axial orientations. The key ROESY correlations between H-1 with H-7/H_3_-14, between H-1' with H-4'/H-7'/H_3_-14', and between H-6' with H-10', implied that compound **20** was an epimer of **19** at C-4' position. This assignment was verified by comparing the experimental and calculated ECD data, and thus the absolute configuration of **20** was determined as 1*S*,7*R*,10*R*,11*R*,1'*S*,4'*R*,5'*S*,6'*R*,7'*R*,10'*R*.

Artemeriopodin F10 (**21**) possessed a molecular formula of C_30_H_40_O_7_ on basis of the HRESIMS (*m/z* 513.2856 [M + H]^+^, calcd. for C_30_H_41_O_7_, 513.2547). Comparison of the NMR data of compound **21** with those of **17** suggested that their moiety B was very similar, but an *α*,*β*-unsaturated five-membered lactone ring in **17** was replaced by *α*,*β*-unsaturated carboxyl group and a methine in **20**, which was proved by the ^1^H-^1^H COSY correlation of H_2_-3'/H_2_-2'/H-1'/H-6'(H-10')/H-7'/H_2_-8'/H_2_-9'/H-10'/H_3_-14' and the HMBC correlations from H_2_-13' to C-7'/C-11'/C-12'. The remained 15 carbon resonances were classified into two methyls, four methylenes, five methines (including an olefinic one at *δ*_C_ 152.3, *δ*_H_ 7.24), and four quaternary carbons (including an olefinic one at *δ*_C_ 145.5, an oxygenated one at *δ*_C_ 84.5, two carbonyls at *δ*_C_ 200.9 and 175.5). Three spin-coupling structural segments of H_2_-2/H-1/H-6/H-5, H-6/H-7/H_2_-8/H_2_-9/H-10/H_3_-14, and H-1/H-10 in ^1^H-^1^H COSY spectrum, together with the HMBC correlations from H_3_-14 to C-1/C-9/C-10, from H_3_-15 to C-3/C-4/C-5, from H-5 to C-1/C-3/C-4/C-15, from H_2_-2 to C-3/C-4/C-6, from H-7 to C-11/C-12/C-13, and from H_2_-13 to C-7/C-11/C-13, established another cadinene-type sesquiterpenoid moiety (moiety A). Compound **21** featured a fused 6,8-dioxabicyclo[3.2.l]octan-7-one ring system, which was supported by the ^1^H-^1^H COSY correlation of H_2_-13/H_2_-15', the HMBC correlations from H_2_-13 to C-4' and from H_2_-15' to C-11, the degrees of unsaturation, as well as the comparison of the NMR data with those of **14**‒**20**. The coupling constants of H-6 with H-1 and H-7 were both 10.2 Hz, which was permitted that they were appointed at axial orientations. The key ROESY correlations between H-1 with H-7/H_3_-14, between H-6 with H-10, between H-1' with H-7'/H_3_-14', and between H-6' with H-10' suggested that compound **21** kept the consistent relative stereochemistry with **17**. Thus, the absolute configuration of compound **21** was identified as 1*S*,6*S*,7*R*,10*R*,11*R*,1'*S*,4'*R*,5'*R*,6'*R*,7'*R*,10'*R* by comparing the experimental and calculated ECD data, of which the calculated ECD spectrum of **21** matched well with the experimental one.

Artemeriopodin F11 (**22**) was determined to have a molecular formula of C_30_H_38_O_6_ from its HRESIMS (*m/z* 495.2742 [M + H]^+^, calcd. for C_30_H_39_O_6_, 495.2741). Its ^1^H and ^13^C NMR data (Tables S5–S7) were closely similar with those **17**, which was indicated that the structure of compound **21** was closely related to compound **17**. Comparison of compounds **21** and **17** revealed that the ester group (*δ*_C_ 173.0) between C-12 and C-5' in the 6,8-dioxabicyclo[3.2.l]octan-7-one ring system of **17** was hydrolyzed in **22**, and meanwhile, an additional double bond was formed between C-4' (*δ*_C_ 110.6) and C-5' (*δ*_C_144.9). The above inference was demonstrated by the key HMBC correlations of H_2_-13 with C-12/C-4' and of H_2_-15' with C-11/C-4'/C-5'. The increased chemical shifts of C-11 (*δ*_C_ 82.6) and C-5' positions, and its HRESIMS supported the presence of an ether bond between C-11 and C-5'. From the above analyses, the planar structure of compound **22** was determined to be a sesquiterpenoid dimer that two sesquiterpenoid moieties were linked through a dihydropyran ring. The stereochemistry of compound **22** kept consistent with **17** at all chiral centers, which was deduced by the coupling constants of *J*_H-1/H-6_ (10.2 Hz) and *J*_H-6/H-7_ (10.2 Hz), as well as the ROESY correlations of H-1/H-7, H-1/H_3_-14, H-6/H-10, H-1'/H-7', and H-1'/H_3_-14'. As a result, the absolute configuration of compound **22** was established as 1*S*,6*S*,7*R*,10*R*,11*R*,1'*S*, 6'*S*,7'*S*,10'*R* by the high match between the experimental and calculated ECD spectra (Fig. S6).

Artemeriopodin F12 (**23**) was assigned to have a molecular formula of C_30_H_40_O_6_ by its HRESIMS data at *m/z* 497.2891 [M + H]^+^ (calcd. for C_30_H_41_O_6_, 497.2898). Its ^1^H and ^13^C NMR data (Tables S5–S7) resembled those of **13**, implying that compound **23** was constructed by a rearranged cadinene-type (moiety A) and a cadinene-type sesquiterpenoids with the acrylic acid unit at C-7' and the carbonyl group at C-5' (moiety B). The inference was confirmed by the ^1^H-^1^H COSY correlations of H_2_-2/H-1/H-6/H-7(H-5)/H_2_-8/H_2_-9/H-10/H_3_-14, H-7/H-11/H_2_-13, and H_2_-2'/H_2_-3'/H-1'/H-6'/H-7'/H_2_-8'/ H_2_-9'/H-10'/H_3_-14'(H-1'), along with the HMBC correlations from H_3_-14 to C-1/C-9/C-10, from H_3_-15 to C-3/C-4/C-5, from H-5 to C-2/C-3/C-4/C-7, from H_2_-2 to C-3/C-4, from H-7 to C-11/C-12/C-13, from H_3_-14' to C-1'/C-9'/C-10', from H_2_-3' with C-4'/C-5', from H_2_-15' with C-3'/C-4'/C-5', from H-6' to C-4'/C-5', and from H_2_-13' to C-7'/C-11'/C-12'. The two moieties were connected *via* a six-membered spiro-lactone ring, which was ascertained by the ^1^H-^1^H COSY correlations of H_2_-13/H_2_-15' and the HMBC correlations from H_2_-13 to C-4' and from H_2_-15' to C-7, as well as the increased chemical shift at C-4' (*δ*_C_ 90.6). The coupling constants of *J*_H-1/H-6_ (10.2 Hz) and *J*_H-6/H-7_ (10.2 Hz) indicated that H-1, H-6, and H-7 were axially oriented. Similarly, the large coupling constants (*J*_H-1'/H-6'_ = *J*_H-6'/H-7'_ =11.4 Hz) allowed *anti*-axial-orientations of H-6' with H-1' and H-7'. In the ROESY spectrum, the key correlations between H-1 with H-7/H_3_-14 and between H-1' with H-7'/H_3_-14' indicated their *α*-orientations. Meanwhile, the ROESY correlations between H-6 with H-10/H_2_-13, between H-6' with H-2'b/H-10'/H-15'a, and between H-10' with H-6'/H-2'b suggested the *β*-orientations of H-6, H-10, CH_2_(C-13), H-6', H-10', and CH_2_(C-15'). The above-mentioned relative configuration of compound **23** was identical with that of compound **13**. Therefore, the absolute configuration of compound **23** was defined as 1*S*,6*R*,7*R*,10*R*,11*S*,1'*S*,4'*S*,6'*R*,7'*R*,10'*R* by theoretically calculated ECD, in which the calculated ECD spectrum was in good accordance with the experimental one.

Artemeriopodin F13 (**24**), white powders, had a molecular formula of C_32_H_48_O_8_ based on its HRESIMS data (*m/z* 583.3232 [M + Na]^+^, calcd. for C_32_H_48_O_8_Na, 583.3241). Detailed inspection of the 1D and 2D NMR data of compounds **24** and **14** revealed they shared the same sesquiterpenoid moieties A and B. The major differences were that the fused 6,8-dioxabicyclo[3.2.l]octan-7-one ring system in compound **14** was hydrolyzed and a keto-carbonyl (*δ*_C_ 215.2, C-5'), a hydroxy, and an ester-carbonyl (*δ*_C_ 176.3, C-12) appeared in compound **24**. In addition, an ethyoxyl group [*δ*_C_ 62.4 (C-1"), 14.7 (C-2"); *δ*_H_ 4.21 (2H, m, H-1"), 1.31 (3H, t, *J* = 7.2 Hz, H-2")] was distinguished in the ^1^H and ^13^C NMR spectra. The above deduction was confirmed by the ^1^H-^1^H COSY correlations of H-1/H_2_-2/H-3/H-5/H-6/H-7(H-1)/H_2_-8/ H_2_-9/H-10/H-1(H_3_-14) and H_2_-13/H_2_-15'/H-4'/H_2_-3'/H_2_-2'/H-1'/H-6'/H-7'/H_2_-8'/H_2_-9'/H-10'/H_3_-14'(H-1'), together with the HMBC correlations from H_3_-14 to C-1/C-9/C-10, from H_3_-15 to C-3/C-4, from H_2_-13 to C-7/C-11/C-12/C-4', from H_3_-14' to C-1'/C-9'/C-10', from H-4' to C-5'/C-6', from H_2_-15' to C-11/C-4'/C-5', from H-6' to C-5', from H-7' to C-5', and from H_2_-13' to C-7'/C-11'/C-12'. The ethyoxyl group was appointed at C-12 position by the HMBC correlation from H_2_-1 to C-12. The diagnostic coupling constants of H-6 with H-1/H-5/H-7 (*J*_H-1/H-6_ = *J*_H-5/H-6_ = *J*_H-6/H-7_ = 9.6 Hz) and the ROESY correlations of H-1 with H-5/H-7/H_3_-14, of H-6 with H-3/H-10, of H-7 with H-13b, and of H-5 with H-13a, proposed the *α*-orientations of H-1, H-5, H-7, and H_3_-14, but the *β*-orientations of H-3, H-6, H-10, and OH-11. Similarly, the coupling relationships of H-6' with H-1'/H-7' (*J*_H-1'/H-6'_ = *J*_H-6'/H-7'_ = 11.4 Hz) permitted their axial orientations, which was in combination with the ROESY correlations between H-1' with H-7'/H_3_-14' and between H-6' with H-4'/H-10', supported the *α*-orientations of H-1', H-7', and H_3_-14', and the *β*-orientations of H-4', H-6', and H-10'. Based on the relative configuration deduced above, the absolute stereochemistry of compound **24** was identified as 1*S*,3*R*,5*R*,6*R*,7*R*,10*R*,11*S*,1'*S*,4'*S*,6'*R*,7'*R*,10'*R* by the high match between the experimental and calculated ECD spectra.

Artemeriopodin F14 (**25**) had a molecular formula of C_28_H_36_O_6_ on the basis its HRESIMS data at *m/z* 469.2593 [M + H]^+^ (calcd. for C_28_H_37_O_6_, 469.2585). Detailed analysis of ^1^H and ^13^C NMR data indicated that compounds **25** and **24** possessed the same cadinane-type sesquiterpenoid moiety B with the keto-carbonyl group at C-5', which was demonstrated by the ^1^H-^1^H COSY correlations of H_2_-15'/H-4'/H_2_-3'/H_2_-2'/H-1'/H-6'/H-7'/H_2_-8'/H_2_-9'/H-10'/H_3_-14'(H-1') and the HMBC correlations from H_3_-14' to C-1'/C-9'/C-10', from H-4' with C-5'/C-6', from H_2_-15' with C-4'/C-5', from H-6' to C-5', from H-7' to C-5', and from H_2_-13' to C-7'/C-11'/C-12'. Besides, the remained 13 carbon signals proposed a rearranged 4,15-*dinor*-cadinane sesquiterpenoid scaffold with an *α*,*β*-unsaturated lactone ring (moiety A), which was supported by the ^1^H-^1^H COSY relationships of H_2_-2/H-1/H-10/H_2_-9(H_3_-14)/H_2_-8, and the HMBC correlations from H_3_-14 to C-1/C-9/C-10, from H-1 to C-3/C-5/C-6, from H_2_-2 to C-3/C-5/C-6, from H_2_-5 to C-3/C-6/C-7, from H_2_-8 to C-6/C-7/C-11, and from H_2_-13 to C-7/C-11/C-12, as well as the chemical shifts at C-6 (*δ*_C_ 92.2) and C-12 (*δ*_C_ 175.0). The key ^1^H-^1^H COSY correlation of H_2_-13/H_2_-15' verified the direct linkage of the above two moieties through C-13‒C-15' single bond. The relative configuration of compound **25** was deduced according to the coupling constants of *J*_H-1'/H-6'_ and *J*_H-6'/H-7'_ (11.4 Hz) and the cross-peaks of H-1/H_3_-14, H-1'/H-7', H-1'/H_3_-14', H-4'/H-6', and H-6'/H-10' in the ROESY spectrum. Based on the above results, the absolute configuration of compound **25** was determined as 1*S*,6*S*,10*R*,1'*S*,4'*S*,6'*R*,7'*R*,10'*R*, which was demonstrated by comparing the calculated ECD and experimental spectra.

Artemeriopodin F15 (**26**) was deduced to share the same molecular formula of C_28_H_36_O_6_ with **25** by their HRESIMS data. Inspection of its 1D and 2D NMR data revealed that compound **26** possessed the same planar structure with compound **25**. The coupling constants of H-1'/H-6' (*J*_H-1'/H-6'_ = 10.8 Hz) and H-6'/H-7' (*J*_H-6'/H-7'_ = 10.8 Hz) suggested they were axially oriented. The obvious correlations between H-1 with H_3_-14, H-1' with H-7'/H_3_-14', H-6' with H-2b'/H-10'/H-15'b, and H-10' with H-2'b, addressed that compound **26** maintained identically relative stereochemistry with that of **25** except for C-4' position. The above-mentioned information indicated that compound **26** was an epimer of **25** at C-4' position. Therefore, the absolute configuration of compound **26** was elucidated as 1*S*,6*S*,10*R*,1'*S*,4'*R*, 6'*R*,7'*R*,10'*R* by comparing the experimental and calculated ECD spectra.

Artemeriopodin G1 (**27**) was isolated as white triclinic crystals (MeOH-H_2_O). Its molecular formula was established as C_30_H_42_O_7_ according to the positive HRESIMS at *m/z* 515.2997 [M + H]^+^ (calcd. for C_30_H_43_O_7_, 515.3003), requiring ten degrees of unsaturation. The ^13^C NMR (DEPT) data (Table S8) revealed the existence of 30 carbons, ascribing to four methyls at *δ*_C_ 29.6, 23.7, 20.7, and 20.2, nine methylenes (including two olefinic ones at *δ*_C_ 125.6 and 122.1), ten methines (including two oxygenated ones at *δ*_C_ 80.9 and 70.8), and seven quaternary carbons (including a keto-carbonyl at *δ*_C_ 213.0, two ester carbonyls at *δ*_C_ 172.7 and 168.7, two olefinic ones at *δ*_C_ 145.8 and 143.7, and two oxygenated ones at *δ*_C_ 88.9 and 84.1). The ^1^H NMR spectrum (Table S9) displayed two doublet methyls at *δ*_H_ 0.90 (3H, d, *J* = 6.0 Hz, H-14) and 0.98 (3H, d, *J* = 6.6 Hz, H-14'), two singlet methyls at *δ*_H_ 2.22 (3H, s, H-15) and 1.52 (3H, s, H-15'), two pair of exocyclic double bond protons [*δ*_H_ 6.27 (1H, s, H-13a), 5.68 (1H, s, H-13b); 6.08 (1H, d, *J* = 1.2 Hz, H-13'a), 5.65 (1H, d, *J* = 0.6 Hz, H-13'b)], and two oxygenated methines at *δ*_H_ 3.89 (1H, dd, *J* = 9.6, 7.2 Hz, H-5) and 4.21 (1H, s, H-5').

The planar structure of compound **27** was established by the interpretation of 2D NMR spectra, which indicated that it could be divided into two moieties. The spin-coupling systems of H-1/H_2_-2/H-3/H-5/H-6/H-7/H_2_-8/H_2_-9/H-10/H_3_-14 and H-6/H-1/H-10 in ^1^H-^1^H COSY spectrum, along with the HMBC correlations from H_3_-15 to C-3/C-4, from H-5 to C-1/C-2/C-3/C-4/C-6/C-7, from H_3_-14 to C-1/C-9/C-10, and from H_2_-13 to C-7/C-11/C-12 constructed a rearranged cadinane-type sesquiterpenoid scaffold (moiety A), which was identical to artemanin B from *A. annua*^4^. Similarly, the planar structure of another cadinane sesquiterpenoid scaffold (moiety B) was established by the ^1^H-^1^H COSY correlations of H_2_-3'/H_2_-2'/H-1'/H-10'/H_3_-14' and H-7'/H_2_-8'/H_2_-9'/H-10', together with the HMBC correlations from H_3_-14' to C-1'/C-9'/C-10', from H_3_-15' to C-3'/C-4'/C-5', from H-5' to C-1'/C-3'/C-4'/C-6', from H-1' to C-7', from H-7' to C-5'/C-11'/C-12', and from H_2_-13' to C-7'/C-12'. The downfielded chemical shift of the oxygenated carbon (C-6', *δ*_C_ 88.9) revealed that a five-numbered lactone ring was formed between C-6' and C-12'. The moieties A and B were assembled through an ester bond between C-12 and C-4' according to the molecular weight and degrees of unsaturation, of which was also in accordance with the chemical shifts of the two carbons. According to the above analyses, the planar structure of compound **27** was deduced as cadinene-type sesquiterpenoid dimer bearing a 5/6/6/6/5 five ring system.

The relative configuration of **27** was partially determined by the interpretation of proton-proton coupling and ROESY correlations. The large coupling constants of H-6 with H-5 and H-7 (*J*_H-5/H-6_ = 9.6 Hz, *J*_H-6/H-7_ = 12.0 Hz) indicated the *α*-axial orientations of H-5 and H-7, as well as the *β*-axial orientation of H-6. For the moiety A, the key ROESY correlations of H-1 with H-5/H-7/H_3_-14 and of H-6 with H-3/H-10 suggested the *α*-orientations of H-1, H-5, H-7, and H_3_-14, and the *β*-orientations of H-3, H-6, H-10, and 5-OH. Similarly, H-1', H-7', and H_3_-14' were positioned in an *α*-orientations and H-6' was *β*-orientation by the key ROESY correlations of H-1' with H_3_-14'/H-7' and of H-6' with H-10' in the moiety B. However, it was difficult to determine the whole stereochemistry of compound **27**, especially the chiral at C-4', C-5', and C-6' relying on ROESY experiments. Thus, a single X-ray crystallographic analysis was conducted by Cu *Kα* radiation (Fig. S4), which not only confirmed the above deduced planar structure of **27**, but also determined its absolute configurations as 1*S*,3*R*,5*R*,6*R*,7*R*,10*R*, 1'*S*,4'*S*,5'*R*,6'*S*,7'*S*,10'*R* [Flack parameter = 0.20(7)].

Artemeriopodin G2 (**28**) had the same molecular formula of C_30_H_42_O_7_ with compound **27** by the positive HRESIMS ion at *m/z* 515.3005 [M + H]^+^ (calcd. for C_30_H_43_O_7_, 515.3003). Detailed comparison of ^1^H and ^13^C NMR data showed that compounds **28** and **27** were a pair of diastereoisomers, and the main differences involved the chemical shift changes at C-3 (*δ*_C_ 55.9 *vs* 59.2) and C-5 (*δ*_C_ 77.9 *vs* 80.9) in ^13^C NMR spectrum and H-3 (*δ*_H_ 3.20 *vs* 2.85) and H-5 (*δ*_H_ 4.25 *vs* 3.89) in ^1^H NMR spectrum. Analyses of ^1^H-^1^H COSY and HMBC spectra revealed that compounds **28** and **27** shared the same planar structure. Therefore, the differences between their NMR data should be derived from configurational variations. The coupling constants of H-6 with H-5 and H-7 respective were 9.6 Hz (*J*_H-5/H-6_) and 11.4 Hz (*J*_H-6/H-7_), which was supported that H-5, H-6, and H-7 were the axial orientations. The same as compound **27**, the key correlations of H-1 with H-5/H-7/H_3_-14, of H-6 with H-10, of H-1' with H_3_-14'/H-7', and of H-6' with H-10' showed in the ROESY spectrum of compound **28**, meanwhile, H-3 showed obvious correlation with H-1 instead of H-6, which was indicated that compound **28** shared the same relative configurations as compound **27** except for C-3 position. As shown in Fig. S6, the calculated ECD curve matched well with the experimental one, by which the absolute configuration of compound **28** was assigned as 1*S*,3*S*,5*R*,6*R*,7*R*,10*R*,1'*S*,4'*S*,5'*R*,6'*S*,7'*S*,10'*R*.

Artemeriopodin G3 (**29**) had a molecular formula of C_30_H_44_O_7_ as determined according to the HRESIMS *m/z* 539.2974 [M + Na]^+^ (calcd. for C_30_H_44_O_7_Na, 539.2979), responding to nine indices of hydrogen deficiency. The ^1^H and ^13^C NMR data of compound **29** closely resembled those of **27**, the main differences were that a doublet methyl [*δ*_C_ 9.6 (C-13'), *δ*_H_ 1.08 (3H, d, *J* = 7.2 Hz, H-13')] and a methine [*δ*_C_ 40.1 (C-11'), *δ*_H_ 3.11 (1H, m, H-11')] signals in compound **29** replaced a pair of exocyclic double bond at C-11' (*δ*_C_ 143.7) and C-13' [*δ*_C_ 122.1, *δ*_H_ 6.08 (1H, d, *J* = 1.2 Hz, H-13'a), 5.65 (1H, d, *J* = 0.6 Hz, H-13'b)] in compound **27**. The above deduction was confirmed by the ^1^H-^1^H COSY correlation of H_3_-13'/H-11'/H-7'/H_2_-8'/H_2_-9'/H-10'/H_3_-14' and the HMBC correlation from H_3_-13' to C-7'/C-11'/C-12'. The characteristic coupling constants of *J*_H-5/H-6_ = 9.6 Hz and *J*_H-6/H-7_ = 12.0 Hz suggested that H-5 and H-7 had an *α*-axial orientations, while H-6 had a *β*-axial orientation. In the ROESY spectrum, the cross-peaks of H-1/H-5, H-1/H-7, H-1/H_3_-14, H-3/H-6, H-6/H-10, H-1'/H-7', and H-1'/H_3_-14', disclosed that the relative configuration of compound **29** was identical to **27** except for C-11'. However, the correlations of H-7' with both H-11' and H_3_-13' were observed in the ROESY, so the stereochemistry of H-11' could not be explained. Followingly, a single X-ray crystallographic analysis with Cu *Kα* radiation [Flack parameter = 0.4(2)] determined the relative configuration of compound **29**. With the above deduction, the absolute stereochemistry of compound **29** was elucidated as 1*S*,3*R*,5*R*,6*R*,7*R*,10*R*,1'*S*,4'*S*, 5'*R*,6'*S*,7'*S*,10'*R*,11'*R* by the high match between the experimental and calculated ECD spectra.

Artemeriopodin G4 (**30**) displayed the protonated ion ([M + H]^+^) at *m/z* 499.3047 (calcd. for C_30_H_43_O_6_, 499.3054) in positive HRESIMS, suggesting a molecular formula of C_30_H_42_O_6_. Its ^1^H and ^13^C NMR data were closely similar with those of compound **29**, but obvious difference existed in the moiety A. A methine at C-3 (*δ*_C_ 59.2, *δ*_H_ 2.87) and an oxygenated methine at C-5 (*δ*_C_ 80.9, *δ*_H_ 3.87) in compound **29** were replaced by a pair of double bonds at C-3 (*δ*_C_ 145.6) and C-5 (*δ*_C_ 147.7, *δ*_H_ 6.94) in compound **30**, which was proved by the ^1^H-^1^H COSY correlation of H-5/H-6, the HMBC correlations from H-5 to C-1/C-2/C-3/C-4/C-6/C-7, and the upfield chemical shifts at C-4 (*δ*_C_ 197.7 *vs* 213.0). For moiety A, the large coupling constants between H-6 with H-1 and H-7 (*J*_H-1/H-6_ = *J*_H-6/H-7_ = 12.0 Hz), together with the ROESY correlations of H-1 with H-7/H_3_-14 and of H-6 with H-10 proposed the *α*-orientations of H-1, H-7, and H_3_-14, as well as the *β*-orientations of H-6 and H-10. The moiety B of compound **30** shared the same planar structure with that of compound **29**, which was confirmed by the ROESY correlations and NMR data. Accordingly, the absolute configuration of compound **30** was addressed as 1*S*,6*S*,7*R*,10*R*,1'*S*,4'*S*,5'*R*,6'*S*,7'*S*,10'*R*,11'*R* by comparing the calculated ECD curves with the experimental one.

Artemeriopodin G5 (**31**) had a molecular formula of C_30_H_40_O_6_ on the basis the HRESIMS *m/z* 497.2903 [M + H]^+^ (calcd. for C_30_H_41_O_6_, 497.2898). Analyses of its NMR spectra showed that compound **31** was a dimeric sesquiterpenoid analogue bearing a rearranged cadinene-type and a normal cadinene-type sesquiterpenoid scaffolds. Its structure was closely related to that of **30**, and the difference was that a pair of exocyclic double bond at C-11' (*δ*_C_ 141.3) and C-13' [*δ*_C_ 121.0, *δ*_H_ 6.13 (1H, d, *J* = 1.2 Hz, H-13'a), 5.53 (1H, d, *J* = 1.2 Hz, H-13'b)] in compound **31** replaced a doublet methyl and a methine signals in **30**. The inference was supported by the HMBC correlations from H_2_-13' to C-7'/C-12'. The large coupling constants of H-1/H-6 and H-6/H-7 (*J*_H-1/H-6_ = *J*_H-6/H-7_ = 10.8 Hz), along with the ROESY correlations of H-1 with H-7/H_3_-14, of H-6 with H-10, and of H-1' with H-7'/H_3_-14' deduced that compound **31** exhibited the same relative configuration with **30** at the common shared stereocenters except for C-11'. The absolute configuration of compound **31** was established as 1*S*,6*S*,7*R*,10*R*,1'*S*,4'*S*,5'*R*,6'*S*,7'*S*,10'*R* based on the relative configuration and comparison of the calculated and experimental ECD curves.

Artemeriopodin G6 (**32**) was assigned the molecular formula of C_30_H_42_O_7_ based on its HRESIMS *m/z* 515.2998 [M + H]^+^ (calcd. for C_30_H_43_O_7_, 515.3003). The comparison of the ^1^H and ^13^C NMR data (Tables S8 and S10) demonstrated that compound **32** contained the same moiety B as **31**. The remained 15 carbon signals, including two methyls [*δ*_C_ 19.1 (C-14), 23.4 (C-15); *δ*_H_ 0.90 (3H, d, *J* = 6.0 Hz, H-14), 1.33 (3H, s, H-15)], five methylenes (*δ*_C_ 15.1, 24.7, 27.5, 34.5, and 124.4), four methines (*δ*_C_ 30.9, 46.4, 48.3, and 60.4), and four quaternary carbons (*δ*_C_ 60.6, 69.3, 143.2, and 168.7), revealed another cadinane-type sesquiterpenoid scaffold with a 4,5-epoxy ring and 6-OH (moiety A). The moiety A was fully constructed by the detailed 2D NMR analyses: the ^1^H-^1^H COSY correlations of H_2_-3/H_2_-2/H-1/H-10 and H-7/H_2_-8/H_2_-9/H-10/H_3_-14, together with the HMBC correlations from H_3_-15 to C-3/C-4/C-5, from H-5 to C-1/C-3/C-4/C-6/C-7/C-15, from H_3_-14 to C-1/C-9/C-10, from H-7 to C-1/C-6/C-11/C-12/C-13, as well as from H_2_-13 to C-7/C-11/C-12. By analyses of ROESY spectra, the relative configuration of moiety B in compound **32** was proved the same as those of **27**, **28**, and **31**. In addition, the ROESY correlations of H-1 with H-7/H_3_-14 and of H-5 with H_3_-15 disclosed the relative stereochemistry of moiety A in compound **32**. The absolute configuration of **32** was elucidated by comparing the experimental and calculated ECD data. The calculated ECD spectrum of **32** matched well with the experimental one proposed the 1*S*,4*R*,5*S*,6*S*,7*S*,10*R*,1'*S*,4'*S*,5'*R*,6'*S*,7'*S*,10'*R*-configuration.

Artemeriopodin G7 (**33**) showed a molecular formula of C_30_H_40_O_6_ as deduced from its HRESIMS *m/z* 497.2904 [M + H]^+^ (calcd. for C_30_H_41_O_6_, 497.2898). Just the same as those of compounds **27**, **28**, **31**, and **32**, the common moiety B existed in compound **33** by comparison of their 1D and 2D NMR data. The remained 15 carbons were described as a doublet methyl [*δ*_C_ 21.4 (C-14); *δ*_H_ 1.04 (3H, d, *J* = 6.0 Hz, H-14)], an *α*,*β*-unsaturated keto [*δ*_C_ 153.7 (C-5), 131.2 (C-15), 205.2 (C-4); *δ*_H_ 6.66 (1H, dd, *J* = 11.4, 4.2 Hz, H-5), 5.91 (1H, dt, *J* = 11.4, 1.8 Hz, H-15)], an *α*,*β*-unsaturated ester [*δ*_C_ 126.2 (C-13), 146.8 (C-11), 168.5 (C-12); *δ*_H_ 6.30 (1H, d, *J* = 0.6 Hz, H-13a), 5.57 (1H, s, H-13b)], as well as four methylenes (*δ*_C_ 26.2, 35.1, 36.7, and 41.5) and four methines (*δ*_C_ 37.6, 46.1, 47.1, and 47.6). The spin-coupling systems of H_2_-3/H_2_-2/H-1/H-6/H-5/H-15 and H-6/H-7/H_2_-8/H_2_-9/H-10/H_3_-14(H-1) in ^1^H-^1^H COSY spectrum along with the HMBC correlations from H_2_-2 to C-4, from H-15 to C-3/C-4/C-6, from H-5 to C-1/C-4/C-6/C-7, from H_3_-14 to C-1/C-9/C-10, and from H_2_-13 to C-7/C-11/C-12 established a rearranged 5(4→15)-*abeo*-cadinane with an unprecedented 7/6 bicyclic scaffold (moiety A). The moiety B of compound **33** had the same relative configuration as **27**, **28**, **31**, and **32** by comparing their ROESY spectra and NMR data. The large coupling constant of H-6 with H-7 (*J*_H-6/H-7_ = 11.4 Hz) indicated the *trans*-axial orientations of H-6 and H-7. The key ROESY correlations of H-1 with H_3_-14/H-7 and of H-6 with H-10 supported the relative configuration of moiety A. The absolute configuration of compound **33** was determined as 1*S*,6*R*,7*R*,10*R*,1'*S*,4'*S*,5'*R*,6'*S*,7'*S*,10'*R* by the ECD calculation in which the calculated curve showed high accordance with the experimental one.

Artemeriopodin G8 (**34**) had a molecular formula of C_30_H_42_O_8_ as established by the HRESIMS (*m/z* 529.2805 [M ‒ H]^‒^, calcd. for C_30_H_41_O_8_, 529.2807). The partial ^1^H and ^13^C NMR spectra of compound **34** displayed the similar resonances with those of **27**, **28**, and **31**‒**33**, which was implied that the moiety B also existed in **34**. The remained 15 carbons corresponded to two methyl [*δ*_C_ 20.3 (C-14), 29.9 (C-15); *δ*_H_ 0.92 (3H, d, *J* = 6.0 Hz, H-14), 2.14 (3H, s, H-15)], an *α*,*β*-unsaturated ester [*δ*_C_ 125.5 (C-13), 143.7 (C-11), 166.6 (C-12); *δ*_H_ 6.30 (1H, s, H-13a), 5.67 (1H, s, H-13b)], a saturated keto (*δ*_C_ 209.6, C-4), a carboxy group (*δ*_C_ 177.2, C-5), as well as four methylenes and four methines. The spin-coupling system of H_2_-3/H_2_-2/H-1/H-6/H-7/H_2_-8/H_2_-9/H-10/H_3_-14 in ^1^H-^1^H COSY spectrum as well as the HMBC correlations from H-15 to C-3/C-4, from H_3_-14 to C-1/C-9/C-10, from H-6 to C-2/C-5/C-10, from H-7 to C-1/C-6/C-11/C-12/C-13, and from H_2_-13 to C-7/C-11/C-12 determined a *seco*-cadinane type sesquiterpenoid scaffold (moiety A), which was similar with the reported compound (1*S*,6*R*,7*R*,10*R*)-6-carboxy-10-methyl-*α*-methylene-1-(1-oxobutyl)-cyclohexaneacetic acid^5^. The difference was the chemical shift changes of C-12 (*δ*_C_ 169.7 for the known compound; *δ*_C_ 166.6 for **34**), which verified the moieties A and B were linked by an ester bond between C-12 and C-4'. The moiety B of compound **34** had the same relative configuration as those of **27**, **28**, and **31**‒**33** by comparing their ROESY spectra and NMR data. For moiety A, the large coupling constants of H-6 with H-1 and H-7 (*J*_H-1/H-6_ = *J*_H-6/H-7_ = 11.4 Hz), along with the ROESY correlations of H-1 with H-7/H_3_-14 and of H-6 with H-10 confirmed the *α*-orientations of H-1, H-7, and H_3_-14, and the *β*-orientations of H-6 and H-10. By comparing the experimental and theoretically calculated ECD spectra, the absolute stereochemistry of **34** was demonstrated as 1*S*,6*R*,7*R*,10*R*,1'*S*,4'*S*,5'*R*,6'*S*,7'*S*,10'*R*.

Artemeriopodin H (**35**) was deduced to have a molecular formula of C_30_H_42_O_5_ based on the negative-ion mode HRESIMS data (*m/z* 483.3108 [M ‒ H]^‒^, calcd. for C_30_H_41_O_5_, 483.3105). The ^1^H and ^13^C NMR spectra of the moiety A in compound **35** were almost identical to those of **34**, indicating that compound **35** had the moiety A, (1*S*,6*R*,7*R*,10*R*)-6-carboxy-10-methyl-*α*-methylene-1- (1-oxobutyl)-cyclohexaneacetic acid.^6^ This inference was corroborated by the ^1^H-^1^H COSY correlations of H_2_-3/H_2_-2/H-1/H-6/H-7/H_2_-8/H_2_-9/H-10/H_3_-14 and the HMBC correlations from H-15 to C-3/C-4, from H_3_-14 to C-1/C-9/C-10, from H-6 to C-2/C-5/C-10, from H-7 to C-1/C-6/C-11/C-12/C-13, and from H_2_-13 to C-7/C-11/C-12. The remained 15 carbon resonances were attributed to two methyls [*δ*_C_ 21.3 (C-12'), 20.2 (C-14'); *δ*_H_ 1.75 (3H, s, H-12'), 0.88 (3H, s, H-14')], two pairs of exocyclic double bonds [*δ*_C_ 148.6 (C-4') and 110.7 (C-15'), 150.8 (C-11') and 109.8 (C-13'); *δ*_H_ 4.90 (1H, overlaped, H-15'a) and 4.74 (1H, s, H-15'b), 4.76 (1H, s, H-13'a) and 4.73 (1H, s, H-13'b)], an oxygenated quaternary carbon at *δ*_C_ 89.0 (C-5'), six methylenes, and a quaternary carbon. An eudesmane-type sesquiterpenoid moiety, 5*α*-hydroxy-eudesma-4(15),11-diene,^6^ was deduced by the interpretation of 2D NMR spectra: the ^1^H-^1^H COSY correlations of H_2_-1'/H_2_-2'/H_2_-3' and H_2_-6'/H-7'/H_2_-8'/H_2_-9', as well as the HMBC correlations from H_3_-14' to C-1'/C-9'/C-10', from H_3_-15' to C-3'/C-4'/C-5', from H-6' to C-4'/C-5'/C-10', from H_3_-12' to C-7'/C-11'/C-13', and from H_2_-13' to C-7'/C-11'/C-12'. The moieties A and B were assembled through an ester bond between C-5 and C-5', which was proved by the upfielded chemical shifts at C-5 (*δ*_C_ 174.2 *vs* 178.4) and the downfielded chemical shifts at C-5' (*δ*_C_ 89.0 *vs* 75.8).

The moiety A of compound **35** shared the same relative configuration with that of **34** by analyzing the coupling constants of H-6 with H-1 and H-7 (*J*_H-1/H-6_ = *J*_H-6/H-7_ = 10.2 Hz) and the ROESY correlations of H-1 with H-7/H_3_-14 and of H-6 with H-10. The relative configuration of moiety B in compound **35** kept consistent with 5*α*-hydroxy-eudesma-4(15), 11-diene, which was supported by the ROESY correlations of H_3_-14' with H-9'b/H_3_-12' and of H-7' with H-9'a, along with the comparison of their 1D NMR data. Finally, comparing the experimental and theoretically calculated ECD spectra identified the 1*S*,6*R*,7*R*,10*R*,5'*R*,7'*R*, 10'*R* absolute stereochemistry of compound **35**.

Artemeriopodin I (**36**) was determined to have a molecular formula of C_25_H_36_O_4_ based on the HRESIMS data at *m/z* 423.2502 [M + Na]^+^ (calcd. for C_25_H_36_O_4_Na, 423.2506). The analyses of 1D and 2D NMR data indicated that the artemanin B unit (moiety A) existed in compound **36**^59^. The remained 10 carbon signals were identified as two methyls at *δ*_C_ 26.2 (C-8') and 21.1 (C-9'), three methylenes (including an oxygenated one at *δ*_C_ 67.5), three methines (including an olefinic one at *δ*_C_ 121.9), and two quaternary carbons (including an olefinic one at *δ*_C_ 142.9). A carane-type monoterpene scaffold (moiety B), 3-caren-10-ol moiety,^7^ was constructed by the ^1^H-^1^H COSY correlations of H-1'/H-6'/H_2_-5'/H-4' and the HMBC correlations from H-6' to C-2'/C-3'/C-10', from H_3_-8' to C-1'/C-6'/C-7'/C-9', from H_3_-9' to C-1'/C-6'/C-7'/C-8', and from H_2_-10' to C-2'/C-3'/C-4'. The moieties A and B were linked by an ester bond between C-12 and C-10', which was demonstrated by the HMBC correlations from H_2_-10' to C-12. For moiety A, the ROESY correlations of H-1 with H-5/H-7/H_3_-14 and of H-6 with H-3/H-10/5-OH suggested that H-1, H-5, H-7, and H_3_-14 were the homolateral orientations, but opposite to H-3, H-6, H-10, and 5-OH. Additionally, the same orientations of H-1', H-6', and H_3_-8' in the moiety B were determined by the correlations of H_3_-8' with H-1'/H-6' in the ROESY spectrum. Subsequently, the absolute stereochemistry of compound **36** was concluded as 1*S*,3*R*,5*R*,6*R*,7*R*,10*R*,1'*S*,6'*R* by the relative stereochemistry deduced above and the comparison of its experimental and calculated ECD spectra.

Although many sesquiterpenoid dimers had been isolated from natural sources, the examples involving the cadinane-type sesquiterpenoids were rarely reported. In this study, 36 unusual cadinane-involved sesquiterpenoid dimers polymerized by two cadinanes or one cadinane with another type of sesquiterpenoid were isolated from *A. eriopoda* for the first time*.* Besides cadinane, the other monomers composing of these dimers included monocyclofarnesane (**1**‒**3**), bisabolane (**4** and **5**, 12-*nor*-cadinane (**6**), 4,5-*seco*-12-*nor*-cadinane (**7**‒**9**), 4,5-*seco*-cadinane (**10** and **34**), 12-*nor*-5,6-*seco*-cadinane (**7**‒**9**), a rare rearranged 5(4→3)-*abeo*-cadinane (**12**‒**18**, **22**‒**24** and **27**‒**31**), 5(4→3)-*abeo*-4,15-*dinor*-cadinane (**19**, **20**, **25**, and **26**), and 5(4→15)-*abeo*-cadinane with an unprecedented 7/6 bicyclic scaffold (**33**); compounds **11**, **21**, and **32** were cadinane-cadinane dimers; compound **35** was cadinane-eudesmane dimers, and compound **36** was a sesquiterpenoid-monoterpenoid conjugate formed by a carane and a 5(4→3)-*abeo*-cadinane. It should be noted that the monomers composing of the dimers were quite unusual, indicating the high structural diversities of sesquiterpenoid dimers in this plant.

Apart from the unusual units forming the sesquiterpenoid dimers, the constructed patterns of the units in these dimers were quite unique. For instance, a new spiro-cyclohexane ring was fused between C-11/C-11' and C-13/C-12' of the two units in compounds **1**‒**3**. An intriguing spiro-lactone ring system was formed between C-11/C-15' and C-12‒*O*‒C-4' of the two units in **4** and **5**. Although a simple C-C signal bond connected the two moieties in compounds **6**‒**10** and **24**‒**26**, their unusual joint positions were very different (**6**‒**9**: C-13/C-13'; **10**: C-5/C-13'; **24**‒**26**: C-13/C-15'). An unprecedented 5,7-dioxabicyclo[2.2.2]octan-6-one ring system was formed *via* hetero-Diels–Alder [4 + 2] cycloaddition and esterification between C-11/C-12/C-13 and C-4'/C-5'/C-15' of the two monomers in **12** and **13**. Eight cadinane-type sesquiterpenoid dimers (**14**‒**21**) were an intricate 6,8-dioxabicyclo[3.2.l]octan-7-one ring system which was formed by hetero-Diels–Alder [4 + 2] cycloaddition and esterification. However, arteannoide A was only one example of cadinane-type sesquiterpenoid dimer from *A. annua*.^43^ The new six-membered rings, tetrahydropyran, dihydropyran or lactone rings were respectively formed in the sesquiterpenoid dimers **11** (between C-4/C-15 and C-5'/C-15'), **22** (between C-11/C-13 and C-5'/C-15'), and **23** (between C-12/C-13 and C-4'/C-15'). In addition, the linkages of the cadinane sesquiterpenoid moieties in **27**‒**35** were through an ester bond at C-12/C-4' except **35** (at C-12/C-5'), and this kind of linkage model was also seldom reported previously. Summarily, 36 sesquiterpenoid dimers were classified as nine types of sesquiterpenoid dimers involving Diels-Alder reaction, radical addition, and esterification, which suggested chemical diversities of sesquiterpenoid dimers in *A. eriopoda*. To our knowledge, our study reported the most sum of sesquiterpenoid dimers with novel structural types in the field of natural products, which indicated that the sesquiterpenoid dimers found in *A. eriopoda* possessed highly structural diversity.

***Cytotoxicity of the isolates***

The isolates except for **4** and **5** (limited amount) were evaluated for cytotoxicity against HepG2, Huh7, and SK-Hep-1 cell lines. As shown in Table S11, compounds **13**, **16**, **31**, and **33** exhibited obvious cytotoxicity against HepG2 cells with IC_50_ values of 14.3, 12.2, 17.2, and 16.0 μmol/L, which were equivalent to that of sorafenib (IC_50_, 11.0 μmol/L). Compounds **12**, **18**, **27**, **28**, **30**, and **32** showed moderate cytotoxicity with IC_50_ values ranging from 20.0 to 45.3 μmol/L. Meanwhile, compounds **1**, **3**, **6**, **19**, **21**‒**24**, **29**, and **34**‒**36** displayed cytotoxicity with IC_50_ values in the range of 52.1**–**94.2 μmol/L, while other compounds were less active with IC_50_ values higher than 100 μmol/L.

Furthermore, compounds **31** and **33** demonstrated cytotoxicity against Huh7 cells with IC_50_ values of 10.3 and 18.3 μmol/L, comparable to sorafenib (IC_50_, 12.3 μmol/L). Compounds **13**, **28**, and **30** exhibited moderate cytotoxicity with IC_50_ values ranging from 26.4 to 39.5 μmol/L, and compounds **1**, **12**, **16**, **27**, **32**, and **34** displayed cytotoxicity with IC_50_ values of 53.1**–**97.5 μmol/L. But other compounds showed weak cytotoxicity with IC_50_ values higher than 100 μmol/L.

For SK-Hep-1 cells, compounds **31** and **33** displayed significant activity with IC_50_ values of 22.3 and 19.0 μmol/L, which were comparable to sorafenib (IC_50_, 18.1 μmol/L). Compounds **24**, **28**, **30**, and **34** showed moderate cytotoxicity with IC_50_ values ranging from 25.4 to 42.6 μmol/L, and compounds **6**, **12**, **13**, **16**, **18**, **27**, **32**, **35**, and **36** possessed activity with IC_50_ values ranging from 50.3 to 88.0 μmol/L. Other compounds exhibited weak cytotoxicity.

Interestingly, artemeriopodins G5 (**31**) and G7 (**33**) displayed the most inhibitory activity against three human hepatoma cell lines with IC_50_ values of 17.2, 10.3, 22.3 and 16.0, 18.3, 19.0 μmol/L equal to those of sorafenib (IC_50_: 11.0, 12.3, and 18.1 μmol/L). Although **31** exhibited a little better inhibitory potency against Huh7 cells and both displayed comparably inhibition in HepG2 and SK-Hep-1 cells, artemeriopodin G7 (**33**) featured an unprecedented 7/6 bicyclic scaffold and was chosen for further investigation. Subsequently, the cytotoxicity of artemeriopodin G7 (**33**) and sorafenib on THLE-2 cells (normal hepatocyte cells) were further evaluated by CCK8 assay (Figs. S12a‒S12f), suggesting that **33** showed a better safety on THLE-2 cells (IC_50_, 32.0 μM) than sorafenib (IC_50_, 16.7 μM). In order to compare the difference between CCK8 and MTT methods, the cytotoxicity of compound **33** and sorafenib on HepG2 cells were tested by CCK8 assay to yield IC_50_ values of 15.3 and 13.1 μM, which was consistent with the MTT results of 16.0 and 11.0 μM, respectively. From a comprehensive consideration, artemeriopodin G7 (**33**) was chosen for further investigation.

***Potential targets prediction for biomarkers of HCC***

Swiss Target Prediction was used to predict the targets of artemeriopodin G7 (**33**) (Table S12), and HCC samples were acquired in the datasets of GSE14520, GSE45267, GSE62232, GSE87630 and GSE112790 (Fig. S7B, Table S13). The results suggested that 75 targets were unanimously upregulated and 205 targets were downregulated in the five GEO datasets. GEO targets and the predicted targets of artemeriopodin G7 (**33**) were intersected using Venny software to obtain 6 potential targets, including HSD11B1, CYP2C9, CYP3A4, PDGFRA, CETP and CCNA2 (Figs. S7C and S7D). Among them, HSD11B1, CYP2C9, CYP3A4, PDGFRA, and CETP were lowly expressed but CCNA2 was highly expressed in HCC tissues, which led to a poor clinical outcome in patients (Figs. S8a and S8b). The expression status of these targets at different pathological stages of HCC suggested that their expression levels were closely related to a poor prognosis in HCC patients (Fig. S8c). The survival analysis results indicated that these targets resulted in poor clinical outcome and had no difference in adjacent (Figs. S8d and S8e). Moreover, their ROC curve analyses exhibited the prognostic values (Fig. S8f). The above results suggested that the potential targets of artemeriopodin G7 (**33**) were HSD11B1, CYP2C9, CYP3A4, PDGFRA, CETP and CCNA2, which showed important function in HCC progression.

***GO and KEGG pathway enrichment analysis***

To explore the molecular mechanism promoting HCC progression, 107 genes’ biological functions of artemeriopodin G7 (**33**) were obtained from GO enrichment analysis. The top 10 markedly enriched gene biological function catalogs were selected for the generation of scatterplots (Fig. S9A). These results showed that artemeriopodin G7 (**33**) was involved in HCC progress through a variety of gene biological functions, contributing to the understanding of mechanisms in HCC. KEGG pathway enrichment analysis of these targets indicated that many signaling pathways were closely associated with artemeriopodin G7 (**33**), such as Ras signaling pathway, PI3K/AKT signaling pathway, MAPK signaling pathway, and cell cycle (Fig. S9B, Table S14). Then, a molecular docking approach was used to predict the interaction between artemeriopodin G7 (**33**) and target proteins. The result manifested that the high binding affinity with PDGFRA (supplementary Fig. 10f), and binding energy was ‒6.9 kcal/mol, which were higher than the empirical threshold (−5.0 kcal/mol). PDGFRA was related to cell proliferation and metastasis. Taken together, these results indicated that artemeriopodin G7 (**33**) might play an antihepatoma role through the expression of PDGFRA protein regulated by the AKT/STAT signaling pathway.

## Materials and Methods

## *General experimental instruments and procedures*

A Shimadzu LC/MS-IT-TOF mass sectrometer (Shimadzu, Kyoto, Japan) was used to gain the high-resolution mass spectra. UV spectra were conducted on a Shimadzu UV2401PC spectrophotometer (Shimadzu, Kyoto, Japan), and IR (KBr) spectra were obtained on a Bio-Rad FTS-135 spectrometer (Hercules, California, USA). 1D and 2D NMR spectra were conducted on Advance III-400 or III-600 spectrometers (Bruker, Bremerhaven, Germany) with TMS as the internal standard. Optical rotations were determined on a JASCO P-1020 digital polarimeter (Horiba, Tokyo, Japan). X-ray crystallographic analyses using Cu K*α* radiation were performed on a Bruker D8 QUEST instrument (Bruker, Karlsruher, Germany). Electronic circular dichroism (ECD) spectra were measured on an Applied Photophysics Circular dichromatograph (Applied Photophysics, Britain). Thin-layer chromatography (TLC) analyses were performed on silica gel GF_254_ plates (Yantai Jiangyou Silicon Development Company, Yantai, China), and spots were monitored under UV light or by heating after sprayed with 10% H_2_SO_4_ in EtOH (*v/v*). Silica gel (200~300 mesh, Linyi Haixiang, Linyi, China) and Sephadex LH-20 (GE Healthcare Bio-Sciences AB, Uppsala, Sweden) were used for column chromatography. Medium pressure liquid chromatography (MPLC) separation was conducted on a Dr-Flash II apparatus using an MCI gel CHP 20P column (75~150 *μ*m, Mitsubishi Chemical Corporation, Tokyo, Japan). High performance liquid chromatography (HPLC) was performed on a Shimadzu LC-CBM-20 system (Shimadzu, Kyoto, Japan) with Agilent XDB-C_18_ (5 *μ*m, 9.4 × 250 mm) columns.

Cell cycle and apoptosis assays were performed on a flow cytometer (FACSCalibur, Becton Dickinson Co., Ltd., New Jersey, USA). Western blot assay was conducted on vertical electrophoresis apparatus (DYCZ-24DN, Liuyi Biotechnology Co., Ltd., Beijing, China) and a Semi-Dry Blotter (YRDIMES, wealtec Co., Ltd., Nevada, USA.), exposured by MultiSpectral imaging system (BioSpectrum, Analytik Jena Co., Ltd., Jena, Germany).

## Plant materials

*Artemisia eriopoda* Bge. (Asteraceae) were collected from Xuchang, Henan province of China, in August 2018, and authenticated by Prof. Dr. Li-Gong Lei (CAS Key Laboratory for Plant Diversity and Biogeography of East Asia, Kunming Institute of Botany, Chinese Academy of Sciences). A voucher specimen (No. 20180805e) was deposited at the Laboratory of Anti-virus and Natural Medicinal Chemistry, Kunming Institute of Botany, Chinese Academy of Sciences.

## *Extraction and isolation*

The dried whole plant of *A. eriopoda* (15.7 kg) was powdered and extracted with ethanol for two times at room temperature. The combined extraction was concentrated under reduced pressure to yield a crude residue, which was suspended in water and extracted with EtOAc. The EtOAc extract (550 g, Fr. A) was separated to six fractions [Frs. A-1 (20 g), A-2 (130 g), A-3 (80 g), A-4 (80 g), A-5 (123 g), and A-6 (85 g)] by silica gel column chromatography (CC) using a mixed solvent of acetone-petroleum ether (0:100, 5:95, 10:90, 20:80, 40:60, 100:0, *v/v*).

Fr. A-4 (80 g) was subjected to MCI gel CHP 20P CC eluting with MeOH-H_2_O system (50:50, 70:30, 90:10, and 100:0) to yield four fractions (Frs. A-4a‒A-4d). Fr. A-4c (14 g) was divided into five fractions (Frs. A-4c-1‒A-4c-5) by silica gel CC (acetone-petroleum ether, 5:95, 10:90, 20:80). Fr. A-4c-4 (2.4 g) was further fractionated by Sephadex LH-20 CC (MeOH-CHCl_3_, 50:50), Rp-C_18_ CC (MeOH-H_2_O, 50:50. 60:40, 70:30, 80:20), and semi-preparative HPLC purification (Agilent XDB-C_18_ column, MeCN-H_2_O, 55:45; MeOH-H_2_O, 80:20) to yield compounds **1** (67 mg, *t_R_* = 15.6 min), **2** (25 mg, *t_R_* = 17.3 min), and **3** (17 mg, *t_R_* =22.6 min). Fr. A-4c-5 (10 g) was separated by Rp-C_18_ CC (MeOH-H_2_O, 50:50, 60:40, 70:30) to obtain six fractions (Frs. A-4c-5a‒A-4c-5f). Fr. A-4c-5a (460 mg) was purified by semi-preparative HPLC (Agilent XDB-C_18_ column, MeCN-H_2_O, 60:40; MeOH-H_2_O, 82:18) to afford compounds **11** (4 mg, *t_R_* = 32.0 min), **12** (43 mg, *t_R_* = 11.5 min), **13** (2 mg, *t_R_* = 9.5 min), **28** (2 mg, *t_R_* = 21.4 min), **29** (2 mg, *t_R_* = 30.5 min), and **36** (5 mg *t_R_* = 20.5 min). Compounds **14** (70 mg, *t_R_* = 26 min), **16** (6 mg, *t_R_* = 22.5 min), **17** (5 mg, *t_R_* = 24.5 min), and **18** (70 mg, *t_R_* = 22.5 min) were obtained from Fr. A-4c-5c (1 g) by Sephadex LH-20 CC (MeOH-CHCl_3_, 50:50), and semi-preparative HPLC purification on an Agilent XDB-C_18_ column (MeCN-H_2_O, 50:50; MeOH-H_2_O, 75:25). Fr. A-4c-5e (2.3 g) was separated by Sephadex LH-20 CC (MeOH-CHCl_3_, 50:50), and semi-preparative HPLC (Agilent XDB-C_18_ column, CH_3_CN-H_2_O, 48:52; MeOH-H_2_O, 78:22) to provide compounds **6** (4 mg, *t_R_* = 25.0 min), and **15** (19 mg, *t_R_* = 38.0 min).

Fr. A-5 (123 g) was divided into four fractions (Frs. A-5a‒A-5d) by MCI gel CHP 20P CC and eluted with MeOH-H_2_O (50:50, 70:30, 90:10, and 100:0). Fr. A-5c (18 g) was further fractionated by Rp-C_18_ CC (MeOH-H_2_O, 50:50, 60:40, 70:30, and 100:0) to yield four fractions (Frs. A-5c-1‒A-5c-4). Compounds **4** (1 mg, *t_R_* = 14.5 min), **5** (1.3 mg, *t_R_* = 16.0 min), **7** (15 mg, *t_R_* = 21. min), **8** (2 mg, *t_R_* = 15.6 min), **9** (5 mg, *t_R_* =18.0 min), and **10** (10 mg, *t_R_* = 24.2 min) were obtained from Fr. A-5c-1 (2 g) by Sephadex LH-20 CC (MeOH-CHCl_3_, 50:50), and semi-preparative HPLC [Agilent XDB-C_18_ column, MeCN-H_2_O (50:50), MeOH-H_2_O (73:27)]. Fr. A-5c-2 (4 g) was fractionated by silica gel CC (acetone-CHCl_3_, 5:95, 10:90) to yield five fractions (Frs. A-5c-2a‒A-5c-2e). Fr. A-5c-2a (1.5 g) was fractionated by Sephadex LH-20 CC (MeOH-CHCl_3_, 50:50) and semi-preparative HPLC purification (Agilent XDB-C_18_ column, MeCN-H_2_O, 47:53; MeOH-H_2_O, 75:25) to yield compounds **22** (5 mg, *t_R_* = 27.3 min), **23** (3 mg, *t_R_* = 21.8 min), and **24** (7 mg, *t_R_* = 19.6 min). Fr. A-5c-2b (1.7 g) was fractionated by Sephadex LH-20 CC (MeOH-CHCl_3_, 50:50) and semi-preparative HPLC purification (Agilent XDB-C_18_ column, MeCN-H_2_O, 55:45; MeOH-H_2_O, 80:20) to yield compounds **27** (21 mg, *t_R_* =18.2 min), **30** (5 mg, *t_R_* =21.0 min), **31** (21 mg, *t_R_* = 29.0 min), and **33** (6 mg, *t_R_* = 22.8 min). Fr. A-5c-2c (1 g) was purified by Sephadex LH-20 CC (MeOH-CHCl_3_, 50:50), and semi-preparative HPLC (Agilent XDB-C_18_ column, MeCN-H_2_O, 48:52; MeOH-H_2_O, 76:24) to afford compounds **19** (1 mg, *t_R_* = 25.0 min), **20** (15 mg, *t_R_* = 25.0 min), and **25** (7 mg, *t_R_* = 21.3 min). Fr. A-5c-3 (2.4 g) was separated by silica gel CC (acetone-CHCl_3_, 10:90, 20:80), Sephadex LH-20 CC (MeOH-CHCl_3_, 50:50), and semi-preparative HPLC (Agilent XDB-C_18_ column, CH_3_CN-H_2_O, 50:50; MeOH-H_2_O, 75:25) to get compounds **21** (18 mg, *t_R_* = 41.0 min), **26** (11 mg, *t_R_* = 20.0 min), **32** (22 mg, *t_R_* = 28.8 min), **34** (22 mg, *t_R_* = 32.1 min), and **35** (26 mg, *t_R_* = 37.0 min).

Artemeriopodin A1 (**1**)

Colorless oils; [*α*]Combin –64.0 (*c* 0.13, MeOH); ECD (*c* 0.21, MeOH) *λ*_max_ (*∆ε*): 208 (+2.33), 235 (–0.48), 265 (+0.26) nm; IR (KBr) *ν*_max_: 3447, 1730, 1658, 1645, 1454, 1443, 1382, 1233, 1193, 1124, 1075, 1024 cm^–1^; ^1^H (600 MHz) and ^13^C NMR (DEPT, 150 MHz) see Tables S1 and S2; HRESIMS *m/z*: 453.3356 [M + H]^+^ (calcd. for C_30_H_45_O_3_, 453.3363).

Artemeriopodin A2 (**2**)

Colorless oils; [*α*]Combin –30.2 (*c* 0.06, MeOH); ECD (*c* 0.18, MeOH) *λ*_max_ (*∆ε*): 196 (+7.81), 240 (–0.65), 271 (+0.27) nm; IR (KBr) *ν*_max_: 3445, 1730, 1714, 1645, 1455, 1397, 1384, 1194, 1103, 1068 cm^–1^; ^1^H and ^13^C NMR data see Tables S1 and S2; HRESIMS *m/z*: 453.3359 [M + H]^+^ (calcd. for C_30_H_45_O_3_, 453.3363).

Artemeriopodin A3 (**3**)

Colorless oils; [*α*]Combin –15.4 (*c* 0.09, MeOH); ECD (*c* 0.27, MeOH) *λ*_max_ (*∆ε*): 203 (+2.75), 235 (–0.91), 270 (+0.43) nm; IR (KBr) *ν*_max_: 3440, 1714, 1640, 1455, 1444, 1382, 1204, 1052 cm^–1^; ^1^H and ^13^C NMR data see Tables S1 and S2; HRESIMS *m/z*: 453.3357 [M + H]^+^ (calcd. for C_30_H_45_O_3_, 453.3363).

Artemeriopodin B1 (**4**)

Colorless oils; [*α*]Combin +15.6 (*c* 0.10, MeOH); UV (MeOH) *λ*_max_ (log *ε*): 247 (2.82) nm; ECD (*c* 0.20, MeOH) *λ*_max_ (*∆ε*): 243 (–0.45), 283 (+0.58) nm; IR (KBr) *ν*_max_: 3443, 1758, 1707, 1626, 1456, 1384, 1168, 1155 cm^–1^; ^1^H and ^13^C NMR data see Tables S1 and S3; HRESIMS *m/z*: 517.3157 [M + H]^+^ (calcd. for C_30_H_45_O_7_, 517.3160).

Artemeriopodin B2 (**5**)

Colorless oils; [*α*]Combin +29.0 (*c* 0.13, MeOH); UV (MeOH) *λ*_max_ (log *ε*): 259 (3.10) nm; ECD (*c* 0.26, MeOH) *λ*_max_ (*∆ε*): 260 (–1.97), 296 (+0.56) nm; IR (KBr) *ν*_max_: 3444, 1760, 1706, 1627, 1457, 1445, 1384, 1295, 1248, 1159, 1026, 1001 cm^–1^; ^1^H and ^13^C NMR data see Tables S1 and S3; HRESIMS *m/z*: 517.3158 [M + H]^+^ (calcd. for C_30_H_45_O_7_, 517.3160).

Artemeriopodin C1 (**6**)

White powders; [*α*]Combin –26.0 (*c* 0.10, MeOH); UV (MeOH) *λ*_max_ (log *ε*): 219 (3.14) nm; ECD (*c* 0.01, MeOH) *λ*_max_ (*∆ε*): 212 (+4.21), 240 (–2.11), 262 (+0.12), 290 (–1.14) nm; IR (KBr) *ν*_max_: 1750, 1707, 1682, 1632, 1449, 1384, 1257, 1143, 1070, 1011 cm^–1^; ^1^H and ^13^C NMR data see Tables S1 and S3; HRESIMS *m/z*: 453.3001 [M + H]^+^ (calcd. for C_29_H_41_O_4_, 453.2999).

Artemeriopodin C2 (**7**)

White powders; [*α*]Combin –40.6 (*c* 0.10, MeOH); UV (MeOH) *λ*_max_ (log *ε*): 221 (3.11) nm; ECD (*c* 0.20, MeOH) *λ*_max_ (*∆ε*): 213 (+2.57), 241 (–2.88), 286 (+0.874) nm; IR (KBr) *ν*_max_: 3429, 1749, 1713, 1618, 1572, 1450, 1378, 1242, 1144, 1091 cm^–1^; ^1^H and ^13^C NMR data see Tables S1 and S3; HRESIMS *m/z*: 501.2853 [M + H]^+^ (calcd. for C_29_H_41_O_7_, 501.2847).

Artemeriopodin C3 (**8**)

White powders; [*α*]Combin –2.5 (*c* 0.20, MeOH); UV (MeOH) *λ*_max_ (log *ε*): 215 (2.94) nm; ECD (*c* 0.22, MeOH) *λ*_max_ (*∆ε*): 207 (+0.38), 227 (+1.83), 247 (+0.38) nm; IR (KBr) *ν*_max_: 3440, 1746, 1711, 1632, 1454, 1384, 1165, 1104 cm^–1^; ^1^H and ^13^C NMR data see Tables S1 and S3; HRESIMS *m/z*: 482.2895 [M + H]^+^ (calcd. for C_29_H_40_NO_5_, 482.2901).

Artemeriopodin C4 (**9**)

White powders; [*α*]Combin +8.0 (*c* 0.05, MeOH); UV (MeOH) *λ*_max_ (log *ε*): 215 (3.08) nm; ECD (*c* 0.18, MeOH) *λ*_max_ (*∆ε*): 221 (+3.69), 245 (–0.01), 264 (+0.71) nm; IR (KBr) *ν*_max_: 3443, 1745, 1706, 1632, 1454, 1384, 1260, 1165, 1049 cm^–1^; ^1^H and ^13^C NMR data see Tables S1 and S3; HRESIMS *m/z*: 482.2882 [M + H]^+^ (calcd. for C_29_H_40_NO_5_, 482.2901).

Artemeriopodin D (**10**)

White powders; [*α*]Combin –13.0 (*c* 0.10, MeOH); UV (MeOH) *λ*_max_ (log *ε*): 220 (2.98) nm; ECD (*c* 0.20, MeOH) *λ*_max_ (*∆ε*): 216 (+1.32), 269 (–1.58) nm; IR (KBr) *ν*_max_: 3428, 1751, 1712, 1620, 1562, 1451, 1382, 1274, 1169, 1096 cm^–1^; ^1^H and ^13^C NMR data see Tables S1 and S4; HRESIMS *m/z*: 513.2844 [M + H]^+^ (calcd. for C_30_H_41_O_7_, 513.2847).

Artemeriopodin E (**11**)

White powders; [*α*]Combin –13.0 (*c* 0.40, MeOH); UV (MeOH) *λ*_max_ (log *ε*): 214 (2.84) nm; ECD (*c* 0.19, MeOH) *λ*_max_ (*∆ε*): 236 (–0.14), 268 (+0.23), 309 (–0.58) nm; IR (KBr) *ν*_max_: 3445, 1762, 1711, 1633, 1454, 1384, 1253, 1133, 1076 cm^–1^; ^1^H and ^13^C NMR data see Tables S1 and S4; HRESIMS *m/z*: 521.2865 [M + Na]^+^ (calcd. for C_30_H_42_O_6_Na, 521.2874).

Artemeriopodin F1 (**12**)

White monoclinic crystals (MeOH–H_2_O); [*α*]Combin –64.7 (*c* 0.12, MeOH); UV (MeOH) *λ*_max_ (log *ε*): 218 (2.80) nm; ECD (*c* 0.23, MeOH) *λ*_max_ (*∆ε*): 217 (–6.00), 256 (+0.93) nm; IR (KBr) *ν*_max_: 3432, 1767, 1708, 1666, 1632, 1455, 1382, 1280, 1264, 1166, 1098 cm^–1^; ^1^H and ^13^C NMR data see Tables S1 and S4; HRESIMS *m/z*: 527.2650 [M ‒ H]^‒^ (calcd. for C_30_H_39_O_8_, 527.2650).

Artemeriopodin F2 (**13**)

White powders; [*α*]Combin –96.5 (*c* 0.08, MeOH); UV (MeOH) *λ*_max_ (log *ε*): 239 (2.96) nm; ECD (*c* 0.16, MeOH) *λ*_max_ (*∆ε*): 215 (–5.48), 243 (–4.48), 270 (+0.60), 316 (–0.86) nm; IR (KBr) *ν*_max_: 3441, 1768, 1713, 1648, 1456, 1378, 1264, 1164, 1082 cm^–1^; ^1^H and ^13^C NMR data see Tables S1 and S4; HRESIMS *m/z*: 511.2699 [M + H]^+^ (calcd. for C_30_H_39_O_7_, 511.2690).

Artemeriopodin F3 (**14**)

White orthorhombic crystals (MeOH–H_2_O); [*α*]Combin –34.9 (*c* 0.12, MeOH); UV (MeOH) *λ*_max_ (log *ε*): 218 (2.80) nm; ECD (*c* 0.24, MeOH) *λ*_max_ (*∆ε*): 215 (+0.83), 234 (–1.77), 54 (+0.01), 278 (–0.54) nm; IR (KBr) *ν*_max_: 3433, 1790, 1712, 1628, 1456, 1382, 1362, 1250, 1223, 1180, 1166, 1105 cm^–1^; ^1^H and ^13^C NMR data see Tables S4 and S5; HRESIMS *m/z*: 515.2999 [M + H]^+^ (calcd. for C_30_H_43_O_7_, 515.3003).

Artemeriopodin F4 (**15**)

White powders; [*α*]Combin –18.2 (*c* 0.19, MeOH); UV (MeOH) *λ*_max_ (log *ε*): 220 (3.59) nm; ECD (*c* 0.23, MeOH) *λ*_max_ (*∆ε*): 205 (–8.55), 230 (–7.84) nm; IR (KBr) *ν*_max_: 3522, 3435, 1789, 1711, 1628, 1455, 1384, 1250, 1223, 1166, 1105 cm^–1^; ^1^H and ^13^C NMR data see Tables S4 and S5; HRESIMS *m/z*: 515.2999 [M + H]^+^ (calcd. for C_30_H_43_O_7_, 515.3003).

Artemeriopodin F5 (**16**)

White orthorhombic crystals (MeOH–H_2_O); [*α*]Combin –58.0 (*c* 0.11, MeOH); UV (MeOH) *λ*_max_ (log *ε*): 241 (2.81) nm; ECD (*c* 0.22, MeOH) *λ*_max_ (*∆ε*): 237 (–7.47), 270 (+0.43), 314 (–0.69) nm; IR (KBr) *ν*_max_: 3443, 1782, 1713, 1659, 1634, 1455, 1376, 1245, 1111, 1063 cm^–1^; ^1^H and ^13^C NMR data see Tables 5 and S6; HRESIMS *m/z*: 483.3145 [M ‒ H]^‒^ (calcd. for C_30_H_43_O_5_, 482.3116).

Artemeriopodin F6 (**17**)

White monoclinic crystals (MeOH–H_2_O); [*α*]Combin –100.3 (*c* 0.20, MeOH); UV (MeOH) *λ*_max_ (log *ε*): 238 (2.98) nm; ECD (*c* 0.22, MeOH) *λ*_max_ (*∆ε*): 230 (–6.10), 271 (+0.66), 314 (–0.44) nm; IR (KBr) *ν*_max_: 3441, 1801, 1771, 1711, 1660, 1636, 1455, 1383, 1261, 1161, 1056 cm^–1^; ^1^H and ^13^C NMR data see Tables S5 and S6; HRESIMS *m/z*: 533.2505 [M + Na]^+^ (calcd. for C_30_H_38_O_7_Na, 533.2510).

Artemeriopodin F7 (**18**)

White powders; [*α*]Combin –62.2 (*c* 0.17, MeOH); UV (MeOH) *λ*_max_ (log *ε*): 240 (2.83) nm; ECD (*c* 0.21, MeOH) *λ*_max_ (*∆ε*): 235 (–2.89), 2.88 (–0.76) nm; IR (KBr) *ν*_max_: 3501, 1803, 1765, 1710, 1667, 1660, 1455, 1384, 1268, 1163, 1027 cm^–1^; ^1^H and ^13^C NMR data see Tables S5 and S6; HRESIMS *m/z*: 499.2701 [M + H]^+^ (calcd. for C_29_H_39_O_7_, 499.2690).

Artemeriopodin F8 (**19**)

White monoclinic crystals (MeOH–H_2_O); [*α*]Combin +2.6 (*c* 0.21, MeOH); UV (MeOH) *λ*_max_ (log *ε*): 224 (2.84) nm; ECD (*c* 0.21, MeOH) *λ*_max_ (*∆ε*): 218 (+2.61), 235 (–4.30), 308 (+0.76) nm; IR (KBr) *ν*_max_: 3411, 1788, 1713, 1611, 1456, 1444, 1384, 1240, 1167, 1089 cm^–1^; ^1^H and ^13^C NMR data see Tables S5 and S6; HRESIMS *m/z*: 469.2591 [M + H]^+^ (calcd. for C_28_H_37_O_6_, 469.2585).

Artemeriopodin F9 (**20**)

White powders; [*α*]Combin +1.3 (*c* 0.14, MeOH); UV (MeOH) *λ*_max_ (log *ε*): 224 (3.09) nm; ECD (*c* 0.17, MeOH) *λ*_max_ (*∆ε*): 217 (+7.77), 235 (–9.08), 310 (+1.77) nm; IR (KBr) *ν*_max_: 3432, 1789, 1714, 1681, 1614, 1459, 1379, 1241, 1193, 1166, 1109 cm^–1^; ^1^H and ^13^C NMR data see Tables S5 and S6; HRESIMS *m/z*: 469.2593 [M + H]^+^ (calcd. for C_28_H_37_O_6_, 469.2585).

Artemeriopodin F10 (**21**)

White powders; [*α*]Combin ‒18.5 (*c* 0.14, MeOH); UV (MeOH) *λ*_max_ (log *ε*): 241 (3.08) nm; ECD (*c* 0.23, MeOH) *λ*_max_ (*∆ε*): 215 (+1.31), 236 (–5.31), 313 (–1.31) nm; IR (KBr) *ν*_max_: 3436, 1791, 1711, 1650, 1632, 1619, 1597, 1455, 1384, 1246, 1183, 1141, 1069 cm^–1^; ^1^H and ^13^C NMR data see Tables S5 and S6; HRESIMS *m/z*: 513.2856 [M + H]^+^ (calcd. for C_30_H_41_O_7_, 513.2547).

Artemeriopodin F11 (**22**)

White powders; [*α*]Combin ‒40.2 (*c* 0.19, MeOH); UV (MeOH) *λ*_max_ (log *ε*): 216 (3.06) nm; ECD (*c* 0.19, MeOH) *λ*_max_ (*∆ε*): 207 (–1.50), 226 (+0.49), 256 (+0.33) nm; IR (KBr) *ν*_max_: 3571, 1756, 1662, 1591, 1455, 1442, 1383, 1278, 1169 cm^–1^; ^1^H and ^13^C NMR data see Tables S5 and S7; HRESIMS *m/z*: 495.2742 [M + H]^+^ (calcd. for C_30_H_39_O_6_, 495.2741).

Artemeriopodin F12 (**23**)

White powders; [*α*]Combin ‒35.9 (*c* 0.08, MeOH); UV (MeOH) *λ*_max_ (log *ε*): 239 (2.82) nm; ECD (*c* 0.20, MeOH) *λ*_max_ (*∆ε*): 208 (–1.27), 232 (+0.72), 250 (–0.56), 291 (+0.76), 323 (–0.66) nm; IR (KBr) *ν*_max_: 3428, 1728, 1656, 1631, 1445, 1378, 1236, 1218, 1160, 1044 cm^–1^; ^1^H and ^13^C NMR data see Tables S5 and S7; HRESIMS *m/z*: 497.2891 [M + H]^+^ (calcd. for C_30_H_41_O_6_, 497.2898).

Artemeriopodin F13 (**24**)

White powders; [*α*]Combin ‒14.5 (*c* 0.05, MeOH); ECD (*c* 0.23, MeOH) *λ*_max_ (*∆ε*): 202 (–3.96), 226 (+2.38), 299 (+0.60) nm; IR (KBr) *ν*_max_: 3445, 1706, 1633, 1455, 1384, 1205, 1048 cm^–1^; ^1^H and ^13^C NMR data see Tables S5 and S7; HRESIMS *m/z*: 583.3232 [M + Na]^+^ (calcd. for C_32_H_48_O_8_Na, 583.3241).

Artemeriopodin F14 (**25**)

White powders; [*α*]Combin +13.7 (*c* 0.07, MeOH); UV (MeOH) *λ*_max_ (log *ε*): 219 (2.89) nm; ECD (*c* 0.20, MeOH) *λ*_max_ (*∆ε*): 203 (–5.54), 226 (+0.75), 242 (–0.12), 296 (+1.66) nm; IR (KBr) *ν*_max_: 3430, 1747, 1711, 1627, 1553, 1444, 1384, 1277, 1136, 1096, 1082 cm^–1^; ^1^H and ^13^C NMR data see Tables 5S and S7; HRESIMS *m/z*: 469.2593 [M + H]^+^ (calcd. for C_28_H_37_O_6_, 469.2585).

Artemeriopodin F15 (**26**)

White powders; [*α*]Combin +26.7 (*c* 0.09, MeOH); UV (MeOH) *λ*_max_ (log *ε*): 218 (3.09) nm; ECD (*c* 0.19, MeOH) *λ*_max_ (*∆ε*): 203 (–10.14), 226 (+1.94), 296 (+2.90) nm; IR (KBr) *ν*_max_: 1748, 1709, 1626, 1553, 1454, 1444, 1383, 1283, 1185, 1080 cm^–1^; ^1^H and ^13^C NMR data see Tables S5 and S7; HRESIMS *m/z*: 491.2398 [M + Na]^+^ (calcd. for C_28_H_36_O_6_Na, 491.2404).

Artemeriopodin G1 (**27**)

White triclinic crystals (MeOH–H_2_O); [*α*]Combin ‒39 (*c* 0.14, MeOH); UV (MeOH) *λ*_max_ (log *ε*): 219 (2.80) nm; ECD (*c* 0.22, MeOH) *λ*_max_ (*∆ε*):214 (–5.92), 287 (–0.27) nm; IR (KBr) *ν*_max_: 3331, 1750, 1705, 1630, 1590, 1454, 1384, 1279, 1180, 1160, 1056 cm^–1^; ^1^H and ^13^C NMR data see Tables S8 and S9; HRESIMS *m/z*: 515.2997 [M + H]^+^ (calcd. for C_30_H_43_O_7_, 515.3003).

Artemeriopodin G2 (**28**)

White powder; [*α*]Combin ‒18 (*c* 0.09, MeOH); UV (MeOH) *λ*_max_ (log *ε*): 219 (2.84) nm; ECD (*c* 0.18, MeOH) *λ*_max_ (*∆ε*): 215 (–4.80), 276 (+0.60) nm; IR (KBr) *ν*_max_: 3408, 1752, 1707, 1630, 1453, 1382, 1278, 1160, 1057 cm^–1^; ^1^H and ^13^C NMR data see Tables S8 and S9; HRESIMS *m/z*: 515.3005 [M + H]^+^ (calcd. for C_30_H_43_O_7_, 515.3003).

Artemeriopodin G3 (**29**)

White monoclinic crystals (MeOH–H_2_O); [*α*]Combin ‒65 (*c* 0.04, MeOH); UV (MeOH) *λ*_max_ (log *ε*): 220 (2.94) nm; ECD (*c* 0.20, MeOH) *λ*_max_ (*∆ε*): 199 (–8.11), 233 (–0.48), 280 (–1.45) nm; IR (KBr) *ν*_max_: 3442, 1756, 1703, 1631, 1455, 1384, 1249, 1160, 1056 cm^–1^; ^1^H and ^13^C NMR data see Tables S8 and S9; HRESIMS *m/z*: 539.2974 [M + Na]^+^ (calcd. for C_30_H_44_O_7_Na, 539.2979).

Artemeriopodin G4 (**30**)

White powder; [*α*]Combin ‒36 (*c* 0.10, MeOH); UV (MeOH) *λ*_max_ (log *ε*): 240 (2.80) nm; ECD (*c* 0.20, MeOH) *λ*_max_ (*∆ε*): 221 (–2.14), 269 (+0.36) nm; IR (KBr) *ν*_max_: 3440, 1770, 1706, 1631, 1455, 1384, 1250, 1160, 1056 cm^–1^; ^1^H and ^13^C NMR data see Tables S8 and S9; HRESIMS *m/z*: 499.3047 [M + H]^+^ (calcd. for C_30_H_43_O_6_, 499.3054).

Artemeriopodin G5 (**31**)

White powder; [*α*]Combin ‒220 (*c* 0.10, MeOH); UV (MeOH) *λ*_max_ (log *ε*): 216 (3.47), 251 (3.12) nm; ECD (*c* 0.20, MeOH) *λ*_max_ (*∆ε*): 215 (–33.4), 260 (+3.25) nm; IR (KBr) *ν*_max_: 3441, 1764, 1707, 1650, 1631, 1455, 1384, 1276, 1160, 1056 cm^–1^; ^1^H and ^13^C NMR data see Tables S8 and S9; HRESIMS *m/z*: 497.2903 [M + H]^+^ (calcd. for C_30_H_41_O_6_, 497.2898).

Artemeriopodin G6 (**32**)

White powder; [*α*]Combin ‒37 (*c* 0.10, MeOH); UV (MeOH) *λ*_max_ (log *ε*): 220 (2.85) nm; ECD (*c* 0.21, MeOH) *λ*_max_ (*∆ε*): 214 (–9.84), 260 (+1.24) nm; IR (KBr) *ν*_max_: 3447, 1761, 1710, 1633, 1454, 1383, 1269, 1161, 1039 cm^–1^; ^1^H and ^13^C NMR data see Tables S8 and S10; HRESIMS *m/z*: 515.2998 [M + H]^+^ (calcd. for C_30_H_43_O_7_, 515.3003).

Artemeriopodin G7 (**33**)

White powder; [*α*]Combin ‒53 (*c* 0.30, MeOH); UV (MeOH) *λ*_max_ (log *ε*): 220 (2.98) nm; ECD (*c* 0.21, MeOH) *λ*_max_ (*∆ε*): 216 (–7.47), 269 (+1.14) nm; IR (KBr) *ν*_max_: 3441, 1763, 1706, 1632, 1454, 1384, 1275, 1159, 1056 cm^–1^; ^1^H and ^13^C NMR data see Tables S8 and S10; HRESIMS *m/z*: 497.2904 [M + H]^+^ (calcd. for C_30_H_41_O_6_, 497.2898).

Artemeriopodin G8 (**34**)

White powder; [*α*]Combin ‒35 (*c* 0.11, MeOH); UV (MeOH) *λ*_max_ (log *ε*):220 (2.94) nm; ECD (*c* 0.23, MeOH) *λ*_max_ (*∆ε*): 214 (–7.38) nm; IR (KBr) *ν*_max_: 3429, 1710, 1624, 1453, 1278, 1158, 1106 cm^–1^; ^1^H and ^13^C NMR data see Tables S8 and S10; HRESIMS *m/z*: 529.2805 [M ‒ H]^‒^ (calcd. for C_30_H_41_O_8_, 529.2807).

Artemeriopodin H (**35**)

White powder; [*α*]Combin +19 (*c* 0.12, MeOH); UV (MeOH) *λ*_max_ (log *ε*): 220 (2.69) nm; ECD (*c* 0.23, MeOH) *λ*_max_ (*∆ε*): 205 (–0.84), 234 (+1.16) nm; IR (KBr) *ν*_max_: 3440, 1716, 1632, 1454, 1384, 1260, 1169, 1152, 1105 cm^–1^; ^1^H and ^13^C NMR data see Tables S8 and S10; HRESIMS *m/z*: 483.3108 [M ‒ H]^‒^ (calcd. for C_30_H_41_O_5_, 483.3105).

Artemeriopodin I (**36**)

White powder; [*α*]Combin ‒7 (*c* 0.12, MeOH); UV (MeOH) *λ*_max_ (log *ε*): 220 (2.35) nm; ECD (*c* 0.24, MeOH) *λ*_max_ (*∆ε*): 215 (–0.14), 242 (+0.44) nm; IR (KBr) *ν*_max_: 3440, 1716, 1632, 1454, 1384, 1260, 1169, 1152, 1105 cm^–1^; ^1^H and ^13^C NMR data see Tables S8 and S10; HRESIMS *m/z*: 423.2502 [M + Na]^+^ (calcd. for C_25_H_36_O_4_Na, 423.2506).

## X-ray crystallographic analyses

Crystals of compounds **12**, **14**, **16**, **17**, **19**, **27**, and **29** were obtained by using the solvent vapor diffusion method. The single-crystal X-ray diffraction data were recorded on a Bruker D8 QUEST instrument (Cu K*α* radiation). Crystals were kept at 100.(2) K during data collection. The crystallographic data of those compounds in standard CIF format were deposited at the Cambridge Crystallographic Data Centre. The data can be accessed free of charge at <http://www.ccdc.cam.ac.uk/>.

Crystallographic data for compound **12**: C_30_H_40_O_8_•CH_4_O, *M* = 560.66, *a* = 10.5181(5) Å, *b* = 9.4074(4) Å, *c* = 14.4284(6) Å, *α* = 90°, *β* = 98.2520(10)°, *γ* = 90°, *V* = 1412.88(11) Å^3^, *T* = 100.(2) K, space group *P*1211, *Z* = 2, *μ*(Cu Kα) = 0.786 mm^-1^, 22677 measured reflections, 5521 independent reflections (*R_int_* = 0.0501). The final *R_1_* values were 0.0387 (*I* > 2*σ*(*I*)). The final *wR*(*F*^2^) values were 0.1065 (*I* > 2*σ*(*I*)). The final *R_1_* values were 0.0388 (all data). The final *wR*(*F*^2^) values were 0.1068 (all data). The goodness of fit on *F*^2^ was 1.060. Flack parameter = 0.02(4). CCDC 2175134.

Crystal data for compound **14**: C_30_H_42_O_7_, *M* = 514.63, *a* = 6.5291(2) Å, *b* = 14.8526(5) Å, *c* = 27.9016(9) Å, *α* = 90°, *β* = 90°, *γ* = 90°, *V* = 2705.73(15) Å^3^, *T* = 100.(2) K, space group *P*212121, *Z* = 4, *μ*(Cu Kα) = 0.717 mm^-1^, 28964 measured reflections, 5317 independent reflections (*R_int_* = 0.0481). The final *R_1_* values were 0.0290 (*I* > 2*σ*(*I*)). The final *wR*(*F*^2^) values were 0.0716 (*I* > 2*σ*(*I*)). The final *R_1_* values were 0.0296 (all data). The final *wR*(*F*^2^) values were 0.0721 (all data). The goodness of fit on *F*^2^ was 1.046. Flack parameter = 0.02(4). CCDC 2175136.

Crystal data for compound **16**: C_30_H_44_O_5_•CH_4_O, M = 516.69, *a* = 9.7508(2) Å, *b* = 15.6539(4) Å, *c* = 18.5081(4) Å, *α* = 90°, *β* = 90°, *γ* = 90°, *V* = 2825.04(11) Å^3^, *T* = 100.(2) K, space group *P*212121, *Z* = 4, *μ*(Cu Kα) = 0.657 mm^-1^, 27658 measured reflections, 5547 independent reflections (*R_int_* = 0.0416). The final *R_1_* values were 0.0297 (*I* > 2*σ*(*I*)). The final *wR*(*F*^2^) values were 0.0751 (*I* > 2*σ*(*I*)). The final *R_1_* values were 0.0305 (all data). The final *wR*(*F*^2^) values were 0.0760 (all data). The goodness of fit on *F*^2^ was 1.052. Flack parameter = 0.04(4). CCDC 2175135.

Crystal data for compound **17**: C_30_H_38_O_7_, M = 510.60, *a* = 6.5775(2) Å, *b* = 13.4562(4) Å, *c* = 15.0547(4) Å, *α* = 90°, *β* = 93.5300(10)°, *γ* = 90°, *V* = 1329.94(7) Å^3^, *T* = 100.(2) K, space group *P*1211, *Z* = 2, *μ*(Cu Kα) = 0.729 mm^-1^, 30680 measured reflections, 5145 independent reflections (*R_int_* = 0.0413). The final *R_1_* values were 0.0271 (*I* > 2*σ*(*I*)). The final *wR*(*F*^2^) values were 0.0686 (*I* > 2*σ*(*I*)). The final *R_1_* values were 0.0272 (all data). The final *wR*(*F*^2^) values were 0.0687 (all data). The goodness of fit on *F*^2^ was 1.061. Flack parameter = 0.04(4). CCDC 2175138.

Crystal data for compound **19**: C_28_H_36_O_6_, M = 468.57, *a* = 6.3785(2) Å, *b* = 13.0497(4) Å, *c* = 15.0504(5) Å, *α* = 90°, *β* = 97.2540(10)°, *γ* = 90°, *V* = 1242.73(7) Å^3^, *T* = 100.(2) K, space group *P*1211, *Z* = 2, *μ*(Cu Kα) = 0.702 mm^-1^, 19324 measured reflections, 4701 independent reflections (*R_int_* = 0.0801). The final *R_1_* values were 0.0389 (*I* > 2*σ*(*I*)). The final *wR*(*F*^2^) values were 0.1003 (*I* > 2*σ*(*I*)). The final *R_1_* values were 0.0438 (all data). The final *wR*(*F*^2^) values were 0.1031 (all data). The goodness of fit on *F*^2^ was 1.042. Flack parameter = 0.19(10). CCDC 2175137.

Crystal data for compound **27**: C_30_H_42_O_7_, M = 514.63, *a* = 9.1256(3) Å, *b* = 9.2284(3) Å, *c* = 16.5768(6) Å, *α* = 89.6560(10)°, *β* = 84.1740(10)°, *γ* = 81.4640(10)°, *V* = 1373.36(8) Å^3^, *T* = 100.(2) K, space group *P*1, *Z* = 2, *μ*(Cu Kα) = 0.707 mm^-1^, 41783 measured reflections, 10366 independent reflections (*R_int_* = 0.0497). The final *R_1_* values were 0.0628 (*I* > 2*σ*(*I*)). The final *wR*(*F*^2^) values were 0.1697 (*I* > 2*σ*(*I*)). The final *R_1_* values were 0.0632 (all data). The final *wR*(*F*^2^) values were 0.1704 (all data). The goodness of fit on *F*^2^ was 1.029. Flack parameter = 0.20(7). CCDC 2175140.

Crystal data for compound **29**: C_30_H_44_O_7_, M = 516.65, *a* = 9.8566(8) Å, *b* = 8.8385(7) Å, *c* = 15.9881(11) Å, *α* = 90°, *β* = 94.190(4)°, *γ* = 90°, *V* = 1389.12(18) Å^3^, *T* = 100.(2) K, space group *P*1211, *Z* = 2, *μ*(Cu Kα) = 0.699 mm^-1^, 31887 measured reflections, 5211 independent reflections (*R_int_* = 0.1979). The final *R_1_* values were 0.0730 (*I* > 2*σ*(*I*)). The final *wR*(*F*^2^) values were 0.1825 (*I* > 2*σ*(*I*)). The final *R_1_* values were 0.1078 (all data). The final *wR*(*F*^2^) values were 0.2074 (all data). The goodness of fit on *F*^2^ was 1.038. Flack parameter = 0.4(2). CCDC 2175139.

## *ECD calculations*

The conformation search was performed by Spartan '14 software using molecular mechanics MMFF94x. The appropriate low-energy conformers were selected and optimized in the gas phase by semi-empirical method in Gaussian 09 program package, and were further optimized and analyzed for frequency using the density functional theory (DFT) at the B3LYP/6-31G(d,p) level, resulting in no imaginary frequencies. Solvent effects were taken into consideration by using the conductor polarizable continuum model (CPCM). All the conformers were used for calculating electronic circular dichroism (ECD) by the time-dependent density functional theory (TD-DFT) method at the B3LYP/6-31G(d,p) level with the CPCM model in MeOH.1 The overall calculated ECD curves were generated by Boltzmann weighting of the selected low-energy conformers using SpecDis 1.62 with σ = ~0.3 eV.

## *Cytotoxicity assays*

The cytotoxicity of the compounds **1**–**3** and **6**–**36** was tested by the MTT assay. Briefly, cells in a density of 3×10^4^ cells/well were seeded into 96-well plates and incubated at 37 °C with 5% CO_2_ for 24 h. The culture medium was replaced with fresh medium containing different concentrations of guaianolide dimers, and cells were incubated for additional 48 h. After removal of the medium, 100 μL of MTT reagent (1mg/mL) was added into each well, and the plates were kept in incubator for 4 h. After that, 100 μL of dimethyl sulfoxide (DMSO) was added into each well, and the plates were measured at 490 nm using microplate reader (BIO-RAD, USA). The inhibitory ratio was calculated as [(A_490_ control ‒ A_490_ treated)/A_490_ control] ×100%. The cytotoxicity of compounds was expressed as IC_50_ values calculated by GraphPad Prism 5 (GraphPad Software, California, USA).

## *Data Source and Processing*

The Swiss Target Prediction (http://swisstargetprediction.ch/) was applied to predict the possible targets, which includes a network of chemicals and associated drug targets. Download the gene expression profile data of HCC from Gene Expression Omnibus (GEO, https://portal.gdc.cancer.gov/) database. Liver Hepatocellular Carcinoma (LIHC) of The Cancer Genome Atlas (TCGA, https://portal.gdc.cancer.gov/)，Integrative Molecular Database of Hepatocellular Carcinoma (HCCDB, http://lifeome.net/database/hccdb/home.html) and The Human Protein Atlas (HPA, https://www.proteinatlas.org/) web was used for testing the gene expression level, survival analysis , the correlation between target gene expression and the pathological stages of cancer and Receiver Operating Characteristic (ROC). Tumor IMmune Estimation Resource 2.0 (TIMER2.0, http://timer.cistrome.org/) web server is a comprehensive resource for systematical analysis of immune infiltrates across diverse cancer types.

## *GO and KEGG Pathway Enrichment Analysis*

First, potential target gene names were transformed into entrezID by the R package “org.Hs.eg.db, version =3.10.0,” which helps to exclude errors caused by capitalization or abbreviations of the target name. Then, GO biological functions and the KEGG pathway enrichment analysis were visualized with the R packages “clusterProfiler” for which the p-value was <0.05 for further analysis.

## *Molecular docking studies*

Docking of potential target gene was explored using Sybyl-X 2.0, and crystal structure of HSD11B1(PDB: LXU9), CYP2C9 (PDB: LOG2), CYP3A4 (PDB: 4D7D), PDGFRA (PDB: 5GRN), CETP (PDB: 4EWS) was obtained from the RCSB Protein Data Bank. All polar hydrogen atoms were added and solvation parameters were assigned. Before the docking process, the ligand substructures were extracted and water molecules were removed. The surflex-dock total score was expressed in ‒log*K*d to represent binding affinities. The higher docking total score, the stronger interaction of proteins and bioactive compounds.

***THLE-2 cell*** ***proliferation assay***

CCK-8 (AC11L057, Shanghai Liji Biotechnology Co., LTD, Shanghai, China) assay wasused to evaluate cell proliferation of the compounds on ThLE-2 cell proliferation. ThLE-2 cells were cultured in MEGM (CC-3170, Lonza Co, Ltd). In brief, ThLE-2 cells in the logarithmic growth phase were seeded into 96-well plates (90 μL per well) at the appropriate density for 24 h. Then, cells were treated with different concentrations of compound **33** and sorafenib for 72 h. Three wells were set for each concentration and corresponding concentrations of solvent control and cell-free zero-adjustment wells were set. Next, 10 μL CCK-8 reagent was added into each well for another 1 to 2 h of incubation. Then, optical density (OD) values were measured at 450 nm using a microplate reader (SpectraMax 190, MolecμLar Device Co, Ltd). The inhibitory ratio was calculated as [(Acontrol ‒ Atreated)/A control] ×100%.

## *Cell migration and invasion assays*

Cell migration and invasion of HepG2 cells were evaluated by Transwell assays (Corning, USA). For cell migration assay, HepG2 cells (10×10^5^/mL) were seeded on the upper chambers. After adherence, the cells were maintained in serum-free DMEM with various concentrations of compound **33** for 48 h. Then, cells in the upper chambers were wiped, and the migrated cells were fixed in 70% ethanol and stained with crystal violet solution (0.1%) for 30 min. After that, images were taken by imaging system (Olympus IX73). For cell invasion assay, matrigel (BD Biosciences) was diluted to 1:50 in pre-cool DMEM medium and added to the upper chamber 6 h prior to seeding the cells. The subsequent procedures were the same as above.

## Flow cytometry assays

We assessed the effect of compound **33** on cell cycle and apoptosis by flow cytometry. HepG2 cells were seeded into 6-well plates at a density of 3×10^5^ cells per well, cultured overnight. Then, cells were treated with various concentrations (0.0, 10.0, 15.0, and 20.0 μM) of **33** for 12 h (cell cycle) or 48h (apoptosis). In cell cycle assay, cells were collected and fixed in 70% ethanol at -20 °C overnight. Thereafter, HepG2 cells were resuspended in PBS containing in staining buffer of 100 μg/mL PI and 200 μg/mL RNase A. In apoptosis assay, cells were harvested and suspended in binding buffer, and stained with fluorochrome Annexin V/PI for 15 min. Finally, the cells were analyzed by flow cytometry. Cell cycle and apoptosis assays were analyzed by using a BD AccuriC6 flow cytometer (BD Biosciences, San Jose, CA, USA).

## Western blot

The expression of apoptosis- and cell cycle-related proteins was determined by Western blot. Briefly, HepG2 cells were seeded into 6-well plates and treated with compound **33** for 48 h. The cells were collected and lysed in RIPA buffer to extract total protein, and the protein concentration was determined by BCA method. Samples were then fractionated using SDS-PAGE and transferred to PVDF membranes. Then, the membranes were incubated with specific primary antibodies at 4 °C overnight. The secondary antibody was incubated for 2 h at room temperature. Proteins were detected by ECL solution (Advansta, USA) and photographed by the multispectral imaging system (UVP, USA).

**CETSA assays**

CETSA was performed as described previously reported.[1](https://www.ncbi.nlm.nih.gov/pmc/articles/PMC8148064/#bib14)7 HepG2 cells were divided into 2 groups, one group hatched with 20 celastrol, and the control group used the same DMSO ,then collected the 2 h cells. PBS washed twiced and resuspended with 550 μL of PBS. Both groups are divided into 6 servings, each of which is 50 μL. Set the PCR meter to 6 temperature gradients (46, 49, 52, 55, 58, and 61 °C) to every sample with the corresponds temperature, heated 3 min, then placed at room temperature for 3 min, and then placed in an ice bath. Finally, the samples are repeatedly frozen and melted for three times. Transfer the processed sample to an imported eppendorf tube, centrifuge at 13000 ×*g* and 4 °C for 30 min, add 6 × loading buffer, heat samples for 10 min.

**Isothermal titration calorimetry (ITC)**

The interactions between PDGFRA (Sinobiolocal, China) and compound **33** was analyzed by Isothermal titration calorimetry. Protein sample was dissolved in PBS (10 mM), and compound **33** was dissolved in DMSO, then attenuated to 0.5 mM in PBS (0.5% DMSO). The titrations were performed by injecting 2 μL protein into solution of compound **33**. The data were analyzed with the MicroCal PEAQ-ITC (Malvern, USA) instrument using the independent ﬁt model.

**Surface Plasmon Resonance (SPR) assay**

The interactions between PDGFRA and the compound **33** was analyzed by Surface Plasmon Resonance (SPR) assay at 25 °C. The protein was dissolved in 10 mM sodium acetate buffer (pH 4.5) at a concentration of 1 mg/mL and covalently linked to a CM5 sensor chip with the Amine Coupling Kit (GE, Boston, USA). Compound **33** (0–100 μM) was dissolved in the running buffer (PBS with 0.005% surfactant P20 and 5% DMSO). Association and dissociation curves were obtained on a BIAcore S200 instrument (Biacore AB, Uppsala, Sweden). The data were analyzed using Biacore S200 (GE Healthcare, Sweden) Evaluation Software 2.0.1.

**References**

1. Khrimian, A. et al. Discovery of the aggregation pheromone of the brown marmorated stink bug (*Halyomorpha halys*) through the creation of stereoisomeric libraries of 1‑bisabolen-3-ols. *J. Nat. Prod.* **77**, 1708−1717 (2014).

2. Khrimian, A. et al. Determination of the stereochemistry of the aggregation pheromone of Harlequin bug, *Murgantia histrionica*. *J. Chem. Eco.l* **40**, 1260−1268 (2014).

3. Qin, D. P. et al. Dimeric cadinane sesquiterpenoid derivatives from *Artemisia annua*. *Org. Lett.* **20**, 453−456 (2018).

4. Takenaka, Y. et al. Two new sesquiterpenes from *Artemisia annua* L. *J. Nat. Med.* **74**, 811–818 (2020).

5. Li, H. B.et al. Two new sesquiterpenoids from *Artemisia annua*. *Magn. Reson. Chem.* **53**, 244–247 (2015).

6. Sy, L. K. & Brown, G. D. Three sesquiterpenes from *Artemisia annua*. *Phytochemistry* **48**, 1207–1211 (1998).

7. Frackowiak, B. et al. Stereochemistry of terpene derivatives. Part 5: Synthesis of chiral lactones fused to a carane system-insect feeding deterrents. *Tetrahedron*: *Asymmetry* **17**, 124–129 (2006).

**supplementary Fig. 1** The structures of compounds **1**–**36**.

# supplementary Fig. 2 The key ^1^H-^1^H COSY and HMBC correlations of compounds 1–36.


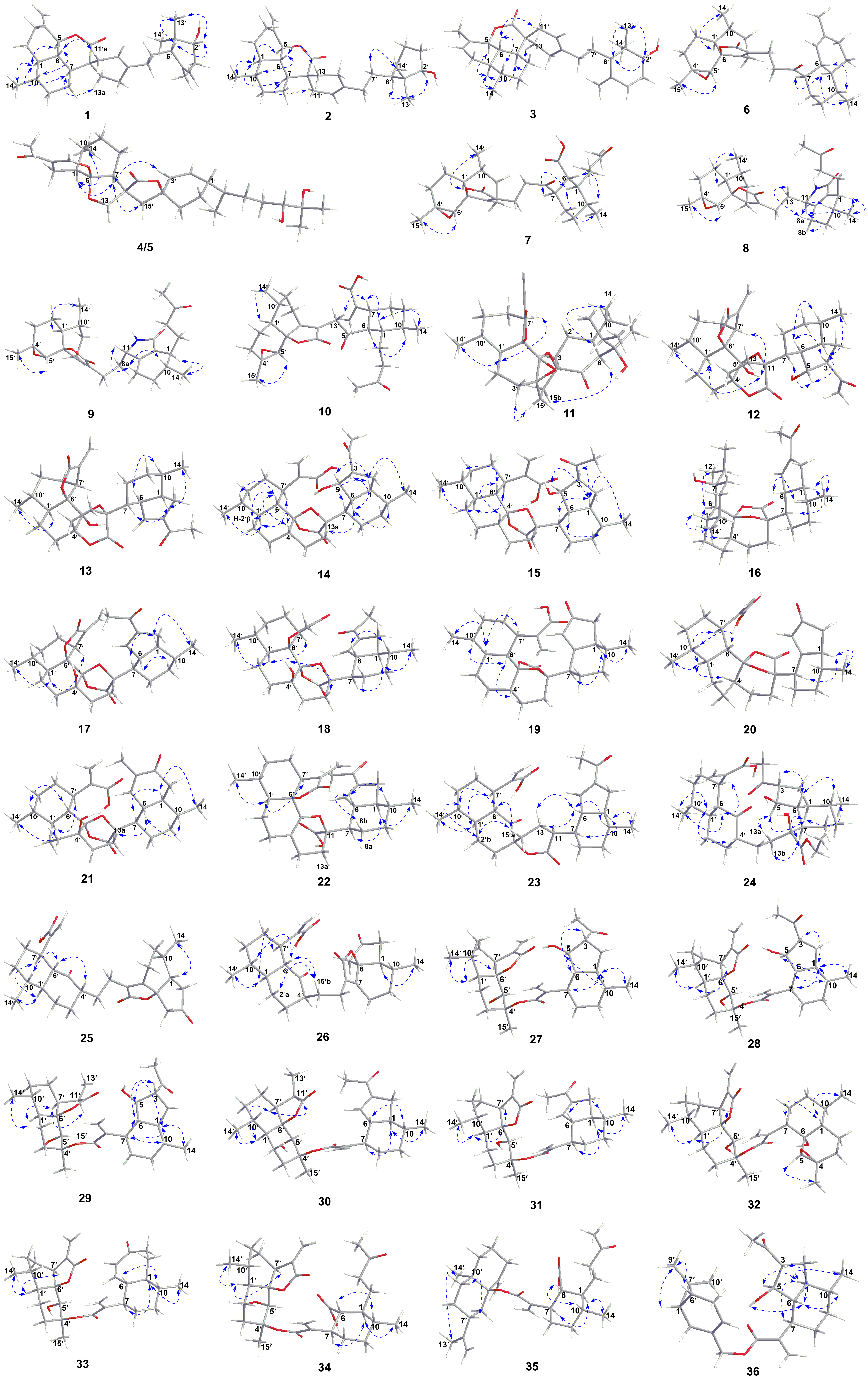


# supplementary Fig. 3 The key ROESY correlations of compounds 1–36.


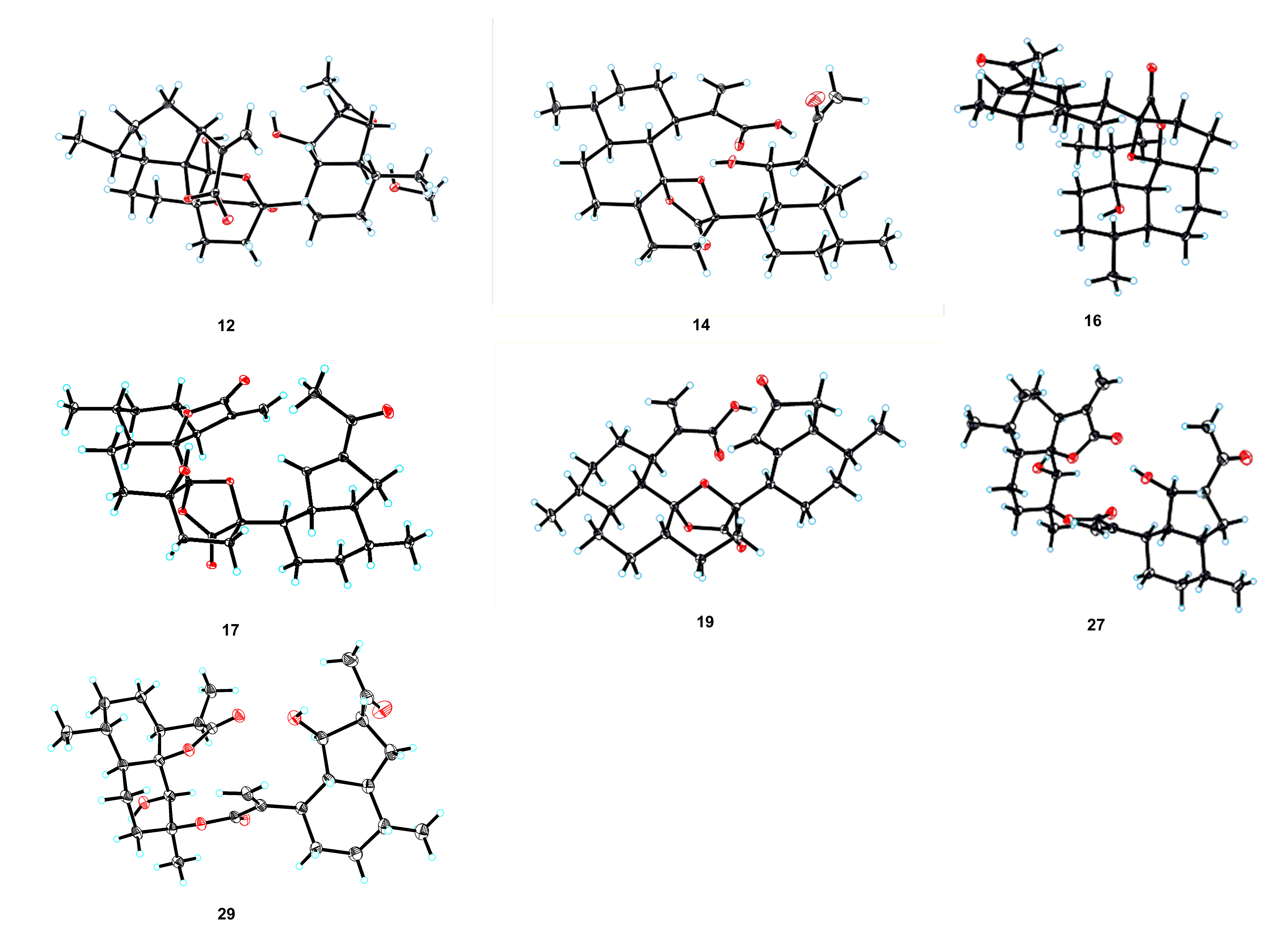


**supplementary Fig. 4** X-ray crystallographic structures of compounds **12**, **14**, **16**, **17**, **19**, **27**, and **29**.


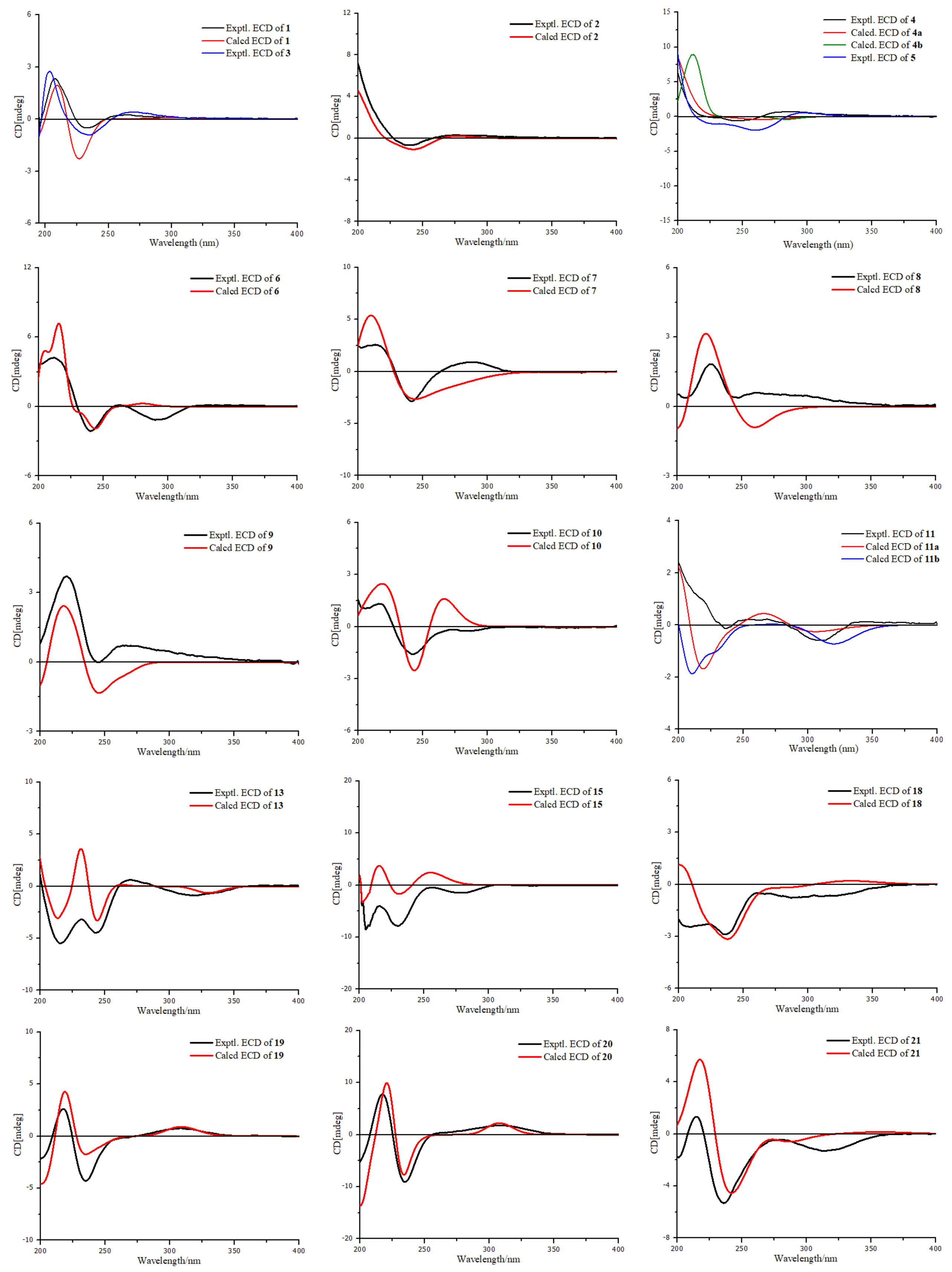


# supplementary Fig. 5 Experimental and calculated ECD spectra of compounds 1–21.


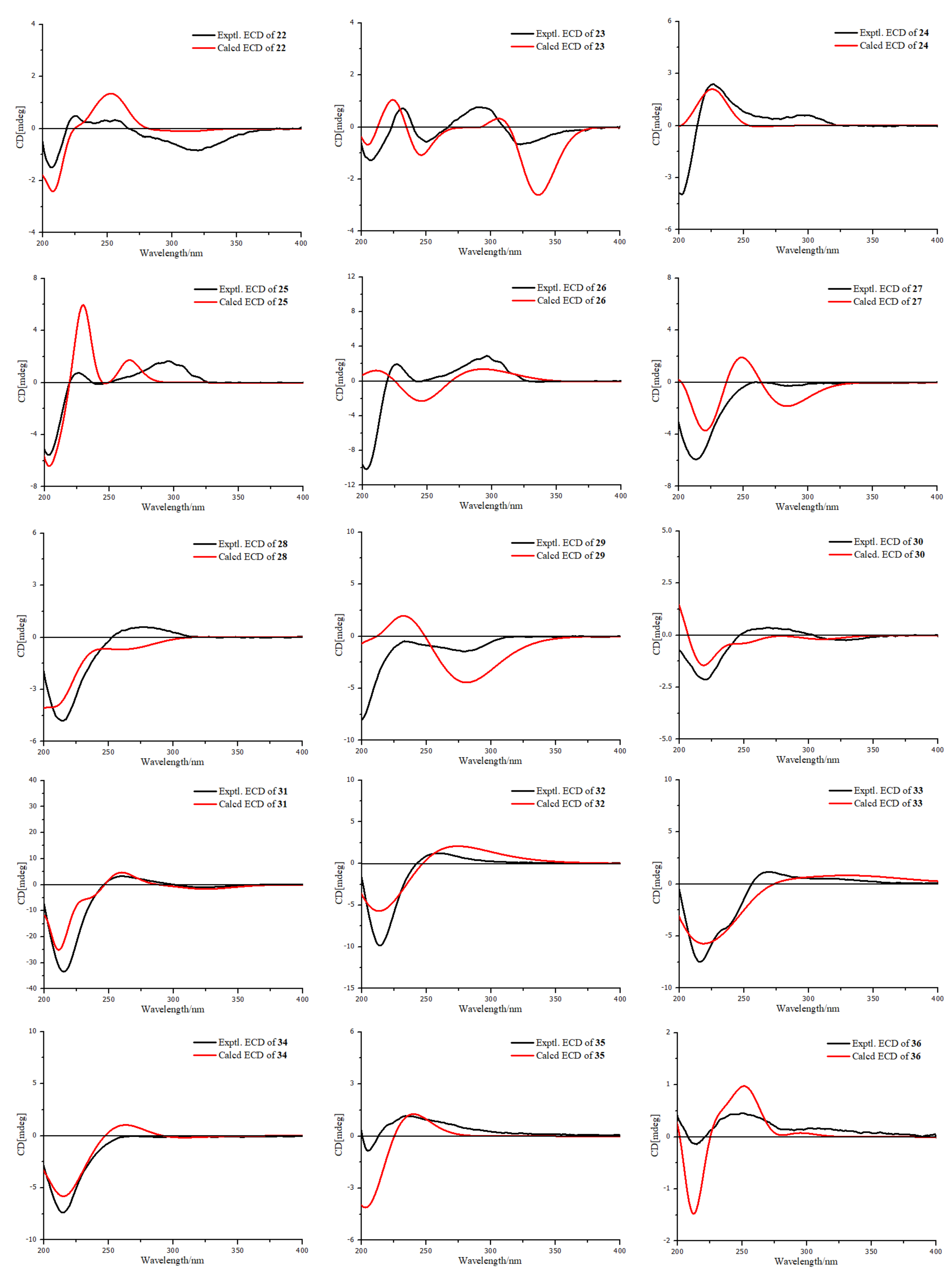


# supplementary Fig. 6 Experimental and calculated ECD spectra of compounds 22–36

**
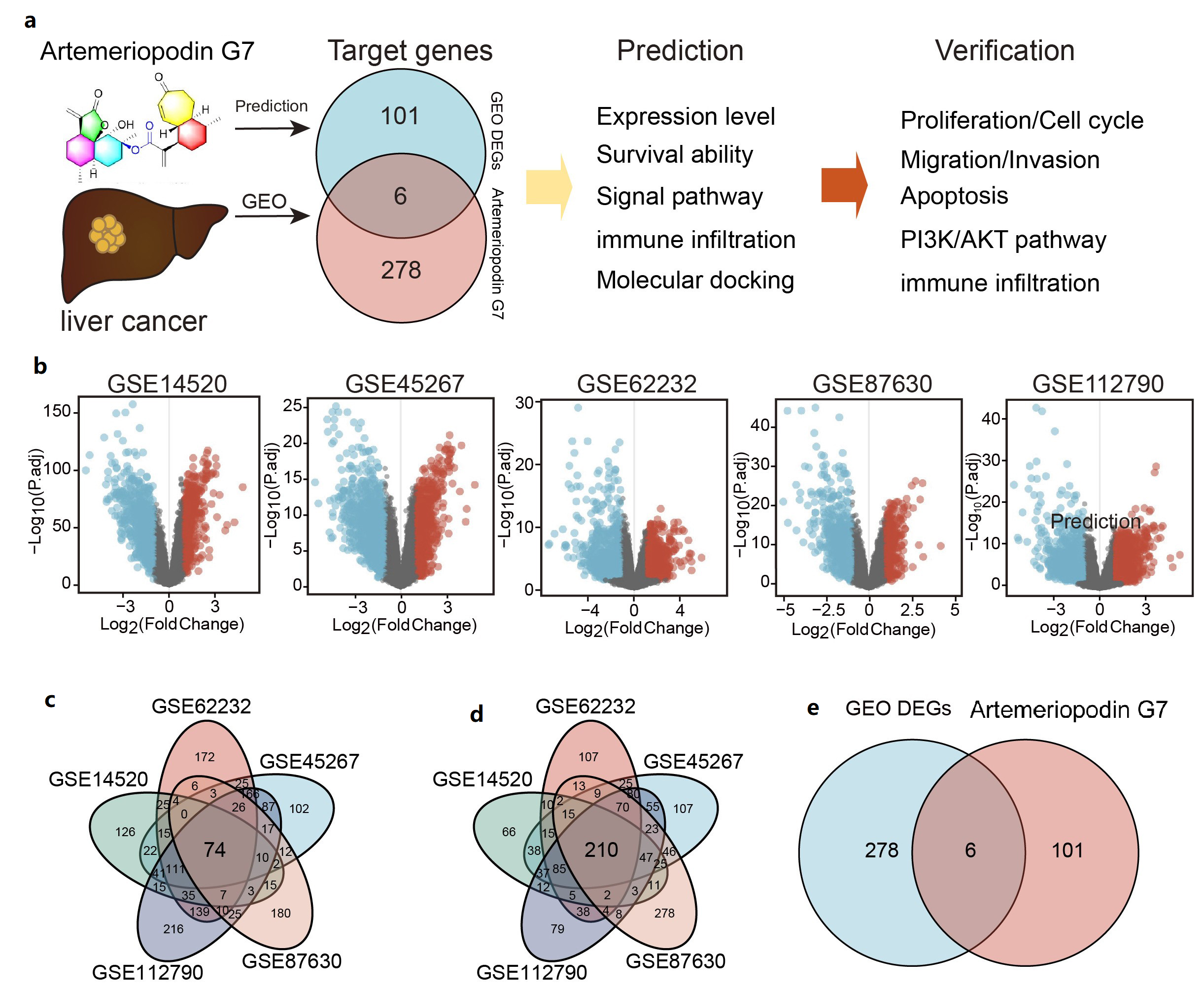
**

**supplementary Fig. 7** Potential target genes of artemeriopodin G7 (**33**) in HCC. (**a**) Schematic diagram of investigation on the target and mechanism analysis of artemeriopodin G7 (**33**). (**b**) Volcano map of DEGs in GSE14520, GSE45267, GSE62232, GSE87630 and GSE112790. (**c**) The Venny results of low expression DEGs in HCC. (**d**) The Venny results of high expression DEGs in HCC. (**e**) The Venny results of potential target genes of artemeriopodin G7 (**33**) for HCC.


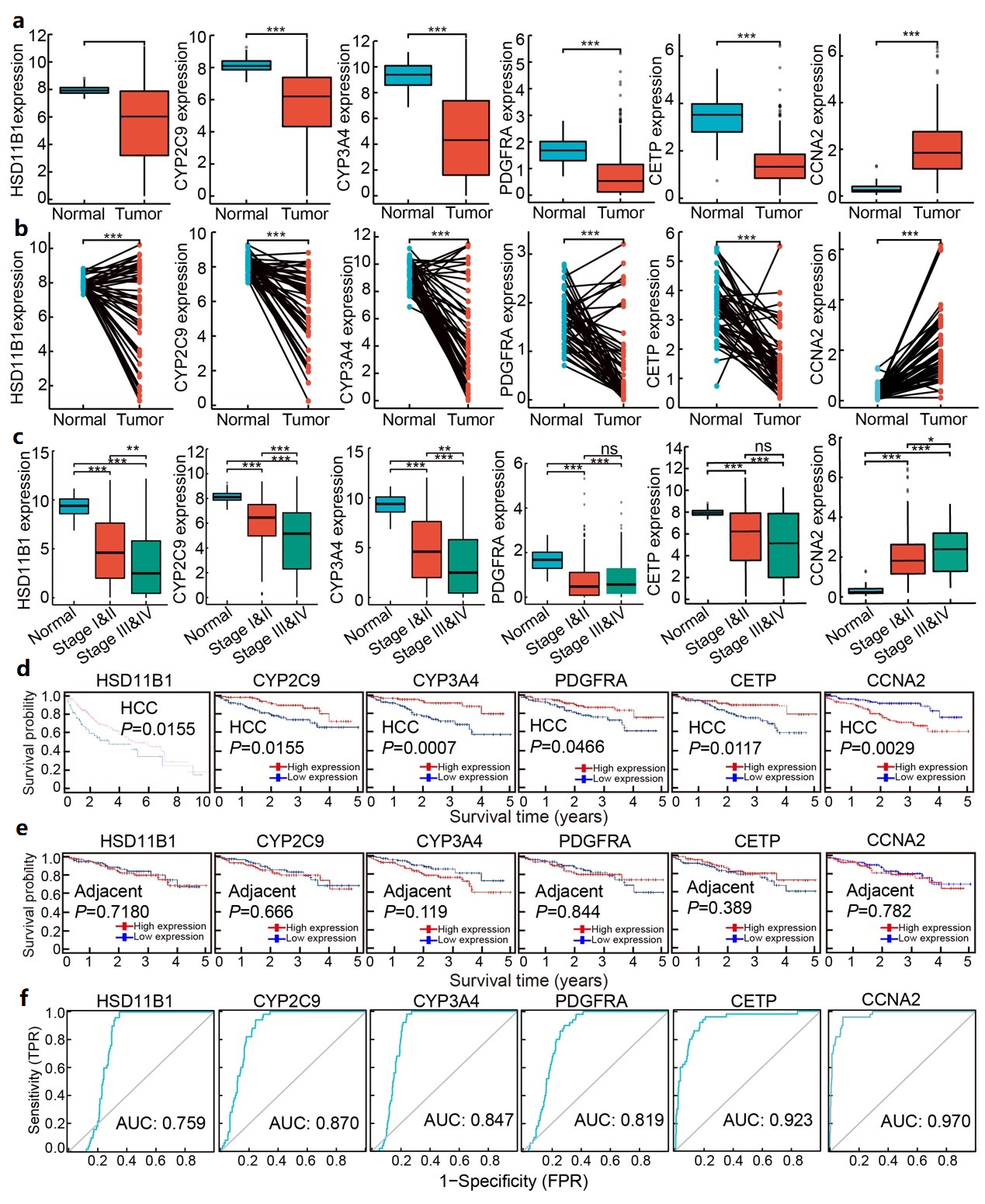


**supplementary Fig. 8.** Artemeriopodin G7 (**33**) targets downregulation correlates with a worse survival outcome in in HCC Patients. (**a** and **b**) The relative expression levels of HSD11B1, CYP2C9, CYP3A4, PDGFRA, CETP and CCNA2 in HCC. (**c**) HSD11B1, CYP2C9, CYP3A4, PDGFRA, CETP and CCNA2 expression levels negative correlate with pathological stages. (**d** and **e**) HSD11B1, CYP2C9, CYP3A4, PDGFRA, CETP and CCNA2 low expression correlates with worse survival rates in HCC, but had no difference in adjacent tissues. (**f**) The ROC curves for HSD11B1, CYP2C9, CYP3A4, PDGFRA, CETP and CCNA2 in LIHC using TCGA dataset. * P < 0.05, ** P < 0.01, *** P < 0.001.


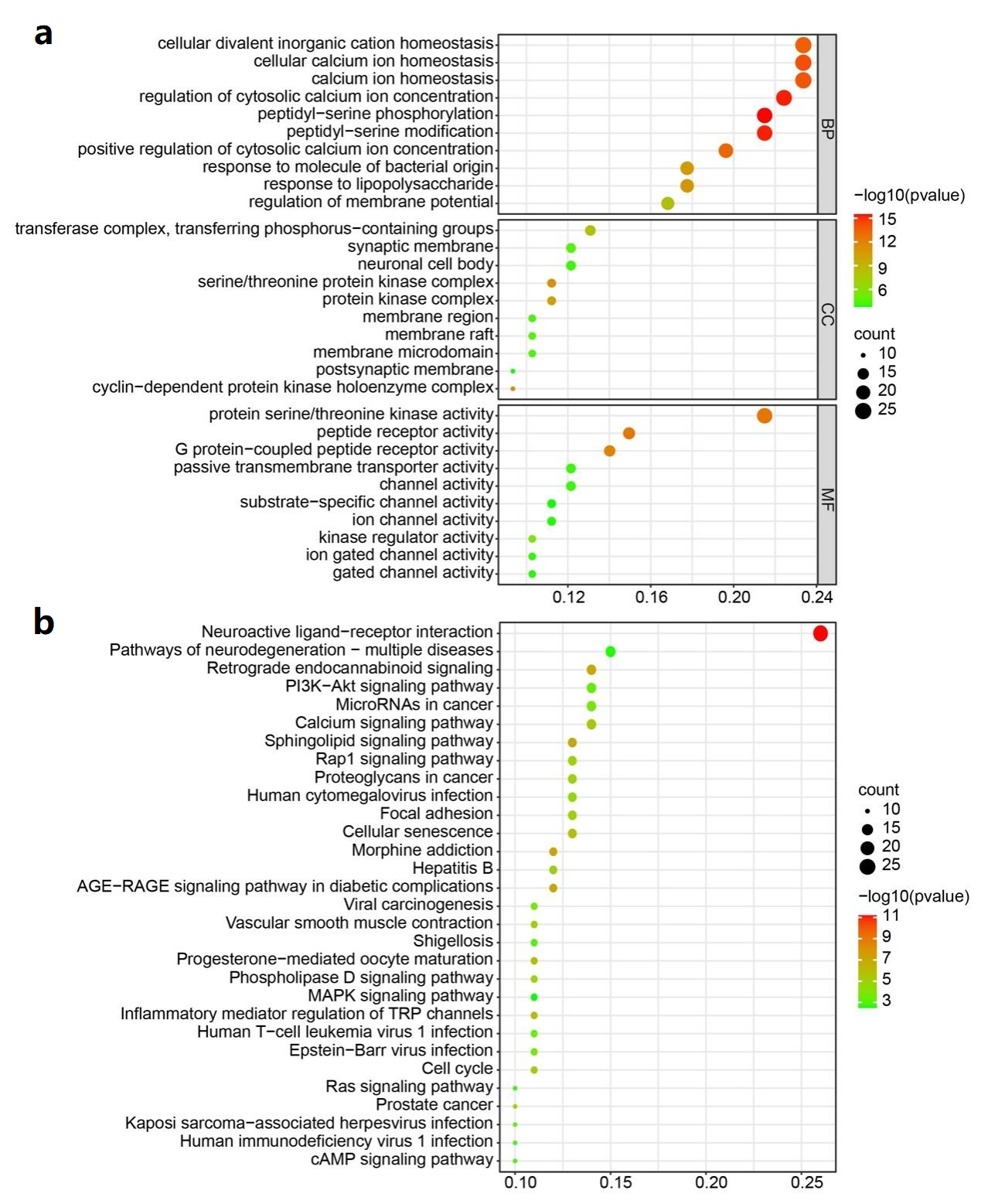


**supplementary Fig. 9** GO and KEGG analyses for biological function of potential target genes of artemeriopodin G7 (**33**) in HCC. (**a**) Histogram of GO enrichment analysis of targets. (**b**) KEGG enrichment analysis of targets.


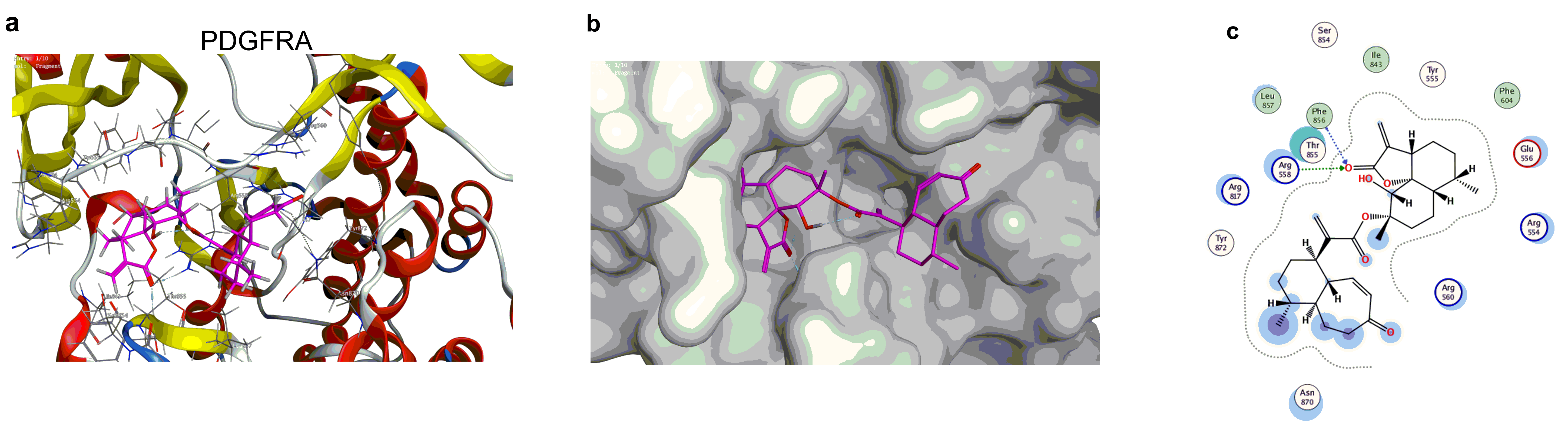


**supplementary Fig. 10** Binding capacity between artemeriopodin G7 (**33**) and PDGFRA by molecular docking.

**
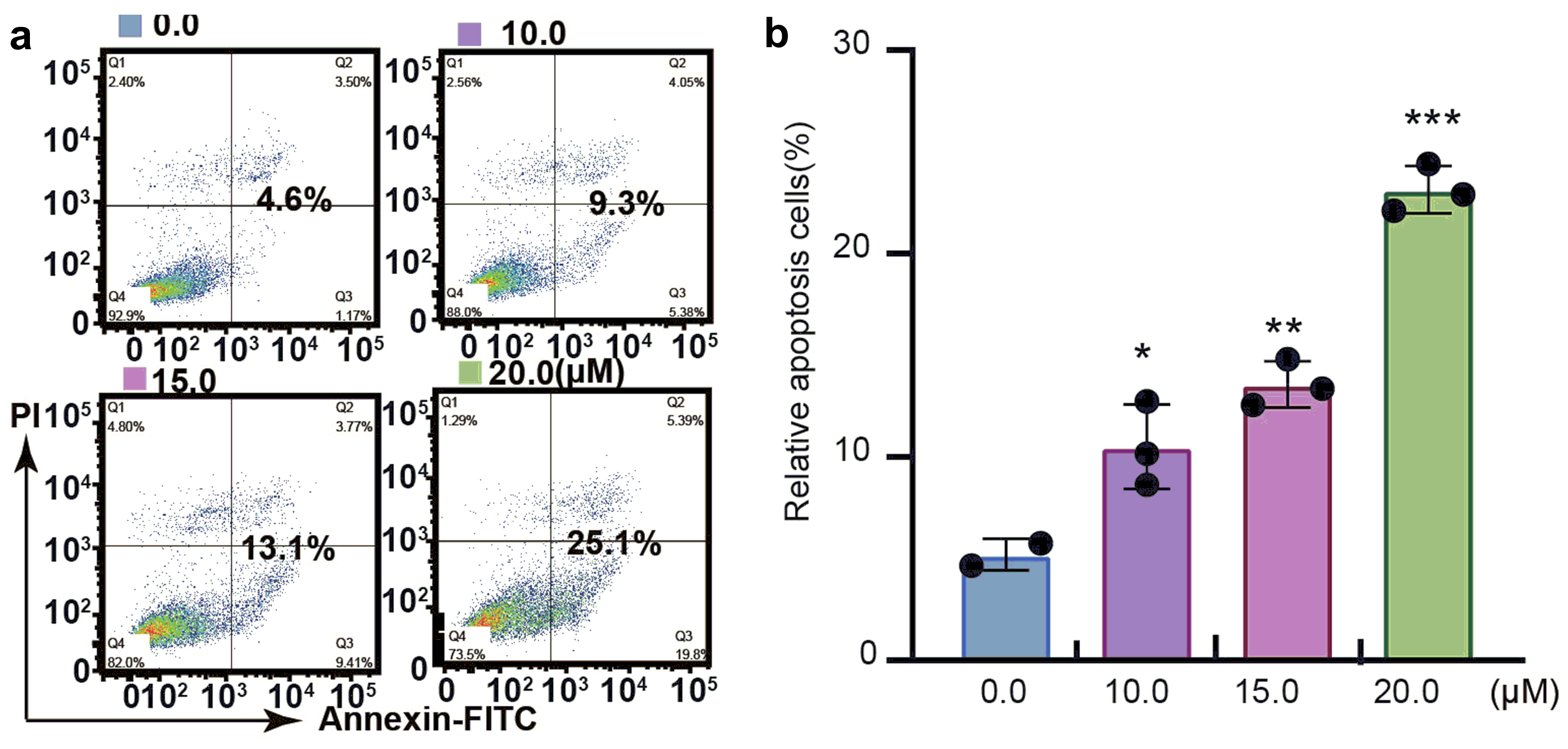
**

**supplementary Fig. 11** Compound **33** induced HepG2 cells apoptosis. **a** HepG2 cells were treated with different concentrations (0.0, 10.0, 15.0 and 20.0 μM) of compound **33** for 48 h, flow cytometric analysis and cell apoptosis quantification of HepG2 cells. **b** is the quantification data for **a**. **P*<0.05, ***P*<0.01, and ****P*<0.001, n = 3.


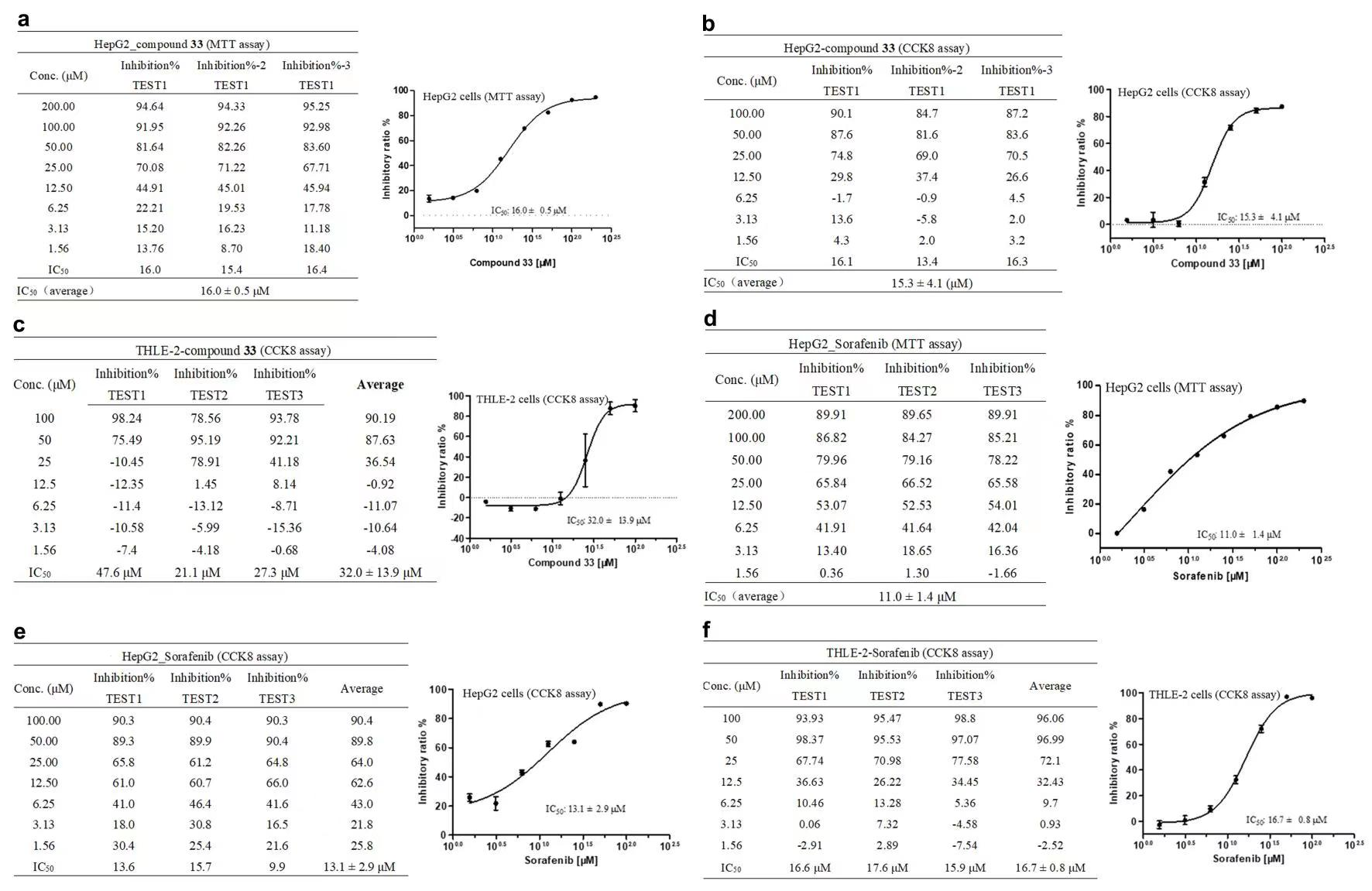


**supplementary Fig. 12** Cytotoxicity of compound **33** were evaluated by CCK8 and MTT assay to compare with sorafenib on THLE-2 and HepG2 cells. **a** Inhibitory ratios of compound **33** on HepG2 cells at different concentrations by MTT assay. **b** Inhibitory ratios of compound **33** on HepG2 cells at different concentrations by CCK8 assay. **c** Inhibitory ratios of compound **33** on THLE-2 cells at different concentrations by CCK8 assay. **d** Inhibitory ratio of sorafenib on HepG2 cells at different concentrations by MTT assay. **e** Inhibitory ratio of sorafenib on HepG2 cells at different concentrations by CCK8 assay. **f** Inhibitory ratios of sorafenib on THLE-2 cells at different concentrations by CCK8 assay.

**supplementary Fig. 13** PDGFRA proteins were examined in HepG2 cells by Western blot. **a** The expression of PDGFRA proteins in HepG2 cells. **b** The quantification data for **a**.


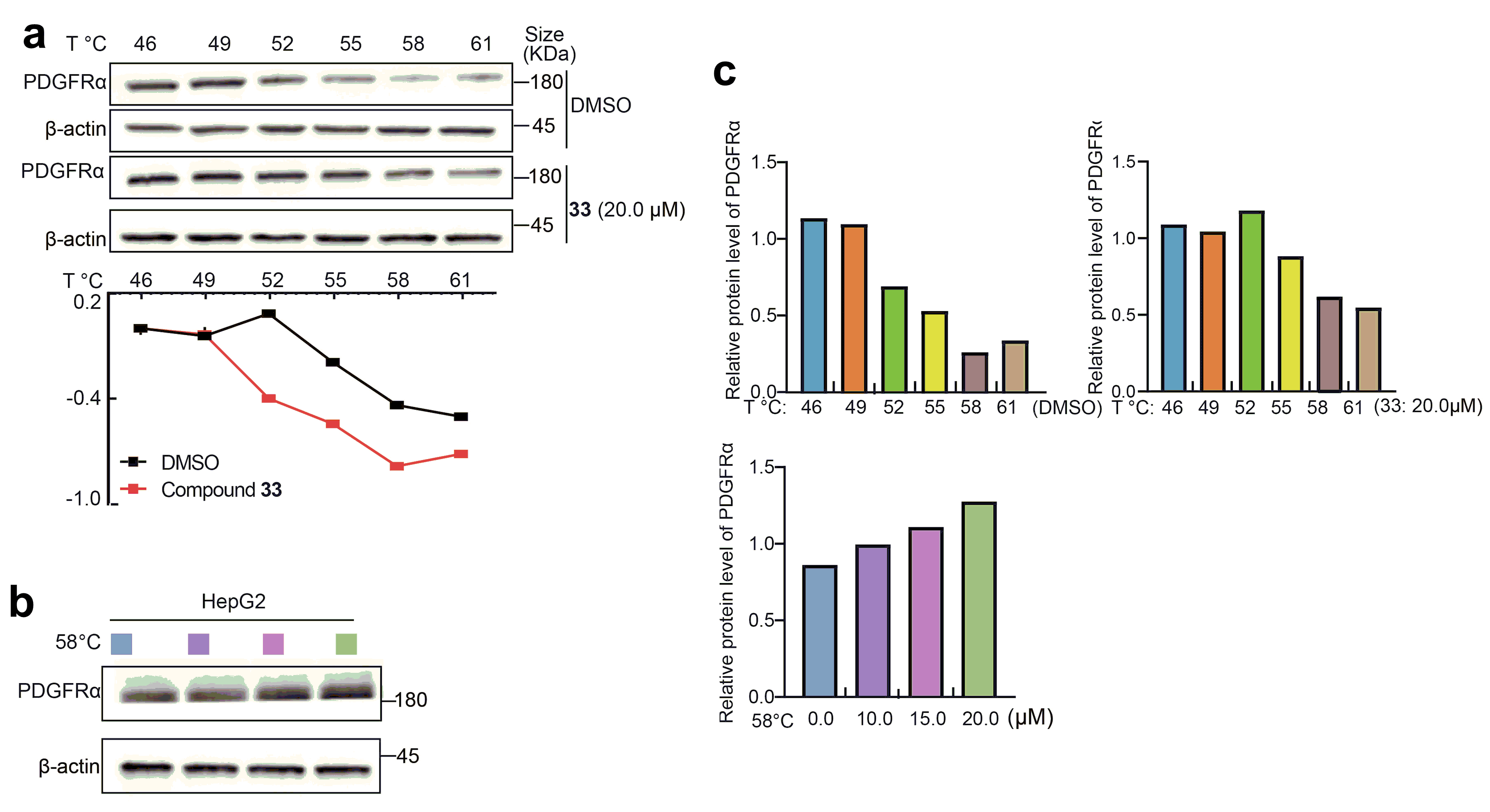


**supplementary Fig. 14** CETSA analysis of intracellular binding between compound **33** and PDGFRA protein. **a** Protein levels were investigated at different temperatures under the treatment of **33** (20.0 μM) in HepG2 cells. **b** Protein levels were investigated at different concentration of **33** (58 ℃). **c** The quantification data for **a** and **b**.


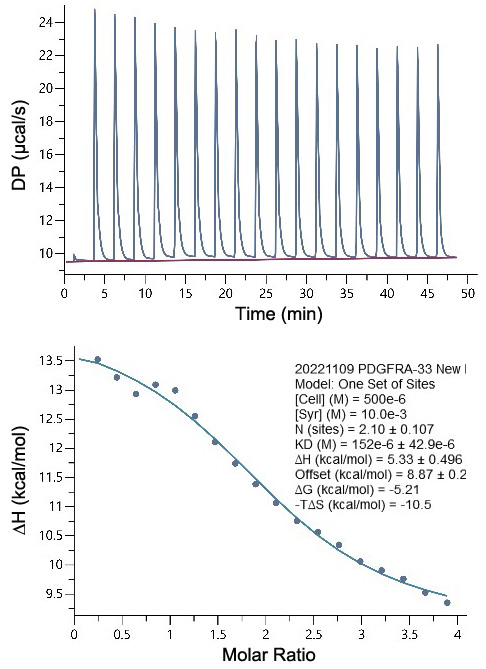


**supplementary Fig. 15** Isothermal titration calorimetry (ITC) enthalpogram of the interaction between **33** and PDGFRA. The titration curve is depicted as a function of the molar ratio between PDGFRA and the calculated concentration of **33** in the assay.


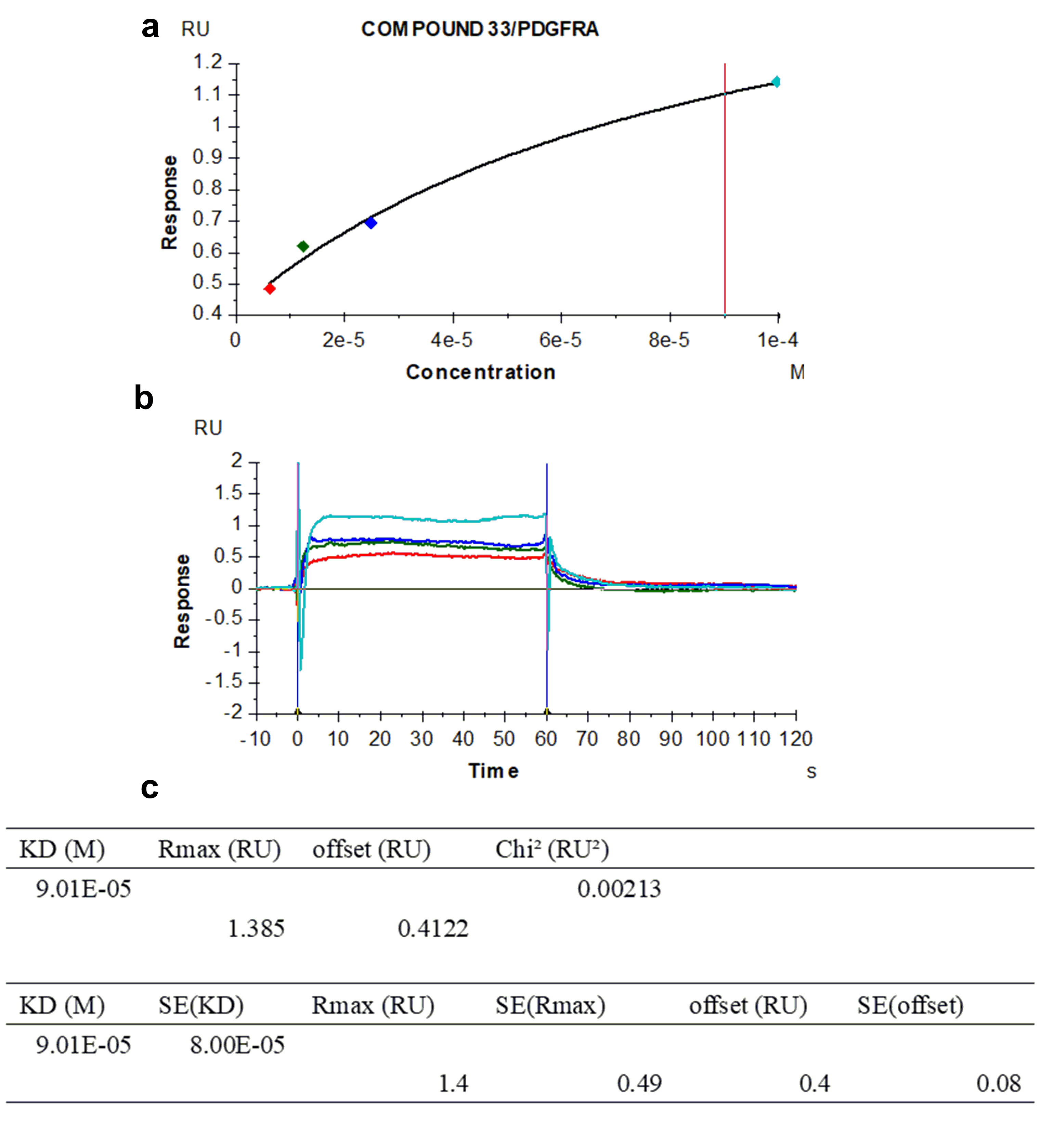


**supplementary Fig. 16** Compound **33** directly target to PDGFRA by Surface Plasmon Resonance (SPR) assay. **a.** KD value (90.1 μM) for compound **33** and PDGFRA binding was measured with BIAcore evaluation software. **b** Compound **33** binds to PDGFRA as determined by SPR. **c** The quantification data for **a** and **b**.


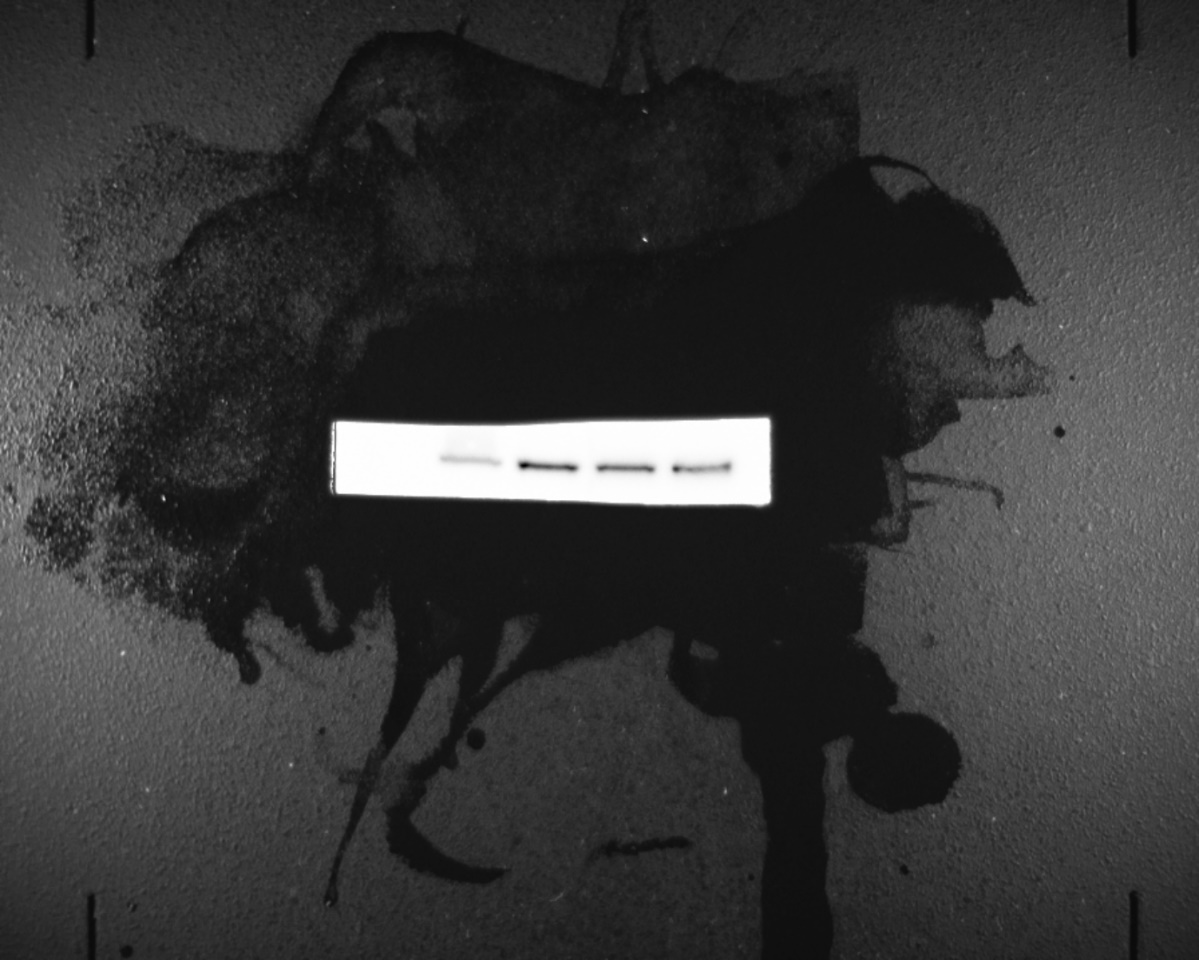


CyclinB1

Compound **33** 0.0 10.0 15.0 20.0 μM


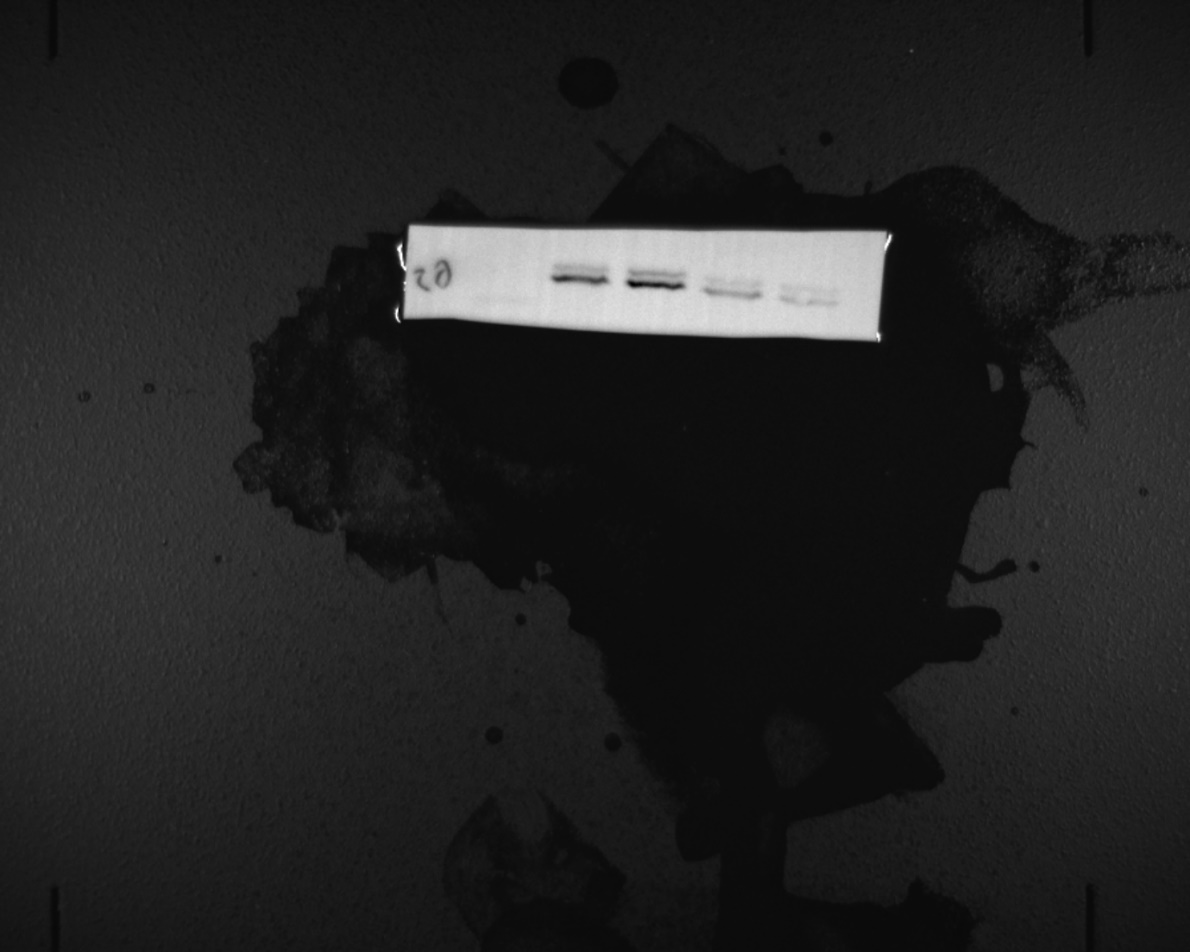


p-cdc2

35

Compound **33** 0.0 10.0 15.0 20.0 μM

Size

KDa


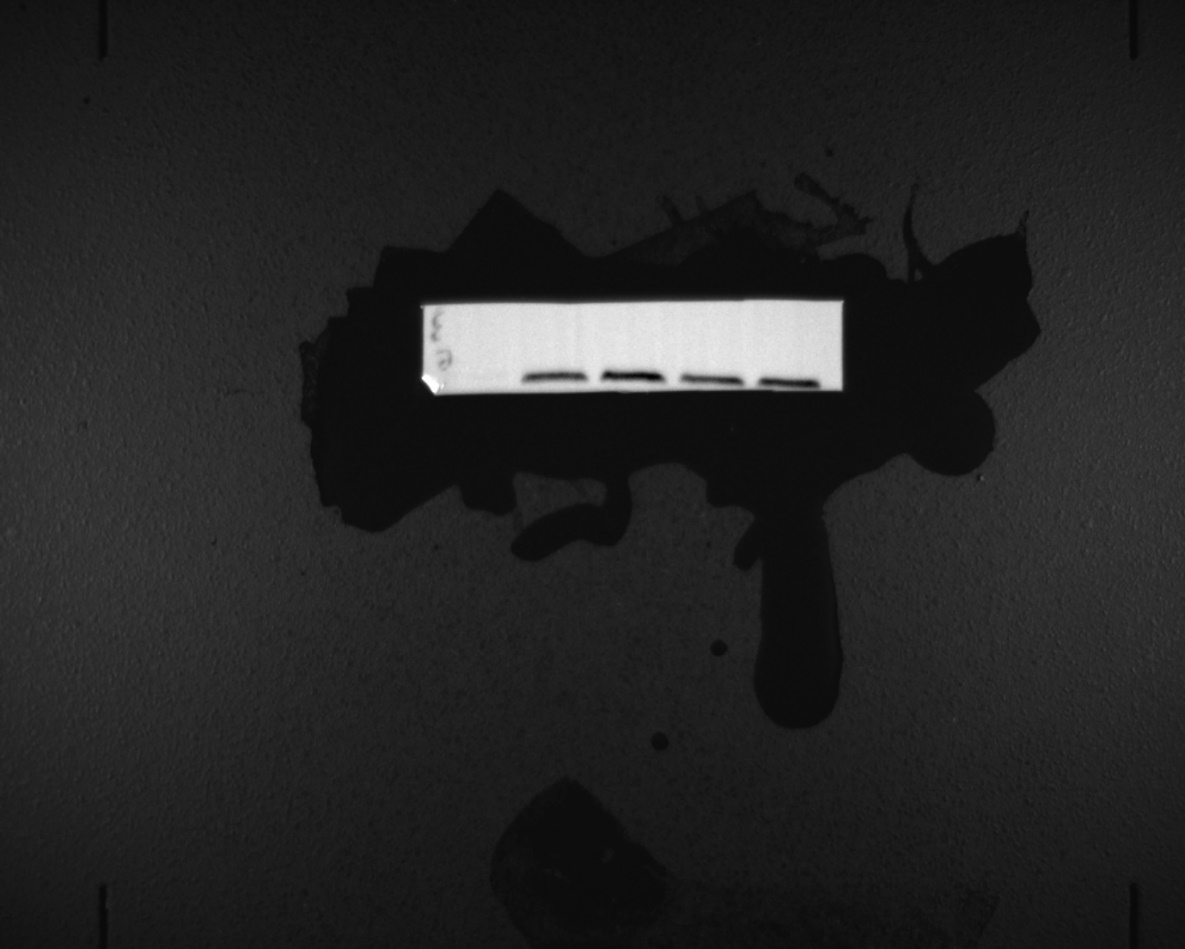


Compound **33** 0.0 10.0 15.0 20.0 μM

cdc2

35

Size

KDa


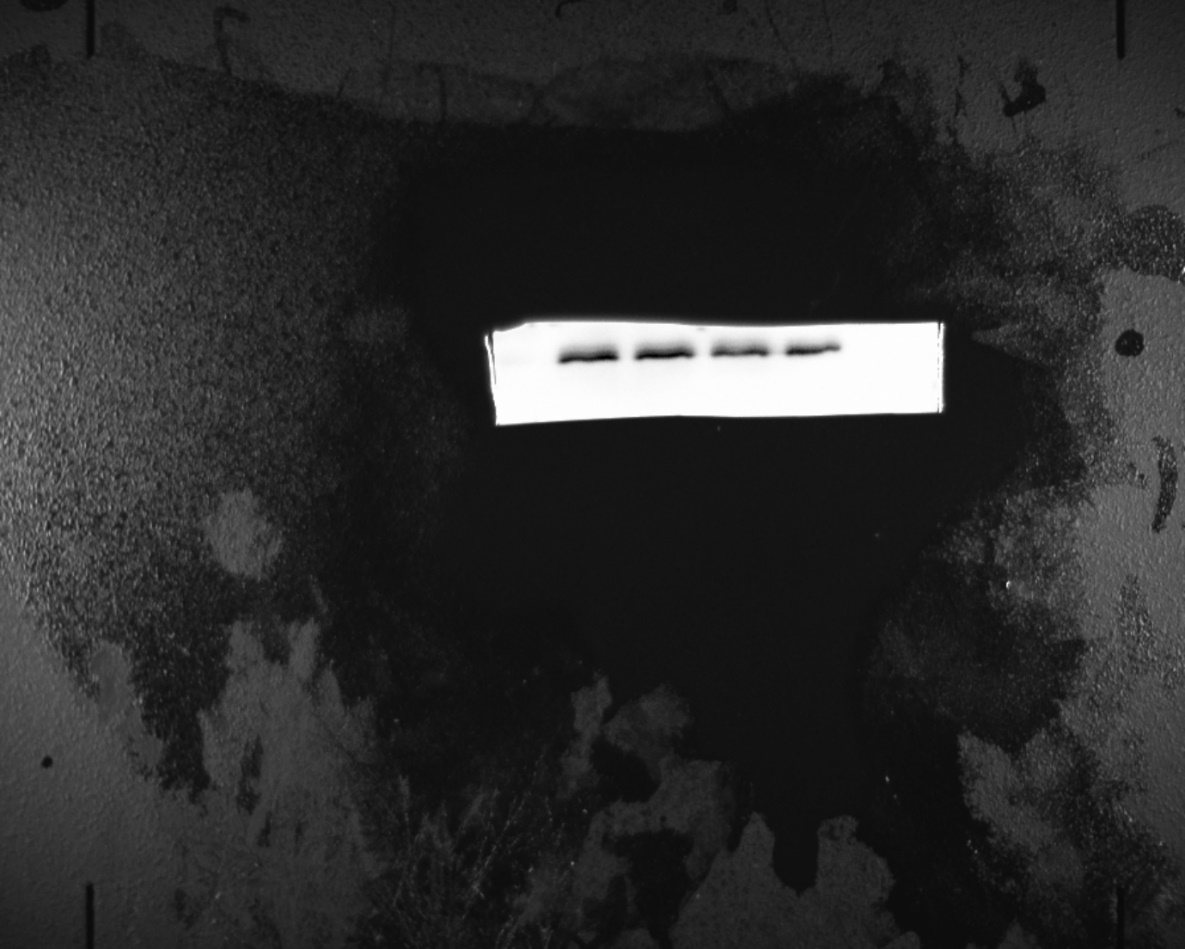


Compound **33** 0.0 10.0 15.0 20.0 μM

β-actin

Size

KDa

45

**supplementary Fig. 17a** All original and uncropped films of Western blots of the key-related proteins with cell cycle.


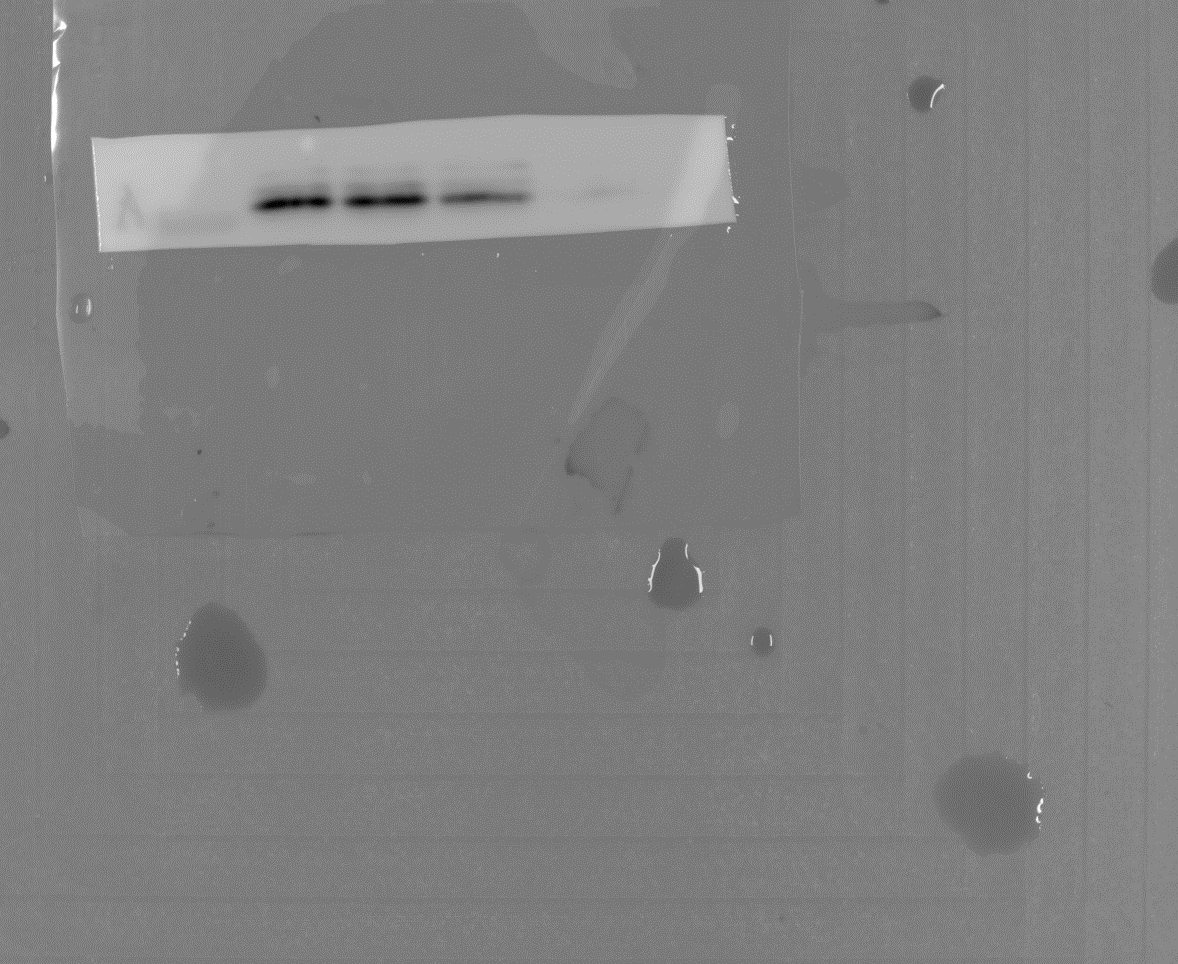


Compound **33**  0.0 10.0 15.0 20.0 μM

Size

KDa

Bcl-2

25


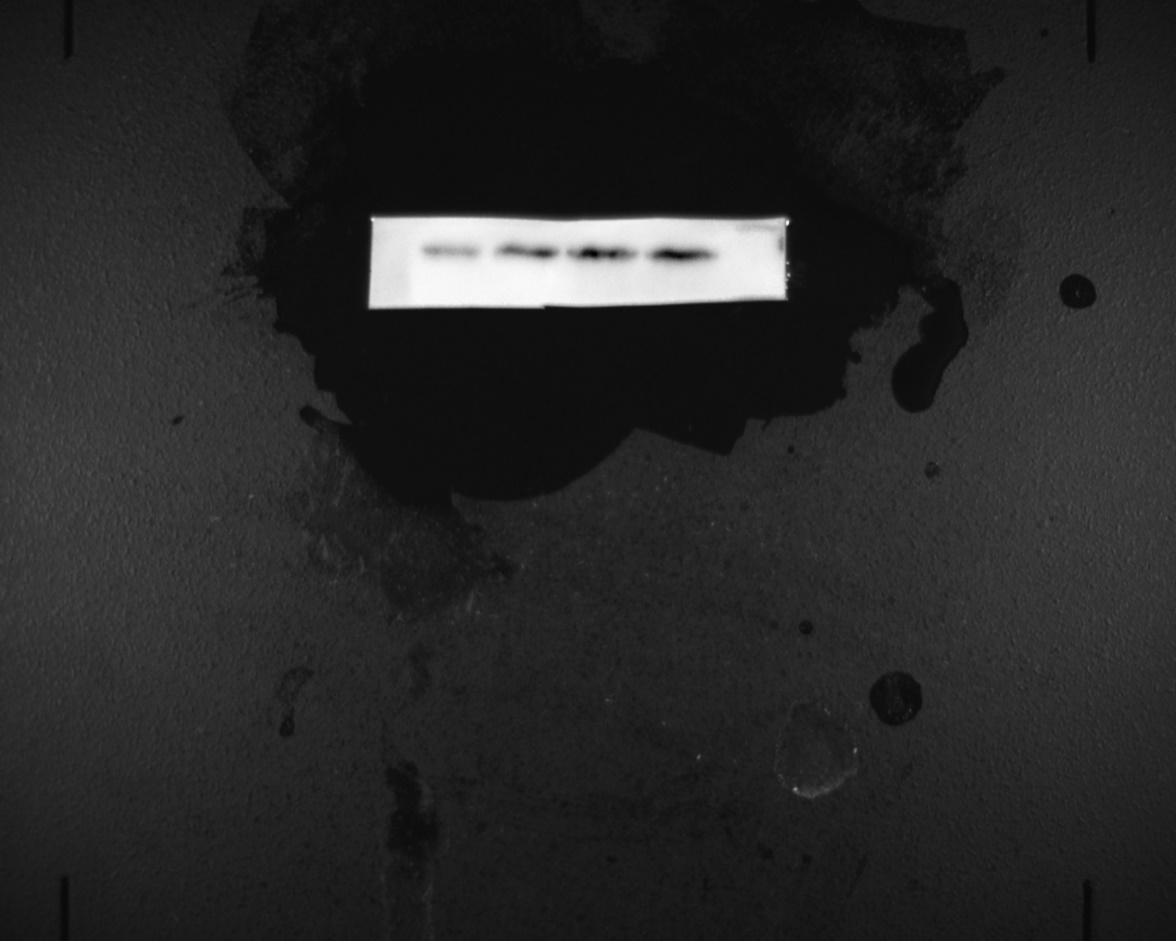


Compound **33** 0.0 10.0 15.0 20.0 μM

Bax

Size

KDa

25


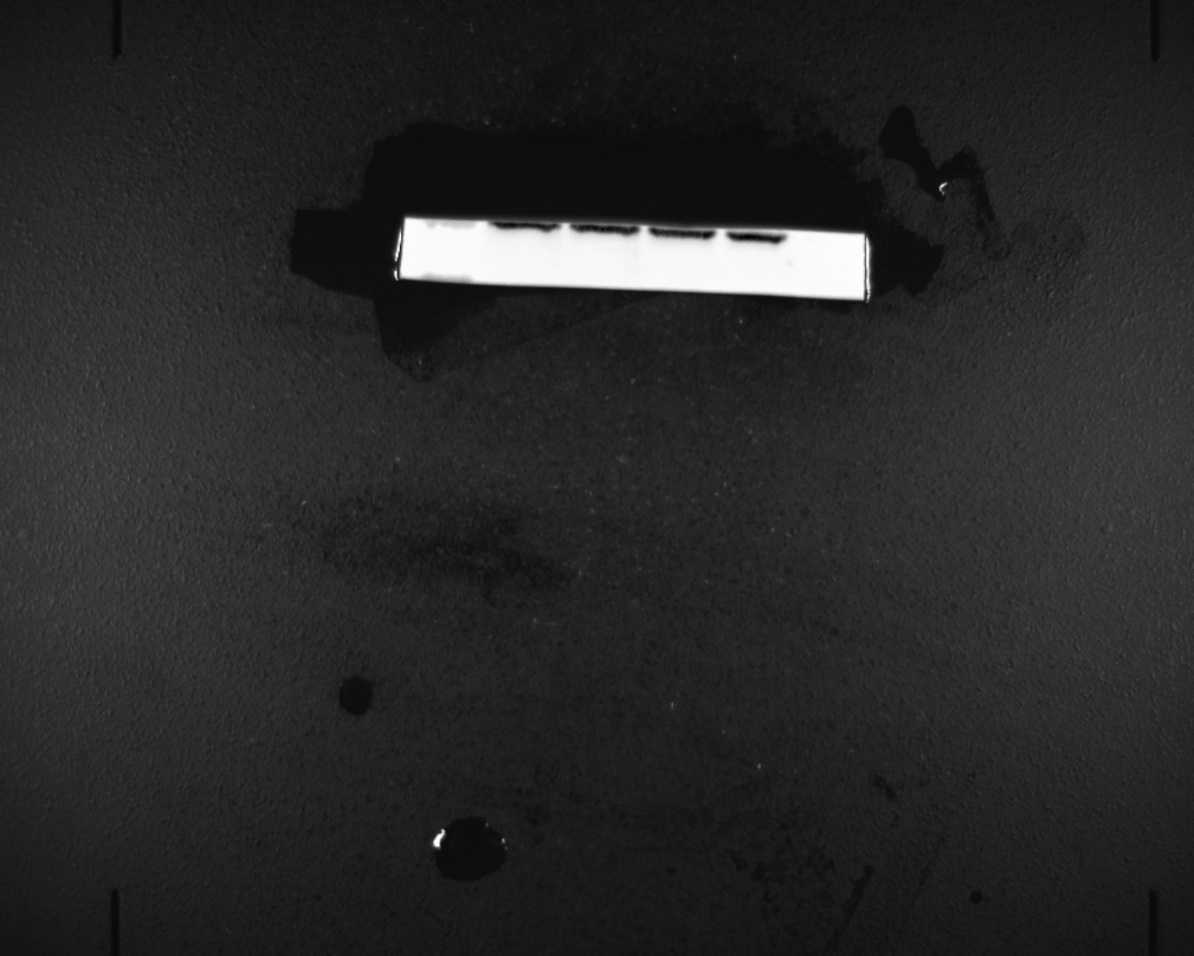


Compound **33** 0.0 10.0 15.0 20.0 μM

β-actin

Size

KDa

45

**supplementary Fig. 17b** All original and uncropped films of Western blots of the key-related proteins with cell apoptosis.


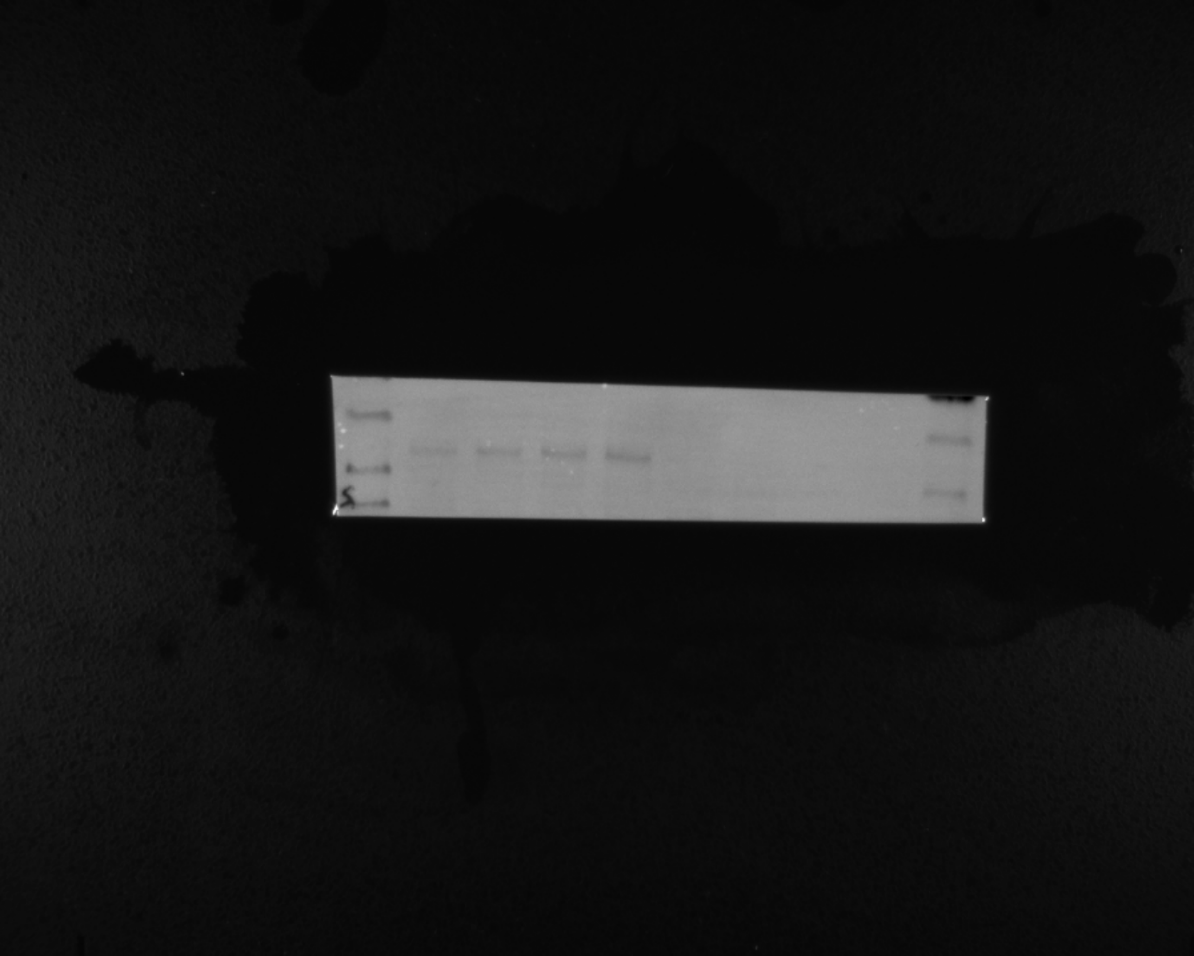


Compound **33** 0.0 10.0 15.0 20.0 μM

PDGFRα

Size

KDa

245

180


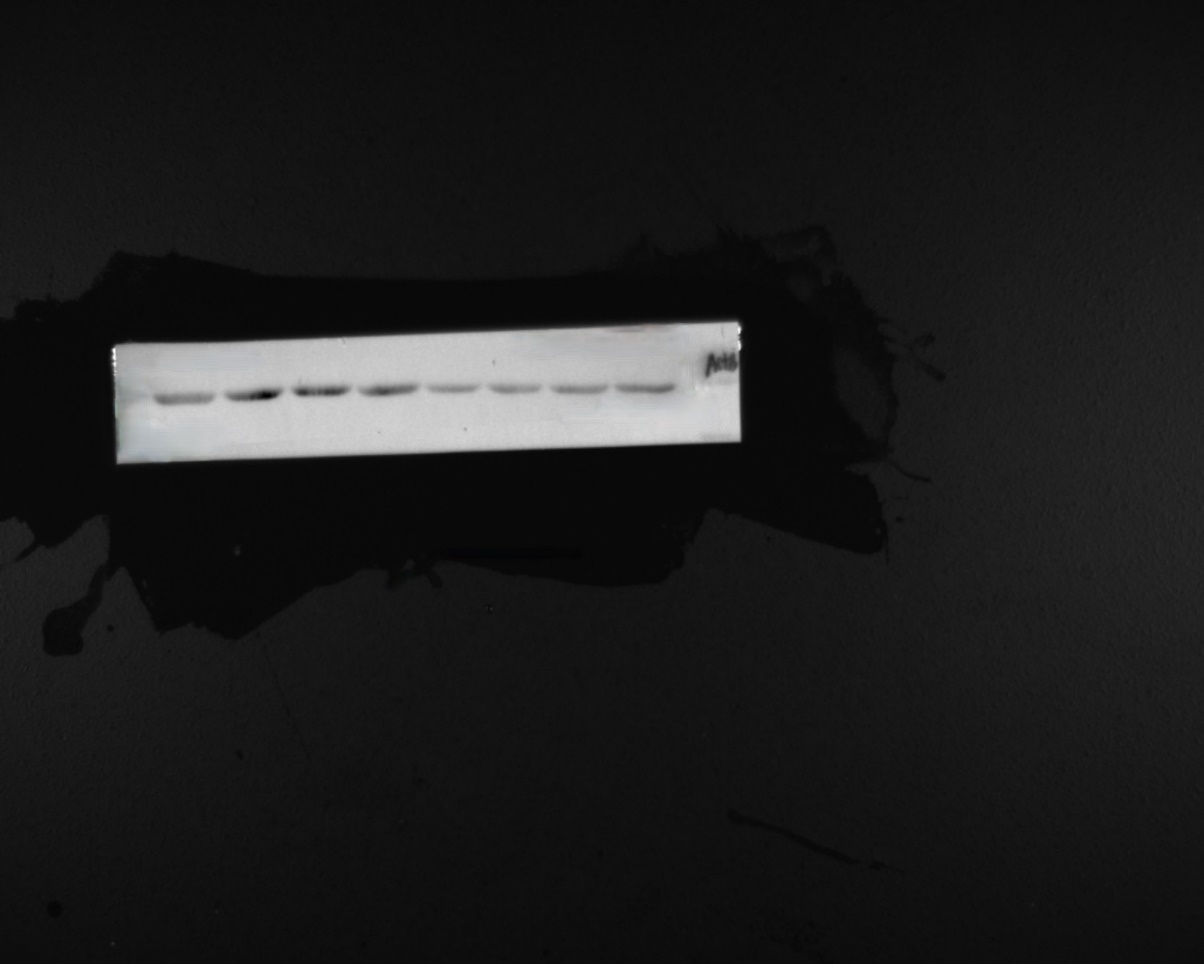


Compound **33** 0.0 10.0 15.0 20.0 μM

β-actin

Size

KDa

45

**supplementary Fig. 17c** All original and uncropped films of Western blots of protein PDGFRA.


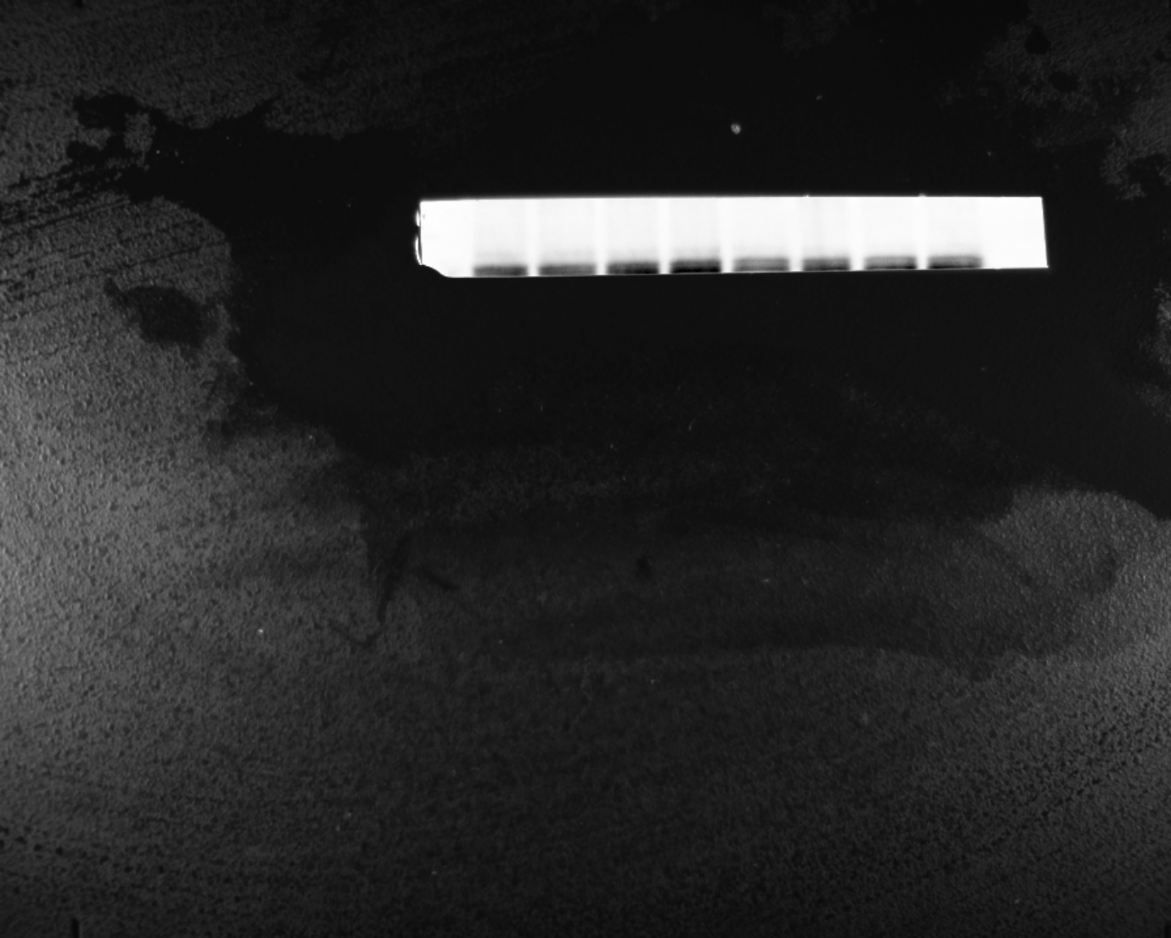


58℃ Compound **33** 0.0 10.0 15.0 20.0 μM

PDGFRα

Size

KDa

180


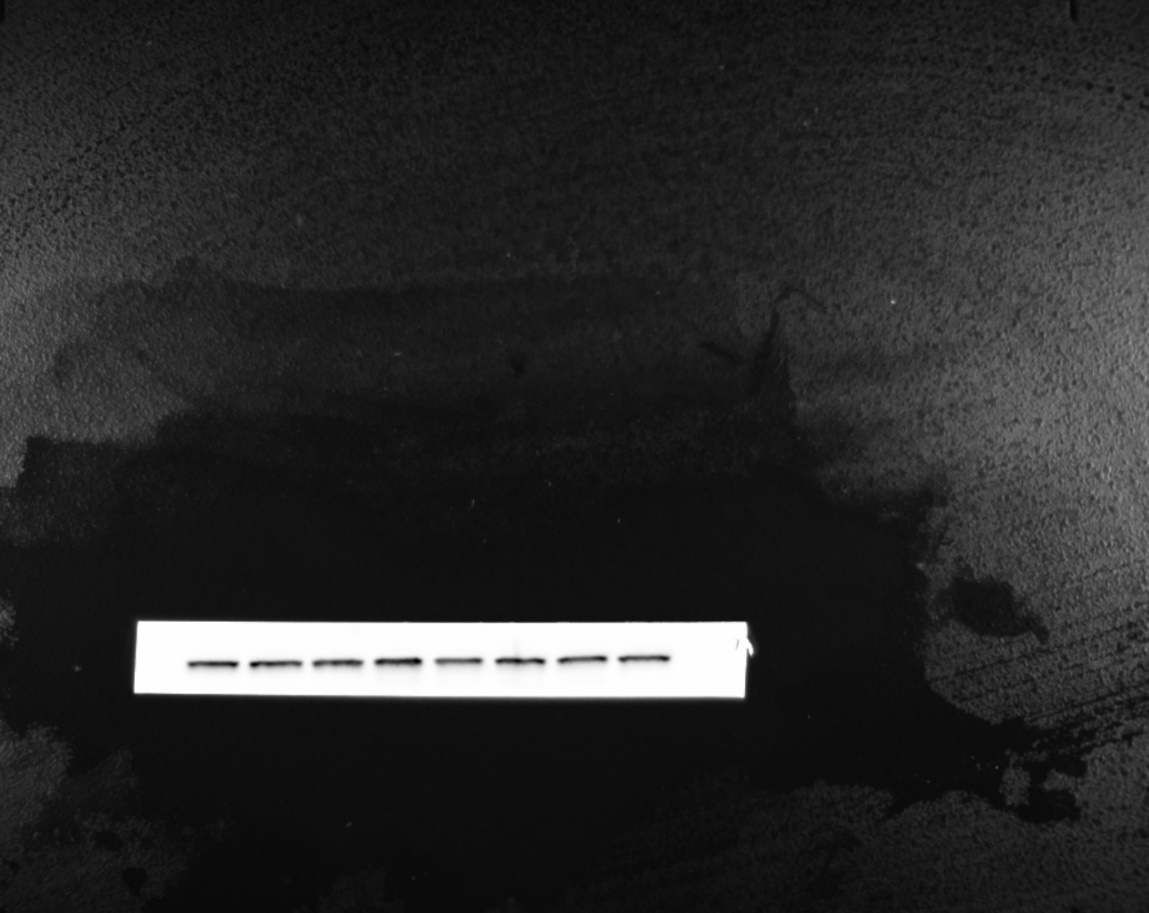


Compound **33** 0.0 10.0 15.0 20.0 μM

β-actin

**supplementary Fig. 17d** All original and uncropped films of Western blots of protein PDGFRA at 58 ℃ (CETSA analysis).


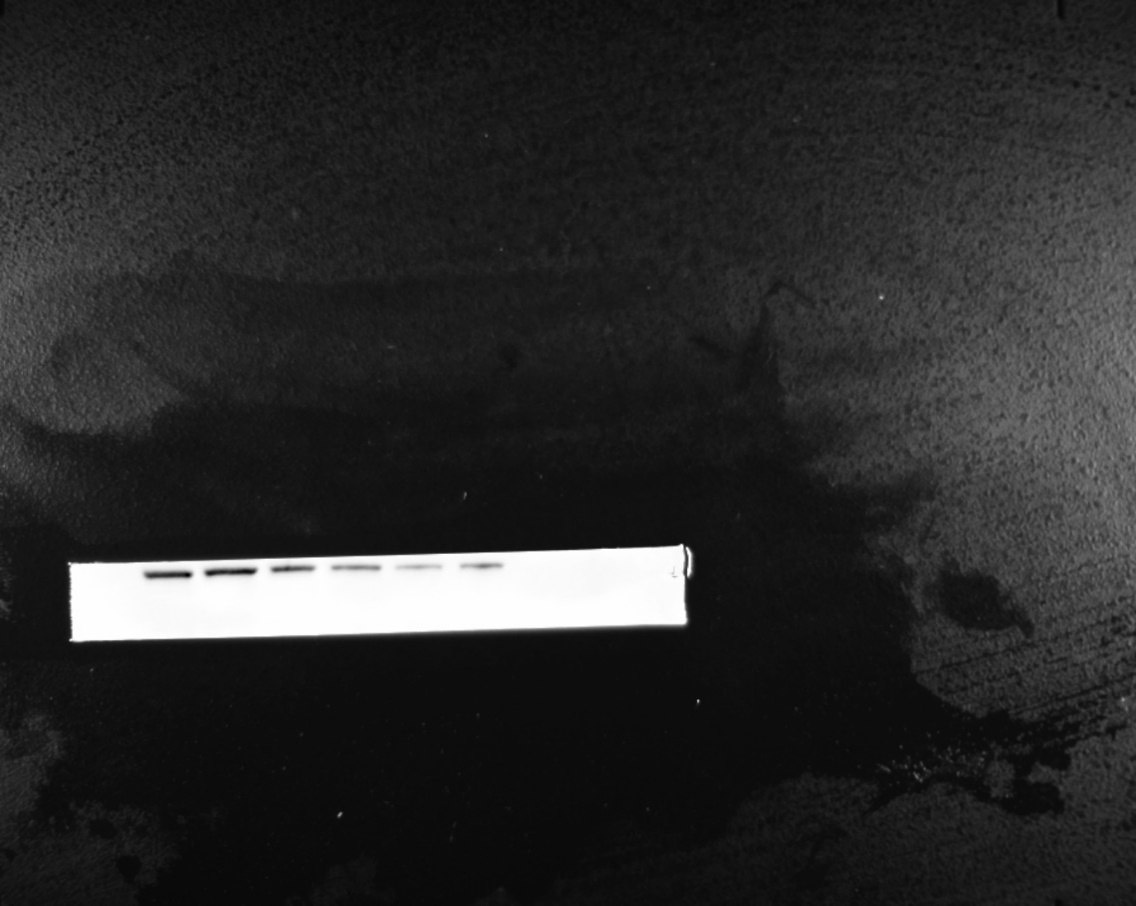


T℃ 46 49 52 55 58 61

PDGFRα (DMSO)


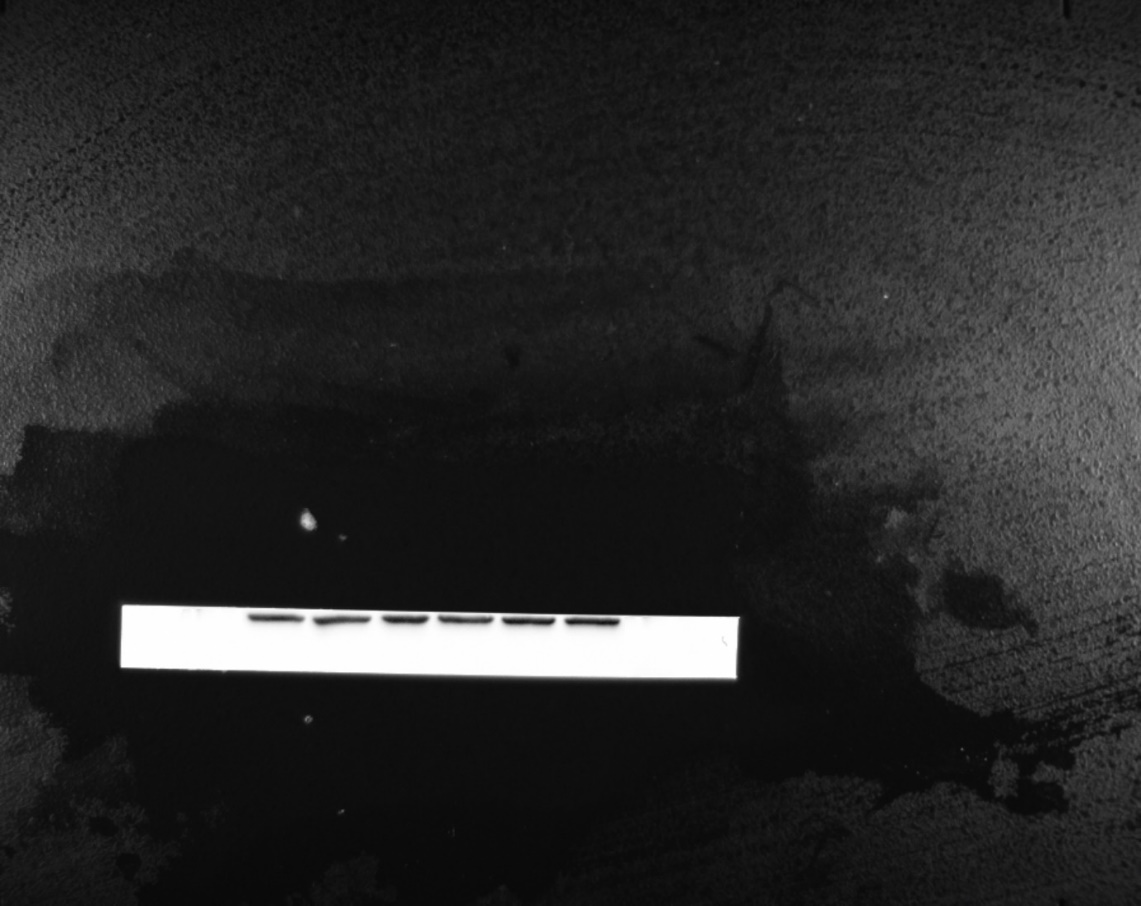


β-actin (DMSO)

T℃ 46 49 52 55 58 61


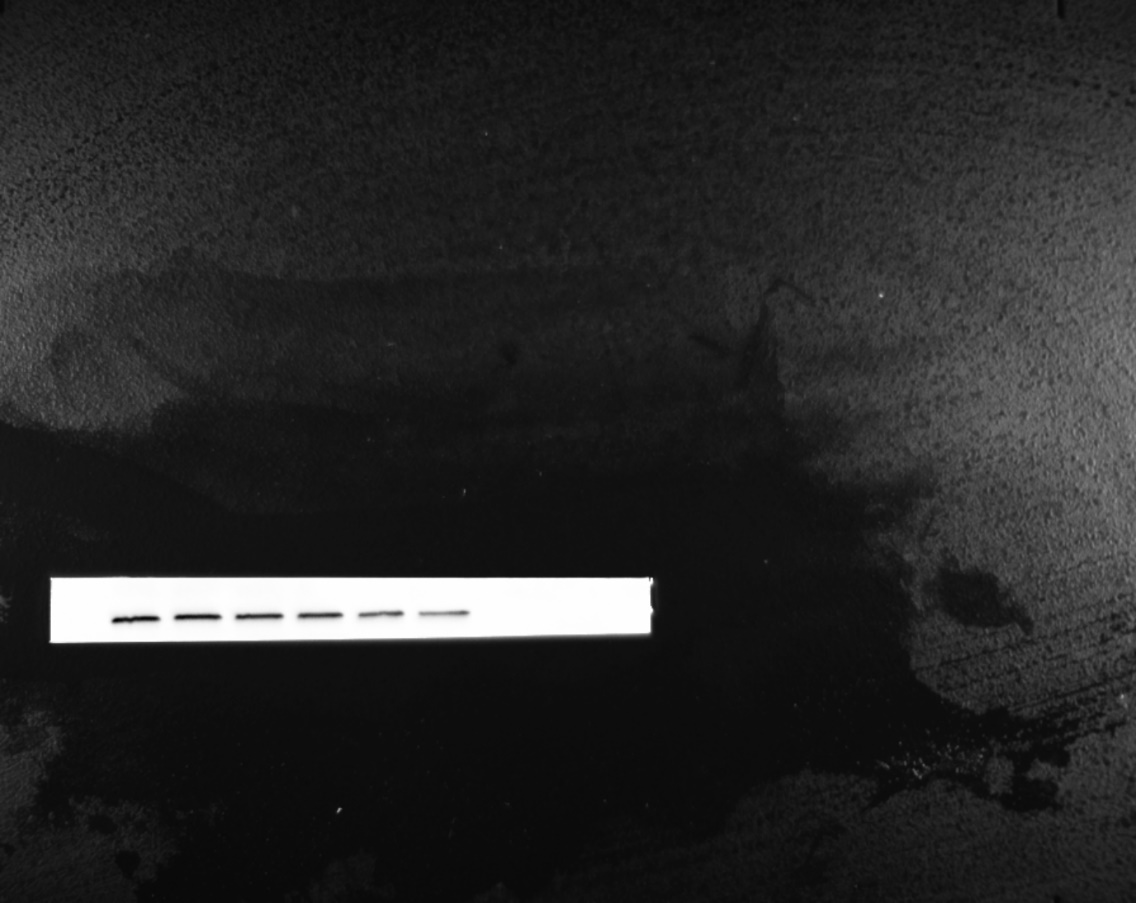


T℃ 46 49 52 55 58 61

PDGFRα (**33 20** μM)


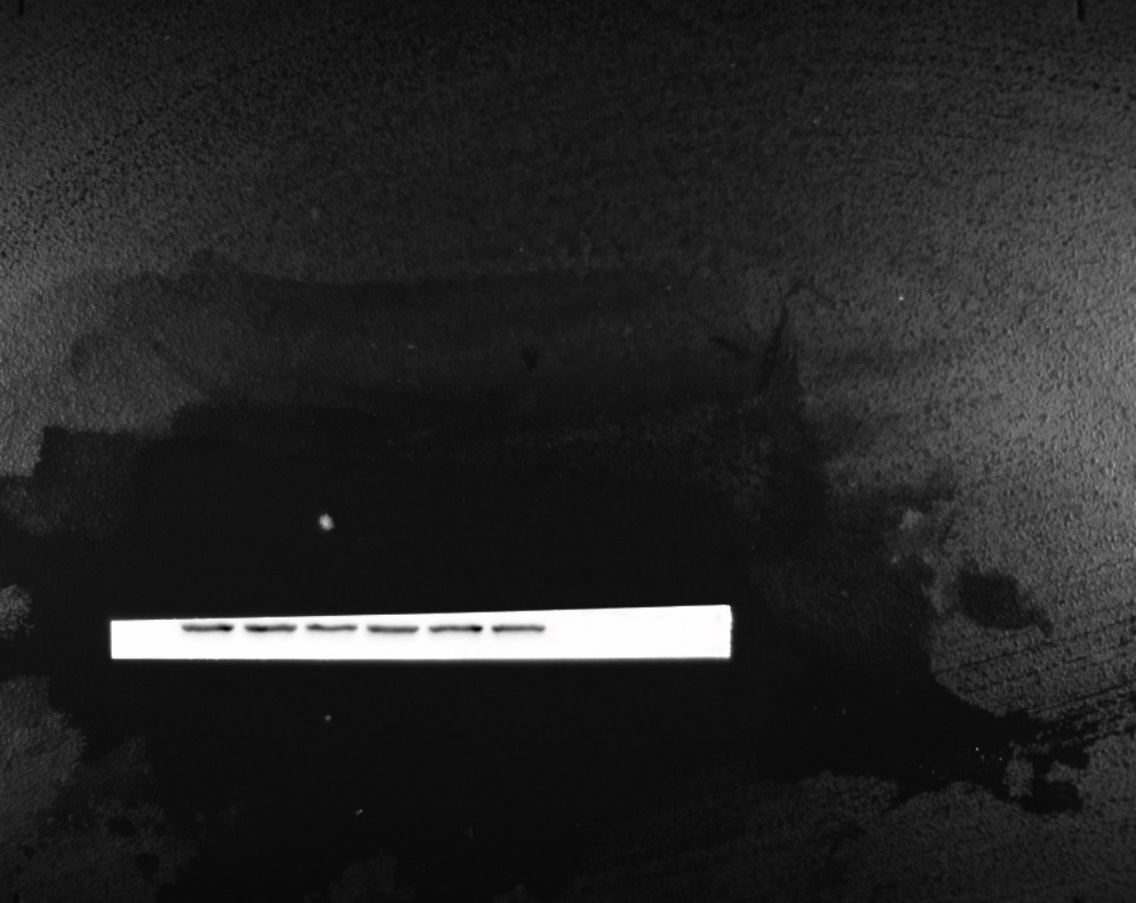


T℃ 46 49 52 55 58 61

β-actin (**33 20**μM)

**supplementary Fig. 17e** All original and uncropped films of Western blots of protein PDGFRA at different temperatures under the treatment of **33** (20.0 μM) (CETSA analysis).


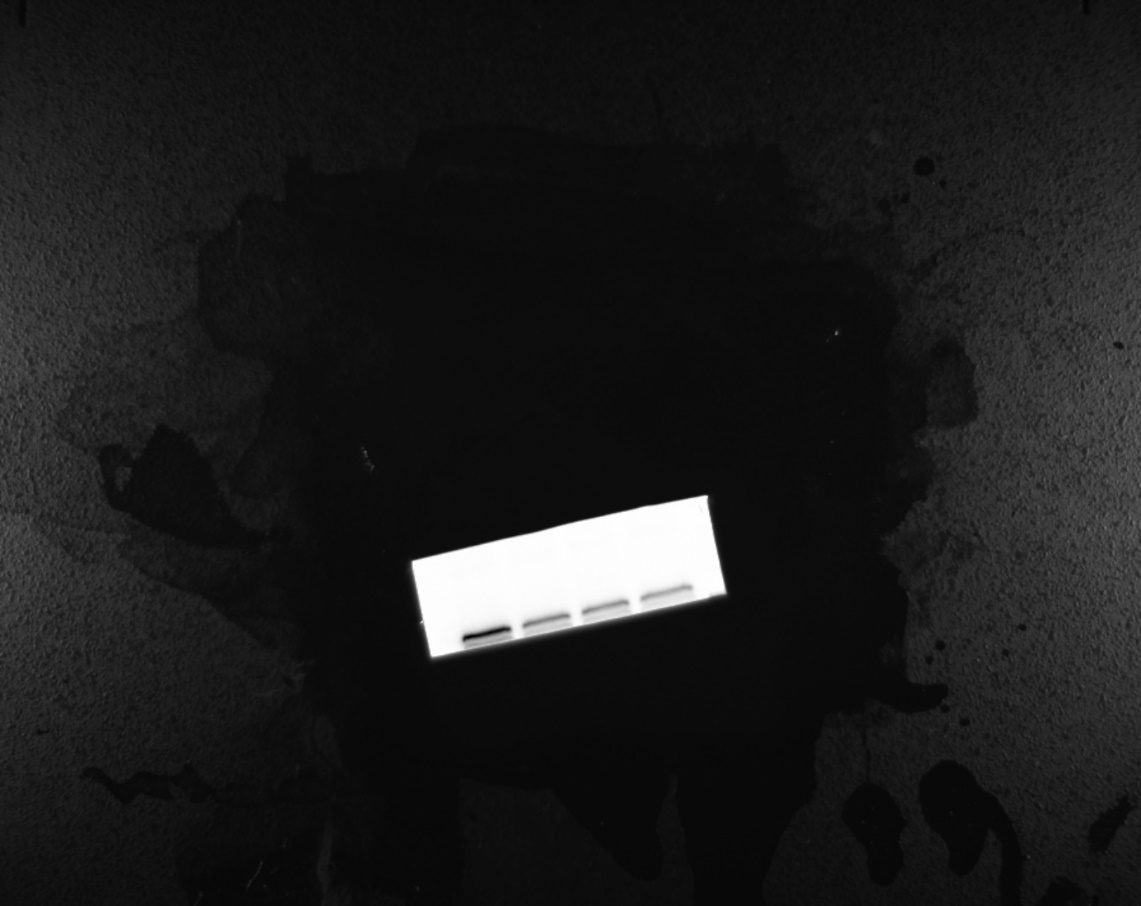


Compound **33** 0.0 10.0 15.0 20.0 μM

P-STAT3^Y785^

Size

KDa 75


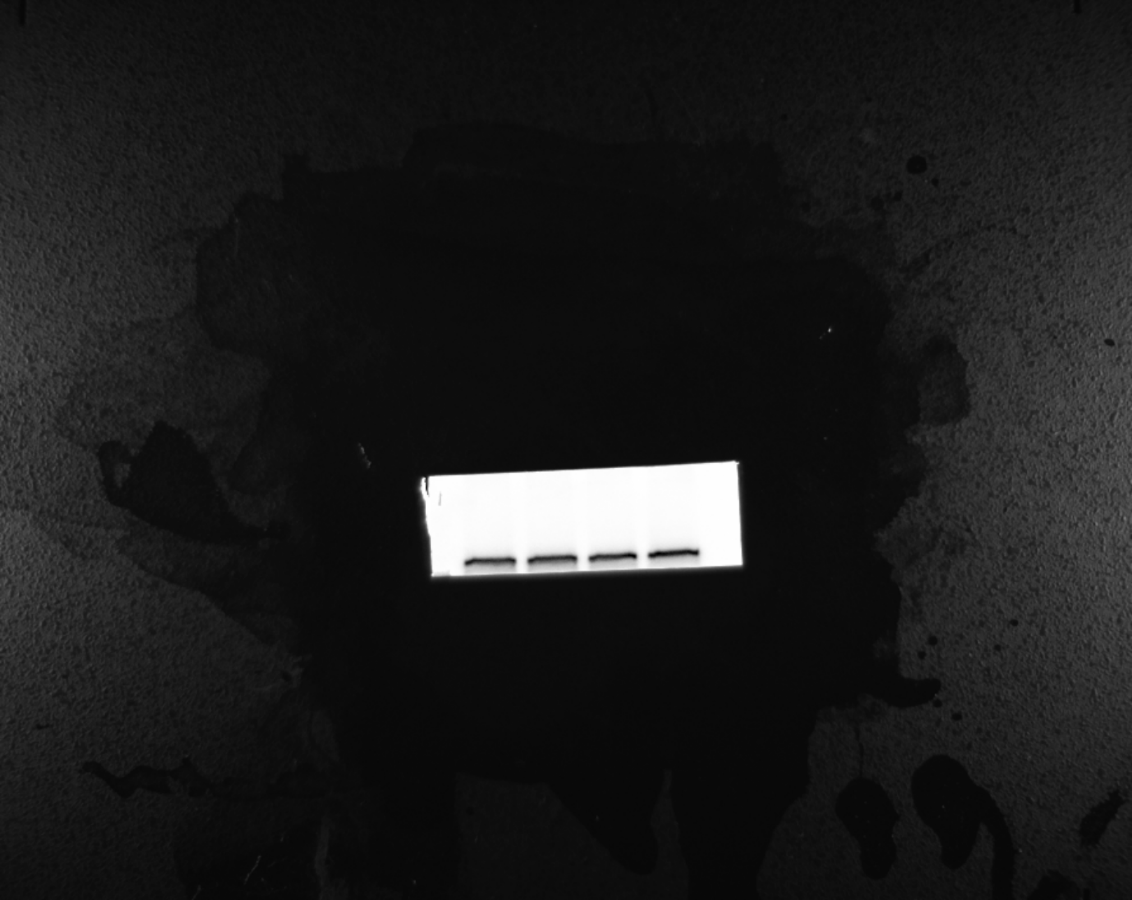


Compound **33** 0.0 10.0 15.0 20.0 μM

STAT3

Size

KDa 75


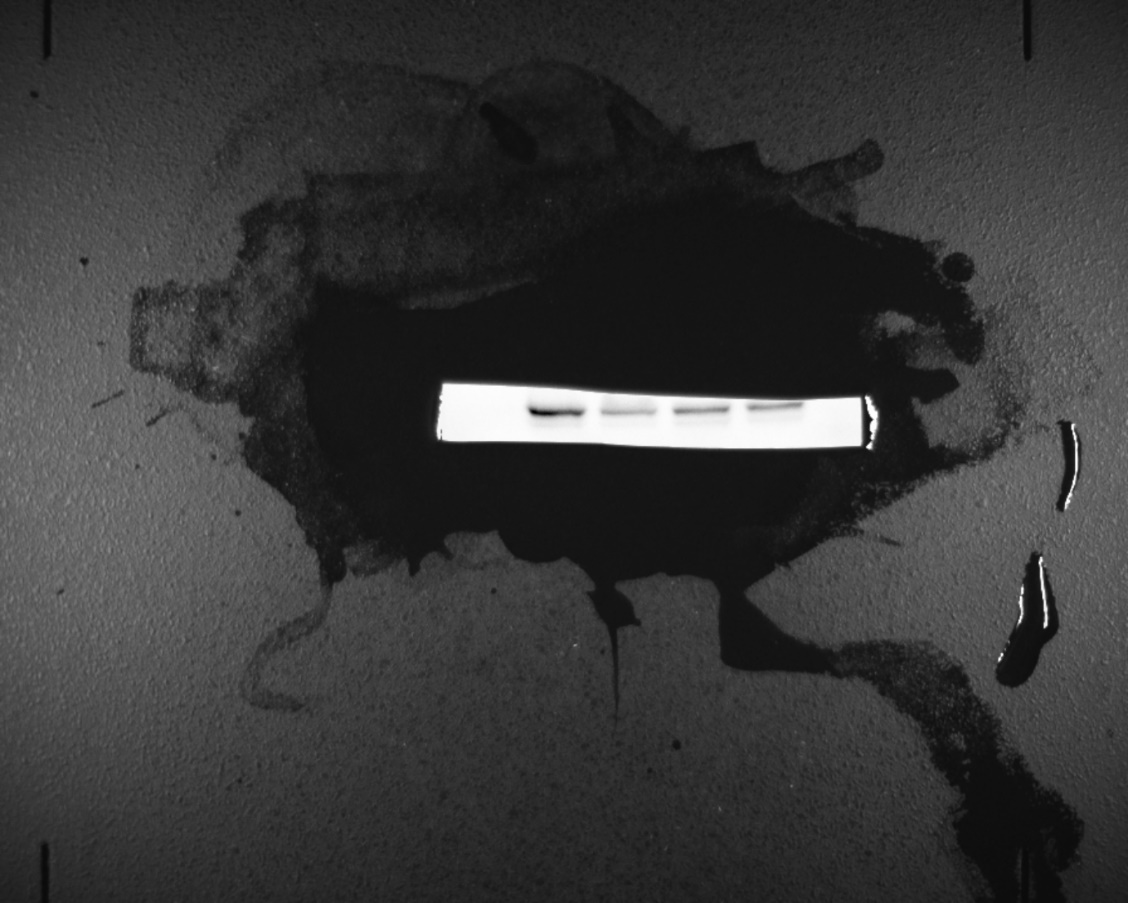


Compound **33** 0.0 10.0 15.0 20.0 μM

p-AKT^T308^

Size 60

KDa


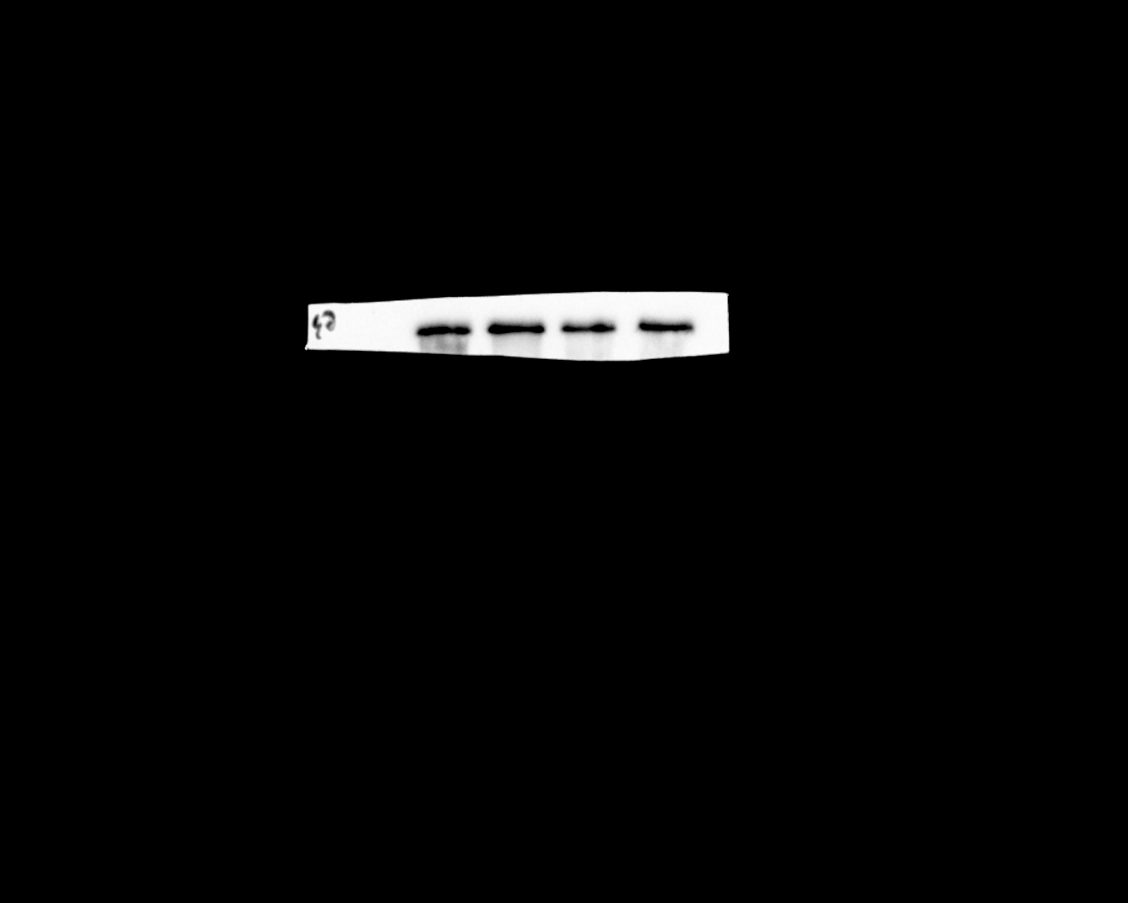


AKT

Compound **33** 0.0 10.0 15.0 20.0 μM

Size 60

KDa


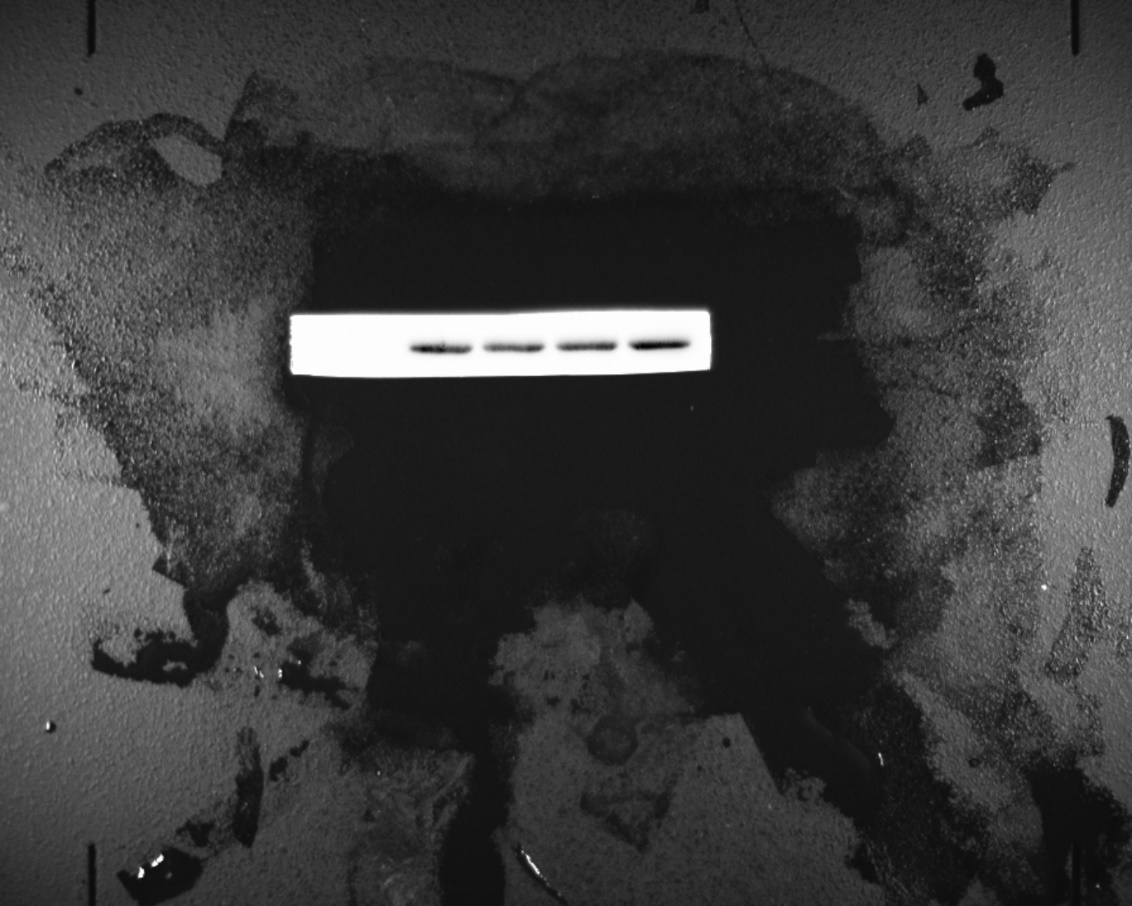


Compound **33** 0.0 10.0 15.0 20.0 μM

β-actin

**supplementary Fig. 17f** All original and uncropped films of Western blots of Total and phosphorylated forms of AKT/STAT proteins.

# supplementary Table 1 Cytotoxicity of all fraction from *A. eriopoda*.*^a^*

| Concentration  Fraction | Inhibitory ratio（%） | | | | | | | |
| --- | --- | --- | --- | --- | --- | --- | --- | --- |
|  | HepG2 | |  | Huh7 | |  | SK-Hep-1 | |
|  | 200 μg/mL | 100 μg/mL |  | 200 μg/mL | 100 μg/mL |  | 200 μg/mL | 100μg/mL |
| 90% EtOH extract | 59.4 | 37.0 |  | 58.9 | 49.6 |  | 53.5 | 39.6 |
| Fr. A | 97.6 | 63.5 |  | 81.7 | 50.8 |  | 79.5 | 32.6 |
| Fr. A-1 | 46.0 | 19.2 |  | 73.5 | 39.1 |  | 77.8 | 35.2 |
| Fr. A-2 | 45.1 | 14.8 |  | 37.7 | 15.7 |  | 70.7 | 33.3 |
| Fr. A-3 | 92.6 | 80.5 |  | 93.8 | 81.8 |  | 94.9 | 86.4 |
| Fr. A-4 | 88.2 | 64.1 |  | 88.4 | 81.7 |  | 92.3 | 66.1 |
| Fr. A-5 | 79.9 | 67.2 |  | 72.9 | 32.7 |  | 88.4 | 35.8 |
| Fr. A-6 | 53.0 | 14.6 |  | 50.0 | 31.3 |  | 49.2 | 38.6 |
| Sorafenib | 100.0 | 97.6 |  | 97.9 | 81.6 |  | 92.5 | 95.3 |

*^a^*Data were expressed as mean ± SD (n = 3)

# supplementary Table 2 ^13^C NMR (*δ* in ppm) data of compounds 1–13 in CDCl_3_.*^a^*

| No. | **1** | **2** | **3** | **4** | **5** | **6** | **7** | **8** | **9** | **10** | **11** | **12** | **13** |
| --- | --- | --- | --- | --- | --- | --- | --- | --- | --- | --- | --- | --- | --- |
| 1 | 43.8 CH | 43.8 CH | 36.7 CH | 42.8 CH | 42.8 CH | 41.3 CH | 43.6 CH | 37.1 CH | 37.0 CH | 44.4 CH | 44.9 CH | 46.8 CH | 52.3 CH |
| 2 | 28.9 CH_2_ | 28.9 CH_2_ | 27.5 CH_2_ | 20.3 CH_2_ | 19.5 CH_2_ | 25.4 CH_2_ | 22.9 CH_2_ | 27.2 CH_2_ | 26.6 CH_2_ | 23.7 CH_2_ | 21.8 CH_2_ | 29.3 CH_2_ | 33.8 CH_2_ |
| 3 | 29.5 CH_2_ | 29.5 CH_2_ | 122.8 CH | 119.0 C | 115.5 C | 26.1 CH_2_ | 37.7 CH_2_ | 41.7 CH_2_ | 41.8 CH_2_ | 38.2 CH_2_ | 37.8 CH_2_ | 58.1 CH | 144.6 C |
| 4 | 146.2 C | 146.3 C | 132.0 C | 196.3 C | 189.1 CH | 136.3 C | 209.3 C | 209.4 C | 209.4 C | 209.0 C | 80.5 C | 210.7 C | 197.6 C |
| 5 | 76.3 CH | 76.3 CH | 75.0 CH | 153.0 CH | 166.4 C | 119.9 CH | 177.8 C | 174.6 C | 164.3 C | 208.3 C | 210.8 C | 78.3 CH | 147.0 CH |
| 6 | 41.0 CH | 41.2 CH | 41.8 CH | 110.2 C | 110.4 C | 38.2 CH | 47.8 CH | 133.1 C | 133.3 C | 58.1 CH | 53.7 CH | 49.5 CH | 54.2 CH |
| 7 | 38.1 CH | 38.1 CH | 38.0 CH | 53.1 CH | 52.9 CH | 54.8 CH | 53.4 CH | 156.4 C | 156.4 C | 43.8 CH | 74.1 C | 41.4 CH | 41.2 CH |
| 8 | 23.7 CH_2_ | 23.8 CH_2_ | 24.0 CH_2_ | 25.8 CH_2_ | 25.8 CH_2_ | 22.7 CH_2_ | 28.2 CH_2_ | 20.4 CH_2_ | 20.0 CH_2_ | 32.5 CH_2_ | 31.0 CH_2_ | 25.8 CH_2_ | 25.7 CH_2_ |
| 9 | 35.3 CH_2_ | 35.2 CH_2_ | 35.3 CH_2_ | 33.2 CH_2_ | 33.1 CH_2_ | 34.2 CH_2_ | 34.5 CH_2_ | 25.2 CH_2_ | 24.4 CH_2_ | 35.0CH_2_ | 30.9 CH_2_ | 34.7 CH_2_ | 35.1 CH_2_ |
| 10 | 27.9 CH | 27.9 CH | 29.3 CH | 33.0 CH | 32.9 CH | 27.5 CH | 34.4 CH | 30.6 CH | 30.3 CH | 33.8 CH | 27.9 CH | 38.1 CH | 36.3 CH |
| 11 | 44.0 C | 44.6 C | 44.8 C | 53.4 C | 53.4 C | 212.2 C | 211.4 C | 57.9 CH | 58.2 CH | 141.8 C | 32.3 CH | 82.4 C | 82.7 C |
| 12 | 178.0 C | 177.8 C | 179.1 C | 176.9 C | 176.7 C |  |  |  |  | 169.8 C | 16.6 CH_3_ | 174.0 C | 173.1 C |
| 13 | 26.3 CH_2_ | 25.9 CH_2_ | 26.2 CH_2_ | 77.5 CH_2_ | 77.5 CH_2_ | 37.9 CH_2_ | 38.3 CH_2_ | 31.2 CH_2_ | 30.0 CH_2_ | 128.2 CH_2_ | 16.0 CH_3_ | 22.7 CH_2_ | 22.9 CH_2_ |
| 14 | 20.1 CH_3_ | 20.0 CH_3_ | 20.2 CH_3_ | 19.0 CH_3_ | 18.9 CH_3_ | 19.7 CH_3_ | 19.5 CH_3_ | 18.6 CH_3_ | 18.6 CH_3_ | 19.6 CH_3_ | 19.0 CH_3_ | 19.6 CH_3_ | 20.0 CH_3_ |
| 15 | 104.9 CH_2_ | 105.0 CH_2_ | 18.3 CH_3_ | 24.8 CH_3_ | 16.2 CH_3_ | 23.6 CH_3_ | 30.0 CH_3_ | 29.9 CH_3_ | 29.9 CH_3_ | 30.0 CH_3_ | 25.4 CH_3_ | 29.0 CH_3_ | 26.4 CH_3_ |
| 1' | 40.5 C | 40.5 C | 40.5 C | 40.1 CH | 39.8 CH | 47.4 CH | 47.2 CH | 47.5 CH | 47.5 CH | 47.5 CH | 30.9 CH | 40.5 CH | 40.5 CH |
| 2' | 77.2 CH | 77.1 CH | 77.2 CH | 130.2 CH | 130.4 CH | 16.7 CH_2_ | 16.7 CH_2_ | 16.7 CH_2_ | 16.7 CH_2_ | 16.7 CH_2_ | 16.2 CH_2_ | 26.6 CH_2_ | 26.9 CH_2_ |
| 3' | 32.2 CH_2_ | 32.1 CH_2_ | 32.2 CH_2_ | 134.9 CH | 134.9 CH | 24.1 CH_2_ | 24.1 CH_2_ | 24.1 CH_2_ | 24.1 CH_2_ | 24.1 CH_2_ | 26.3 CH_2_ | 30.2 CH_2_ | 30.5 CH_2_ |
| 4' | 32.7 CH_2_ | 32.6 CH_2_ | 32.8 CH_2_ | 81.9 C | 81.7 C | 57.2 C | 57.3 C | 57.2 C | 57.2 C | 57.2 C | 60.7 C | 77.5 CH | 77.3 C |
| 5' | 147.1 C | 147.2 C | 147.1 C | 36.4 CH_2_ | 36.2 CH_2_ | 58.9 CH | 58.8 CH | 58.8 CH | 58.7 CH | 58.6 CH | 87.0 C | 97.3 C | 97.6 C |
| 6' | 50.9 CH | 51.1 CH | 50.9 CH | 21.2 CH_2_ | 20.8 CH_2_ | 83.1 C | 83.3 C | 83.3 C | 83.3 C | 83.8 C | 84.5 C | 86.2 C | 85.7 C |
| 7' | 23.1 CH_2_ | 23.6 CH_2_ | 23.1 CH_2_ | 36.5 CH | 36.4 CH | 165.3 C | 165.7 C | 165.0 C | 164.9 C | 168.4 C | 53.5 CH | 38.5 CH | 38.4 CH |
| 8' | 36.1 CH_2_ | 36.8 CH_2_ | 36.1 CH_2_ | 30.9 CH_2_ | 31.0 CH_2_ | 25.9 CH_2_ | 25.9 CH_2_ | 25.8 CH_2_ | 25.7 CH_2_ | 26.4 CH_2_ | 22.1 CH_2_ | 27.6 CH_2_ | 27.2 CH_2_ |
| 9' | 137.6 C | 134.4 C | 137.4 C | 29.3 CH_2_ | 29.3 CH_2_ | 35.3 CH_2_ | 35.2 CH_2_ | 35.5 CH_2_ | 35.5 CH_2_ | 35.1 CH_2_ | 34.3 CH_2_ | 27.4 CH_2_ | 27.0 CH_2_ |
| 10' | 117.0 CH | 120.2 CH | 117.3 CH | 78.5 CH | 78.5 CH | 30.1 CH | 30.1 CH | 30.1 CH | 30.1 CH | 30.2 CH | 46.1 CH | 28.4 CH | 28.2 CH |
| 11' | 35.6 CH_2_ | 38.8 CH_2_ | 36.2 CH_2_ | 73.2 C | 73.2 C | 122.9 C | 122.4 C | 123.2 C | 123.0 C | 117.2 C | 139.4 C | 142.8 C | 142.0 C |
| 12' | 24.5 CH_2_ | 21.5 CH_2_ | 24.7 CH_2_ | 26.7 CH_3_ | 26.7 CH_3_ | 173.2 C | 173.3 C | 173.1 C | 173.0 C | 172.8 C | 170.6 C | 170.9 C | 170.7 C |
| 13' | 26.1 CH_3_ | 26.1 CH_3_ | 26.1 CH_3_ | 23.2 CH_3_ | 23.2 CH_3_ | 17.5 CH_2_ | 17.2 CH_2_ | 18.7 CH_2_ | 18.5 CH_2_ | 36.2 CH_2_ | 114.0 CH_2_ | 118.9 CH_2_ | 119.8 CH_2_ |
| 14' | 15.9 CH_3_ | 16.0 CH_3_ | 15.9 CH_3_ | 15.7 CH_3_ | 15.4 CH_3_ | 17.7 CH_3_ | 17.7 CH_3_ | 17.1 CH_3_ | 17.7 CH_3_ | 17.6 CH_3_ | 18.8 CH_3_ | 20.8 CH_3_ | 20.9 CH_3_ |
| 15' | 108.6 CH_2_ | 108.6 CH_2_ | 108.6 CH_2_ | 51.3 CH_2_ | 51.1 CH_2_ | 22.5 CH_3_ | 22.5 CH_3_ | 22.5 CH_3_ | 22.5 CH_3_ | 22.5 CH_3_ | 23.6 CH_3_ | 27.9 CH_2_ | 27.5 CH_2_ |

^a^ Compounds **1**‒**3** and **6**‒**13** were recorded in 150 MHz; compounds **4** and **5** were recorded in 200 MHz.

# supplementary Table 3 ^1^H NMR (*δ* in ppm, *J* in Hz) data for compounds 1–5 in CDCl_3_.*^a^*

| No. | **1** | **2** | **3** | **4** | **5** |
| --- | --- | --- | --- | --- | --- |
| 1 | 1.47 m | 1.49 m | 2.33 m | 1.43 m | 1.42 m |
| 2a | 2.01 m | 2.01 m | 2.26 m | 2.62 dd (15.2, 4.0) | 2.58 dd (16.8, 4.0) |
| 2b | 1.49 m | 1.48 m | 2.16 m | 1.90 m | 1.86 m |
| 3a | 2.25 m | 2.25 m | 5.37 m |  |  |
| 3b | 2.16 m | 2.17 m |  |  |  |
| 4 |  |  |  |  | 9.83 s |
| 5 | 4.97 d (12.0) | 4.98 d (12.0) | 4.98 d (11.4) | 7.38 d (1.6) |  |
| 6 | 2.19 m | 2.15 m | 1.45 m |  |  |
| 7 | 1.80 m | 1.80 m | 1.81 m | 2.38 dd (12.8, 6.4) | 2.40 dd (12.8, 6.4) |
| 8a | 1.79 m | 1.77 m | 1.81 m | 1.78 m | 1.76 m |
| 8b | 1.44 m | 1.43 m | 1.54 m | 1.62 m | 1.62 m |
| 9a | 1.91 dq (13.2, 3.0) | 1.89 dq (13.2, 3.6) | 1.87 m | 1.73 m | 1.73 m |
| 9b | 1.04 qd (13.2, 3.6) | 1.05 m | 1.03 m | 1.07 m | 1.06 m |
| 10 | 1.76 m | 1.76 m | 1.42 m | 1.43 m | 1.42 m |
| 13a | 2.22 ddd (13.8, 7.8, 5.4) | 2.14 m | 2.25 ddd (13.8, 10.2, 7.8) | 4.56 d (9.6) | 4.56 d (8.8) |
| 13b | 1.83 m | 1.80 m | 1.84 m | 3.88 d (9.6) | 3.86 d (8.8) |
| 14 | 0.91 d (6.0) | 0.90 d (6.6) | 0.87 d (6.0) | 0.97 d (6.4) | 0.96 d (6.4) |
| 15a | 5.04 d (1.2) | 5.07 d (1.2) | 1.79 brs | 2.22 s | 2.16 s |
| 15b | 4.81 d (1.2) | 4.83 d (1.2) |  |  |  |
| 1' |  |  |  | 2.21 m | 2.24 m |
| 2'a | 3.42 dd (9.6, 4.2) | 3.42 dd (9.6, 4.2) | 3.42 dd (9.0, 3.6) | 5.71 d (10.4) | 5.74 d (10.2) |
| 2'b |  |  |  |  |  |
| 3'a | 1.86 dq (12.6, 4.8) | 1.85 dq (12.6, 4.8) | 1.87 m | 5.75 dd (10.4, 2.4) | 5.74 d (10.2) |
| 3'b | 1.53 m | 1.51 m | 1.52 m |  |  |
| 4'a | 2.34 dt (13.8, 4.8) | 2.33 dt (13.2, 4.8) | 2.36 m |  |  |
| 4'b | 1.97 m | 1.98 m | 1.99 m |  |  |
| 5'a |  |  |  | 2.05 m | 2.05 m |
| 5'b |  |  |  | 1.81 m | 1.81 m |
| 6'a | 1.63 dd (10.2, 1.8) | 1.64 t (6.6) | 1.64 m | 1.81 m | 1.81 m |
| 6'b |  |  |  | 1.48 m | 1.46 m |
| 7'a | 1.65 m | 1.57 m | 1.65 m | 1.60 m | 1.60 m |
| 7'b | 1.59 m | 1.57 m | 1.59 m |  |  |
| 8'a | 2.08 m | 2.01 m | 2.29 m | 1.44 m | 1.49 m |
| 8'b | 1.81 m | 1.72 m | 2.08 m | 1.44 m | 1.44 m |
| 9'a |  |  |  | 1.46 m | 1.44 m |
| 9'b |  |  |  | 1.39 m | 1.38 m |
| 10' | 5.21 brd (1.2) | 5.42 brs | 5.23 m | 3.36 brd (9.6) | 3.35 brd (10.4) |
| 11'a | 2.37 m | 2.27 m | 2.35 m |  |  |
| 11'b | 2.26 m | 2.18 m | 1.85 m |  |  |
| 12'a | 1.99 m | 2.12 m | 1.97 m | 1.23 s | 1.23 s |
| 12'b | 1.96 m | 1.96 m | 1.97 m |  |  |
| 13' | 1.03 s | 1.02 s | 1.04 s | 1.18 s | 1.18 s |
| 14' | 0.74 s | 0.72 s | 0.74 s | 0.88 (7.2) | 0.87 d (6.4) |
| 15'a | 4.88 s | 4.87 s | 4.89 s | 3.02 d (13.6) | 3.08 d (13.6) |
| 15'b | 4.59 s | 4.57 s | 4.60 s | 2.25 d (13.6) | 2.45 d (13.6) |

^a^Compounds **1**‒**3** were recorded in 150 MHz; compounds **4** and **5** were recorded in 200 MHz.

# supplementary Table 4 ^1^H NMR (600 MHz, *δ* in ppm, *J* in Hz) data for compounds 6–10 in CDCl_3_.

| No. | **6** | **7** | **8** | **9** | **10** |
| --- | --- | --- | --- | --- | --- |
| 1 | 1.35 m | 1.37 m | 2.14 m | 2.14 m | 1.57 m |
| 2a | 1.94 m | 1.90 m | 1.85 m | 1.80 m | 1.84 m |
| 2b | 1.60 m | 1.61 m | 1.21 m | 1.80 m | 1.52 m |
| 3a | 1.86 m | 2.63 ddd (16.2, 12.0, 4.2) | 2.55 td (9.6, 2.4) | 2.57 m | 2.49 ddd (16.2, 12.0, 4.2) |
| 3b | 1.80 m | 2.31 ddd (16.2, 12.0, 4.8) | 2.55 td (9.6, 2.4) | 2.57 m | 2.39 ddd 16.2, 12.0, 4.2) |
| 5 | 4.74 brs |  |  |  |  |
| 6 | 2.84 m | 2.46 t (11.4) |  |  | 2.80 t (10.8) |
| 7 | 2.41 dt (12.6, 3.6) | 2.77 m |  |  | 2.80 m |
| 8a | 1.55 m | 1.97 m | 1.06 m | 1.05 m | 1.85 m |
| 8b | 1.40 m | 1.17 m | 0.91 m | 0.96 m | 1.49 m |
| 9a | 1.66 dq (13.2, 3.0) | 1.82 m | 1.56 m | 2.00 m | 1.76 m |
| 9b | 0.92 m | 1.17 m | 1.25 m | 1.52 m | 1.21 m |
| 10 | 1.40 m | 1.25 m | 1.80 m | 1.86 m | 1.32 m |
| 11 |  |  | 3.96 m | 4.00 dd (6.0, 3.0) |  |
| 13a | 2.89 dt (18.0, 6.6) | 2.92 dt (18.0, 7.2) | 1.95 m | 1.94 m | 6.29 s |
| 13b | 2.72 dt (18.0, 7.2) | 2.72 dt (18.0, 6.6) | 1.60 m | 1.64 m | 5.65 s |
| 14 | 0.88 d (6.0) | 0.91 d (6.6) | 0.92 d (6.6) | 0.90 d (7.2) | 0.95 d (6.0) |
| 15 | 1.57 s | 2.15 s | 2.16 s | 2.16 s | 2.15 s |
| 1' | 1.11 dt (10.8, 3.6) | 1.34 ddd (15.0, 12.0, 1.8) | 1.15 m | 1.13 m | 1.25 m |
| 2'a | 1.82 m | 1.80 m | 1.85 m | 1.85 m | 1.80 m |
| 2'b | 1.56 m | 1.55 m | 1.58 m | 1.56 m | 1.55 m |
| 3'a | 1.92 m | 1.93 m | 1.94 m | 1.85 m | 1.93 |
| 3'b | 1.73 ddd (18.0, 13.2, 4.8) | 1.73 td (14.4, 4.8) | 1.73 m | 1.73 td (14.4, 4.8) | 1.73 m |
| 5' | 2.68 s | 2.67 s | 2.72 s | 2.75 s | 2.76 s |
| 8'a | 3.06 dt (13.8, 3.0) | 3.00 dt (13.8, 3.0) | 2.80 dt (12.6, 3.6) | 2.76 m | 2.58 dt (13.8, 4.2) |
| 8'b | 2.10 td (13.8, 5.4) | 2.08 td (13.8, 4.8) | 1.38 m | 2.11 m | 2.08 m |
| 9'a | 2.00 m | 1.97 m | 1.98 m | 2.00 m | 1.93 m |
| 9'b | 1.15 qd (13.2, 3.6) | 1.13 m | 1.09 m | 1.05 m | 1.21 m |
| 10' | 1.54 m | 1.51 m | 1.51 m | 1.54 m | 1.55 m |
| 13'a | 2.54 dt (13.8, 6.6) | 2.48 m | 2.30 m | 2.24 m | 3.37 d (17.4) |
| 13'b | 2.50 dt (13.8, 7.2) | 2.48 m | 2.17 m | 2.12 m | 3.25 d (17.4) |
| 14' | 0.94 d (6.6) | 0.93 d (6.6) | 0.95 d (6.6) | 0.94 d (6.6) | 0.93 d (6.6) |
| 15' | 1.37 s | 1.37 s | 1.38 s | 1.38 s | 1.37 s |

# supplementary Table 5 ^1^H NMR (600 MHz, *δ* in ppm, *J* in Hz) data for compounds 11–15.

| No. | **11***^a^* | **12***^a^* | **13***^a^* | **14***^a^* | **15***^b^* |
| --- | --- | --- | --- | --- | --- |
| 1 | 2.20 m | 1.07 m | 2.16 m | 0.96 m | 0.99 m |
| 2a | 1.83 dt (15.0, 4.8) | 1.94 m | 2.64 dd (15.0, 7.2) | 1.81 ddd (12.6, 6.6, 3.0) | 2.09 ddd (12.6, 7.2, 3.6) |
| 2b | 1.58 m | 1.62 m | 1.92 m | 1.64 m | 1.44 q (12.0) |
| 3a | 1.66 m | 2.74 m |  | 2.99 ddd (12.0, 6.0, 3.0) | 2.83 ddd (11.4, 7.8, 3.6) |
| 3b | 1.60 m |  |  |  |  |
| 5 |  | 4.37 ddd (11.4, 7.8, 4.2) | 6.66 s | 4.23 dd (9.0, 6.6) | 3.98 t (7.8) |
| 6 | 3.09 dd (5.4, 1.8) | 1.45 q (10.8) | 1.44 m | 1.34 q (10.2) | 1.67 q (10.8) |
| 7 |  | 2.14 ddd (13.2, 10.2, 3.6) | 2.15 m | 1.68 m | 1.65 m |
| 8a | 1.90 m | 1.63 m | 1.93 m | 2.67 m | 2.68 m |
| 8b | 1.66 m | 0.93 m | 1.41 m | 0.92 m | 0.97 m |
| 9a | 1.61 m | 1.76 m | 1.83 dq (13.2, 3.0) | 1.72 m | 1.75 m |
| 9b | 1.29 m | 0.91 m | 0.98 m | 0.93 m | 0.94 m |
| 10 | 1.10 m | 1.03 m | 1.33 m | 1.17 m | 1.18 m |
| 11 | 2.02 m |  |  |  |  |
| 12 | 0.86 d (6.6) |  |  |  |  |
| 13a | 0.98 d (6.6) | 1.88 m | 2.21 ddd (13.8, 9.6, 6.6) | 2.05 td (13.2, 6.0) | 1.83 ddd (11.4, 7.8, 3.6) |
| 13b |  | 1.30 m | 1.54 m | 1.68 m | 1.73 m |
| 14 | 0.76 d (6.0) | 0.85 d (6.0) | 0.90 d (6.6) | 0.84 d (6.6) | 0.87 d (6.6) |
| 15a | 1.97 ddd (13.8, 7.8, 0.6) | 2.17 s | 2.24 s | 2.28 s | 2.26 s |
| 15b | 1.10 td (13.8, 7.2) |  |  |  |  |
| 1' | 1.45 m | 1.65 m | 1.75 ddd (12.6, 8.4, 4.2) | 1.05 m | 1.10 m |
| 2'a | 1.70 m | 2.13 m | 1.97 dq (15.0, 4.2) | 1.96 dq (13.2, 3.0) | 2.00 m |
| 2'b | 1.45 m | 1.47 m | 1.45 m | 0.94 m | 1.73 m |
| 3'a | 1.95 m | 2.10 td (12.6, 4.8) | 2.09 td (13.2, 4.8) | 1.58 m | 2.03 m |
| 3'b | 1.55 m | 1.81 dt (12.6, 3.6) | 1.91 m | 1.12 td (12.6, 3.0) | 1.00 m |
| 4' |  |  |  | 1.61 m | 1.73 m |
| 6' |  |  |  | 2.31 t (10.2) | 1.34 m |
| 7' | 2.78 dq (12.0, 3.6) | 3.55 t (6.0) | 3.47 td (4.2, 1.2) | 2.51 brt (10.8) | 2.68 m |
| 8'a | 2.08 m | 2.47 m | 1.71 dq (12.6, 2.4) | 1.96 dq (13.2, 3.0) | 1.66 m |
| 8'b | 1.88 m | 1.68 m | 1.07 m | 1.55 m | 1.66 m |
| 9'a | 1.92 m | 1.56 m | 1.56 m | 1.70 m | 1.69 m |
| 9'b | 1.32 m | 1.13 m | 1.14 m | 1.10 m | 1.07 m |
| 10' | 1.58 m | 1.48 m | 1.50 m | 1.25 m | 1.26 m |
| 13'a | 6.15 d (3.6) | 6.13 d (1.8) | 6.16 d (2.4) | 6.07 brs | 6.14 s |
| 13'b | 5.35 d (3.6) | 5.54 d (1.2) | 5.55 d (2.4) | 5.58 d (1.2) | 5.57 s |
| 14' | 1.00 d (6.6) | 0.98 d (6.6) | 1.00 d (6.6) | 0.91 d (6.6) | 0.96 d (6.6) |
| 15'a | 2.37 ddd (15.0, 12.6, 7.2) | 1.92 m | 2.81 ddd (14.4, 10.8, 2.4) | 1.79 m | 1.86 dt (14.4, 5.4) |
| 15'b | 1.74 ddd (15.0, 7.8, 1.2) | 1.40 m | 1.77 ddd (14.4, 11.4, 6.0) | 1.41 m | 1.33 m |
| 5-OH |  | 3.04 d (4.2) |  |  |  |
| 5'-OH |  | 5.18 s |  |  |  |

*^a^*Recorded in CDCl_3_; *^b^*recorded in CD_3_OD.

# supplementary Table 6 ^13^C NMR (150MHz, *δ* in ppm) data and the types of carbon for compounds 14–26.

| No. | **14***^a^* | **15***^b^* | **16***^a^* | **17***^a^* | **18***^a^* | **19***^b^* | **20***^a^* | **21***^b^* | **22***^a^* | **23***^b^* | **24***^b^* | **25***^b^* | **26***^a^* |
| --- | --- | --- | --- | --- | --- | --- | --- | --- | --- | --- | --- | --- | --- |
| 1 | 48.6 CH | 49.5 CH | 55.0 CH | 54.9 CH | 54.9 CH | 49.6 CH | 49.6 CH | 55.9 CH | 54.9 CH | 56.8 CH | 49.8 CH | 54.3 CH | 52.8 CH |
| 2 | 31.1 CH_2_ | 30.2 CH_2_ | 33.4 CH_2_ | 33.4 CH_2_ | 33.4 CH_2_ | 39.5 CH_2_ | 40.1 CH_2_ | 34.4 CH_2_ | 33.4 CH_2_ | 34.4 CH_2_ | 31.4 CH_2_ | 43.3 CH_2_ | 42.5 CH_2_ |
| 3 | 56.5 CH | 57.8 CH | 146.3 C | 146.3 C | 146.2 C | 210.8 C | 210.2 C | 200.9 C | 146.0 C | 145.9 C | 58.1 CH | 216.1 C | 213.9 C |
| 4 | 213.4 C | 212.2 C | 197.0 C | 197.5 C | 196.9 C |  |  | 145.5 C | 198.3 C | 200.6 C | 212.7 C |  |  |
| 5 | 75.6 CH | 78.9 CH | 146.1 CH | 145.5 CH | 144.7 CH | 126.6 CH | 127.4 CH | 152.3 CH | 146.6 CH | 151.0 CH | 79.5 CH | 46.0 CH_2_ | 44.6 CH_2_ |
| 6 | 53.2 CH | 54.4 CH | 52.0 CH | 51.9 CH | 51.2 CH | 182.3 C | 181.2 C | 54.2 CH | 52.2 CH | 54.4 CH | 54.2 CH | 92.2 C | 90.0 C |
| 7 | 45.1 CH | 46.4 CH | 44.0 CH | 43.6 CH | 43.2 CH | 44.6 CH | 44.4 CH | 44.8 CH | 46.5 CH | 42.0 CH | 51.3 CH | 165.2 C | 162.2 C |
| 8 | 25.8 CH_2_ | 27.2 CH_2_ | 27.0 CH_2_ | 26.6 CH_2_ | 27.0 CH_2_ | 26.8 CH_2_ | 27.1 CH_2_ | 27.7 CH_2_ | 27.5 CH_2_ | 32.3 CH_2_ | 29.1 CH_2_ | 25.4 CH_2_ | 24.5 CH_2_ |
| 9 | 34.5 CH_2_ | 35.7 CH_2_ | 34.8 CH_2_ | 34.6 CH_2_ | 34.8 CH_2_ | 33.0 CH_2_ | 33.4 CH_2_ | 36.2 CH_2_ | 34.9 CH_2_ | 36.9 CH_2_ | 36.3 CH_2_ | 36.5 CH_2_ | 35.4 CH_2_ |
| 10 | 37.9 CH | 39.5 CH | 35.9 CH | 35.9 CH | 35.6 CH | 40.8 CH | 40.9 CH | 37.7 CH | 35.9 CH | 37.4 CH | 39.2 CH | 36.0 CH | 34.8 CH |
| 11 | 82.6 C | 84.7 C | 84.2 C | 85.2 C | 85.4 C | 80.8 C | 80.9 C | 84.5 C | 82.6 C | 46.4 CH | 81.7 C | 126.2 C | 125.5 C |
| 12 | 173.7 C | 175.0 C | 174.4 C | 173.0 C | 173.5 C | 174.4 C | 174.6 C | 175.5 C | 176.8 C | 175.6 C | 176.3 C | 175.0 C | 172.8 C |
| 13 | 23.8 CH_2_ | 25.3 CH_2_ | 23.8 CH_2_ | 22.6 CH_2_ | 23.9 CH_2_ | 23.1 CH_2_ | 23.3 CH_2_ | 22.4 CH_2_ | 23.6 CH_2_ | 19.3 CH_2_ | 31.3 CH_2_ | 22.3 CH_2_ | 21.4 CH_2_ |
| 14 | 19.6 CH_3_ | 20.2 CH_3_ | 19.9 CH_3_ | 19.9 CH_3_ | 19.9 CH_3_ | 19.5 CH_3_ | 20.8 CH_3_ | 20.6 CH_3_ | 19.9 CH_3_ | 20.6 CH_3_ | 20.2 CH_3_ | 19.4 CH_3_ | 19.0 CH_3_ |
| 15 | 30.2 CH_3_ | 29.4 CH_3_ | 25.9 CH_3_ | 26.2 CH_3_ | 26.4 CH_3_ |  |  | 26.7 CH_3_ | 26.4 CH_3_ | 26.5 CH_3_ | 29.6 CH_3_ |  |  |
| 1' | 46.7 CH | 47.8 CH | 39.6 CH | 42.0 CH | 44.9 CH | 46.2 CH | 46.2 CH | 47.5 CH | 43.3 CH | 52.8 CH | 54.4 CH | 54.4 CH | 52.6 CH |
| 2' | 28.5 CH_2_ | 28.6 CH_2_ | 32.2 CH_2_ | 20.8 CH_2_ | 18.6 CH_2_ | 28.4 CH_2_ | 28.7 CH_2_ | 25.3 CH_2_ | 22.7 CH_2_ | 27.5 CH_2_ | 31.2 CH_2_ | 31.2 CH_2_ | 29.9 CH_2_ |
| 3' | 29.2 CH_2_ | 30.1 CH_2_ | 24.5 CH_2_ | 35.2 CH_2_ | 36.0 CH_2_ | 29.0 CH_2_ | 29.2 CH_2_ | 35.8 CH_2_ | 29.2 CH_2_ | 40.8 CH_2_ | 35.9 CH_2_ | 36.8 CH_2_ | 34.9 CH_2_ |
| 4' | 40.7 CH | 41.6 CH | 39.7 CH | 69.4 C | 69.9 C | 39.3 CH | 39.3 CH | 68.9 C | 110.6 C | 90.6 C | 52.7 CH | 51.9 CH | 50.7 CH |
| 5' | 110.4 C | 112.2 C | 113.1 C | 105.6 C | 107.2 C | 111.5 C | 111.7 C | 112.4 C | 144.9 C | 210.1 C | 215.2 C | 214.4 C | 213.0 C |
| 6' | 47.6 CH | 46.3 CH | 47.6 C | 85.8 C | 85.7 C | 48.0 C | 47.4 C | 46.0 CH | 84.3 C | 56.3 CH | 60.8 CH | 60.8 CH | 60.2 CH |
| 7' | 45.1 CH | 45.6 CH | 75.3 C | 37.8 CH | 44.5 CH | 45.0 CH | 45.5 CH | 37.7 CH | 39.8 CH | 39.6 CH | 39.1 CH | 39.0 CH | 37.5 CH |
| 8' | 32.6 CH_2_ | 31.2 CH_2_ | 27.6 CH_2_ | 26.9 CH_2_ | 23.9 CH_2_ | 33.3 CH_2_ | 33.0 CH_2_ | 36.7 CH_2_ | 27.3 CH_2_ | 35.0 CH_2_ | 34.7 CH_2_ | 34.9 CH_2_ | 33.4 CH_2_ |
| 9' | 35.4 CH_2_ | 36.4 CH_2_ | 30.7 CH_2_ | 26.2 CH_2_ | 31.6 CH_2_ | 35.1 CH_2_ | 35.6 CH_2_ | 36.8 CH_2_ | 24.4 CH_2_ | 36.0 CH_2_ | 36.3 CH_2_ | 36.3 CH_2_ | 34.8 CH_2_ |
| 10' | 37.5 CH | 38.4 CH | 28.0 CH | 27.9 CH | 30.2 CH | 37.2 CH | 37.7 CH | 38.7 CH | 27.2 CH | 39.1 CH | 39.6 CH | 39.5 CH | 38.2 CH |
| 11' | 144.8 C | 149.1 C | 34.8 CH | 140.0 C | 211.4 C | 146.4 C | 145.6 C | 145.5 C | 143.8 C | 148.1 C | 148.3 C | 147.1 C | 145.3 C |
| 12' | 167.2 C | 170.6 C | 17.1 CH_3_ | 168.4 C |  | 168.6 C | 170.1 C | 170.3 C | 170.8 C | 170.8 C | 170.1 C | 171.4 C | 171.3 C |
| 13' | 125.6 CH_2_ | 124.2 CH_2_ | 16.8 CH_3_ | 121.5 CH_2_ | 69.4 CH_2_ | 122.4 CH_2_ | 124.6 CH_2_ | 124.3 CH_2_ | 119.7 CH_2_ | 122.3 CH_2_ | 121.9 CH_2_ | 122.3 CH_2_ | 123.6 CH_2_ |
| 14' | 20.2 CH_3_ | 20.8 CH_3_ | 20.2 CH_3_ | 20.8 CH_3_ | 19.8 CH_3_ | 18.9 CH_3_ | 20.1 CH_3_ | 20.7 CH_3_ | 20.7 CH_3_ | 20.3 CH_3_ | 20.5 CH_3_ | 20.5 CH_3_ | 20.0 CH_3_ |
| 15' | 24.4 CH_2_ | 26.0 CH_2_ | 22.8 CH_2_ | 31.9 CH_2_ | 31.6 CH_2_ | 24.2 CH_2_ | 24.5 CH_2_ | 33.2 CH_2_ | 24.8 CH_2_ | 31.5 CH_2_ | 24.6 CH_2_ | 29.7 CH_2_ | 28.6 CH_2_ |
| 1'' |  |  |  |  |  |  |  |  |  |  | 62.4 CH_2_ |  |  |
| 2'' |  |  |  |  |  |  |  |  |  |  | 14.7 CH_3_ |  |  |

*^a^*Recorded in CDCl_3_; *^b^*recorded in CD_3_OD.

# supplementary Table 7 ^1^H NMR data (600 MHz, *J* in Hz, *δ* in ppm) of compounds 16–21.

| No. | **16***^a^* | **17***^a^* | **18***^a^* | **19***^b^* | **20***^a^* | **21***^b^* |
| --- | --- | --- | --- | --- | --- | --- |
| 1 | 1.42 m | 1.31 m | 1.37 m | 2.37 m | 2.33 m | 1.40 m |
| 2a | 2.67 dd (15.0, 6.6) | 2.67 dd (15.6, 7.2) | 2.67 dd (15.0, 6.6) | 2.57 dd (192, 6.6) | 2.57 dd (18.6, 6.6) | 2.60 dd (14.4, 6.6) |
| 2b | 2.00 m | 1.90 dd (15.0, 4.2) | 2.04 dq (13.2, 2.4) | 2.00 m | 1.93 dd (18.6, 1.2) | 1.91 m |
| 5 | 7.02 m | 6.78 s | 6.68 t (1.8) | 6.04 s | 6.22 s | 7.24 s |
| 6 | 2.33 brt (10.8) | 2.23 m | 2.20 td (11.4, 2.4) |  |  | 2.30 brt (10.2) |
| 7 | 1.85 m | 1.83 m | 1.84 m | 2.81 dd (12.0, 4.8) | 2.76 dd (12.6, 3.6) | 1.88 td (12.0, 3.0) |
| 8a | 2.41 m | 2.28 m | 2.10dq (13.2, 3.0) | 2.57 m | 2.63 m | 2.50 m |
| 8b | 1.21 m | 1.20 m | 1.22 m | 1.31 m | 1.21 m | 1.13 m |
| 9a | 1.85 m | 1.80 m | 1.82 m | 1.87 m | 1.83 m | 1.80 m |
| 9b | 1.06 m | 0.99 m | 1.02 m | 1.29 m | 1.29 m | 1.03 m |
| 10 | 1.48 m | 1.43 m | 1.48 m | 1.29 m | 1.26 m | 1.48 m |
| 13a | 2.13 m | 2.20 td (13.2, 6.0) | 2.28 m | 2.02 m | 2.02 td (13.8, 6.0) | 2.04 td (12.6, 6.0) |
| 13b | 1.57 m | 1.61 m | 1.53 m | 1.73 m | 1.71 dd (13.8, 6.0) | 1.62 m |
| 14 | 0.93 d (6.6) | 0.91 d (6.0) | 0.91 d (6.6) | 1.04 d (6.0) | 1.03 d (6.0) | 0.94 d (6.6) |
| 15 | 2.28 s | 2.33 s | 2.29 s |  |  | 2.38 s |
| 1' | 2.10 m | 1.56 m | 1.56 m | 1.07 m | 1.09 m | 1.10 m |
| 2'a | 1.72 td (13.8, 4.8) | 1.82 m | 1.80 m | 2.04 m | 1.99 m | 1.78 m |
| 2'b | 1.65 m | 1.68 m | 1.72 m | 1.02 m | 1.00 m | 1.44 m |
| 3'a | 1.83 m | 1.84 m | 1.82 m | 1.65 m | 1.59 m | 1.62 m |
| 3'b | 1.39 m | 1.60 m | 1.60 td (13.2, 4.2) | 1.12 m | 1.15 m | 1.03 m |
| 4' | 1.76 m |  |  | 1.80 m | 1.85 m |  |
| 6' | 2.22 dd (4.2, 1.2) |  |  | 2.14 m | 2.18 m | 2.48 m |
| 7' |  | 3.13 m | 2.32 dd (10.2, 7.2) | 2.56 m | 2.50 brt (10.2) | 1.48 m |
| 8'a | 2.05 dq (13.2, 2.4) | 1.94 m | 1.89 m | 1.88 m | 2.00 m | 1.73 m |
| 8'b | 1.32 m | 1.45 m | 1.35 m | 1.53 m | 1.61 m | 1.46 m |
| 9'a | 1.58 m | 1.57 m | 1.70 m | 1.71 m | 1.73 dq (13.2, 3.0) | 1.71 m |
| 9'b | 1.36 m | 1.17 m | 1.14 m | 1.14 m | 1.12 m | 1.13 m |
| 10' | 1.94 m | 1.58 m | 1.58 m | 1.25 m | 1.24 m | 1.29 m |
| 11' | 1.87 m |  |  |  |  |  |
| 12' | 1.02 d (6.0) |  |  |  |  |  |
| 13'a | 0.83 d (6.6) | 6.17 d (1.2) | 4.19 d (2.4) | 5.86 s | 5.92 brs | 5.96 s |
| 13'b |  | 5.43 s | 4.19 d (2.4) | 5.39 s | 5.48 d (1.8) | 5.48 s |
| 14' | 0.88 d (6.6) | 1.02 d (6.0) | 0.99 d (6.6) | 0.95 d (6.6) | 0.92 d (6.6) | 0.96 d (6.0) |
| 15'a | 1.88 m | 1.87 dt (15.6, 2.4) | 1.86 m | 1.90 dt (14.4, 5.4) | 1.86 m | 1.69 m |
| 15'b | 1.60 m | 1.70 m | 1.68 m | 1.37 m | 1.43 m | 1.69 m |
| OH |  |  | 4.47 d (2.4) |  |  |  |

*^a^*Recorded in CDCl_3_; *^b^*recorded in CD_3_OD.

# supplementary Table 8 ^1^H NMR data (600 MHz, *J* in Hz, *δ* in ppm) of compounds 22–26.

| No. | **22***^a^* | **23***^b^* | **24***^b^* | **25***^b^* | **26***^a^* |
| --- | --- | --- | --- | --- | --- |
| 1 | 1.29 m | 1.33 m | 0.97 m | 1.92 m | 1.90 m |
| 2a | 2.64 dd (15.0, 6.0) | 2.60 dd (15.0, 7.2) | 2.05 ddd (13.2, 7.2, 3.6) | 2.72 dd (19.2, 7.8) | 2.81 dd (19.2, 7.8) |
| 2b | 1.88 m | 1.90 m | 1.55 q (12.0) | 2.39 brd (19.2) | 2.38 brd (19.2) |
| 3 |  |  | 2.92 ddd (10.8, 6.6, 3.0) |  |  |
| 5a | 6.87 s | 7.30 s | 4.00 dd (9.0, 6.6) | 2.97 d (19.8) | 2.67 d (19.2) |
| 5b |  |  |  | 2.17 d (19.8) | 2.27 d (19.2) |
| 6 | 2.25 brt (10.2) | 2.06 brt (10.2) | 1.42 q (9.6) |  |  |
| 7 | 1.90 m | 2.27 m | 1.74 m |  |  |
| 8a | 1.68 m | 1.59 m | 1.60 m | 2.85 ddd (14.4, 4.2, 3.0) | 2.87 dt (13.8, 3.0) |
| 8b | 1.18 m | 1.38 m | 1.23 m | 2.37 td (13.8, 4.8) | 2.22 m |
| 9a | 1.79 m | 1.83 m | 1.71 dq (13.8, 3.0) | 2.05 m | 2.04 m |
| 9b | 0.96 m | 1.09 m | 0.93 m | 1.29 m | 1.26 m |
| 10 | 1.41 m | 1.45 m | 1.16 m | 1.46 m | 1.30 m |
| 11 |  | 2.57 ddd (12.0, 6.0, 1.2) |  |  |  |
| 13a | 2.11 m | 1.81 m | 1.78 m | 2.23 t (7.8) | 2.22 m |
| 13b | 1.91 m | 1.14 m | 1.63 m | 2.23 t (7.8) | 2.22 m |
| 14 | 0.89 d (5.4) | 0.94 d (6.6) | 0.89 d (6.6) | 0.99 d (6.6) | 0.98 d (6.0) |
| 15 | 2.34 s | 2.38 s | 2.24 s |  |  |
| 1' | 1.43 m | 1.20 m | 1.12 m | 1.10 m | 1.14 m |
| 2'a | 1.79 m | 2.19 m | 2.18 dq (13.8, 3.0) | 2.14 m | 2.13 m |
| 2'b | 1.62 m | 1.66 m | 1.50 m | 1.50 m | 1.42 m |
| 3'a | 2.15 m | 2.31 dt (13.2, 2.4) | 2.28 m | 2.20 m | 2.17 m |
| 3'b | 1.93 m | 1.92 m | 1.24 m | 1.31 m | 1.28 m |
| 4' |  |  | 2.41 td (12.0, 6.0) | 2.47 m | 2.42 m |
| 6' |  | 3.06 t (11.4) | 2.65 t (11.4) | 2.61 t (11.4) | 2.47 t (10.8) |
| 7' | 3.50 m | 2.81 td (11.4, 3.6) | 2.79 td (12.0, 3.0) | 2.78 td (11.4, 3.0) | 2.76 m |
| 8'a | 1.93 m | 1.85 m | 1.88 dq (13.2, 3.0) | 1.86 m | 1.88 m |
| 8'b | 1.38 m | 1.29 m | 1.20 m | 1.18 m | 1.22 m |
| 9'a | 1.59 m | 1.72 dq (13.8, 3.0) | 1.71 dq (13.8, 3.0) | 1.68 dq (13.2, 3.6) | 1.70 dq (14.4, 2.4) |
| 9'b | 1.13 m | 1.12 m | 1.09 m | 1.07 m | 1.07 m |
| 10' | 1.64 m | 1.48 m | 1.40 m | 1.40 m | 1.37 m |
| 13'a | 6.09 s | 5.94 s | 6.01 s | 6.02 s | 6.15 s |
| 13'b | 5.43 s | 5.45 s | 5.28 s | 5.39 s | 5.45 s |
| 14' | 1.00 d (6.0) | 1.02 d (6.6) | 0.99 d (6.6) | 0.97 d (6.6) | 0.95 d (6.6) |
| 15'a | 2.08 m | 2.93 dt (14.4, 3.0) | 1.81 m | 1.85 m | 1.86 m |
| 15'b | 1.88 m | 1.95 m | 1.03 m | 1.27 m | 1.33 m |
| 1'' |  |  | 4.21 m |  |  |
| 2'' |  |  | 1.31 t (7.2) |  |  |

*^a^*Recorded in CDCl_3_; *^b^*recorded in CD_3_OD.

# supplementary Table 9 ^13^C NMR (150 MHz, *δ* in ppm) and the types of carbon data for compounds 27–36.

| No. | **27***^b^* | **28***^b^* | **29***^b^* | **30***^a^* | **31***^a^* | **32***^a^* | **33***^b^* | **34***^a^* | **35***^b^* | **36***^a^* |
| --- | --- | --- | --- | --- | --- | --- | --- | --- | --- | --- |
| 1 | 49.8 CH | 50.0 CH | 49.8 CH | 55.2 CH | 55.2 CH | 48.3 CH | 47.6 CH | 44.3 CH | 46.0 CH | 48.0 CH |
| 2 | 30.3 CH_2_ | 30.8 CH_2_ | 30.3 CH_2_ | 33.9 CH_2_ | 33.8 CH_2_ | 15.1 CH_2_ | 41.5 CH_2_ | 23.9 CH_2_ | 25.1 CH_2_ | 29.2 CH_2_ |
| 3 | 59.2 CH | 55.9 CH | 59.2 CH | 145.6 C | 145.5 C | 24.7 CH_2_ | 26.2 CH_2_ | 38.2 CH_2_ | 37.9 CH_2_ | 57.8 CH |
| 4 | 213.0 C | 212.8 C | 213.0 C | 197.7 C | 197.8 C | 60.6 C | 205.2 C | 209.6 C | 211.6 C | 210.0 C |
| 5 | 80.9 CH | 77.9 CH | 80.9 CH | 147.7 CH | 148.0 CH | 60.4 CH | 153.7 CH | 177.2 C | 174.2 CH | 79.3 CH |
| 6 | 56.6 CH | 56.9 CH | 56.5 CH | 55.8 CH | 56.0 CH | 69.3 C | 47.1 CH | 52.6 CH | 53.1 CH | 56.1 CH |
| 7 | 44.3 CH | 44.2 CH | 44.6 CH | 41.6 CH | 41.5 CH | 46.4 CH | 46.1 CH | 42.3 CH | 43.2 CH | 44.8 CH |
| 8 | 36.0 CH_2_ | 36.5 CH_2_ | 36.0 CH_2_ | 34.8 CH_2_ | 34.8 CH_2_ | 27.5 CH_2_ | 35.1 CH_2_ | 32.8 CH_2_ | 35.1 CH_2_ | 33.0 CH_2_ |
| 9 | 36.8 CH_2_ | 36.9 CH_2_ | 36.8 CH_2_ | 35.8 CH_2_ | 35.8 CH_2_ | 34.5 CH_2_ | 36.7 CH_2_ | 35.2 CH_2_ | 36.3 CH_2_ | 35.1 CH_2_ |
| 10 | 39.1 CH | 38.9 CH | 39.1 CH | 36.0 CH | 36.0 CH | 30.9 CH | 37.6 CH | 33.5 CH | 34.2 CH | 37.6 CH |
| 11 | 145.8 C | 145.7 C | 145.8 C | 144.5 C | 144.6 C | 143.2 C | 146.8 C | 143.7 C | 146.0 C | 144.4 C |
| 12 | 168.7 C | 169.0 C | 169.0 C | 167.6 C | 167.1 C | 168.7 C | 168.5 C | 166.6 C | 170.1 C | 167.8 C |
| 13 | 125.6 CH_2_ | 125.9 CH_2_ | 125.6 CH_2_ | 124.4 CH_2_ | 124.3 CH_2_ | 124.4 CH_2_ | 126.2 CH_2_ | 125.5 CH_2_ | 125.5 CH_2_ | 124.3 CH_2_ |
| 14 | 20.2 CH_3_ | 20.4 CH_3_ | 20.2 CH_3_ | 20.1 CH_3_ | 20.1 CH_3_ | 19.1 CH_3_ | 21.4 CH_3_ | 20.3 CH_3_ | 20.2 CH_3_ | 19.7 CH_3_ |
| 15 | 29.6 CH_3_ | 31.9 CH_3_ | 29.6 CH_3_ | 26.2 CH_3_ | 26.2 CH_3_ | 23.4 CH_3_ | 131.2 CH | 29.9 CH_3_ | 30.0 CH_3_ | 29.3 CH_3_ |
| 1' | 41.1 CH | 41.0 CH | 43.1 CH | 41.3 CH | 39.5 CH | 39.6 CH | 41.1 CH | 39.2 CH | 36.0 CH_2_ | 43.6 CH |
| 2' | 22.3 CH_2_ | 22.2 CH_2_ | 21.9 CH_2_ | 20.7 CH_2_ | 20.8 CH_2_ | 20.6 CH_2_ | 22.3 CH_2_ | 20.6 CH_2_ | 23.3 CH_2_ | 31.5 CH_2_ |
| 3' | 34.9 CH_2_ | 34.9 CH_2_ | 34.9 CH_2_ | 33.3 CH_2_ | 33.6 CH_2_ | 32.2 CH_2_ | 34.9 CH_2_ | 33.5 CH_2_ | 33.3 CH_2_ | 142.9 C |
| 4' | 84.1 C | 84.1 C | 84.3 C | 82.7 C | 82.0 C | 82.5 C | 84.5 C | 82.6 C | 148.6 C | 121.9 CH |
| 5' | 70.8 CH | 70.9 CH | 67.3 CH | 67.9 CH | 70.6 CH | 72.4 CH | 70.7 CH | 71.0 CH | 89.0 CH | 31.3 CH_2_ |
| 6' | 88.9 C | 89.0 C | 89.2 C | 86.0 C | 85.7 C | 86.2 C | 88.3 C | 87.0 C | 31.2 CH_2_ | 40.7 CH |
| 7' | 43.4 CH | 43.3 CH | 40.9 CH | 39.4 CH | 41.8 CH | 41.7 CH | 43.4 CH | 41.6 CH | 41.0 CH | 38.1 C |
| 8' | 29.8 CH_2_ | 29.8 CH_2_ | 25.2 CH_2_ | 24.0 CH_2_ | 28.4 CH_2_ | 27.9 CH_2_ | 29.8 CH_2_ | 27.6 CH_2_ | 27.0 CH_2_ | 26.2 CH_3_ |
| 9' | 31.3 CH_2_ | 31.2 CH_2_ | 33.7 CH_2_ | 32.3 CH_2_ | 29.8 CH_2_ | 29.4 CH_2_ | 31.4 CH_2_ | 28.9 CH_2_ | 35.7 CH_2_ | 21.1 CH_3_ |
| 10' | 30.2 CH | 30.1 CH | 31.5 CH | 30.2 CH | 28.4 CH | 28.0 CH | 30.2 CH | 27.6 CH | 40.3 C | 67.5 CH_2_ |
| 11' | 143.7 C | 143.7 C | 40.1 CH | 38.6 CH | 141.3 C | 141.3 C | 143.9 C | 140.9 C | 150.8 C |  |
| 12' | 172.7 C | 172.8 C | 182.0 C | 178.4 C | 169.6 C | 170.4 C | 172.0 C | 171.0 C | 21.3 CH_3_ |  |
| 13' | 122.1 CH_2_ | 122.1 CH_2_ | 9.6 CH_3_ | 9.2 CH_3_ | 121.0 CH_2_ | 121.1 CH_2_ | 121.5 CH_2_ | 122.0 CH_2_ | 109.8 CH_2_ |  |
| 14' | 20.7 CH_3_ | 20.7 CH_3_ | 20.5 CH_3_ | 19.9 CH_3_ | 20.2 CH_3_ | 20.2 CH_3_ | 20.7 CH_3_ | 19.6 CH_3_ | 20.2 CH_3_ |  |
| 15' | 23.7 CH_3_ | 23.8 CH_3_ | 24.0 CH_3_ | 23.2 CH_3_ | 23.3 CH_3_ | 22.6 CH_3_ | 23.9 CH_3_ | 23.2 CH_3_ | 110.7 CH_2_ |  |

*^a^*Recorded in CDCl_3_; *^b^*recorded in CD_3_OD.

# supplementary Table 10 ^1^H NMR (600 MHz, *δ* in ppm, *J* in Hz) data for compounds 27–31.

| No. | **27***^b^* | **28***^b^* | **29***^b^* | **30***^a^* | **31***^a^* |
| --- | --- | --- | --- | --- | --- |
| 1 | 0.92 m | 0.88 m | 0.91 m | 1.41 m | 1.42 m |
| 2a | 2.07 ddd (13.2, 7.2, 3.0) | 1.79 m | 2.09 ddd (13.2, 7.2, 3.0) | 2.70 dd (14.8, 6.4) | 2.71 dd (15.0, 6.6) |
| 2b | 1.46 q (11.4) | 1.69 m | 1.47 q (12.0) | 2.03 m | 2.05 m |
| 3 | 2.85 ddd (11.4, 7.2, 3.0) | 3.20 m | 2.87 ddd (10.8, 6.6, 3.0) |  |  |
| 5 | 3.89 dd (9.6, 7.2) | 4.25 dd (9.6, 8.4) | 3.87 dd (9.6, 7.2) | 6.94 t (1.8) | 7.01 t (1.8) |
| 6 | 1.53 m | 1.48 m | 1.54 m | 2.37 brt (12.0) | 2.38 brt (10.8) |
| 7 | 2.39 td (12.0, 3.0) | 2.42 td (11.4, 3.0) | 2.38 td (12.0, 3.0) | 2.49 td (12.0, 3.2) | 2.52 td (11.4, 3.6) |
| 8a | 1.75 m | 1.77 m | 1.74 m | 1.95 dq (13.6, 3.2) | 1.96 dq (13.2, 3.0) |
| 8b | 1.29 m | 1.27 m | 1.33 m | 1.24 m | 1.28 m |
| 9a | 1.72 m | 1.73 m | 1.72 m | 1.79 m | 1.80 m |
| 9b | 0.96 m | 0.95 m | 1.00 m | 1.07 m | 1.08 m |
| 10 | 1.26 m | 1.31 m | 1.27 m | 1.54 m | 1.56 m |
| 13a | 6.27 s | 6.26 d (0.6) | 6.25 s | 6.32 brs | 6.38 d (0.6) |
| 13b | 5.68 s | 5.68 s | 5.68 s | 5.58 brs | 5.61 brs |
| 14 | 0.90 d (6.0) | 0.93 d (6.6) | 0.92 d (6.6) | 0.92 d (6.4) | 0.93 d (6.0) |
| 15 | 2.22 s | 2.18 s | 2.24 s | 2.31 s | 2.32 s |
| 1' | 1.57 m | 1.56 m | 1.58 ddd (15.6, 8.4, 4.8) | 1.48 td (12.0, 4.0) | 1.53 m |
| 2'a | 1.70 m | 1.67 m | 1.74 m | 1.73 m | 1.70 m |
| 2'b | 1.67 m | 1.01 m | 1.67 m | 1.66 td (12.8, 4.0) | 1.70 m |
| 3'a | 1.96 m | 1.91 m | 1.94 dt (13.8, 4.2) | 1.99 m | 2.02 m |
| 3'b | 1.81 td (12.6, 4.2) | 1.81 td (12.6, 4.2) | 1.82 td (12.6, 3.6) | 1.77 m | 1.77 m |
| 5' | 4.21 s | 4.24 s | 4.75 s | 4.74 d (5.6) | 4.31 d (6.0) |
| 7' | 3.21 t (7.2) | 3.22 t (6.0) | 2.58 dt (10.8, 6.6) | 2.57 dt (11.2, 6.4) | 3.17 t (7.2) |
| 8'a | 1.91 m | 1.93 m | 1.76 m | 1.71 m | 1.89 m |
| 8'b | 1.34 m | 1.34 m | 1.07 m | 1.11 m | 1.38 m |
| 9'a | 1.58 m | 1.58 m | 1.64 m | 1.61 m | 1.61 m |
| 9'b | 1.13 m | 1.34 m | 1.10 m | 1.04 m | 1.12 m |
| 10' | 1.40 m | 1.35 m | 1.30 m | 1.38 m | 1.48 m |
| 11' |  |  | 3.11 m | 3.00 m |  |
| 13'a | 6.08 d (1.2) | 6.11 d (1.2) | 1.08 d (7.2) | 1.08 d (7.2) | 6.13 d (1.2) |
| 13'b | 5.65 d (0.6) | 5.66 d (1.2) |  |  | 5.53 d (1.2) |
| 14' | 0.98 d (6.6) | 0.97 d (6.6) | 0.97 d (6.6) | 0.93 d (6.4) | 0.96 d (6.0) |
| 15' | 1.52 s | 1.51 s | 1.56 s | 1.60 s | 1.60 s |
| 5'-OH |  |  |  |  | 2.32 d (6.0) |

*^a^*Recorded in CDCl_3_; *^b^*recorded in CD_3_OD.

# supplementary Table 11 ^1^H NMR (600 MHz, *δ* in ppm, *J* in Hz) data for compounds 32–36.

| No. | **32***^a^* | **33***^b^* | **34***^a^* | **35***^b^* | **36***^a^* |
| --- | --- | --- | --- | --- | --- |
| 1 | 1.13 dt (11.4, 2.4) | 1.32 m | 1.49 m | 1.59 m | 0.98 m |
| 2a | 1.65 m | 2.72 ddd (17.4, 10.2, 3.6) | 1.86 m | 1.93m | 2.08 ddd (12.6, 7.2, 3.6) |
| 2b | 1.38 m | 2.47 ddd (17.4, 7.2, 3.0) | 1.62 m | 1.54 m | 1.52 m |
| 3a | 1.82 m | 1.94 m | 2.58 ddd (16.2, 11.4, 4.2) | 2.70 ddd (16.8, 12.6, 4.2) | 2.80 ddd (10.8, 6.6, 3.6) |
| 3b | 1.63 m | 1.94 m | 2.33 m | 2.43 ddd (16.8, 12.0, 4.2) |  |
| 5 | 2.94 s | 6.66 dd (11.4, 4.2) |  |  | 3.98 ddd (9.6, 6.6, 4.8) |
| 6 |  | 2.67 m | 2.37 t (11.4) | 2.61 t (10.2) | 1.56 m |
| 7 | 3.09 dd (13.8, 1.8) | 2.58 td (11.4, 3.0) | 2.81 brt (11.4) | 2.99 brt (9.6) | 2.36 m |
| 8a | 1.72 m | 1.91 m | 1.90 m | 1.96 m | 1.69 dq (13.2, 3.0) |
| 8b | 1.40 m | 1.36 m | 1.77 m | 1.12 m | 1.50 m |
| 9a | 1.72 m | 1.80 m | 1.73 m | 1.76 m | 1.74 dq (13.2, 3.0) |
| 9b | 1.21 m | 1.18 m | 1.67 m | 1.20 m | 0.98 m |
| 10 | 1.26 m | 1.45 m | 2.06 m | 1.37 m | 1.26 m |
| 13a | 6.43 s | 6.30 d (0.6) | 6.30 s | 6.28 s | 6.13 s |
| 13b | 5.46 s | 5.57 s | 5.67 s | 5.72 s | 5.63 s |
| 14 | 0.90 d (6.0) | 1.04 d (6.0) | 0.92 d (6.0) | 0.96 d (6.6) | 0.89 d (6.6) |
| 15 | 1.33 s | 5.91 dt (11.4, 1.8) | 2.14 s | 2.13 s | 2.22 s |
| 1'a | 1.50 m | 1.59 m | 1.56dt (10.8, 5.4) | 1.84 ddd (18.0, 13.2, 4.8) | 2.15 m |
| 1'b |  |  |  | 1.01 m |  |
| 2'a | 1.63 m | 1.71 m | 1.64 m | 1.62 m | 2.41 dt (9.0, 6.0) |
| 2'b | 1.63 m | 1.65 m | 0.88 m | 1.49 m | 1.19 brd (9.0) |
| 3'a | 2.36 dt (13.8, 4.2) | 1.89 m | 1.76 m | 2.10 m |  |
| 3'b | 1.70 m | 1.84 m | 1.28 m | 1.98 m |  |
| 4' |  |  |  |  | 5.60 m |
| 5'a | 3.99 s | 4.27 s | 4.19 s |  | 2.33 m |
| 5'b |  |  |  |  | 2.26 m |
| 6'a |  |  |  | 2.40 dd (12.6, 3.0) | 2.12 m |
| 6'b |  |  |  | 1.73 t (13.8) |  |
| 7' | 3.24 t (6.6) | 3.22 t (7.2) | 3.22 t (6.0) | 2.21 m |  |
| 8'a | 1.88 m | 1.95 m | 1.88 m | 1.62 m | 1.29 s |
| 8'b | 1.46 m | 1.33 m | 1.42 m | 1.56 m |  |
| 9'a | 1.60 m | 1.61 m | 1.60 m | 1.86 m | 0.84 s |
| 9'b | 1.09 m | 1.15 m | 1.10 m | 1.25 m |  |
| 10' | 1.48 m | 1.42 m | 1.41 m |  | 4.53 s |
| 12' |  |  |  | 1.75 s |  |
| 13'a | 6.16 d (1.2) | 6.09 d (1.2) | 6.18 s | 4.76 s |  |
| 13'b | 5.54 d (1.2) | 5.65 d (1.2) | 5.56 s | 4.73 s |  |
| 14' | 0.93 d (6.6) | 0.99 d (6.6) | 0.94 d (6.6) | 0.88 s |  |
| 15'a | 1.56 s | 1.53 s | 1.52 s | 4.90 overlaped |  |
| 15'b |  |  |  | 4.74 s |  |
| 5-OH |  |  |  |  | 1.96 d (4.8) |

*^a^*Recorded in CDCl_3_; *^b^*recorded in CD_3_OD.

**supplementary Table 12** Cytotoxicity of compounds **1–3** and **6–36** from *A. eriopoda*.*^a^*

| No. | IC_50_ (μM) | | | | |
| --- | --- | --- | --- | --- | --- |
|  | HepG2 |  | Huh7 |  | SK-Hep-1 |
| **1** | 52.1 ± 1.3 |  | 91.8 ± 0.6 |  | 106.2 ± 1.1 |
| **2** | 134.2 ± 2.0 |  | 156.8 ± 2.2 |  | 165.8 ± 2.4 |
| **3** | 94.2 ± 0.9 |  | 109.4 ± 0.4 |  | 135.7 ± 1.3 |
| **6** | 71.6 ± 2.7 |  | ˃ 200 |  | 87.1 ± 0.9 |
| **7** | ˃ 200 |  | ˃ 200 |  | ˃ 200 |
| **8** | ˃ 200 |  | ˃ 200 |  | 192.3 ± 2.5 |
| **9** | 197.6 ± 11.4 |  | ˃ 200 |  | ˃ 200 |
| **10** | 133.9 ± 1.7 |  | 120.4 ± 1.8 |  | 170.2 ± 2.4 |
| **11** | ˃ 200 |  | ˃ 200 |  | 121.5 ± 1.5 |
| **12** | 34.7 ± 1.5 |  | 57.1 ±0.7 |  | 65.9 ± 0.4 |
| **13** | 14.3 ± 0.6 |  | 39.5 ± 0.5 |  | 54.2 ± 0.4 |
| **14** | ˃ 200 |  | ˃ 200 |  | 164.1 ± 3.8 |
| **15** | ˃ 200 |  | ˃ 200 |  | ˃ 200 |
| **16** | 12.2 ± 0.4 |  | 53.1 ±1.0 |  | 50.3 ± 0.3 |
| **17** | ˃ 200 |  | ˃ 200 |  | ˃ 200 |
| **18** | 44.2 ± 0.9 |  | 131.9 ± 0.5 |  | 71.4 ± 0.2 |
| **19** | 83.4 ± 2.7 |  | 100.0 ± 1.0 |  | 105.7 ± 0.6 |
| **20** | ˃ 200 |  | ˃ 200 |  | ˃ 200 |
| **21** | 89.8 ± 1.4 |  | ˃ 200 |  | ˃ 200 |
| **22** | 91.7 ± 2.6 |  | 158.6 ± 1.6 |  | 110.1 ± 2.0 |
| **23** | 88.3 ± 1.7 |  | ˃ 200 |  | 104.7 ± 1.3 |
| **24** | 67.2 ± 3.1 |  | 103.7 ± 0.4 |  | 40.0 ± 1.3 |
| **25** | 146.5 ± 2.1 |  | ˃ 200 |  | 100.5 ± 0.7 |
| **26** | 176.0 ± 3.5 |  | ˃ 200 |  | 193.7 ± 3.8 |
| **27** | 34.0 ± 0.6 |  | 56.2 ± 0.3 |  | 51.5 ± 0.2 |
| **28** | 25.1 ± 0.3 |  | 37.6 ± 0.8 |  | 25.4 ± 0.9 |
| **29** | 91.8 ± 2.5 |  | 103.1 ± 2.4 |  | ˃ 200 |
| **30** | 33.0 ± 1.7 |  | 39.2 ± 0.6 |  | 38.5 ± 0.7 |
| **31** | 17.2 ± 0.6 |  | 10.3 ± 1.7 |  | 22.3 ± 0.3 |
| **32** | 45.3 ± 1.4 |  | 53.3 ± 0.2 |  | 55.4 ± 1.5 |
| **33** | 16.0 ± 0.5 |  | 18.3 ± 0.9 |  | 19.0 ± 0.5 |
| **34** | 69.9 ± 1.4 |  | 97.5 ± 1.1 |  | 42.6 ± 1.6 |
| **35** | 61.8 ± 2.8 |  | 118.2 ± 0.4 |  | 64.0 ± 1.0 |
| **36** | 69.5 ± 1.3 |  | 153.1 ± 0.6 |  | 88.0 ± 0.8 |
| Sorafenib | 11.0 ± 1.4 |  | 12.3 ± 0.3 |  | 18.1 ± 0.2 |

*^a^*Data were expressed as mean ± SD (n = 3)

# S1. ^1^H NMR (600 MHz, CDCl_3_) of compound 1


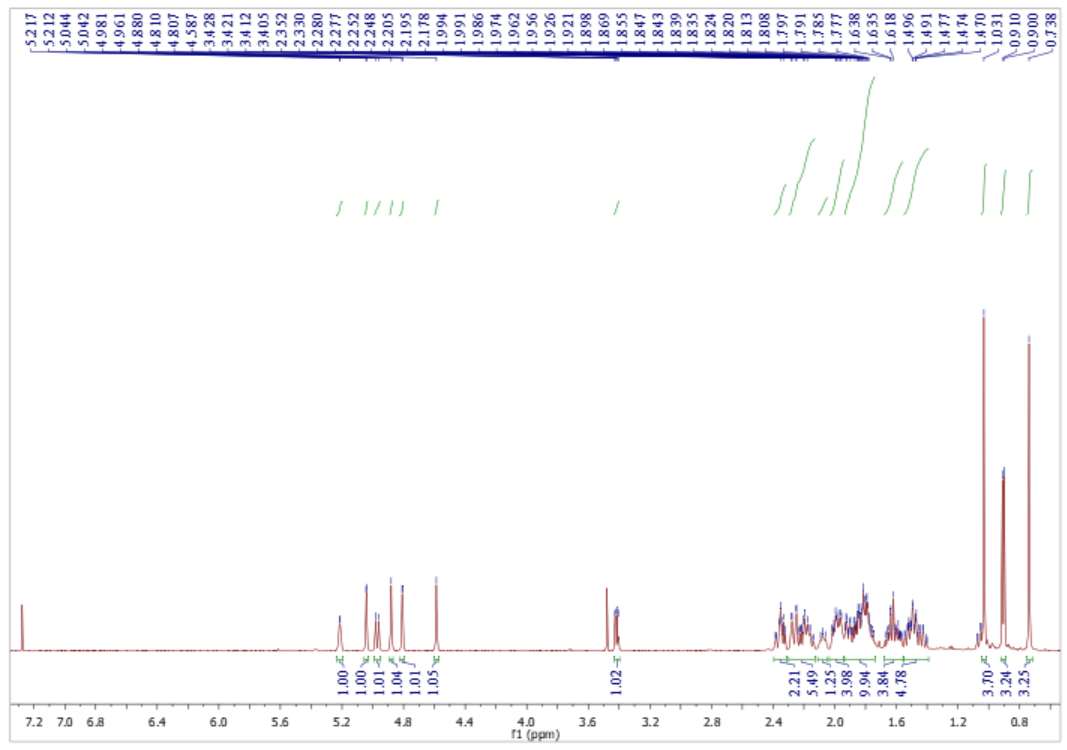


# S2. ^13^C NMR (DEPT) (150 MHz, CDCl_3_) of compound 1


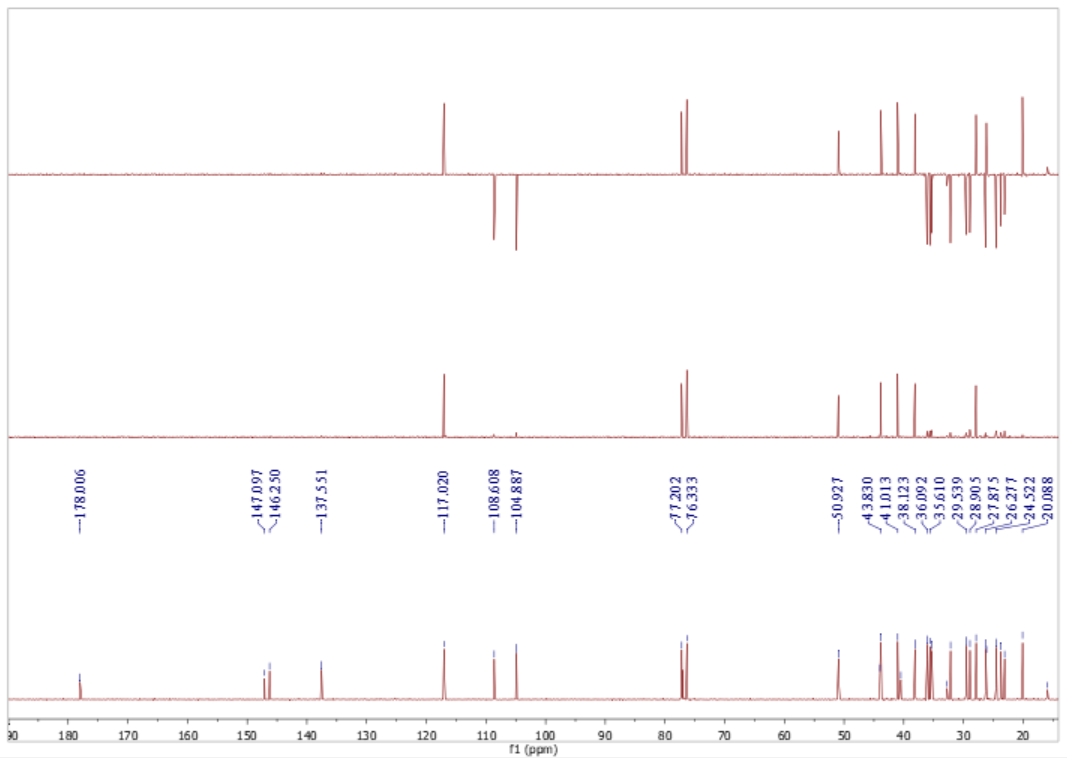


# S3. ^1^H-^1^H COSY (600 MHz, CDCl_3_) of compound 1


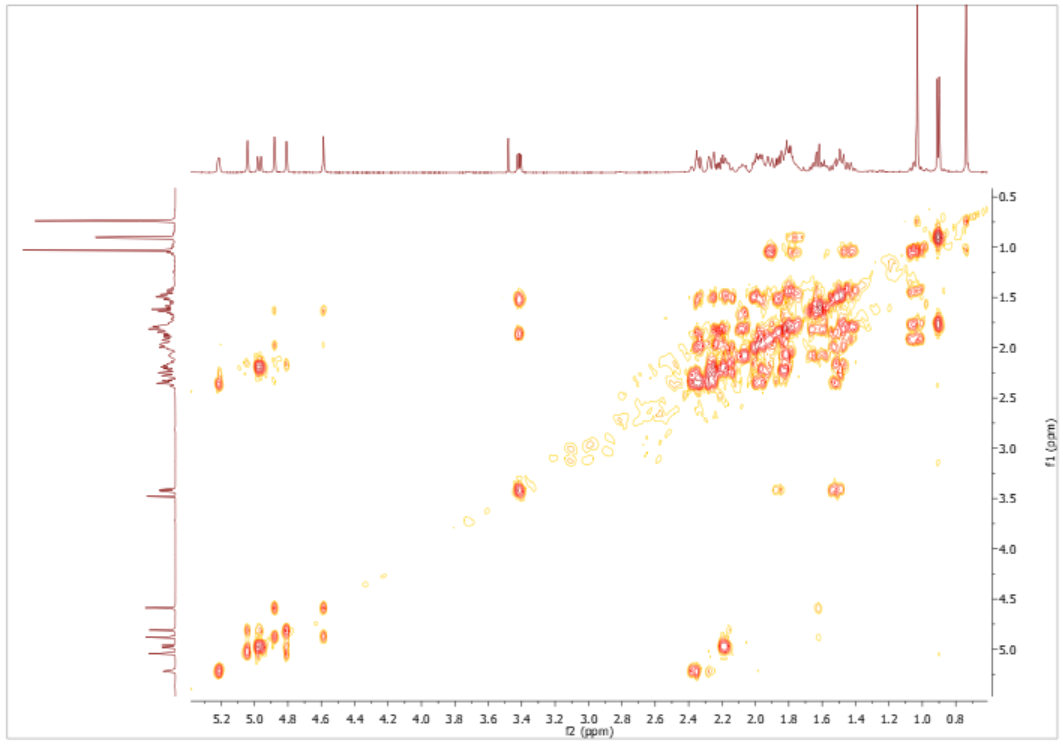


# S4. HSQC (600 MHz, CDCl_3_) of compound 1


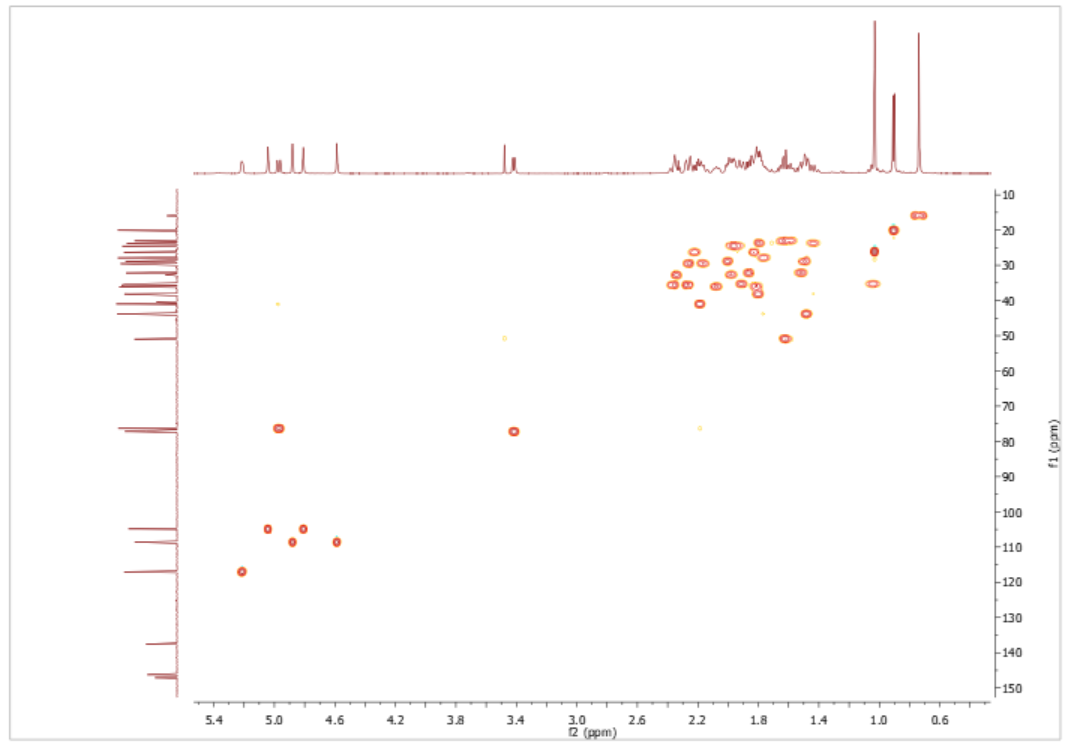


# S5. HMBC (600 MHz, CDCl_3_) of compound 1


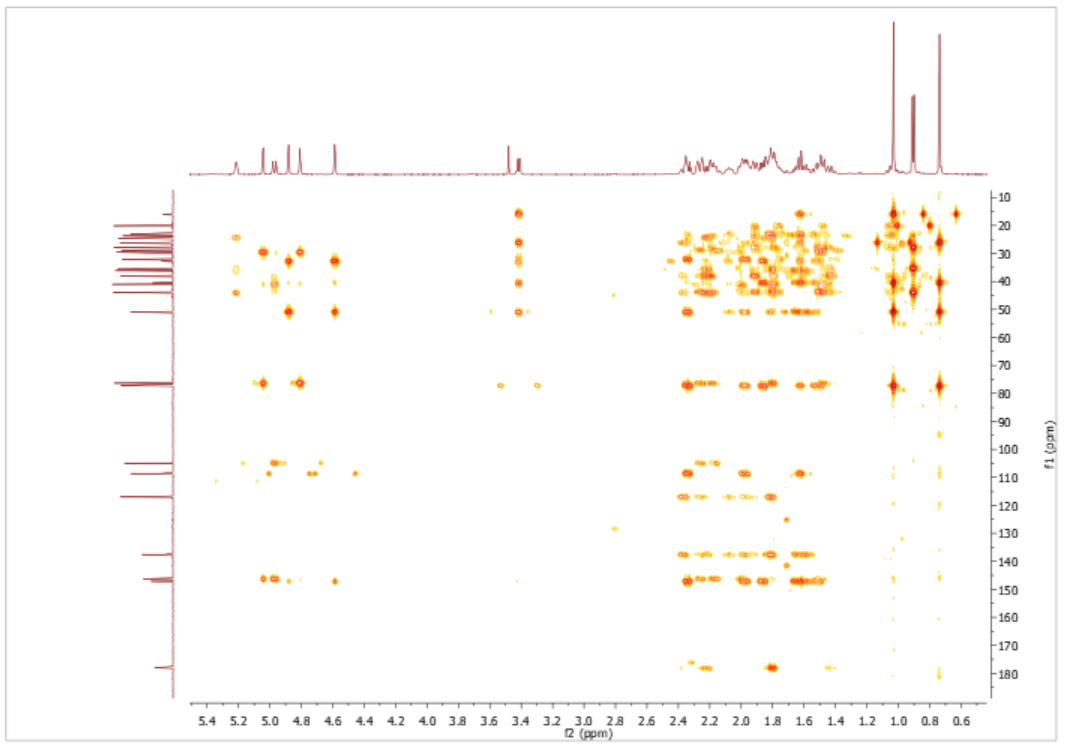


# S6. ROESY (600 MHz, CDCl_3_) of compound 1


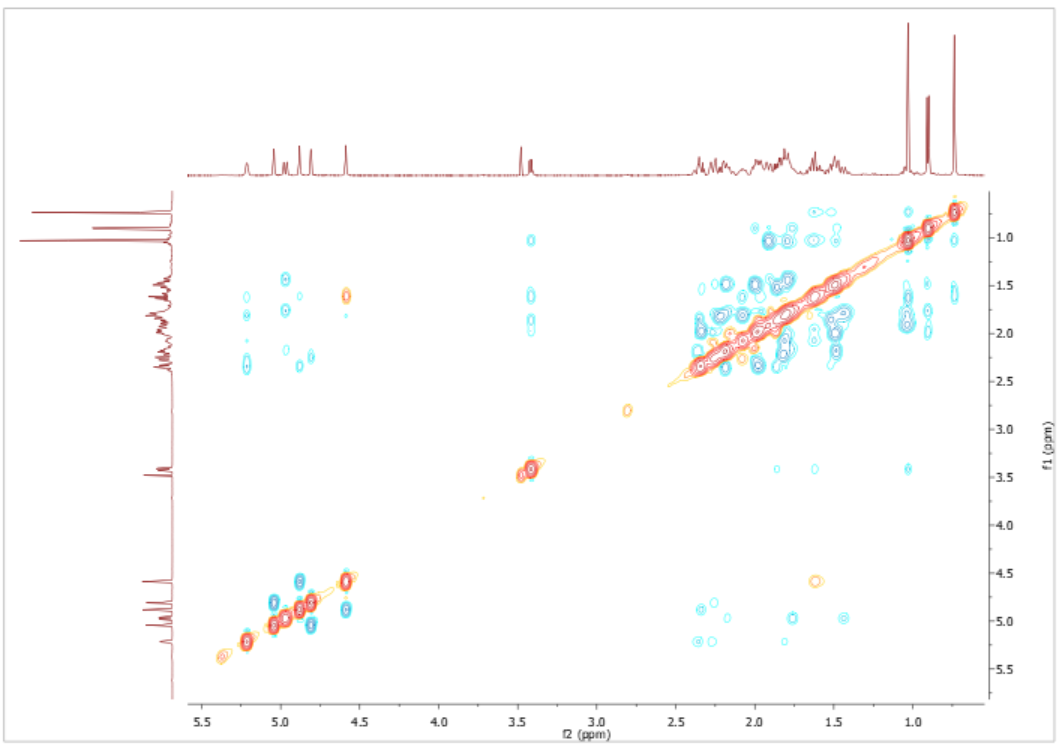


# S7. IR of compound 1


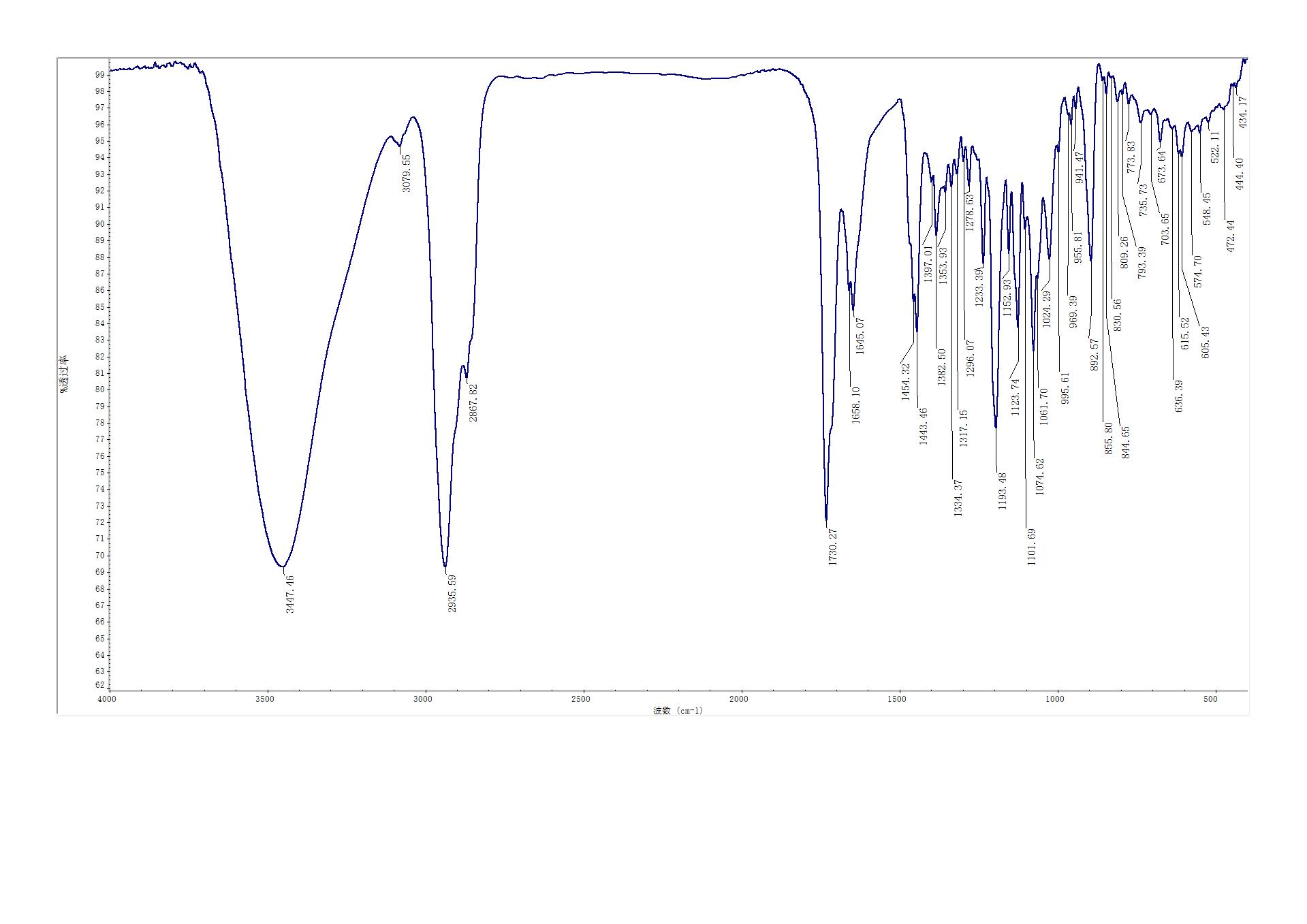


# S8. ECD of compound 1


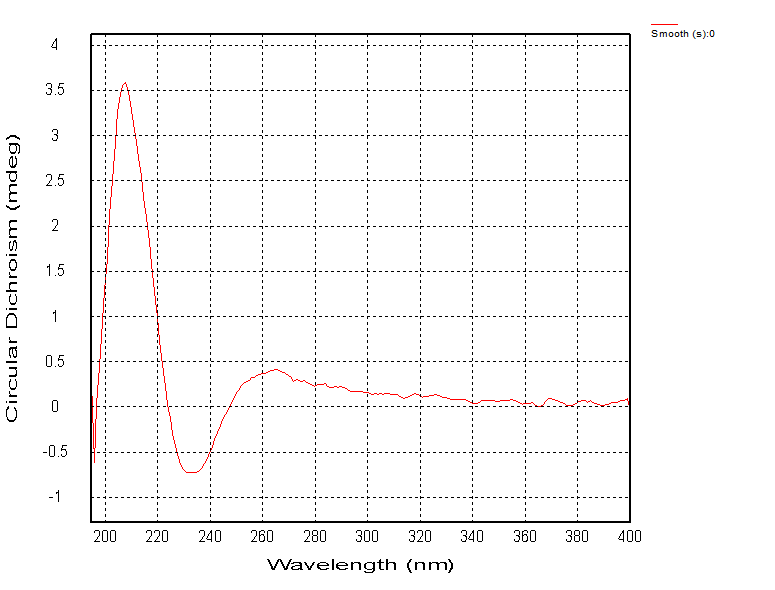


# S9. HRESIMS of compound 1


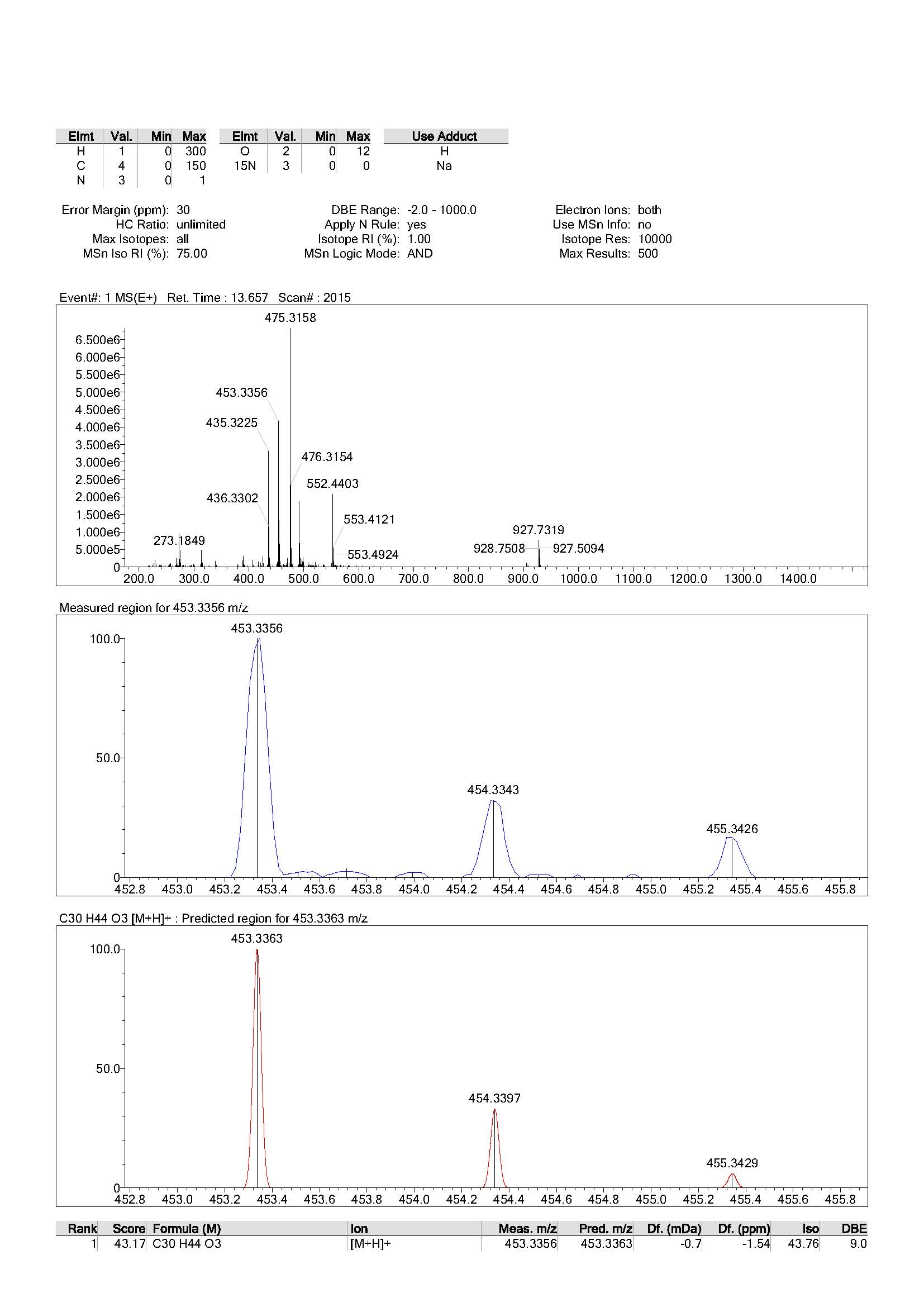


# S10. ^1^H NMR (600 MHz, CDCl_3_) of compound 2


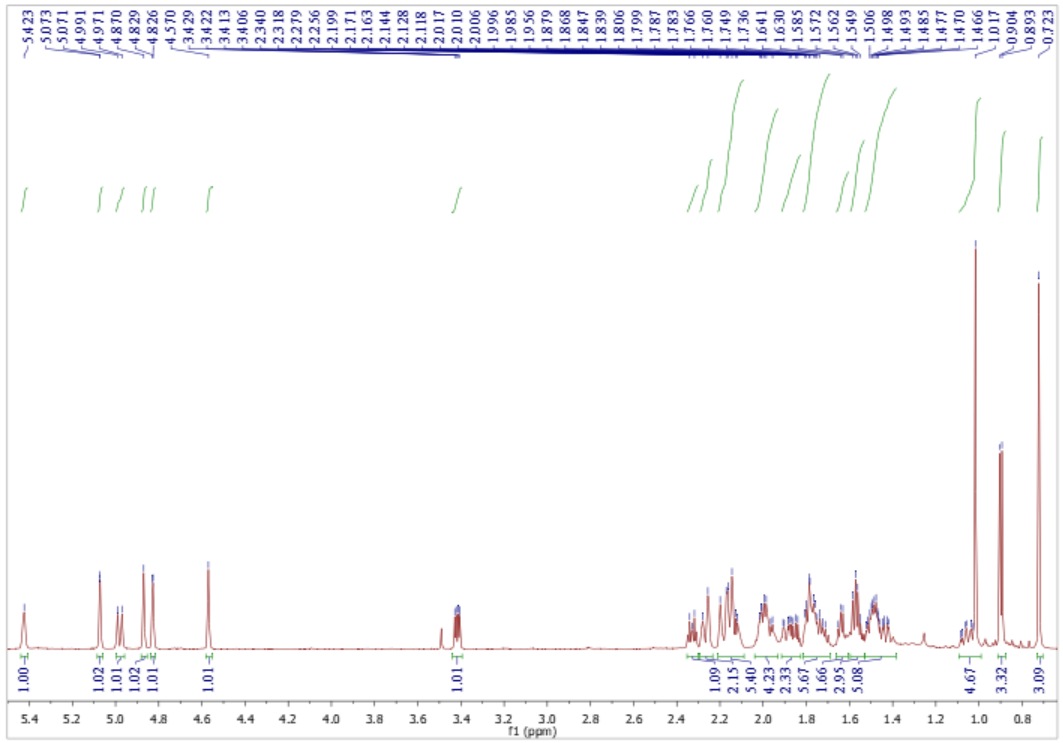


# S11. ^13^C NMR (DEPT) (150 MHz, CDCl_3_) of compound 2


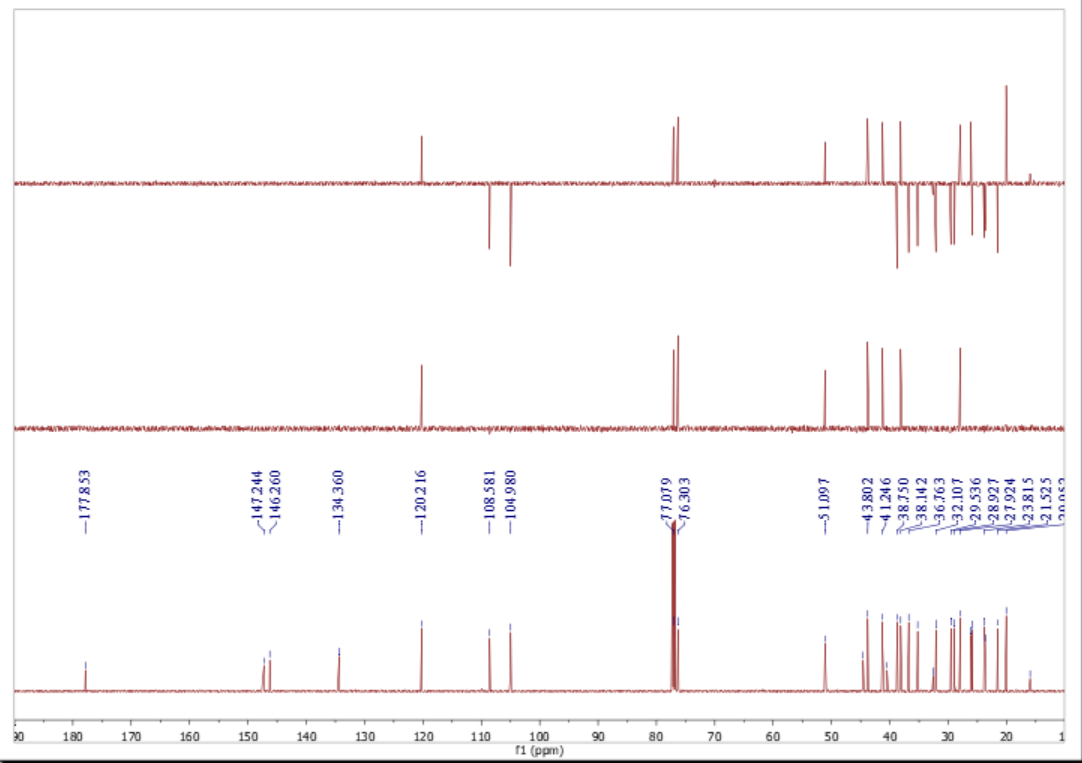


# S12. ^1^H-^1^H COSY (600 MHz, CDCl_3_) of compound 2


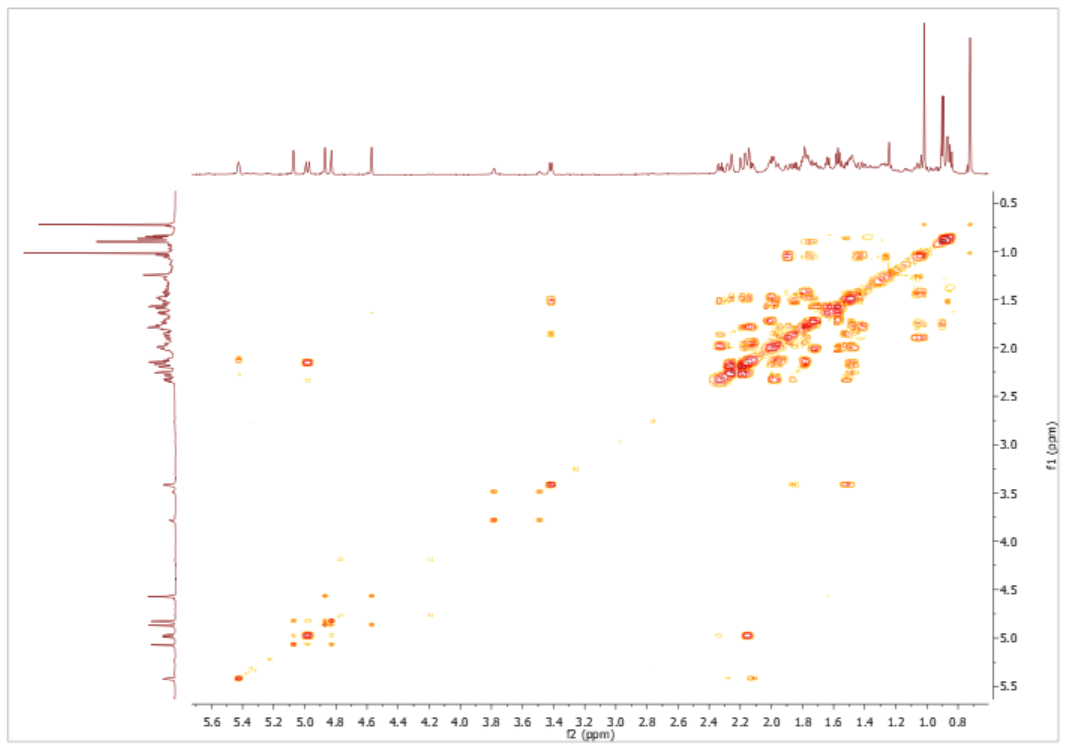


# S13. HSQC (600 MHz, CDCl_3_) of compound 2


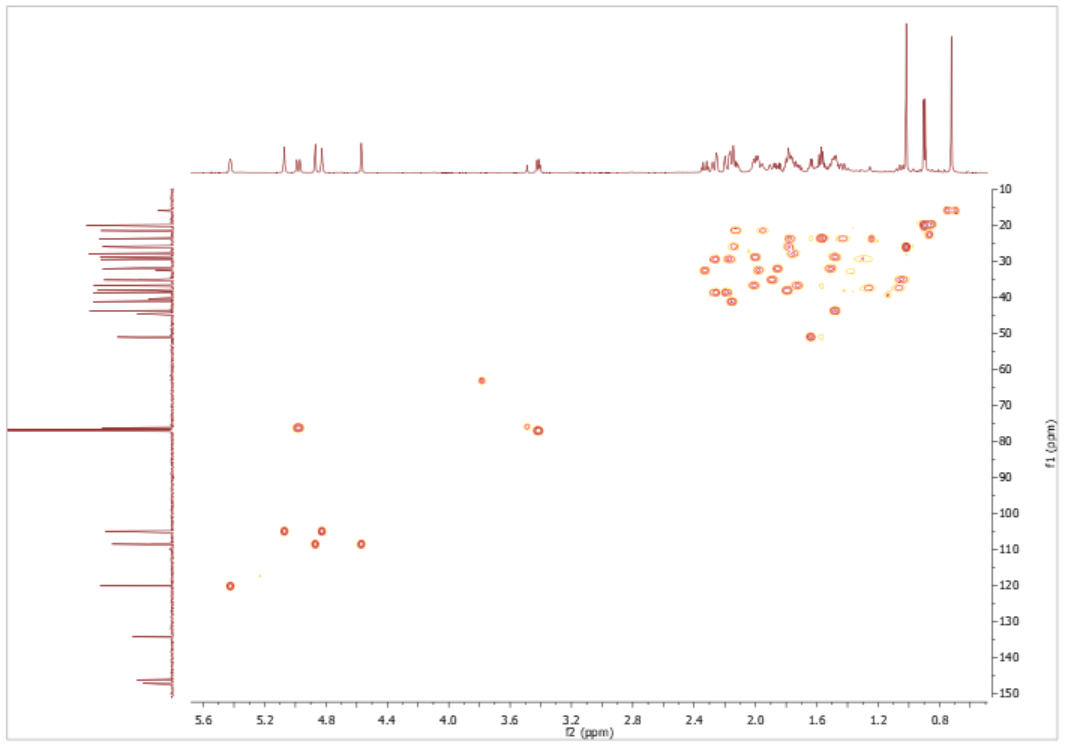


# S14. HMBC (600 MHz, CDCl_3_) of compound 2


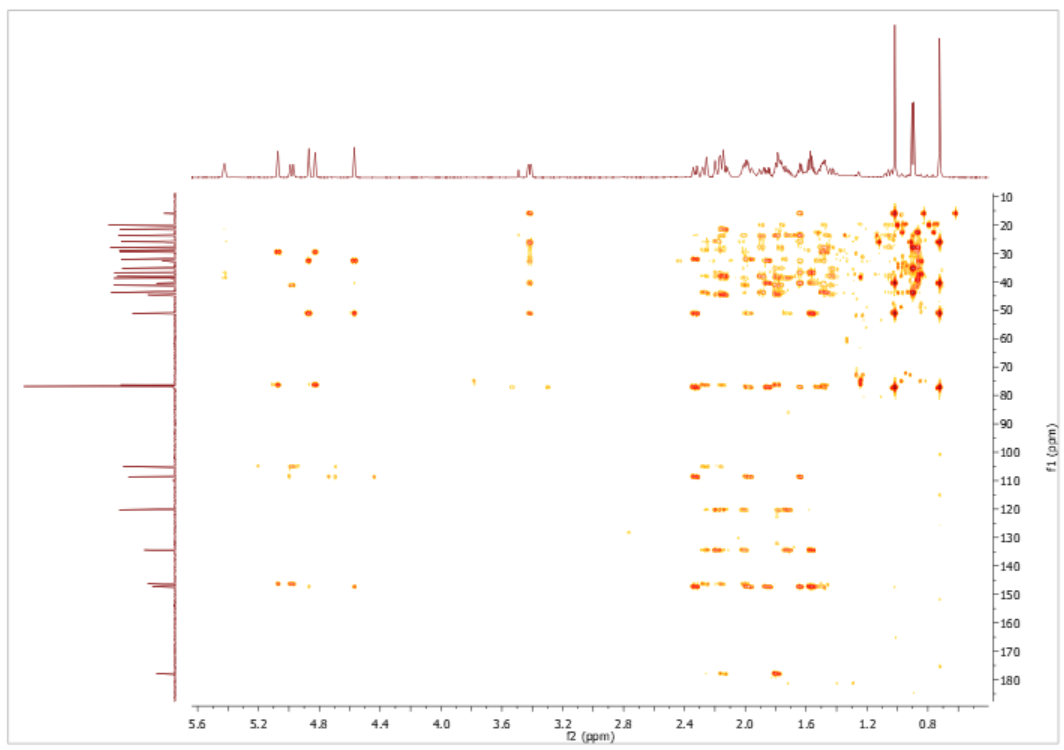


# S15. ROESY (600 MHz, CDCl_3_) of compound 2


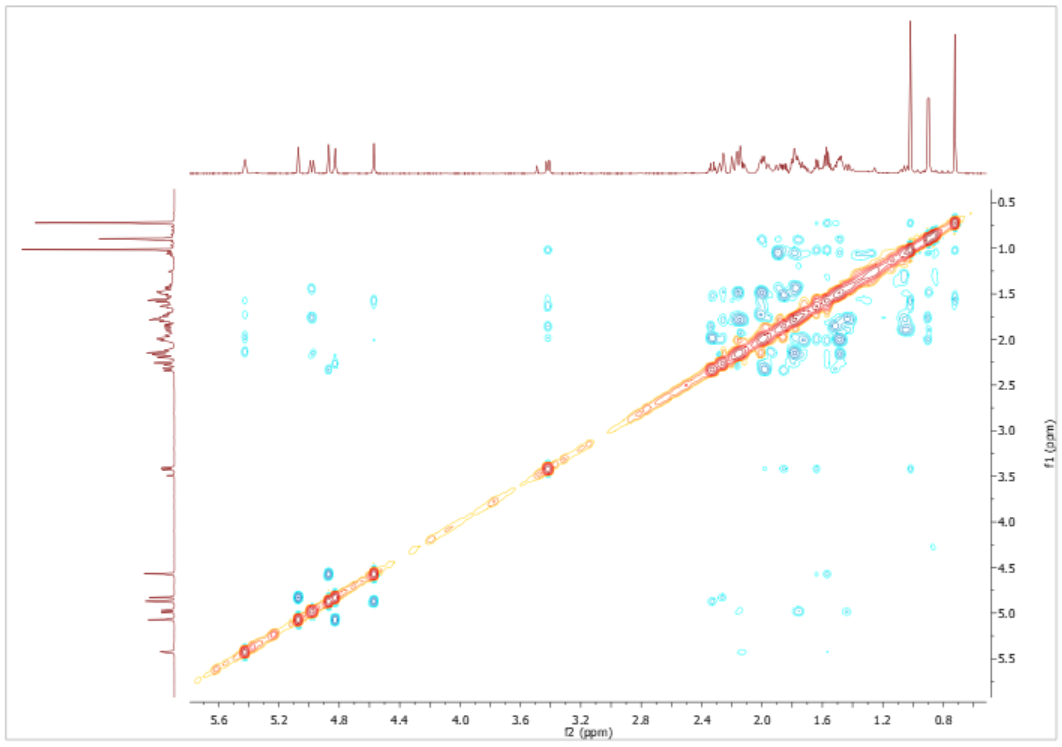


# S16. IR of compound 2


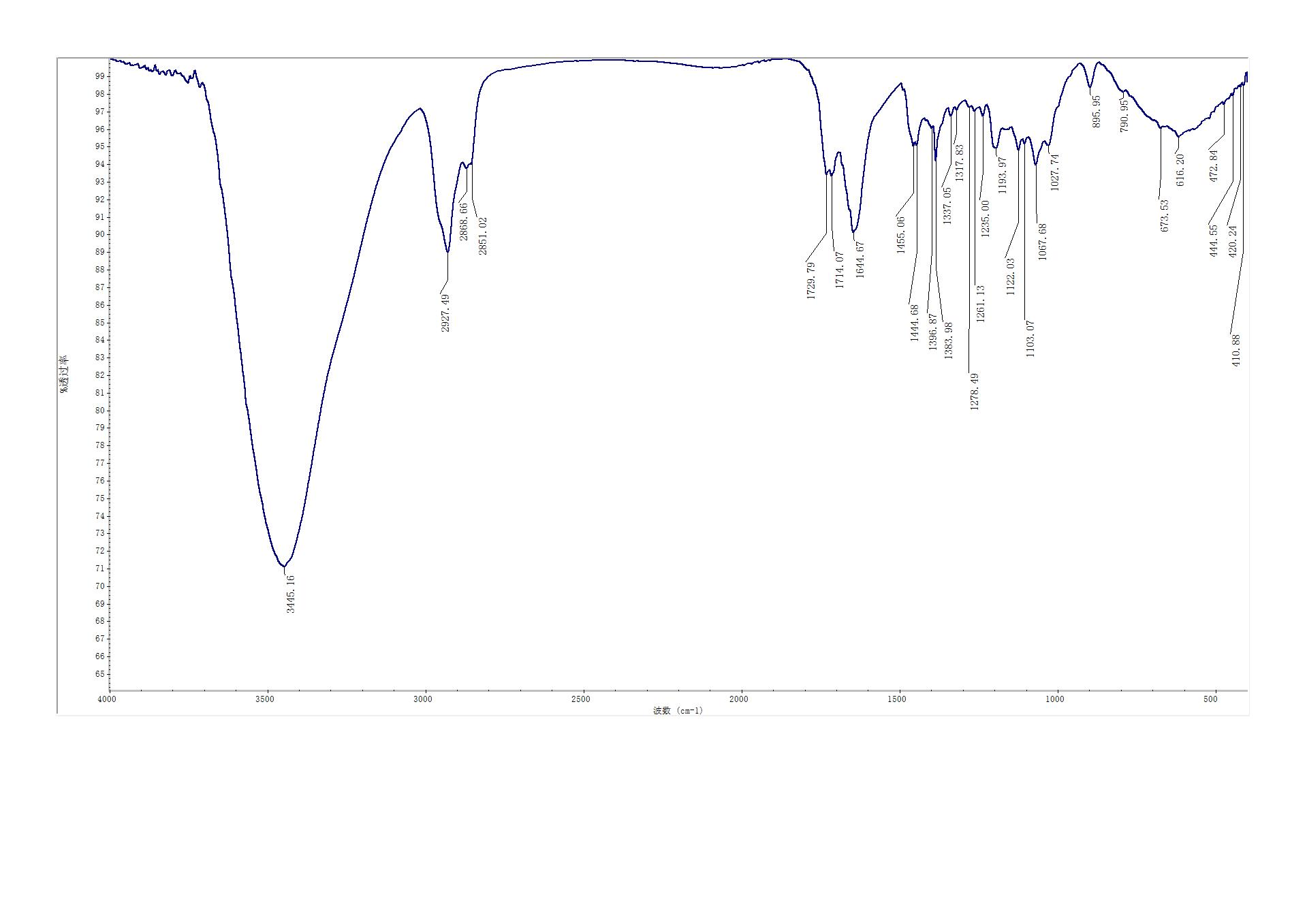


# S17. ECD of compound 2


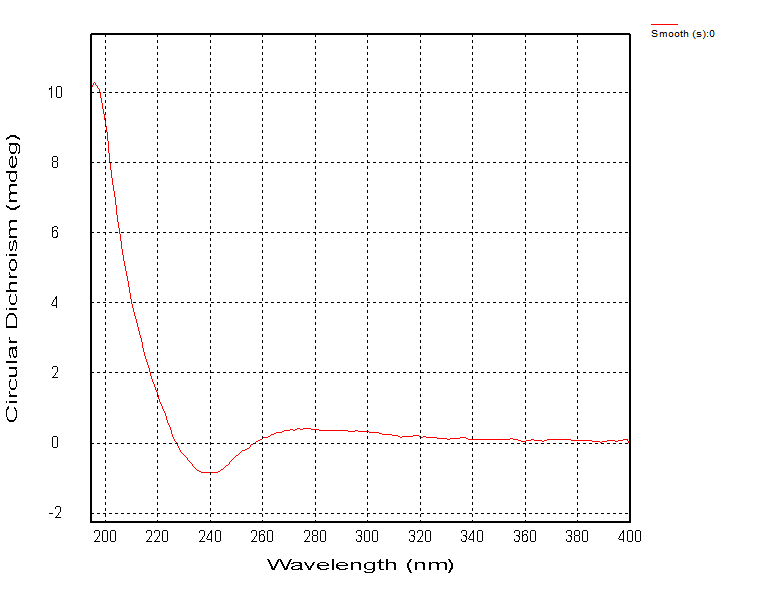


# S18. HRESIMS of compound 2


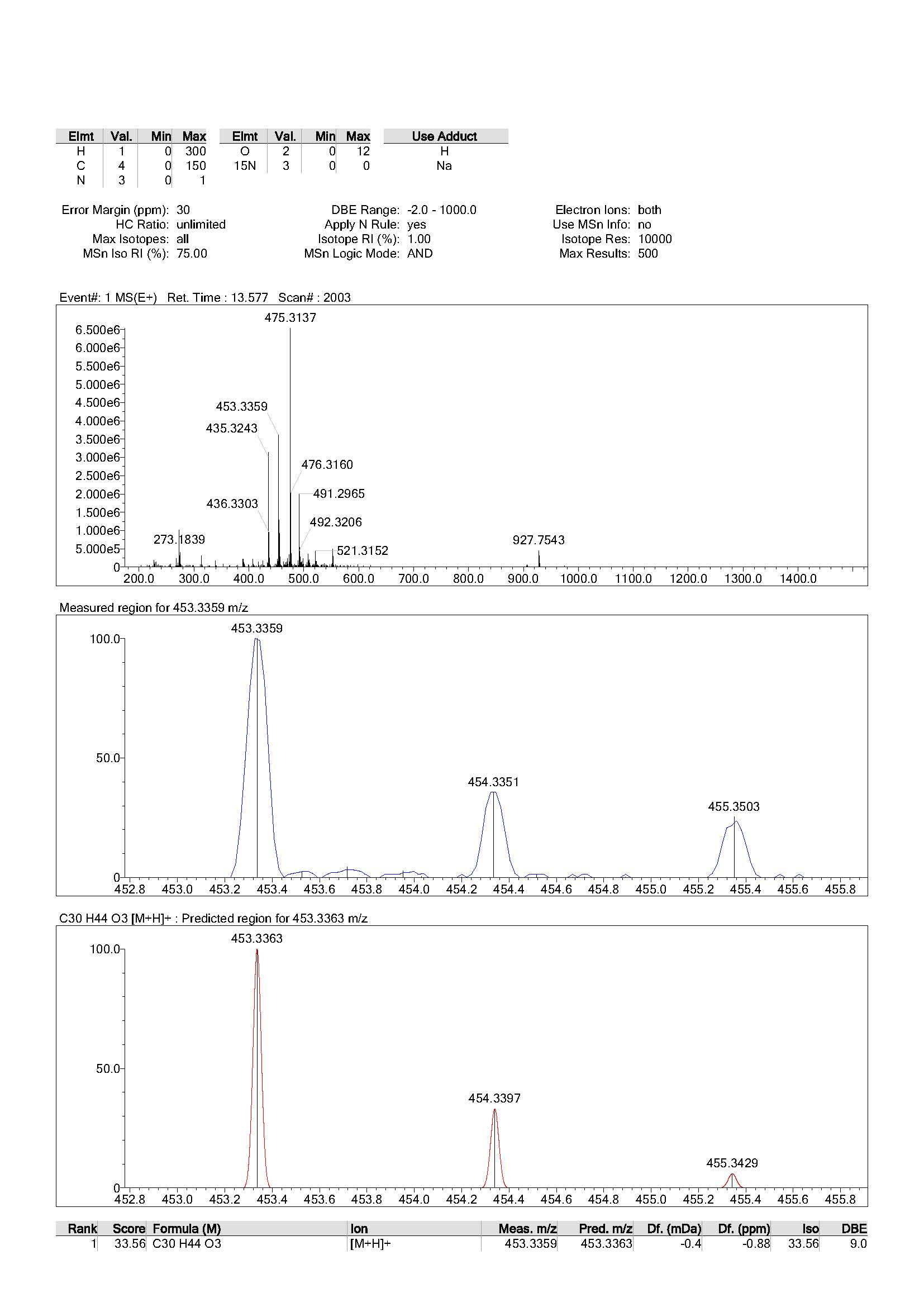


S19. ^1^H NMR (600 MHz, CDCl3) of compound **3**


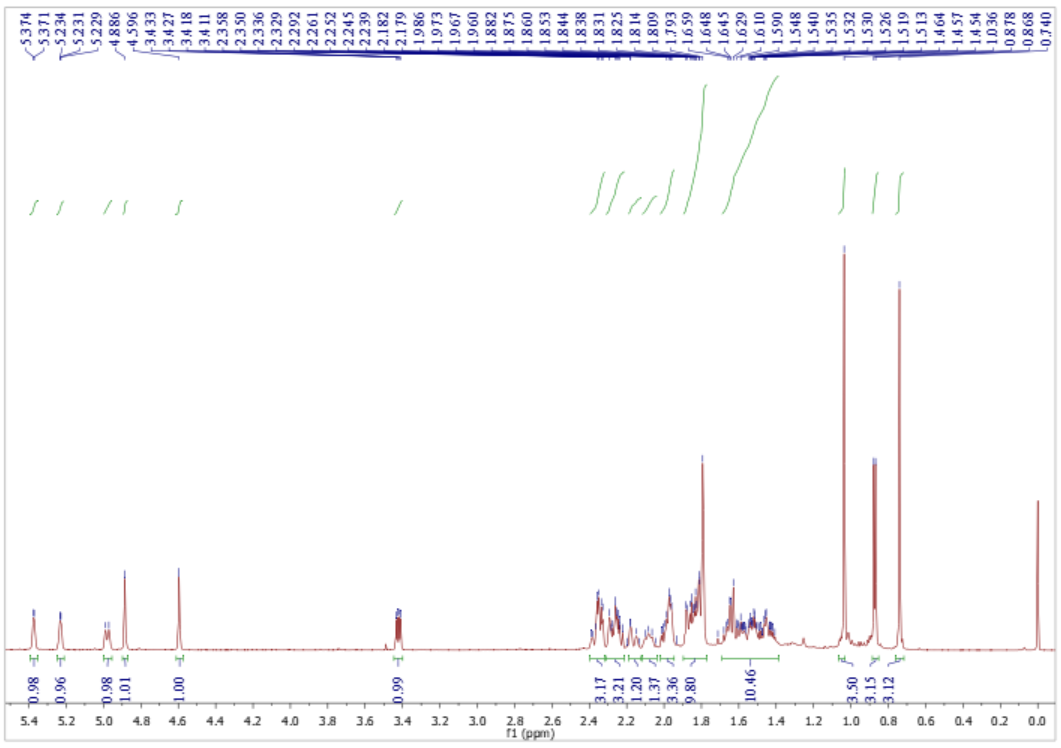


# S20. ^13^C NMR (DEPT) (150 MHz, CDCl_3_) of compound 3


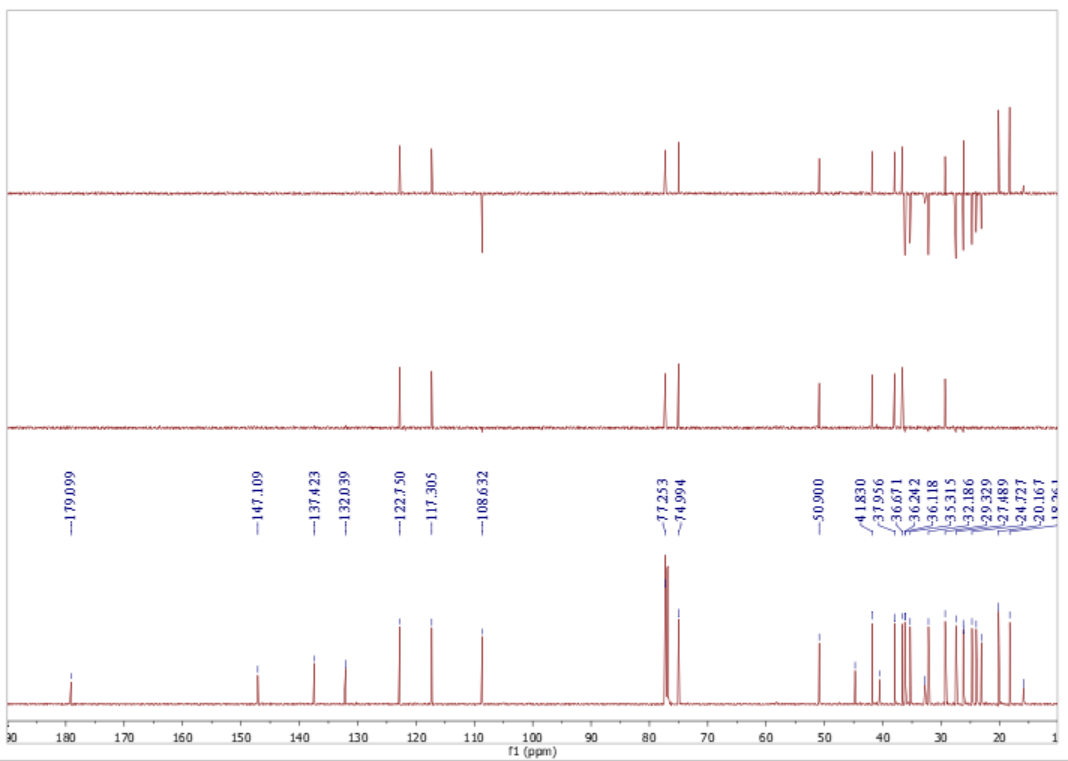


# S21. ^1^H-^1^H COSY (600 MHz, CDCl_3_) of compound 3


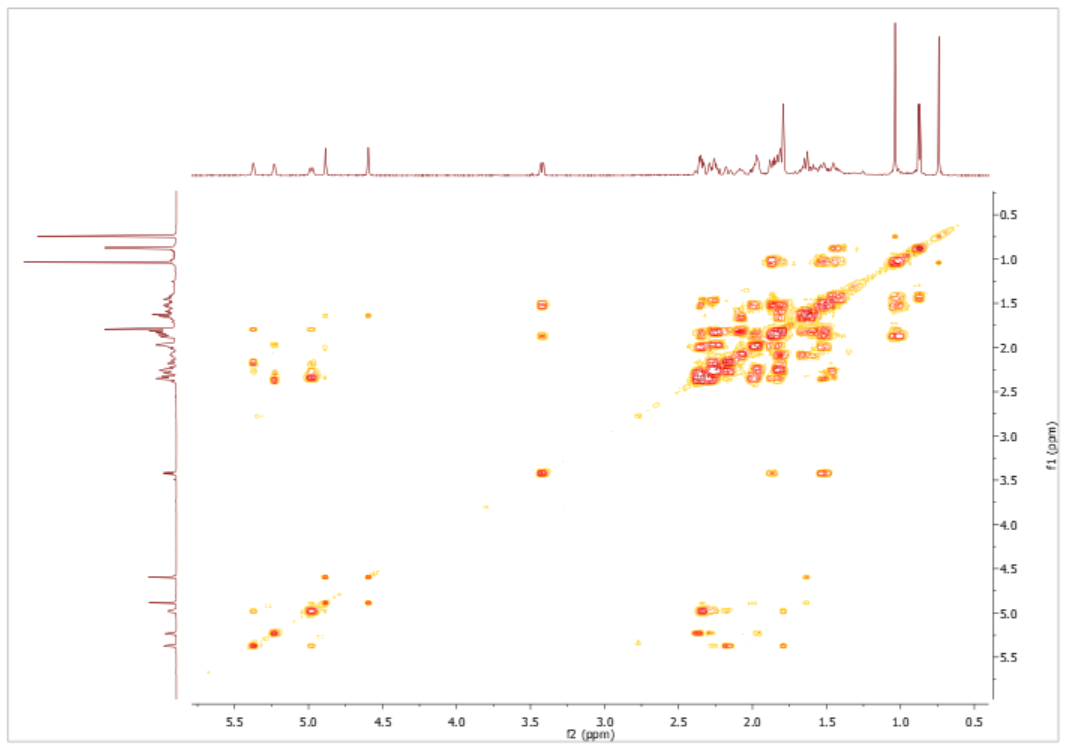


# S22. HSQC (600 MHz, CDCl_3_) of compound 3


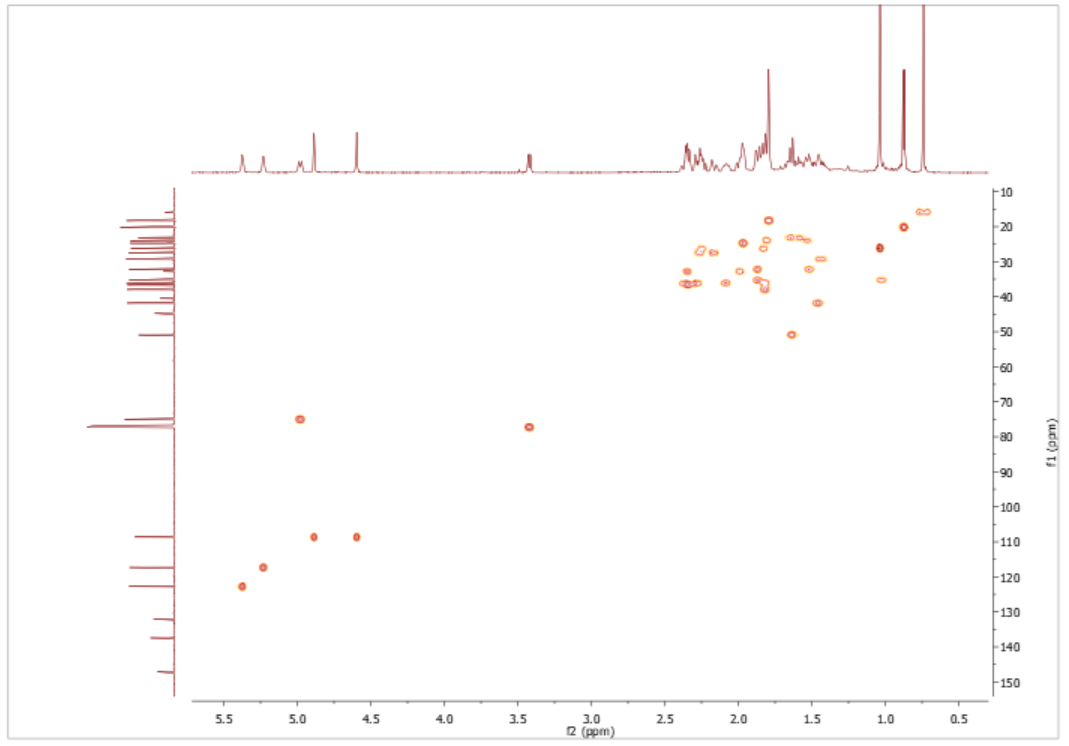


# S23. HMBC (600 MHz, CDCl_3_) of compound 3


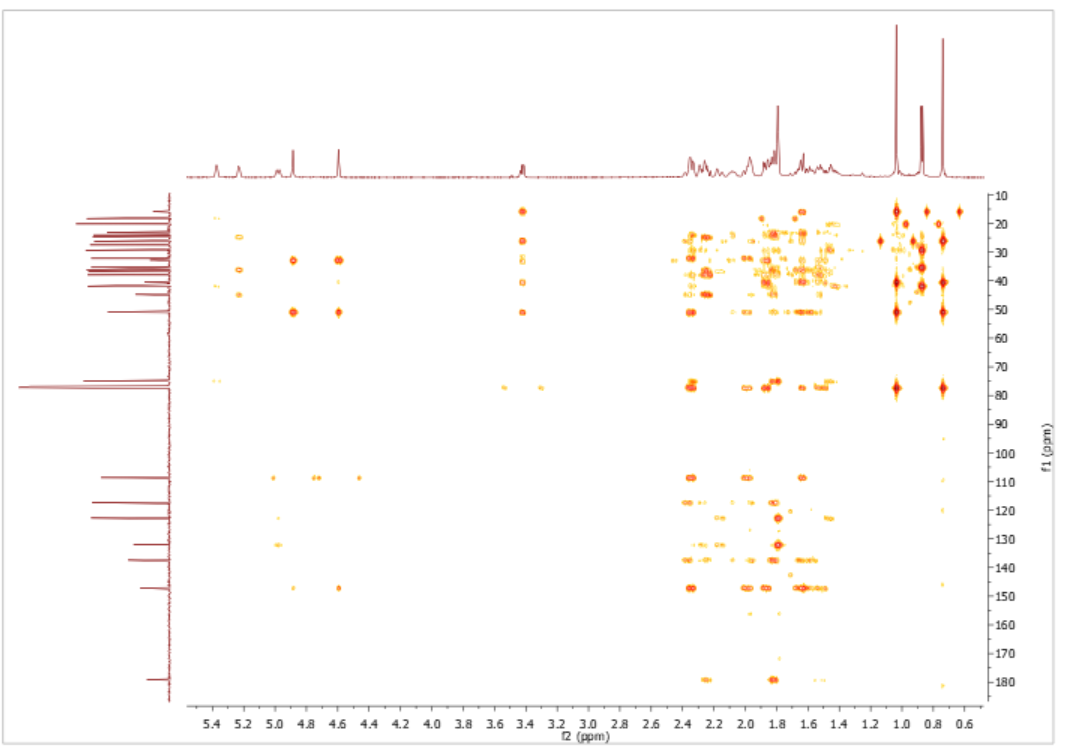


# S24. ROESY (600 MHz, CDCl_3_) of compound 3


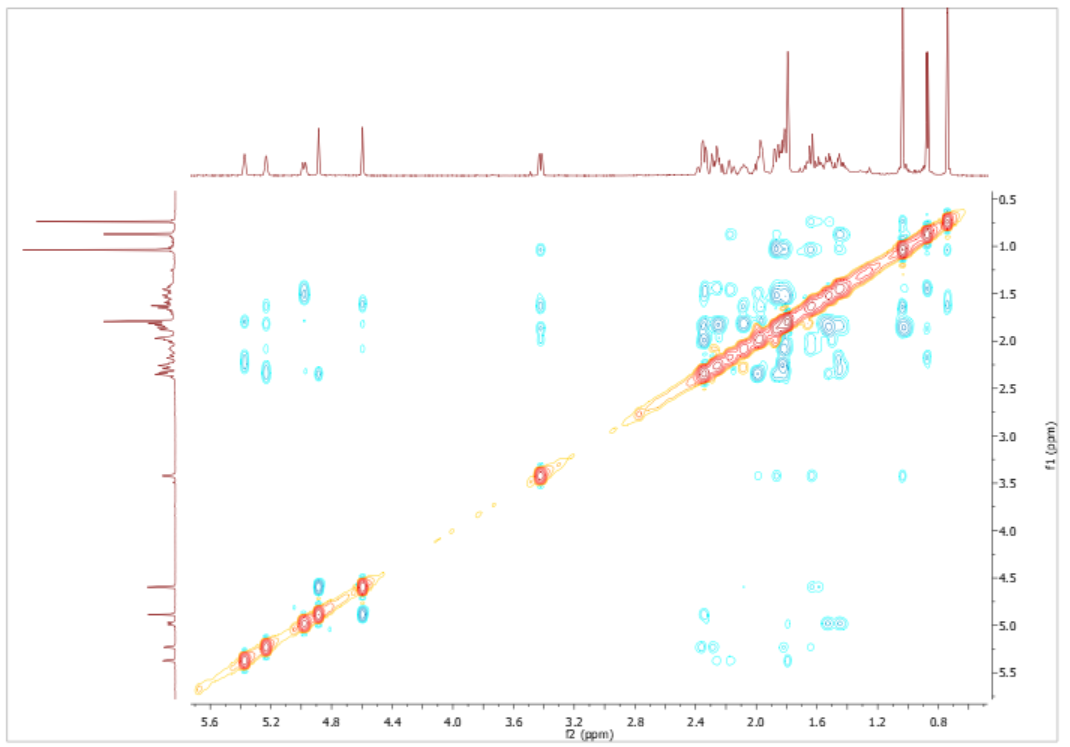


# S25. IR of compound 3


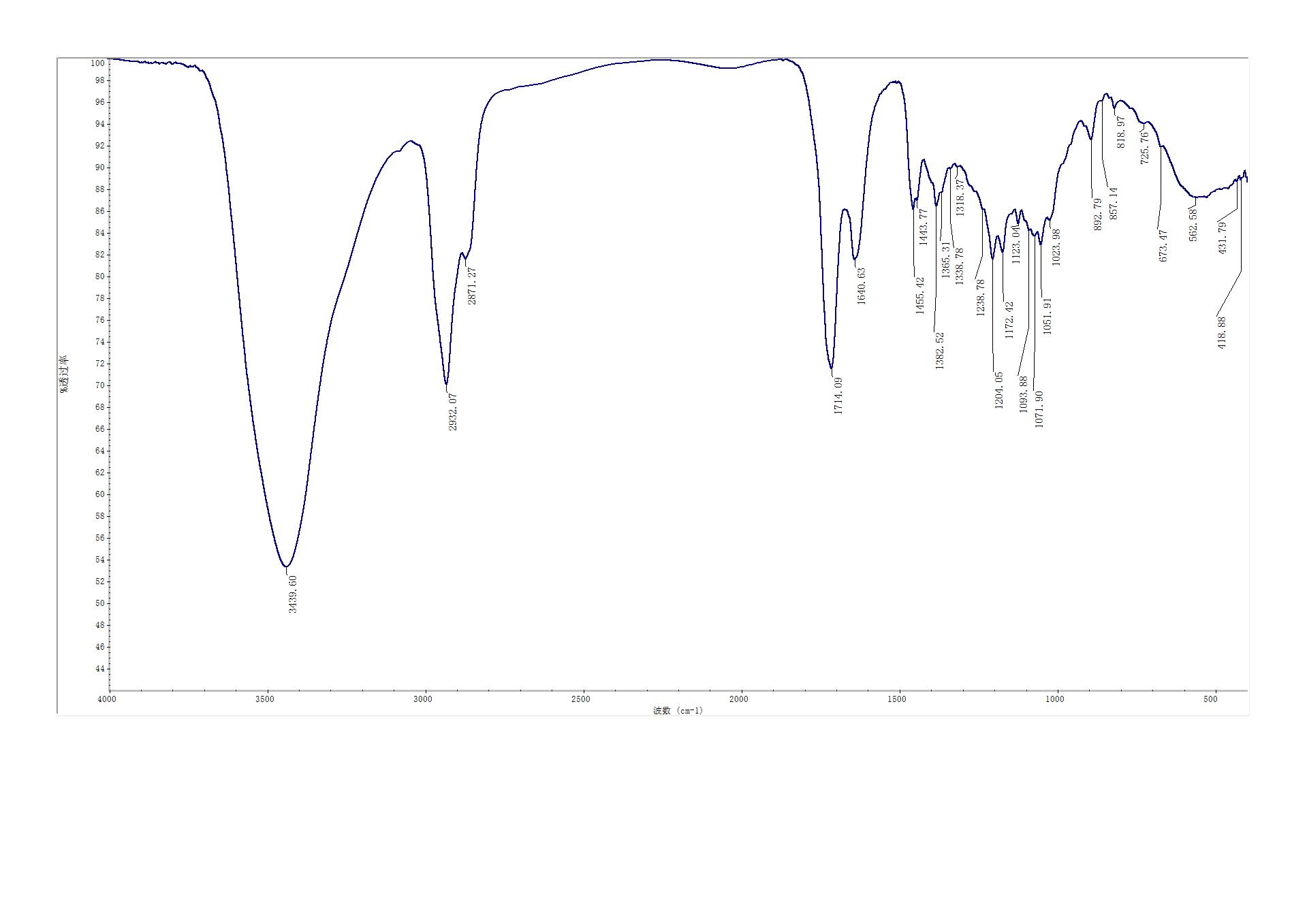


# S26. ECD of compound 3


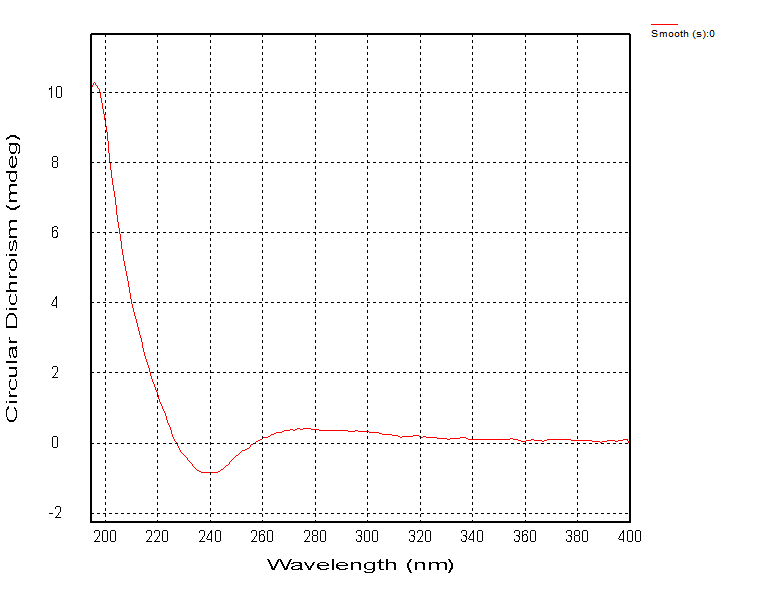


# S27. HRESIMS of compound 3


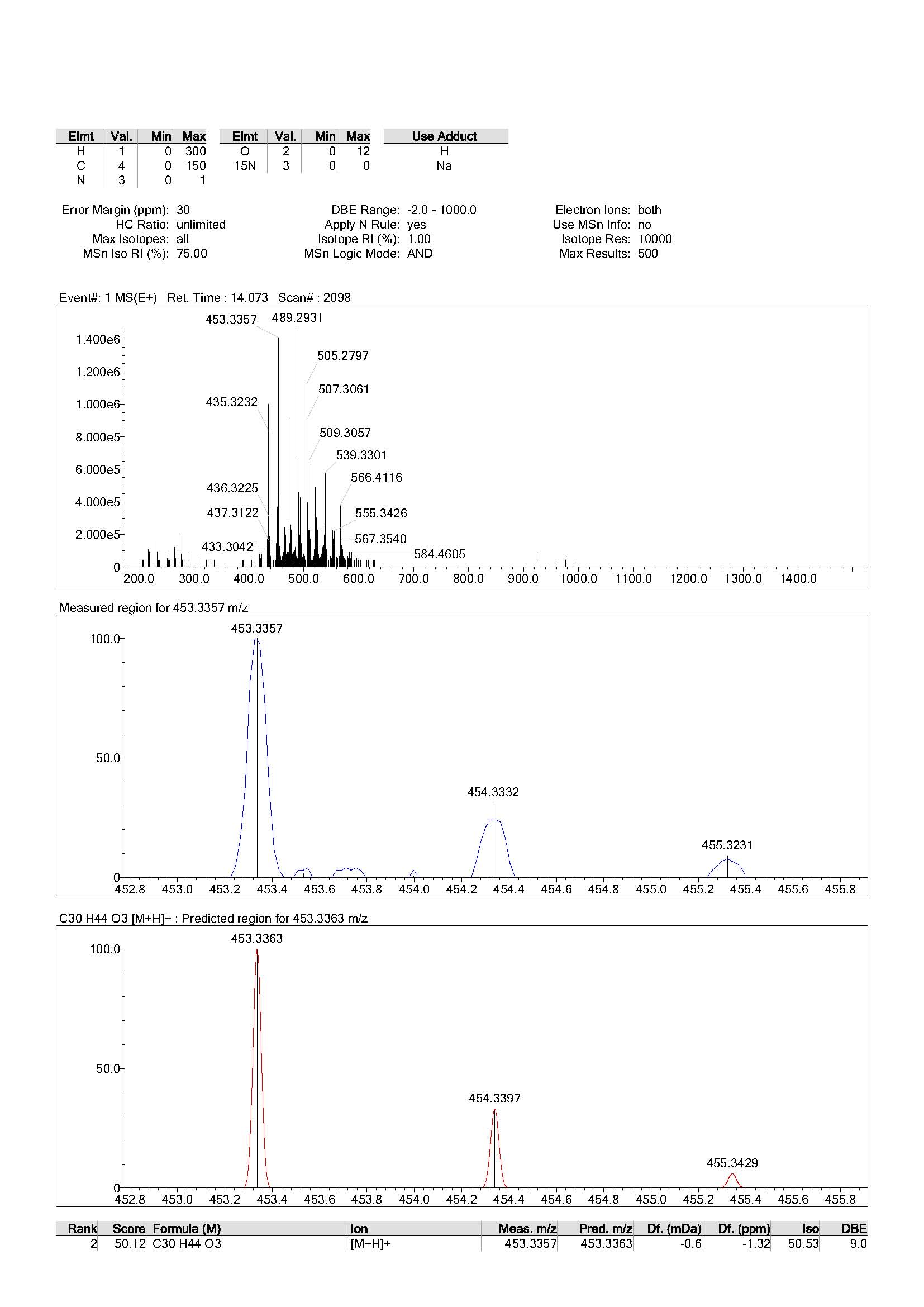


# S28. ^1^H NMR (800 MHz, CDCl_3_) of compound 4


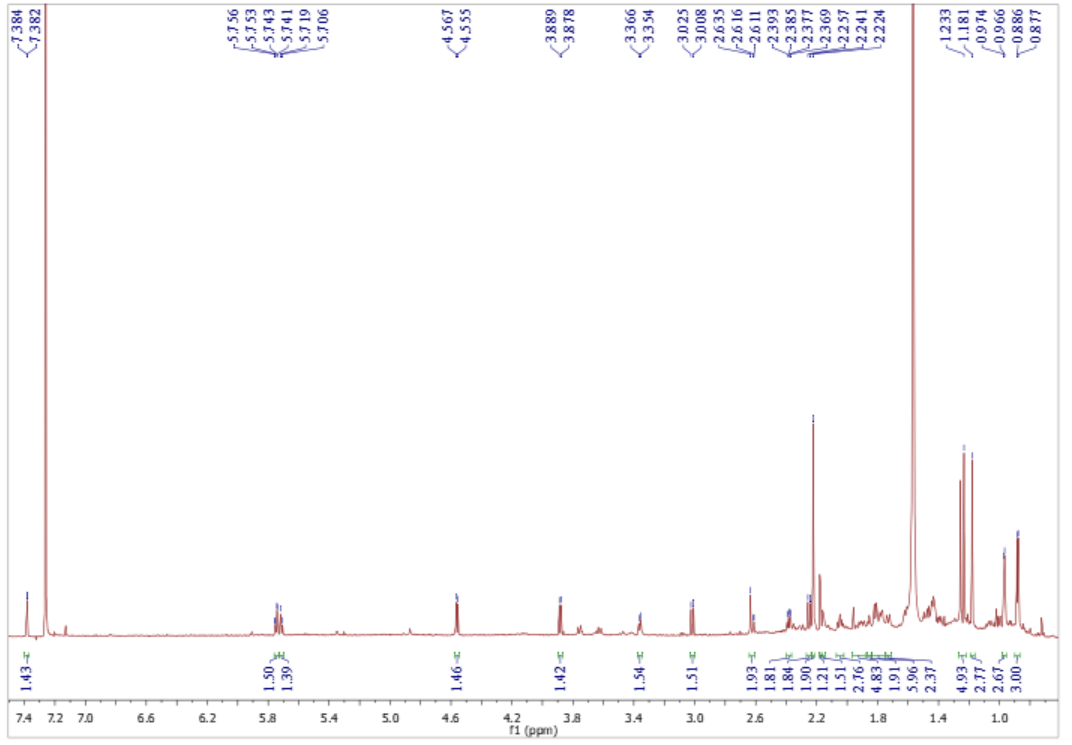


# S29. ^13^C NMR (DEPT) (200 MHz, CDCl_3_) of compound 4


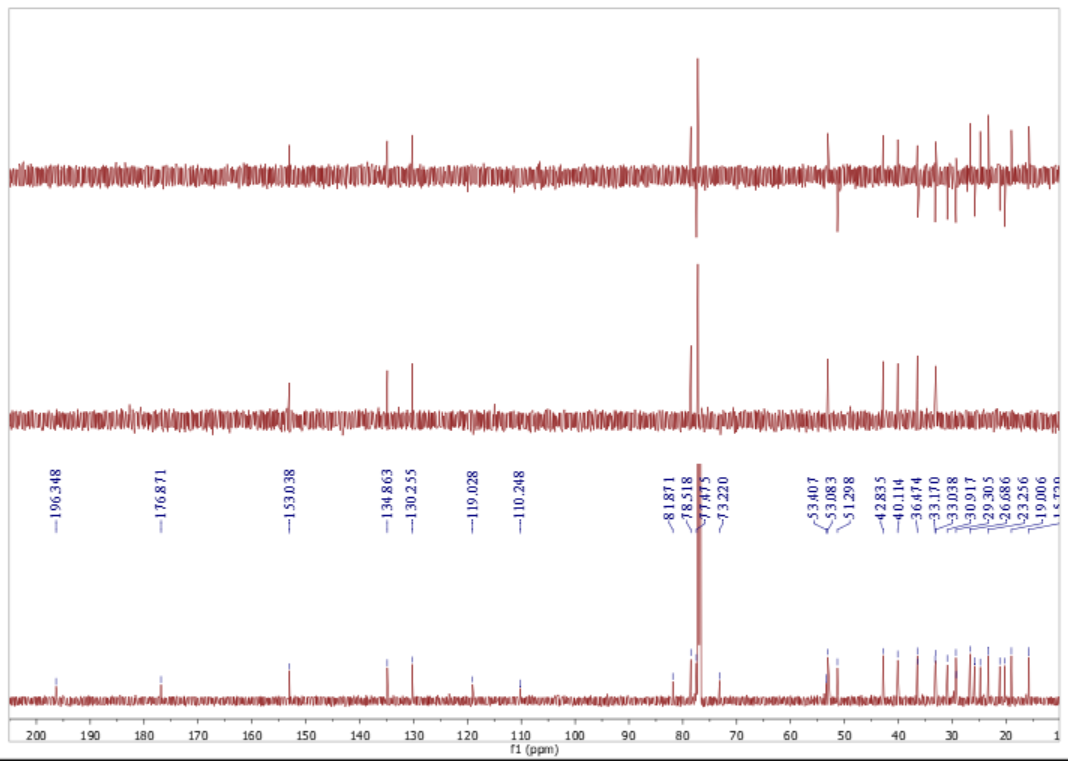


# S30. ^1^H-^1^H COSY (800 MHz, CDCl_3_) of compound 4


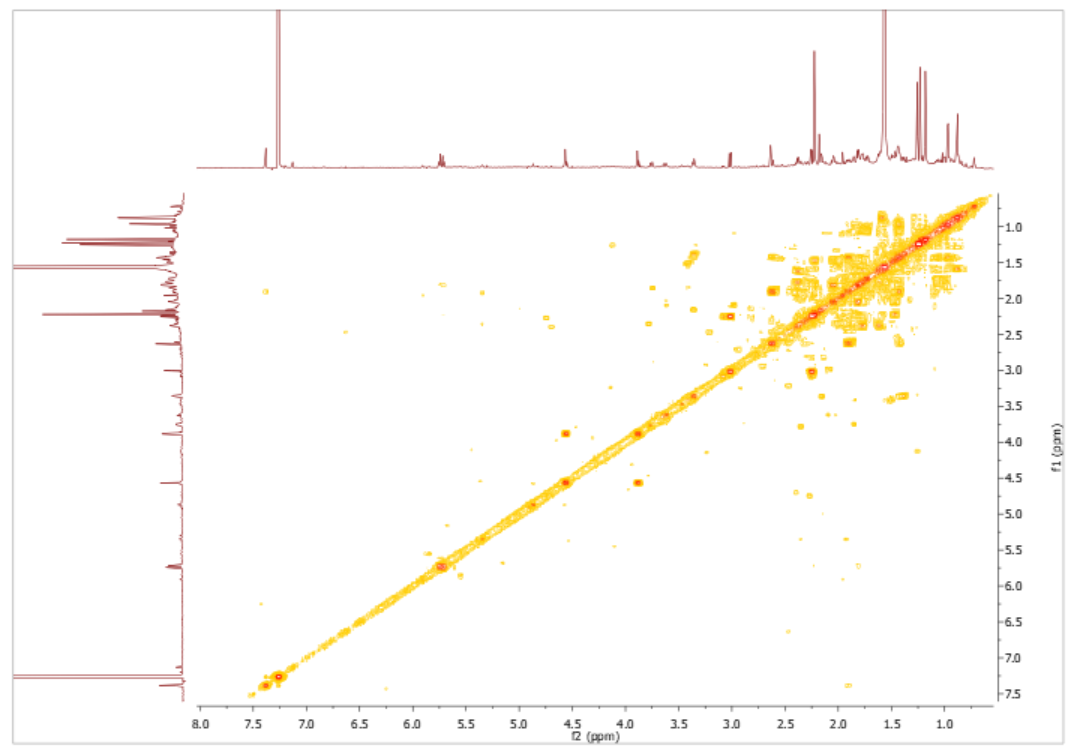


# S31. HSQC (800 MHz, CDCl_3_) of compound 4


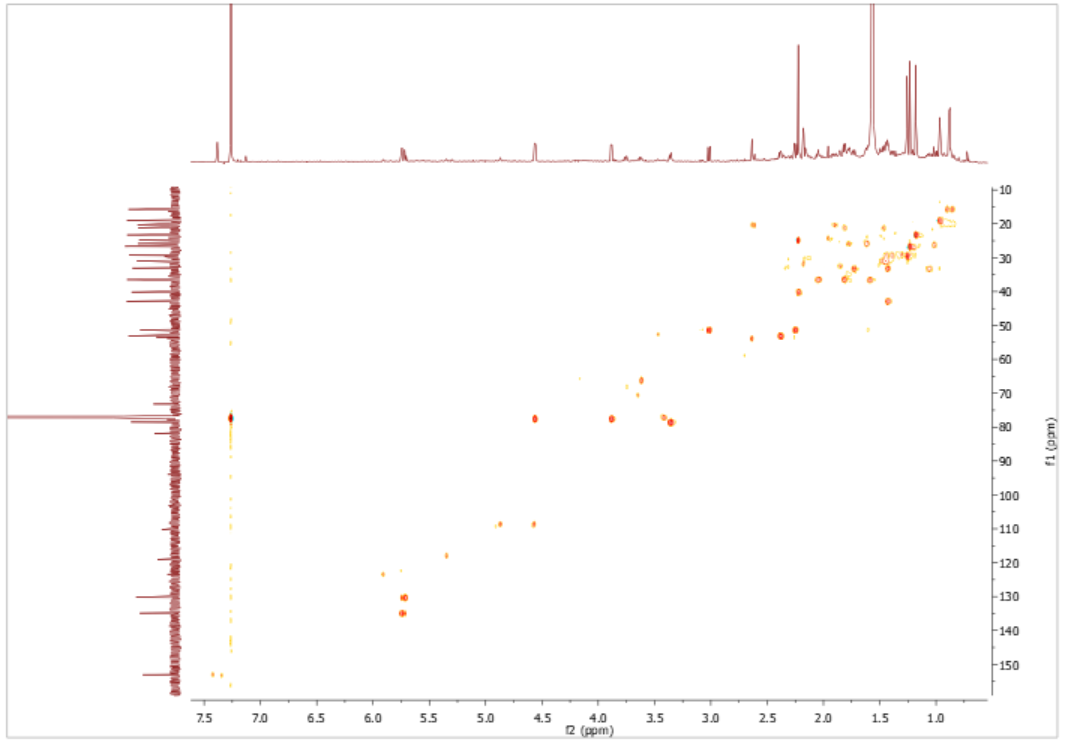


# S32. HMBC (800 MHz, CDCl_3_) of compound 4


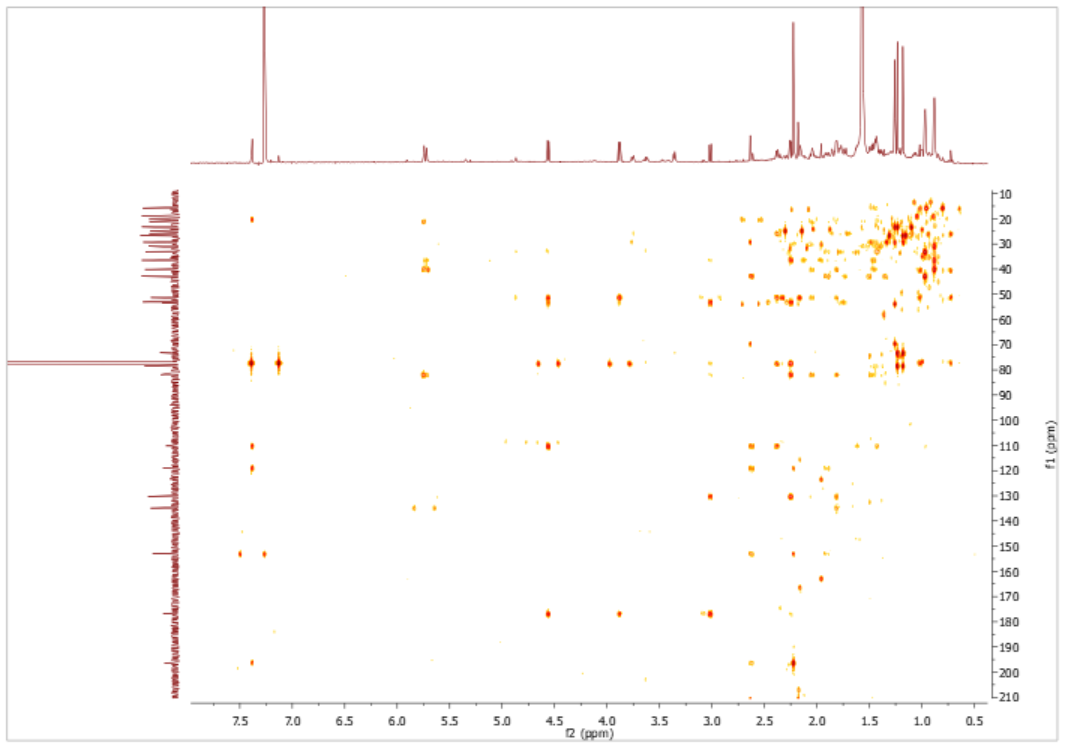


# S33. ROESY (800 MHz, CDCl_3_) of compound 4


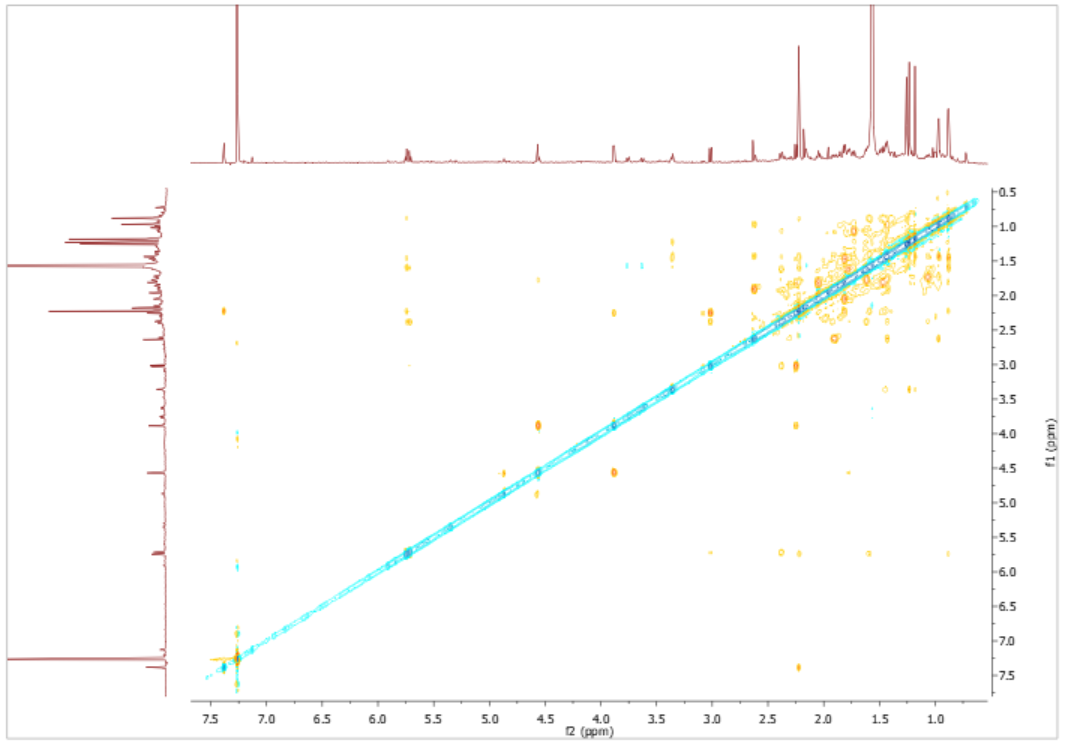


# S34. IR of compound 4


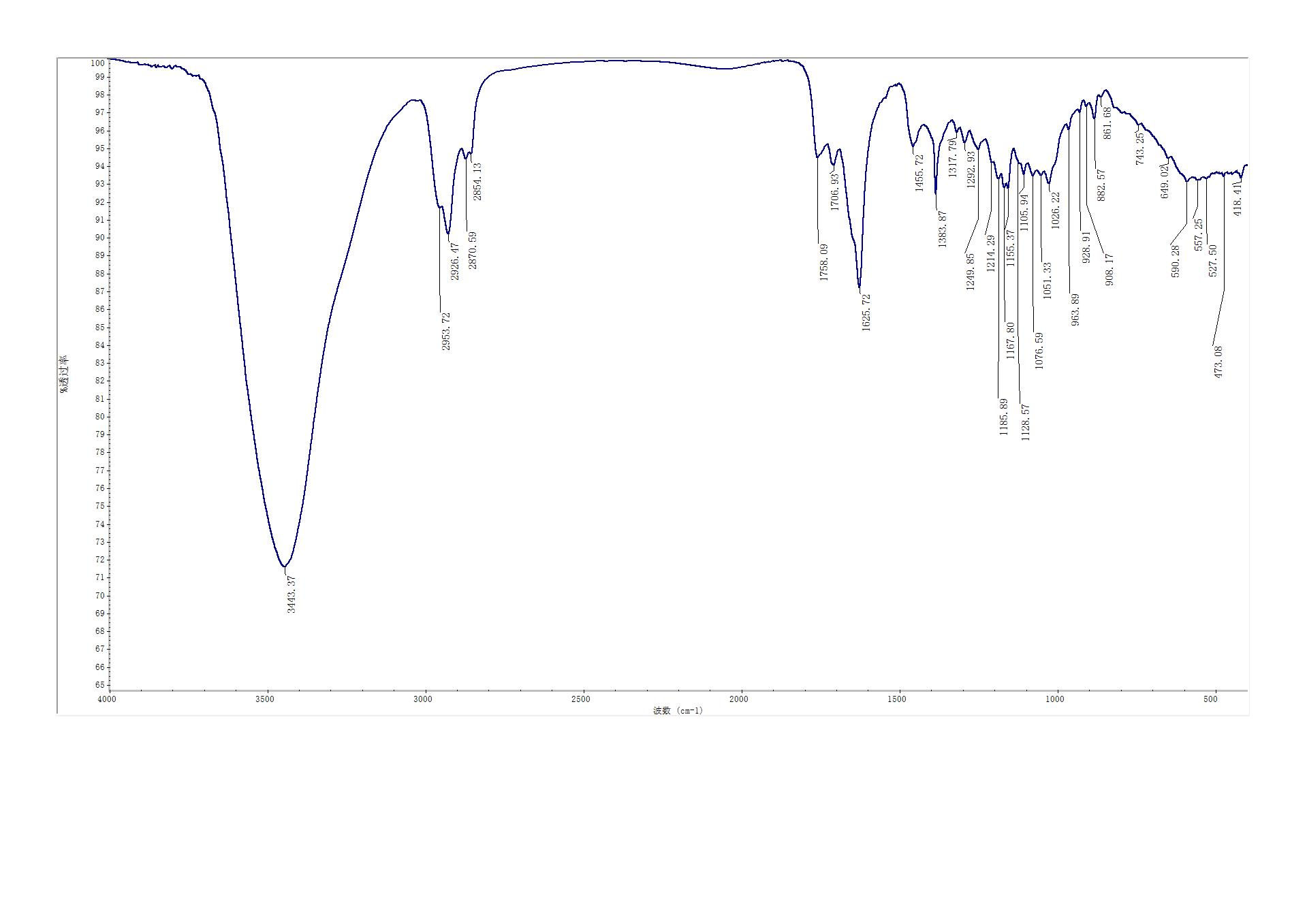


S35. ECD of compound **4**


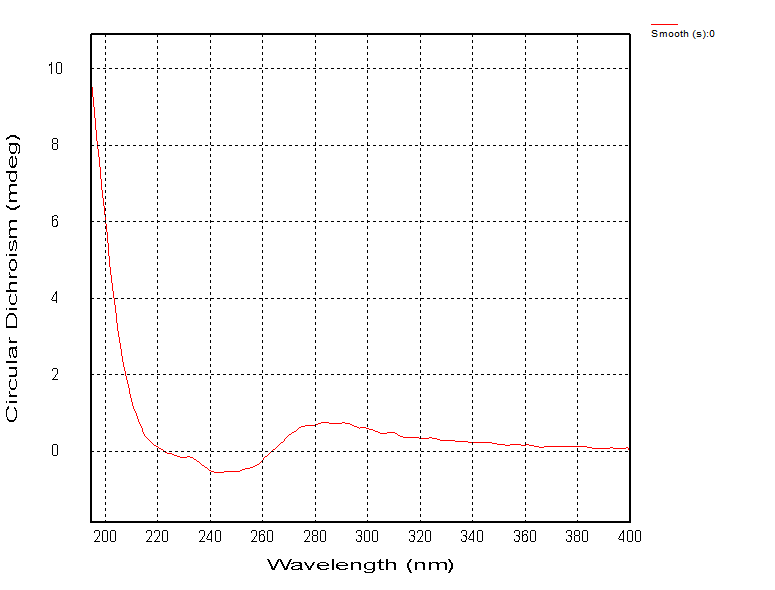


# S36. HRESIMS of compound 4


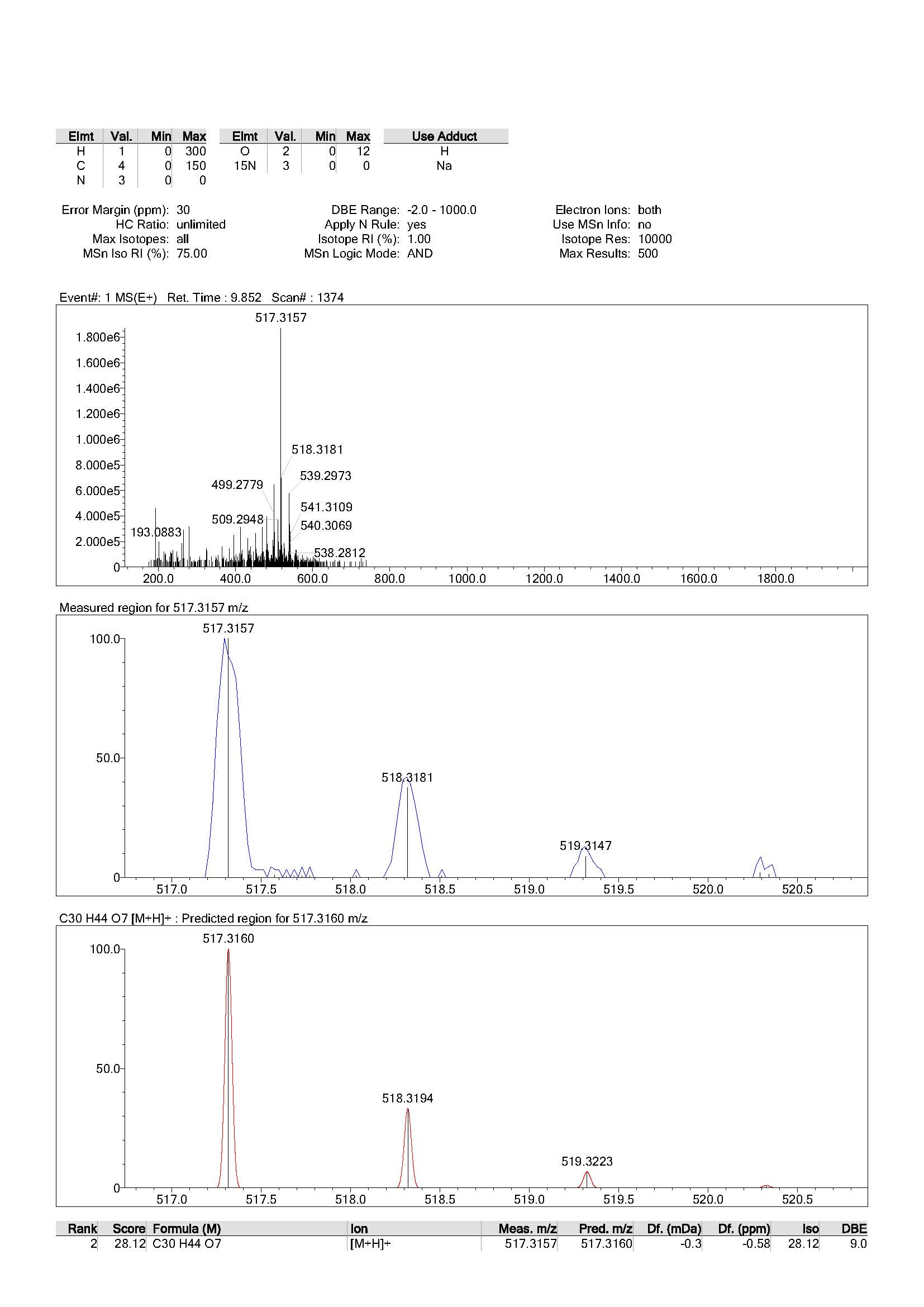


# S37. ^1^H NMR (800 MHz, CDCl_3_) of compound 5


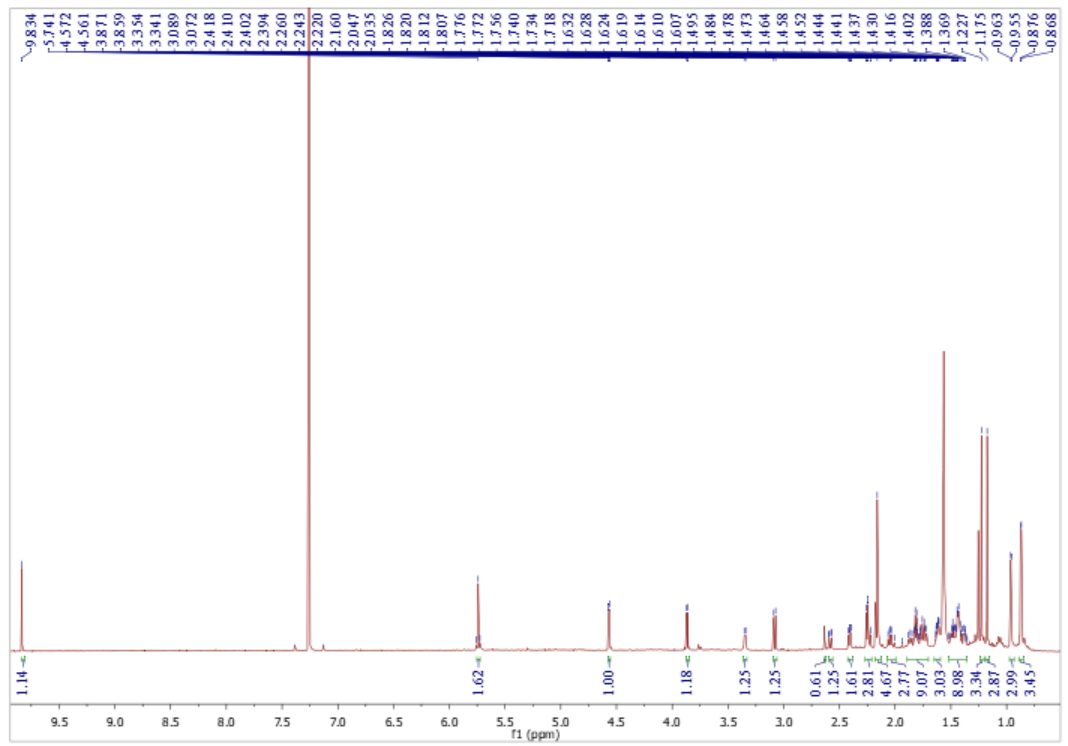


# S38. ^13^C NMR (DEPT) (200 MHz, CDCl_3_) of compound 5


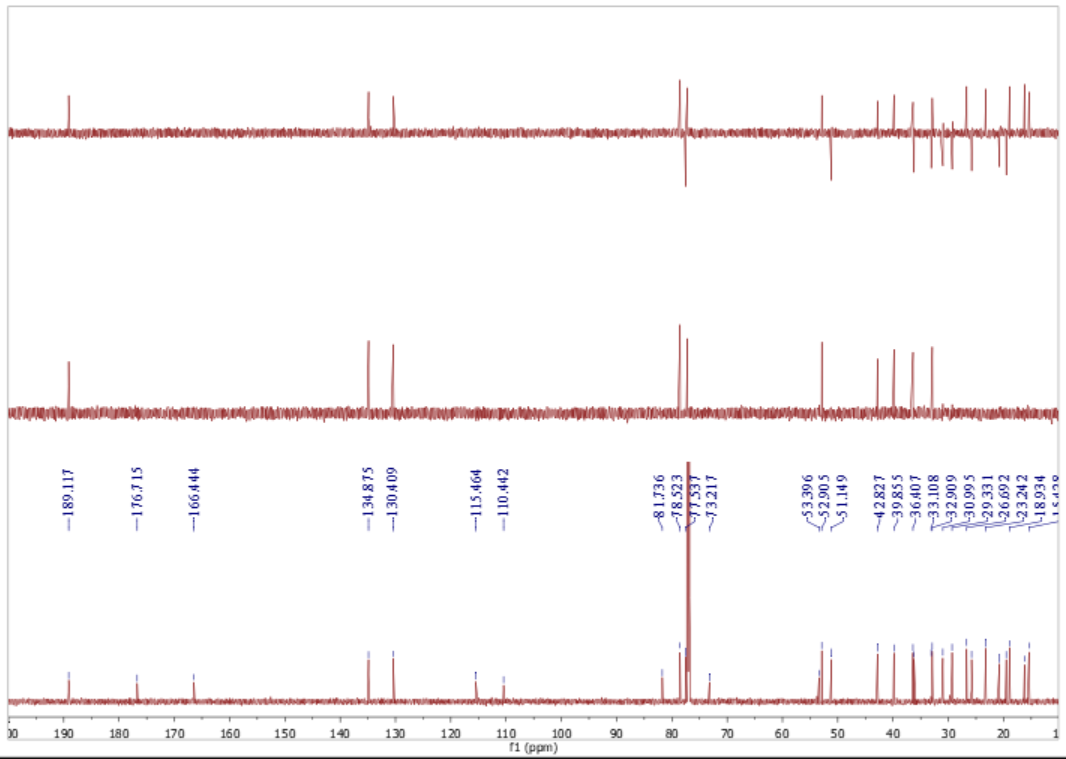


# S39. ^1^H-^1^H COSY (800 MHz, CDCl_3_) of compound 5


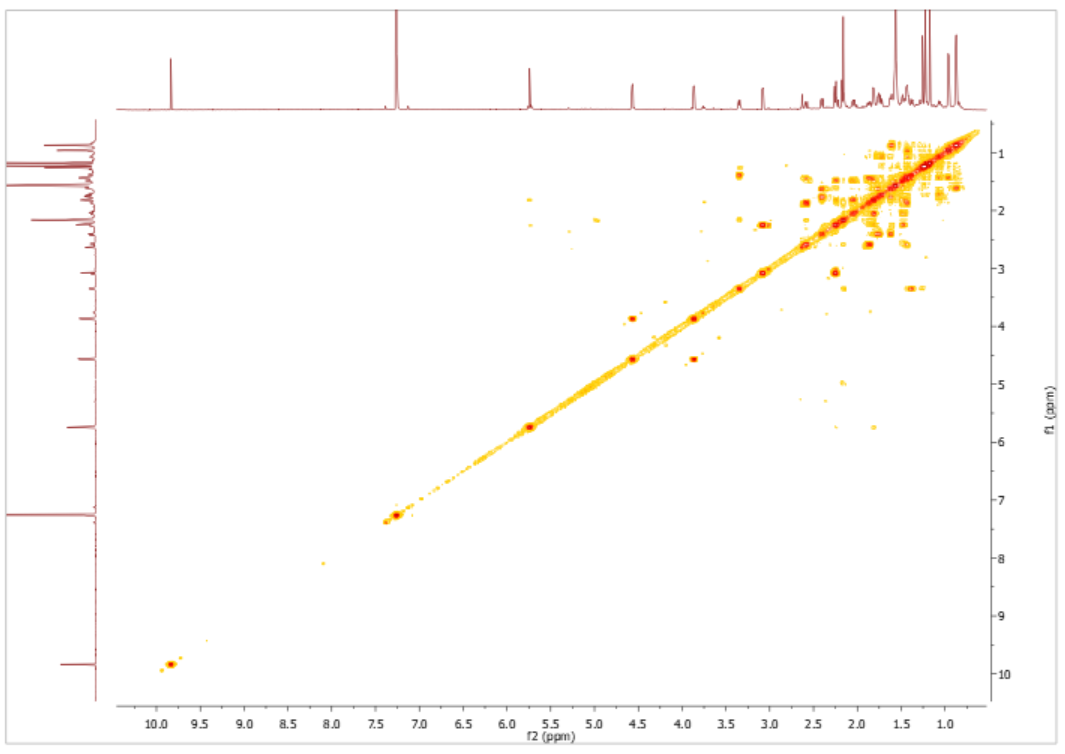


# S40. HSQC (800 MHz, CDCl_3_) of compound 5


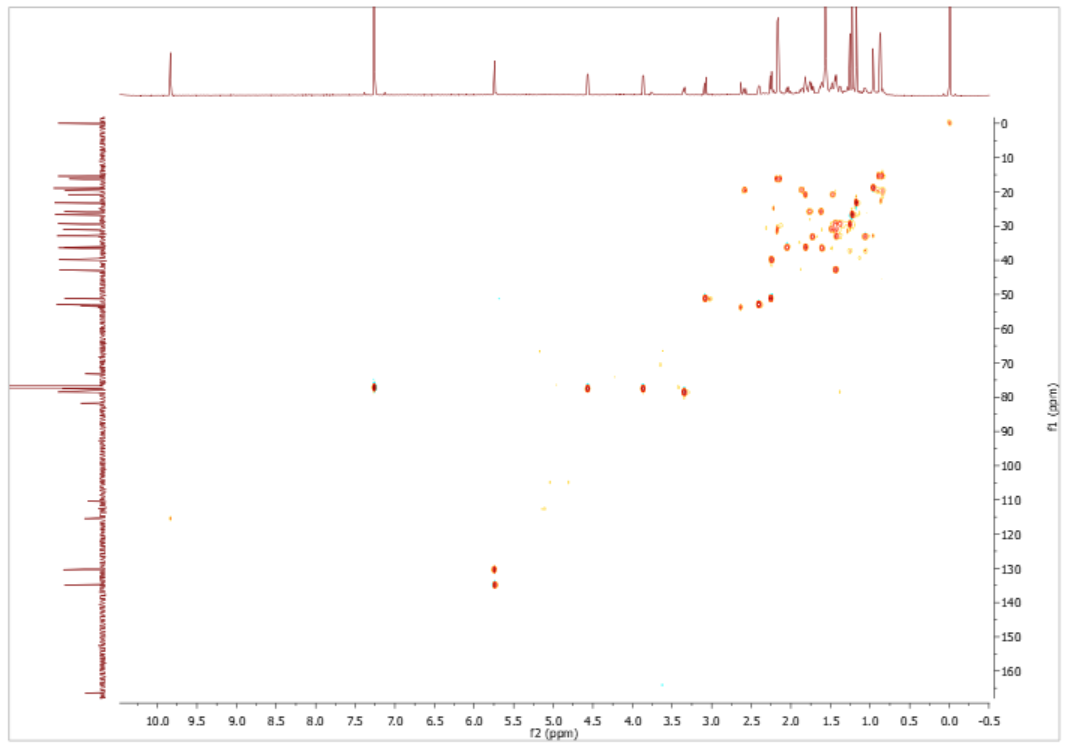


# S41. HMBC (800 MHz, CDCl_3_) of compound 5


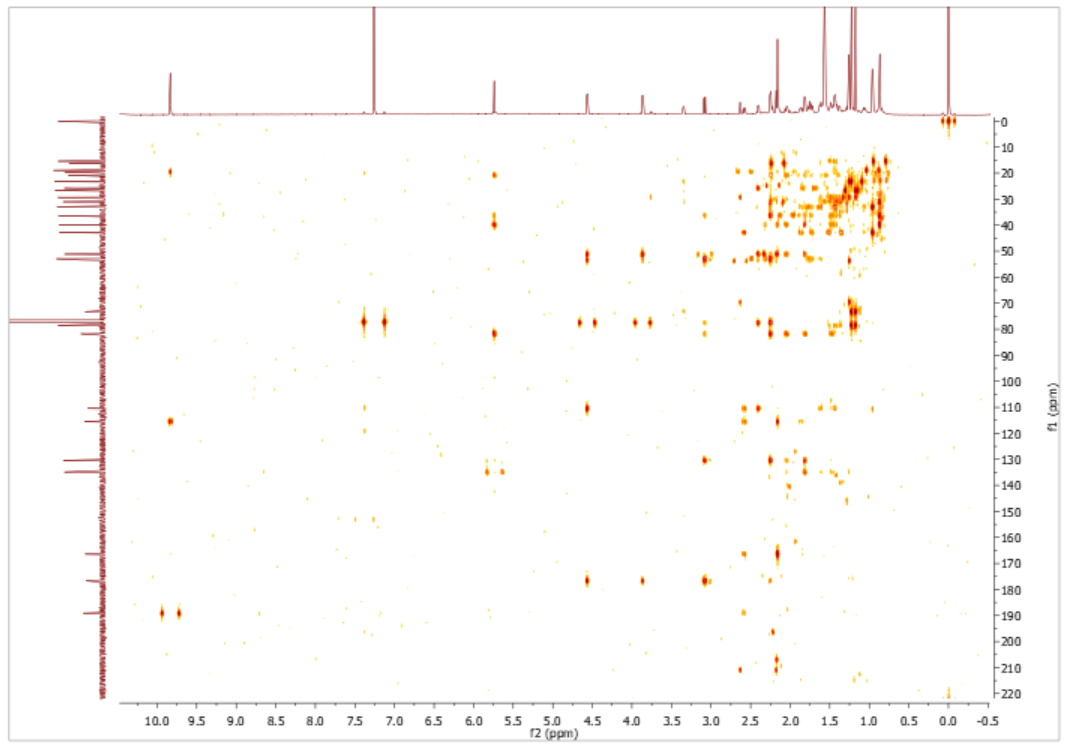


# S42. ROESY (800 MHz, CDCl_3_) of compound 5


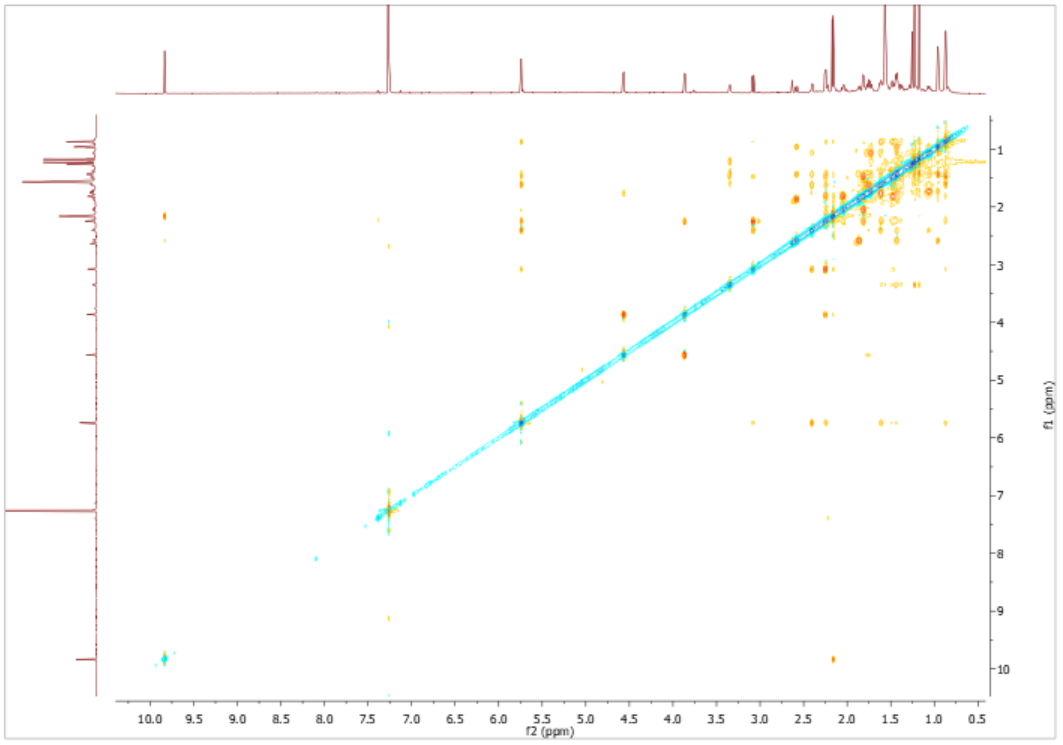


# S43. IR of compound 5


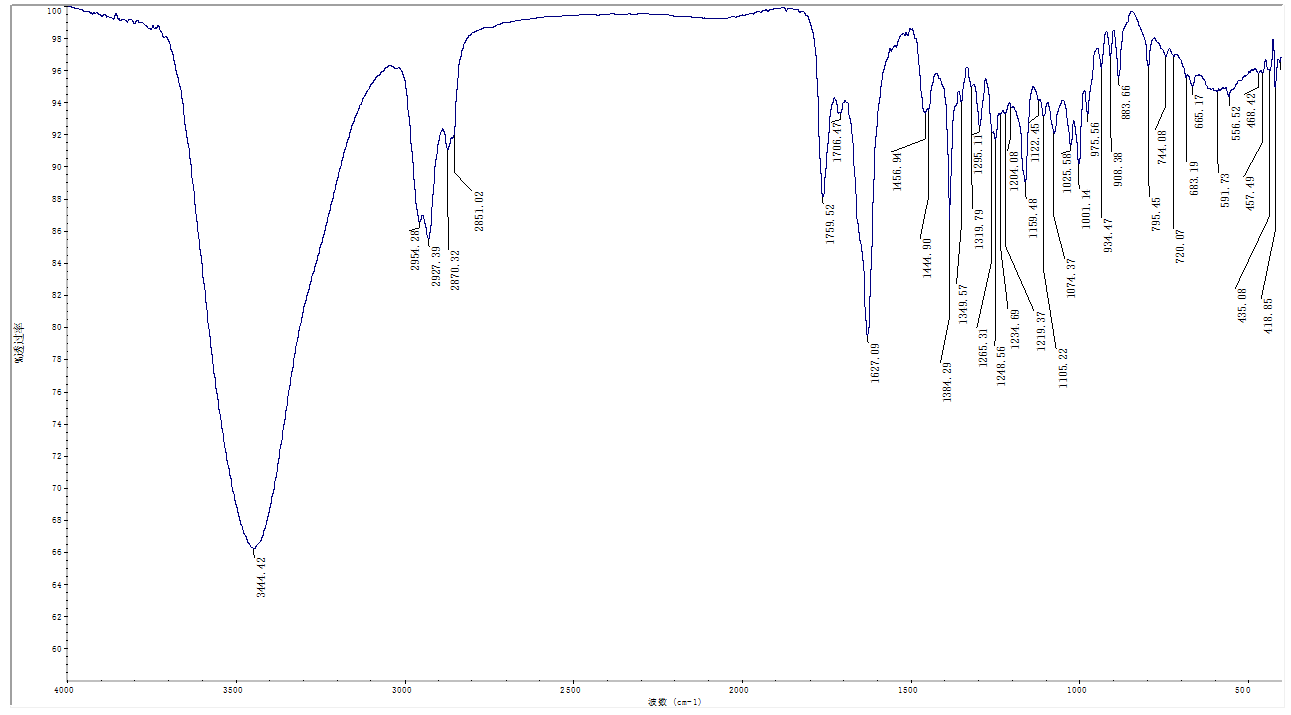


# S44. ECD of compound 5


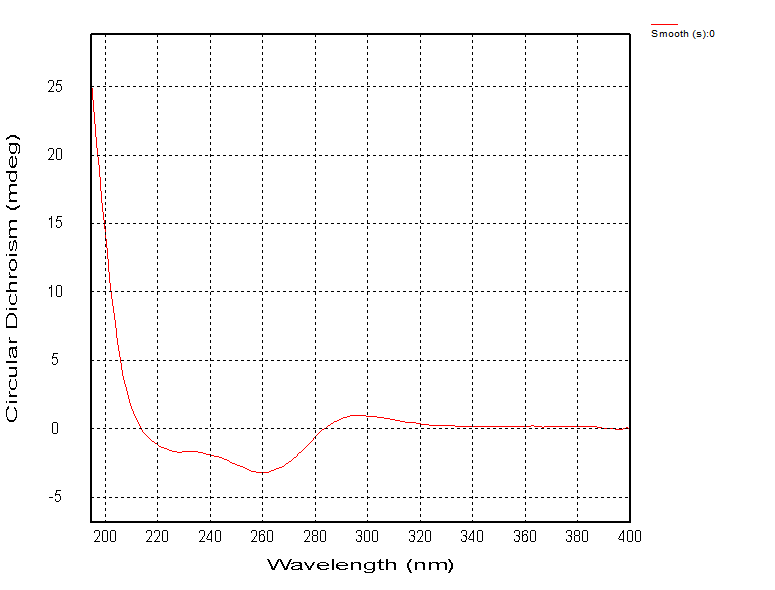


# S45. HRESIMS of compound 5


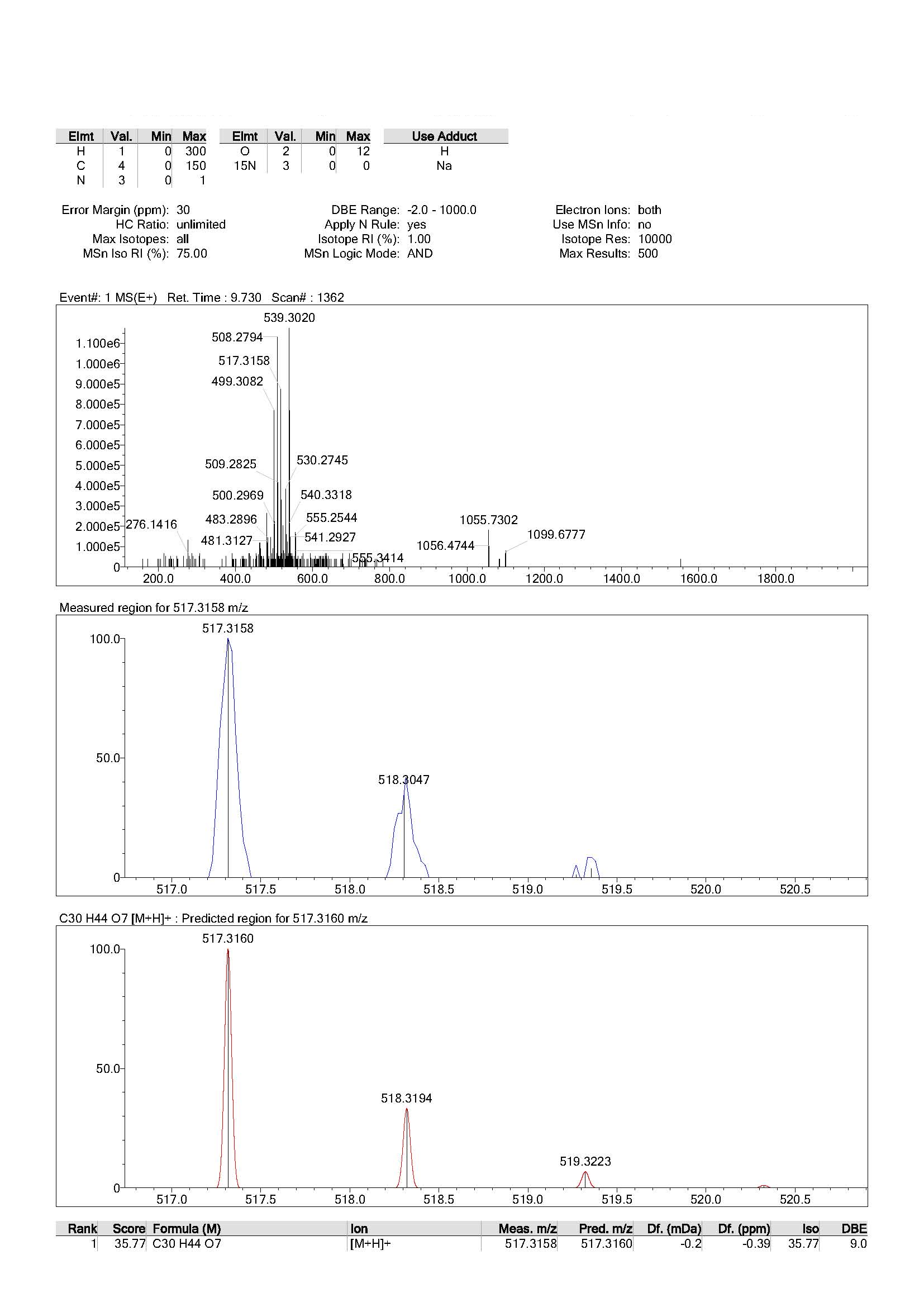


# S46. ^1^H NMR (600 MHz, CDCl_3_) of compound 6


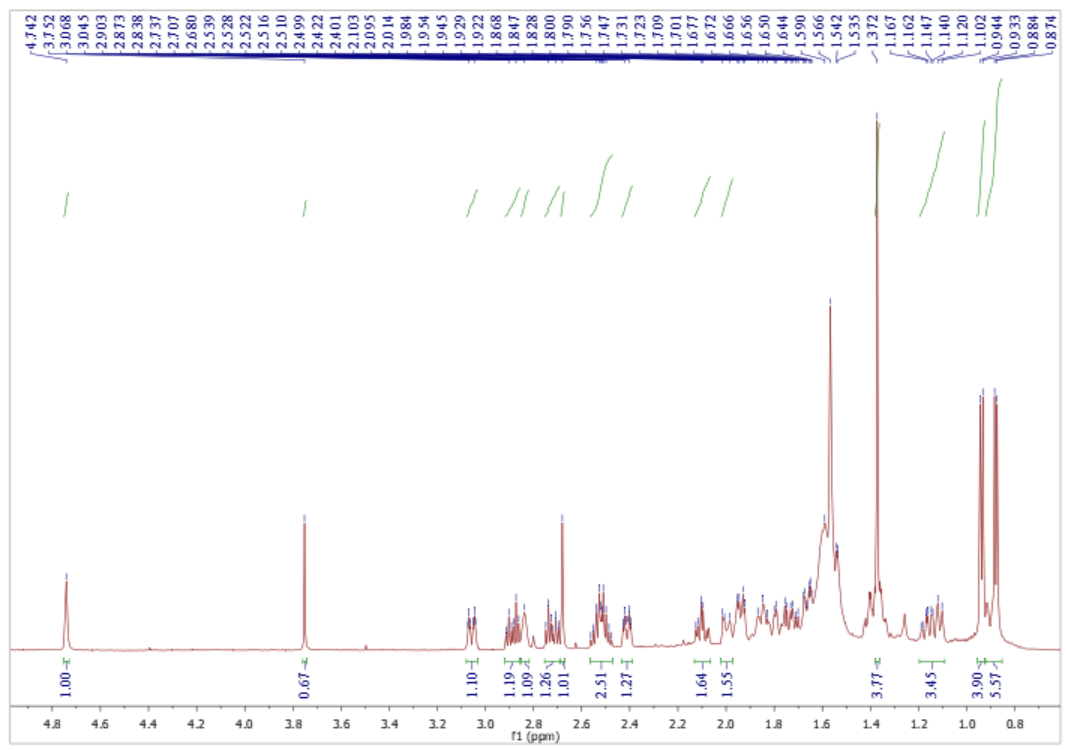


# S47. ^13^C NMR (DEPT) (150 MHz, CDCl_3_) of compound 6


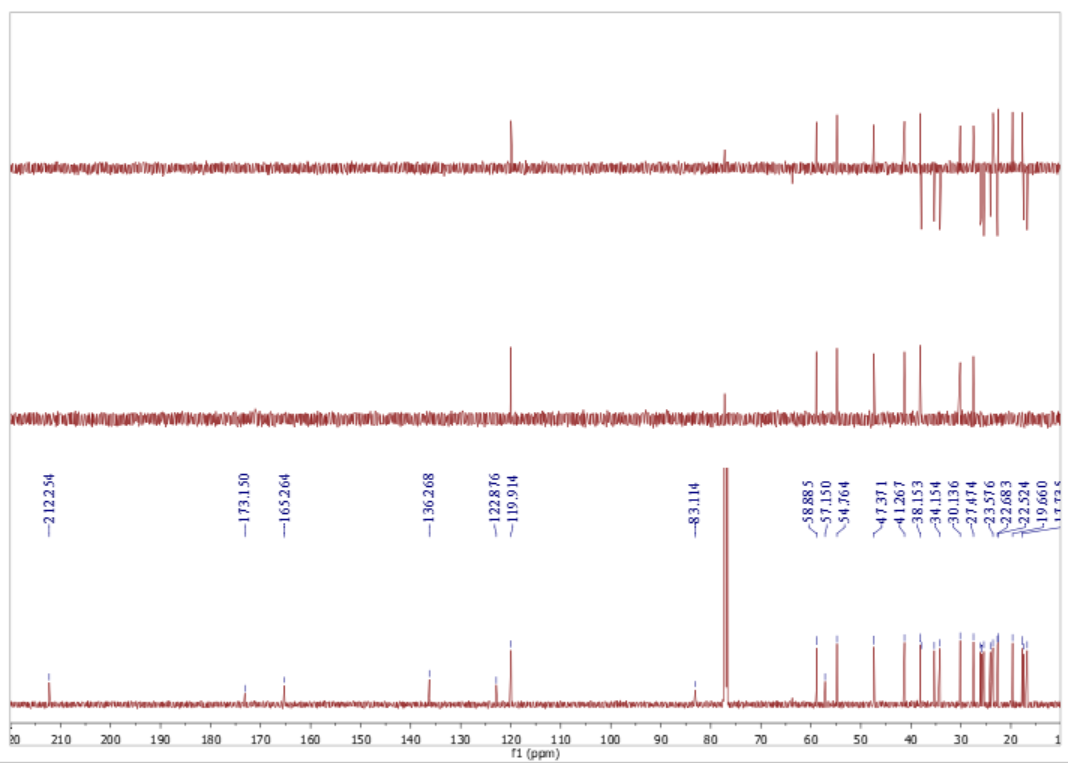


# S48. ^1^H-^1^H COSY (600 MHz, CDCl_3_) of compound 6


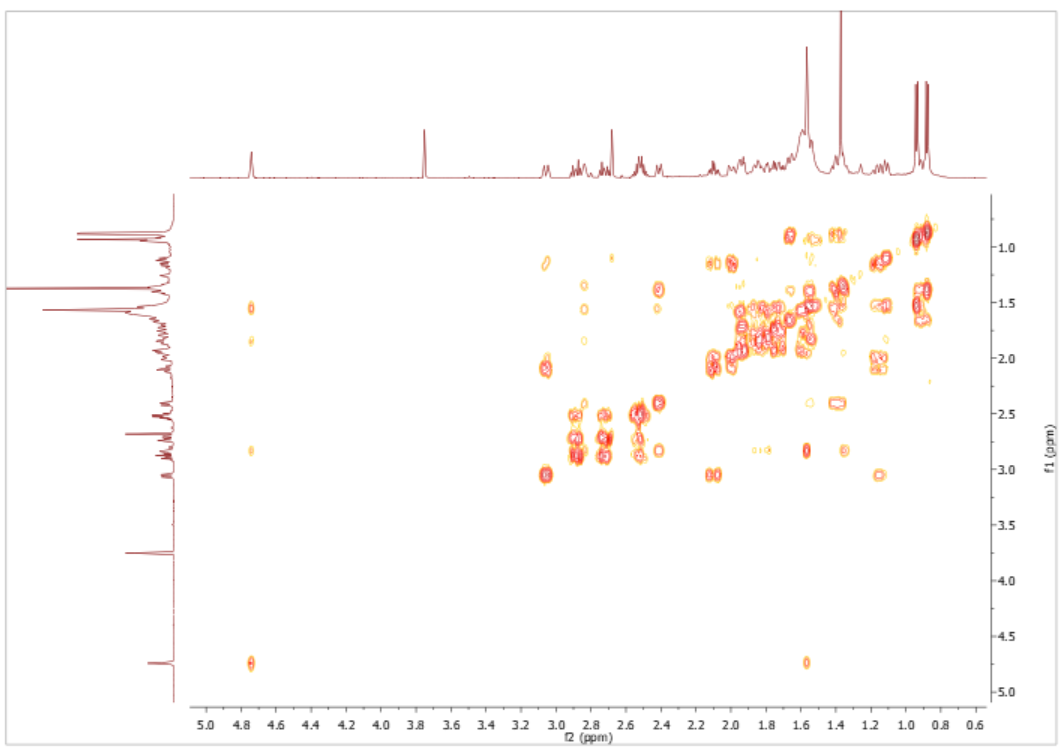


# S49. HSQC (600 MHz, CDCl_3_) of compound 6


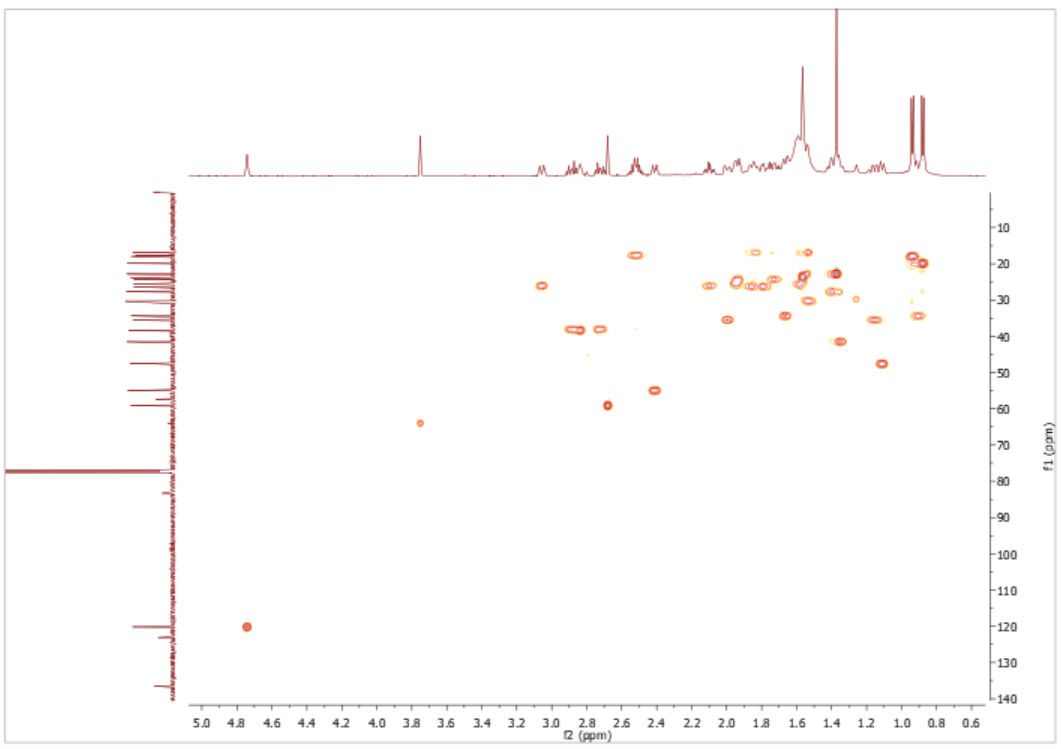


# S50. HMBC (600 MHz, CDCl_3_) of compound 6


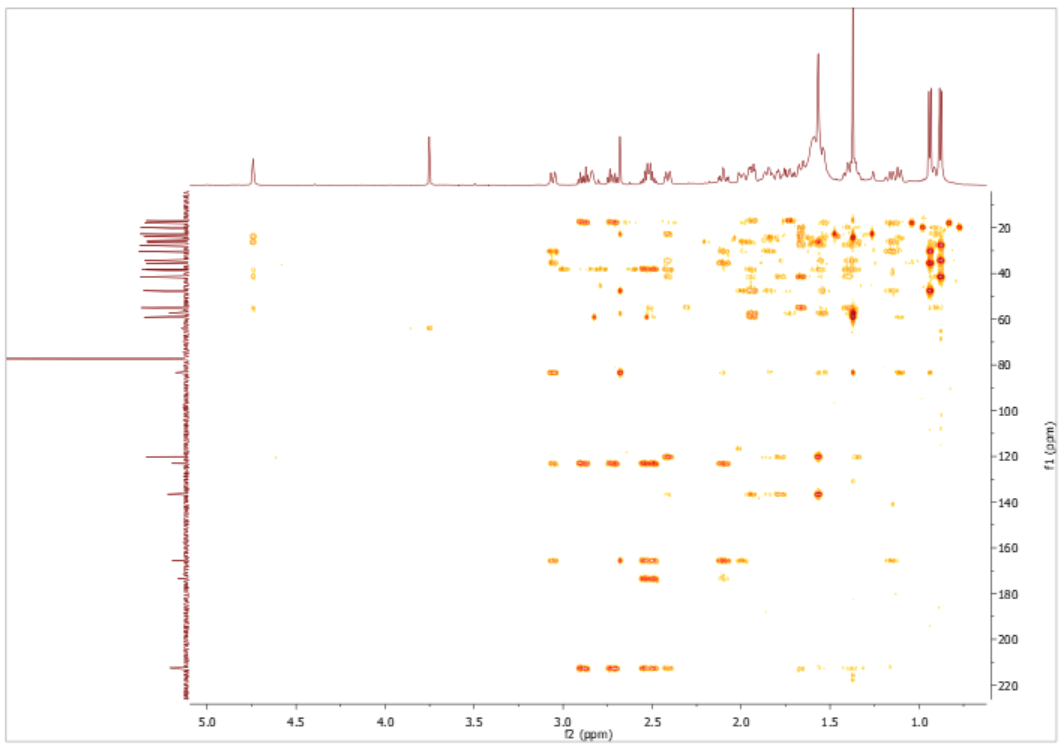


# S51. ROESY (600 MHz, CDCl_3_) of compound 6


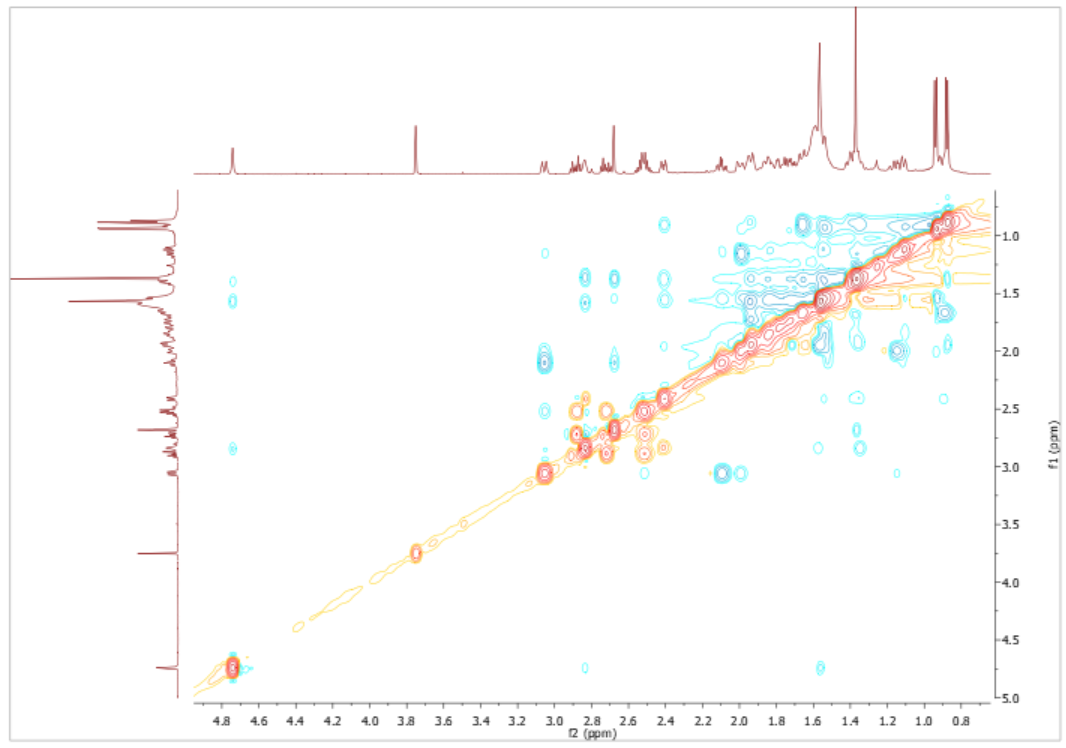


# S52. IR of compound 6


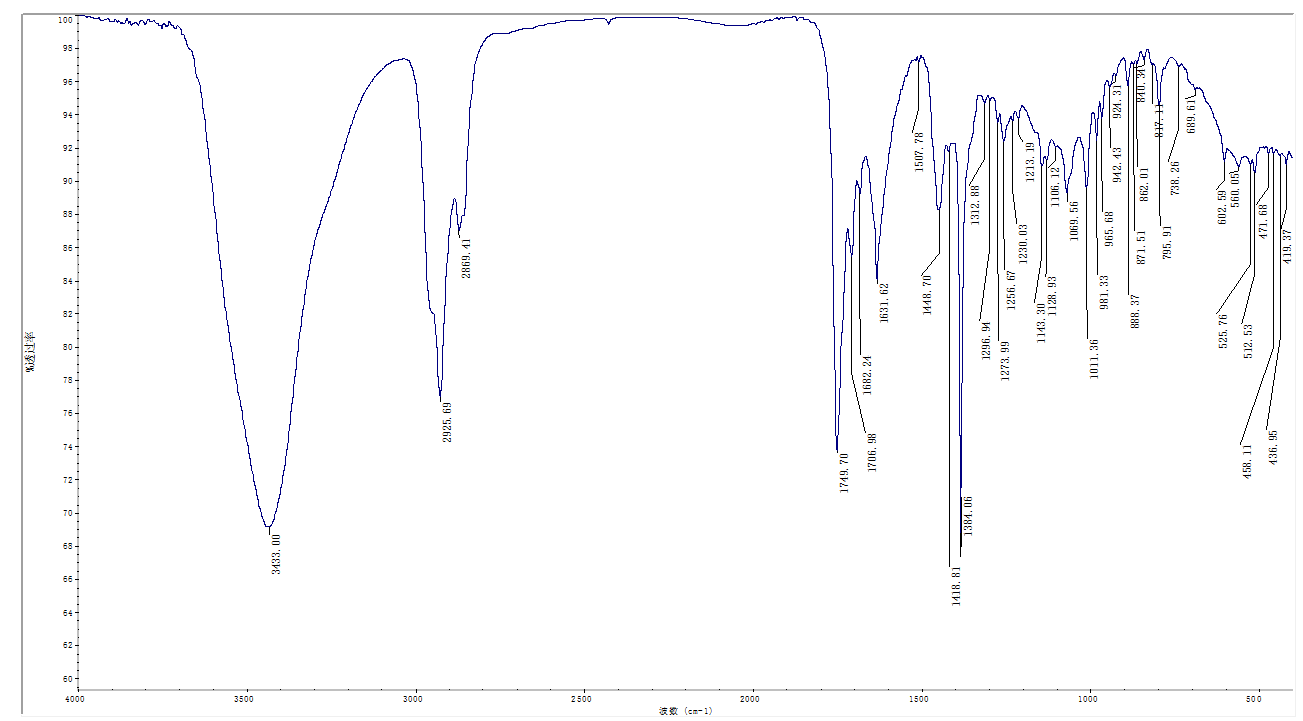


# S53. ECD of compound 6


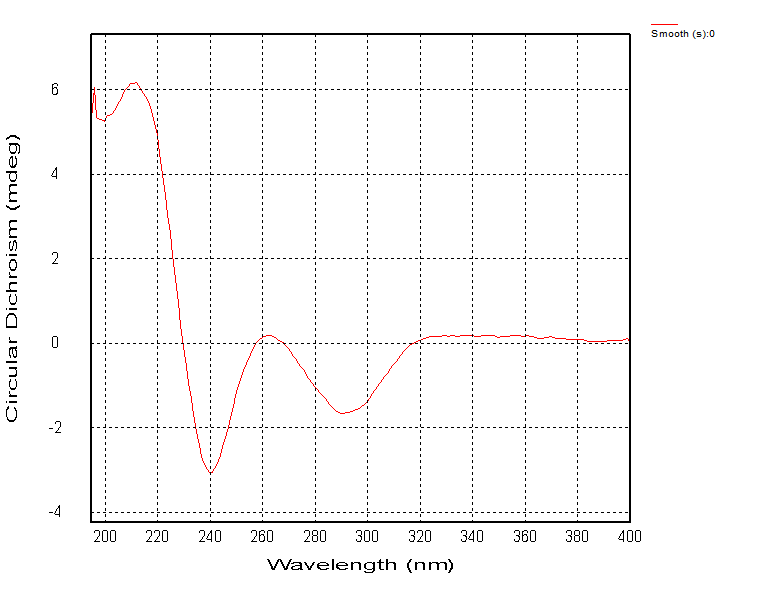


# S54. HRESIMS of compound 6


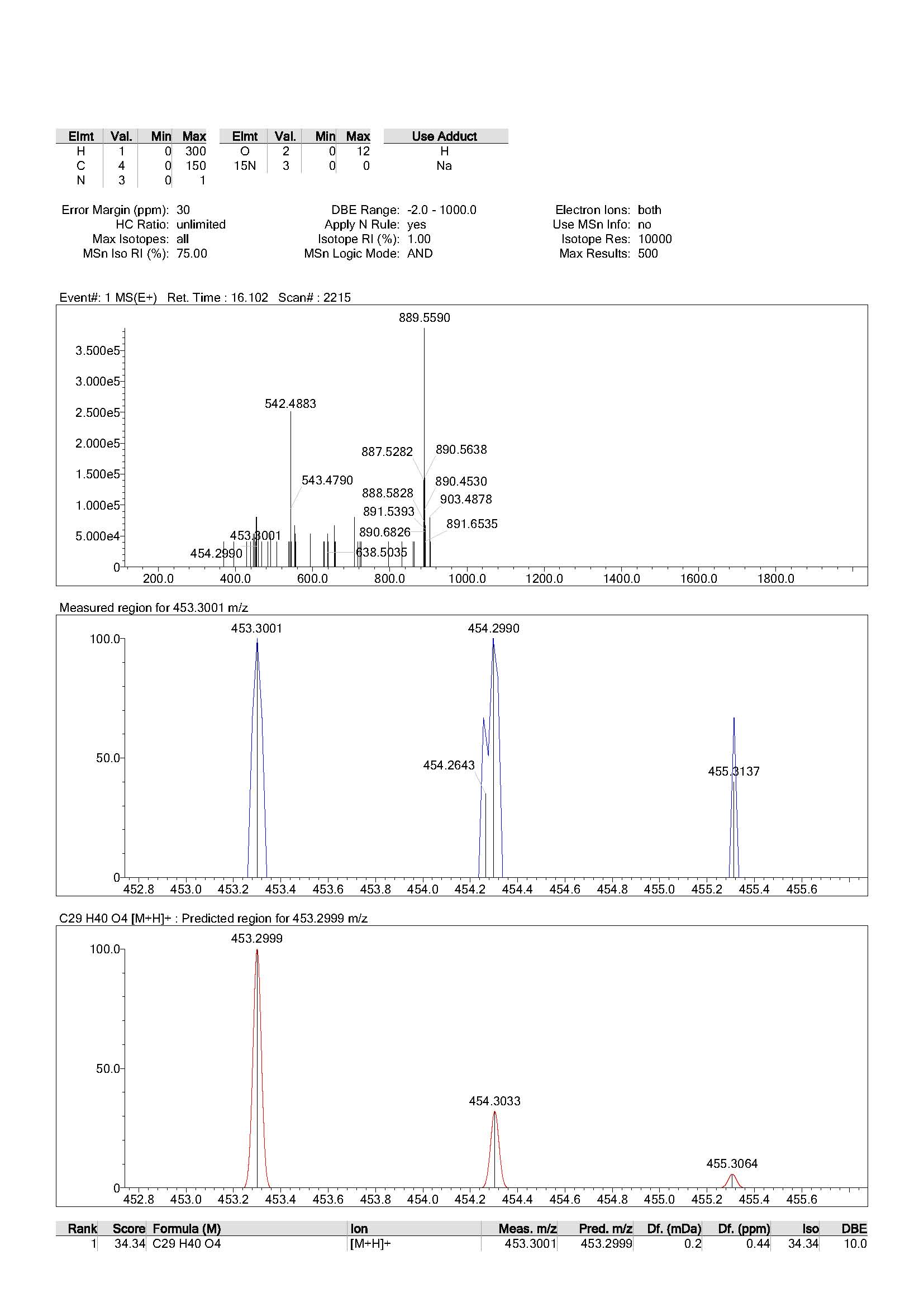


# S55. ^1^H NMR (600 MHz, CDCl_3_) of compound 7


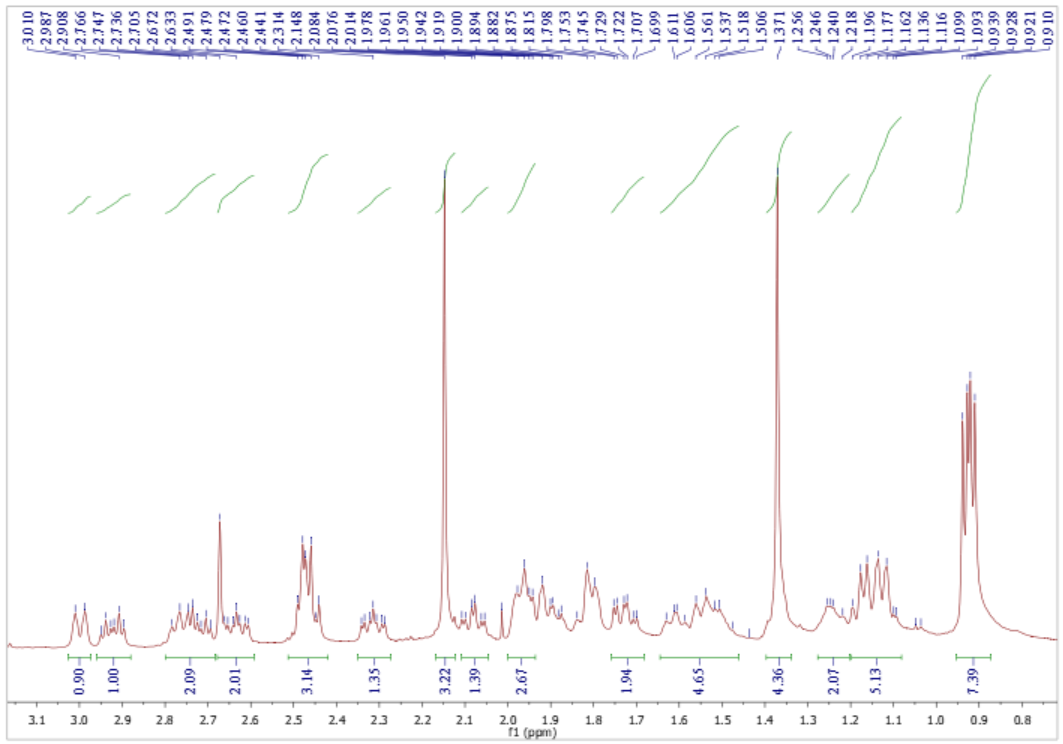


S56. ^13^C NMR (DEPT) (150 MHz, CDCl_3_) of compound **7**


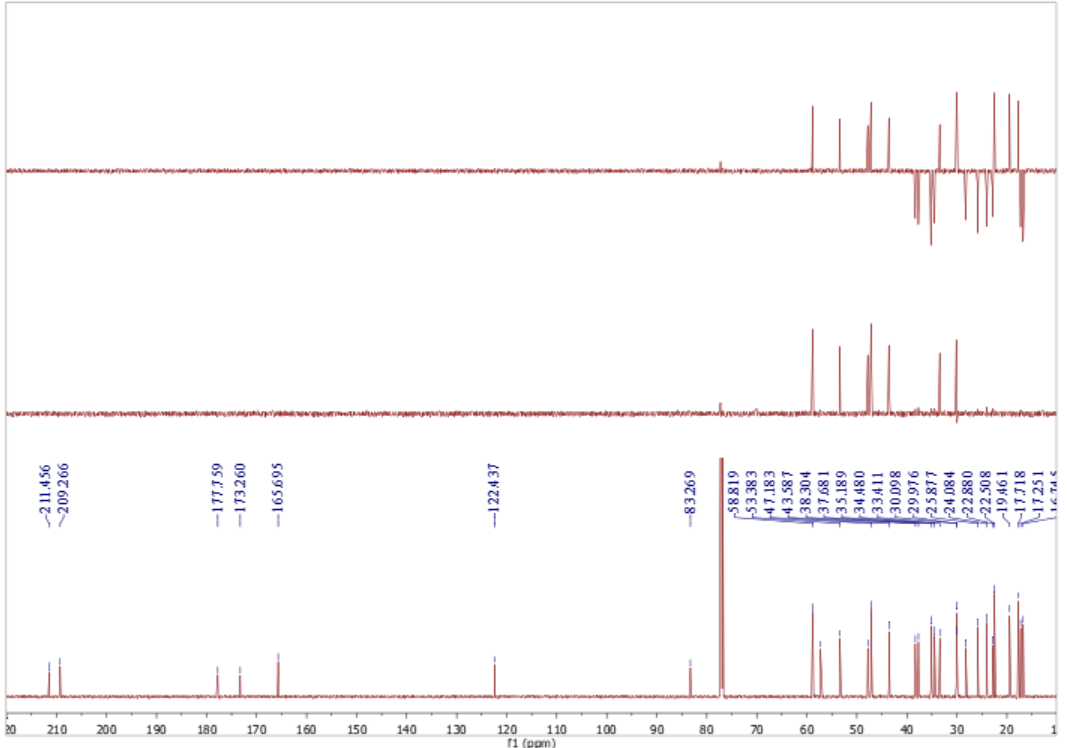


# S57. ^1^H-^1^H COSY (600 MHz, CDCl_3_) of compound 7


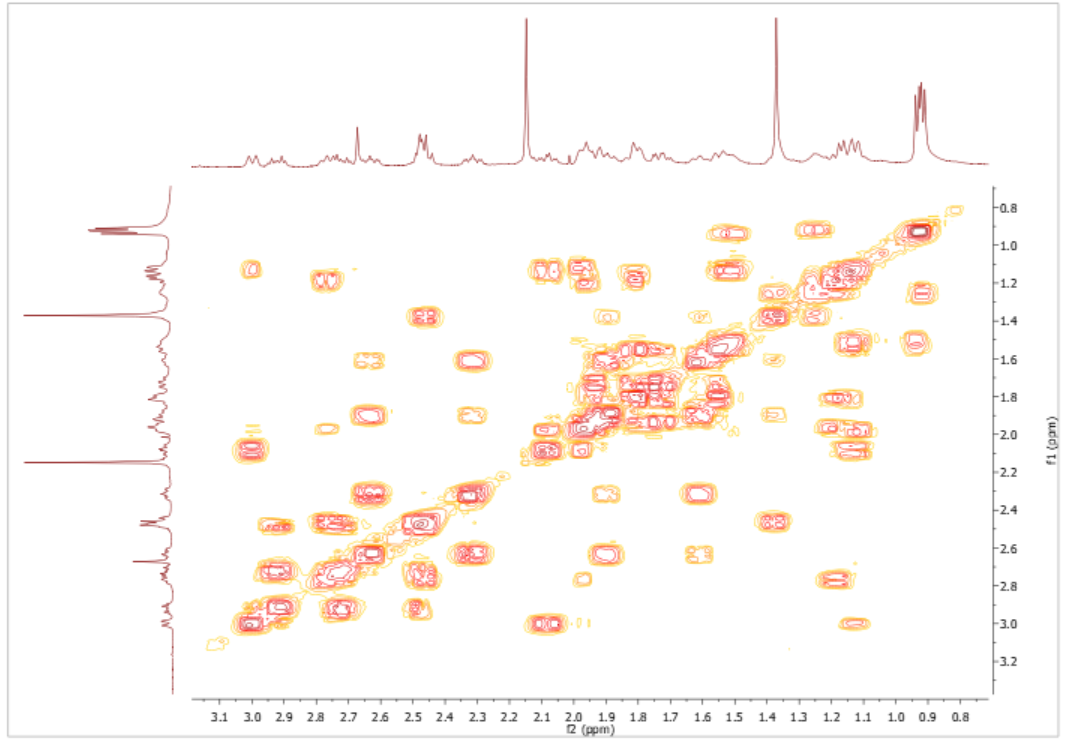


# S58. HSQC (600 MHz, CDCl_3_) of compound 7


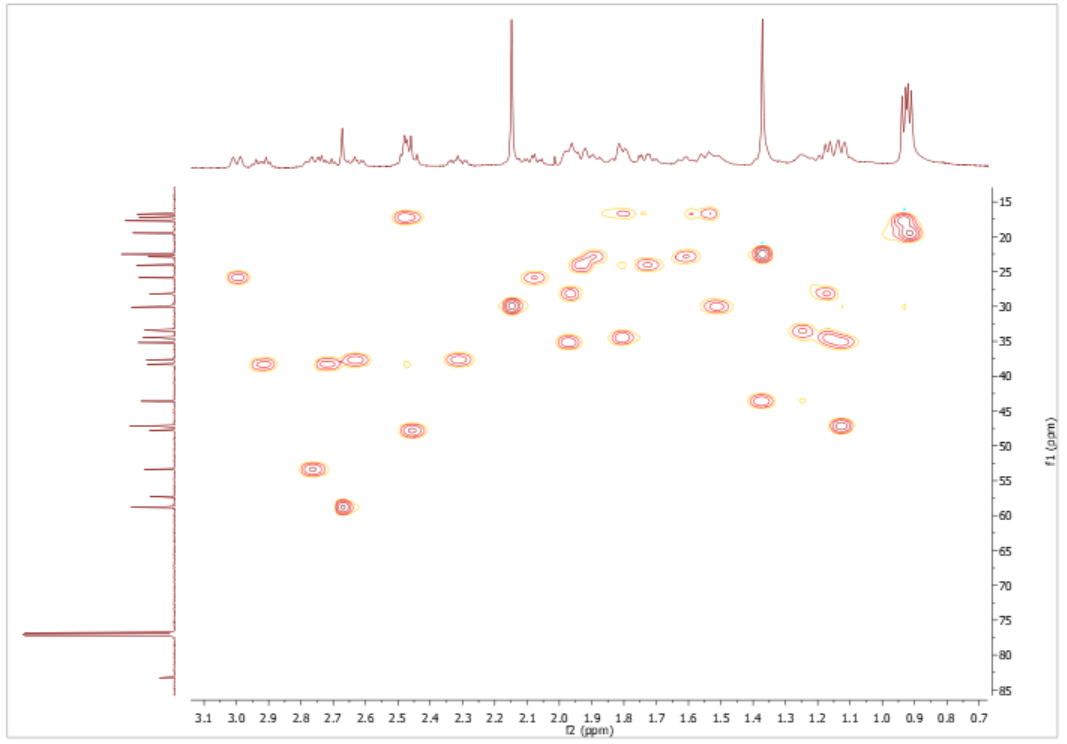


# S59. HMBC (600 MHz, CDCl_3_) of compound 7


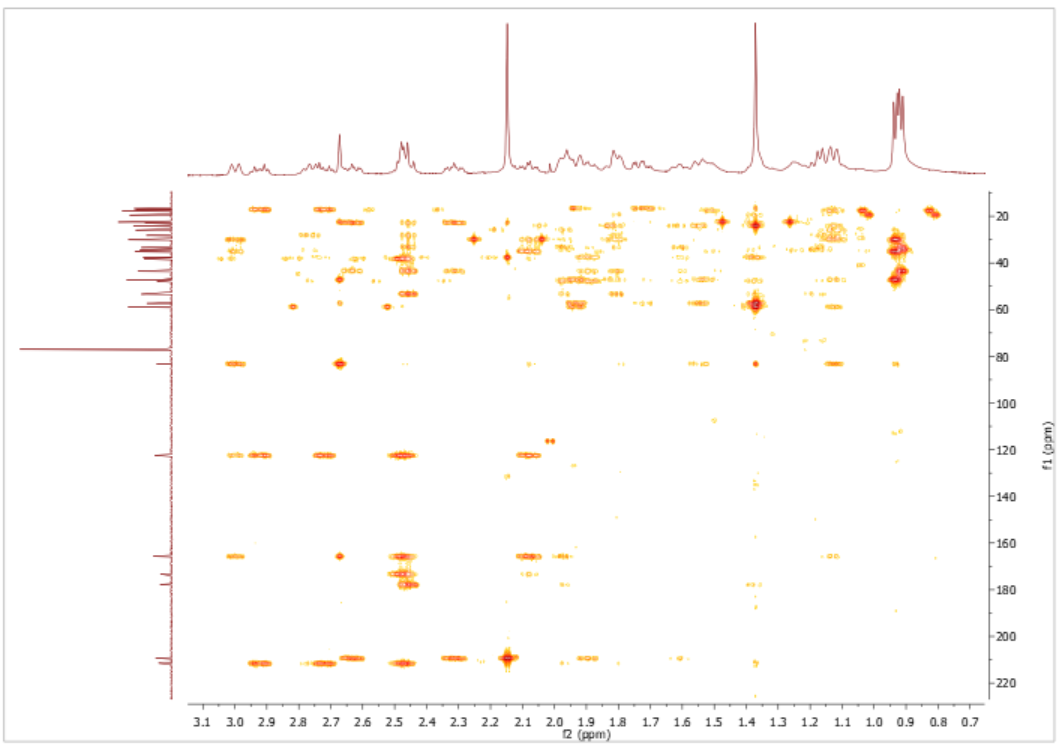


# S60. ROESY (600 MHz, CDCl_3_) of compound 7


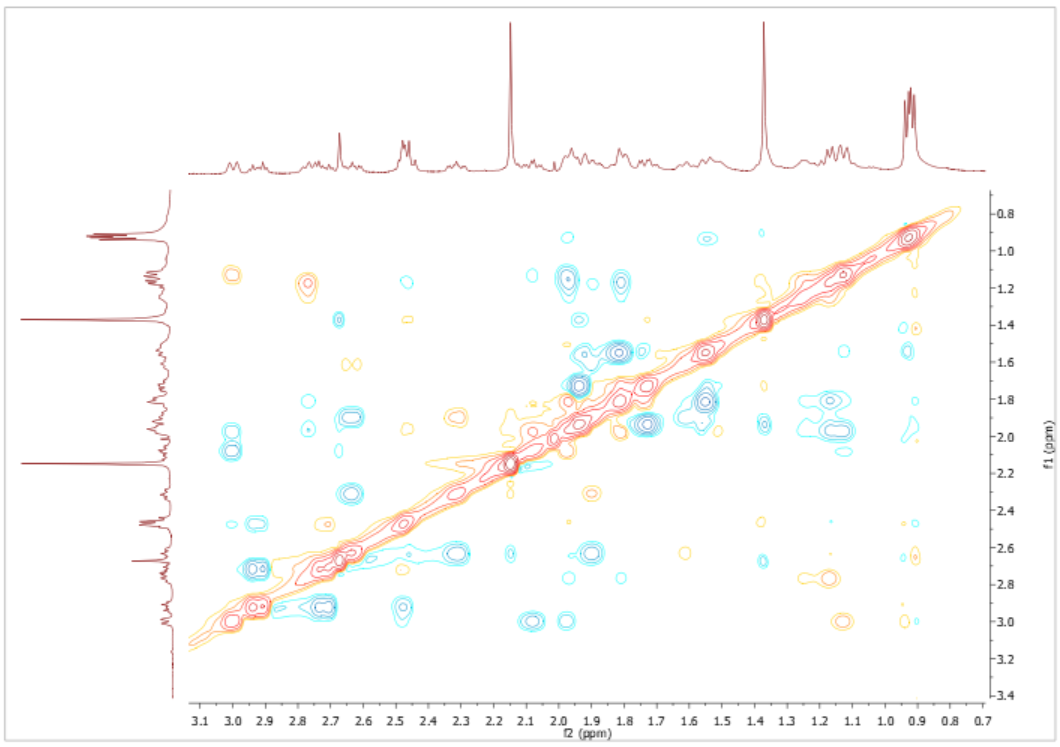


# S61. IR of compound 7


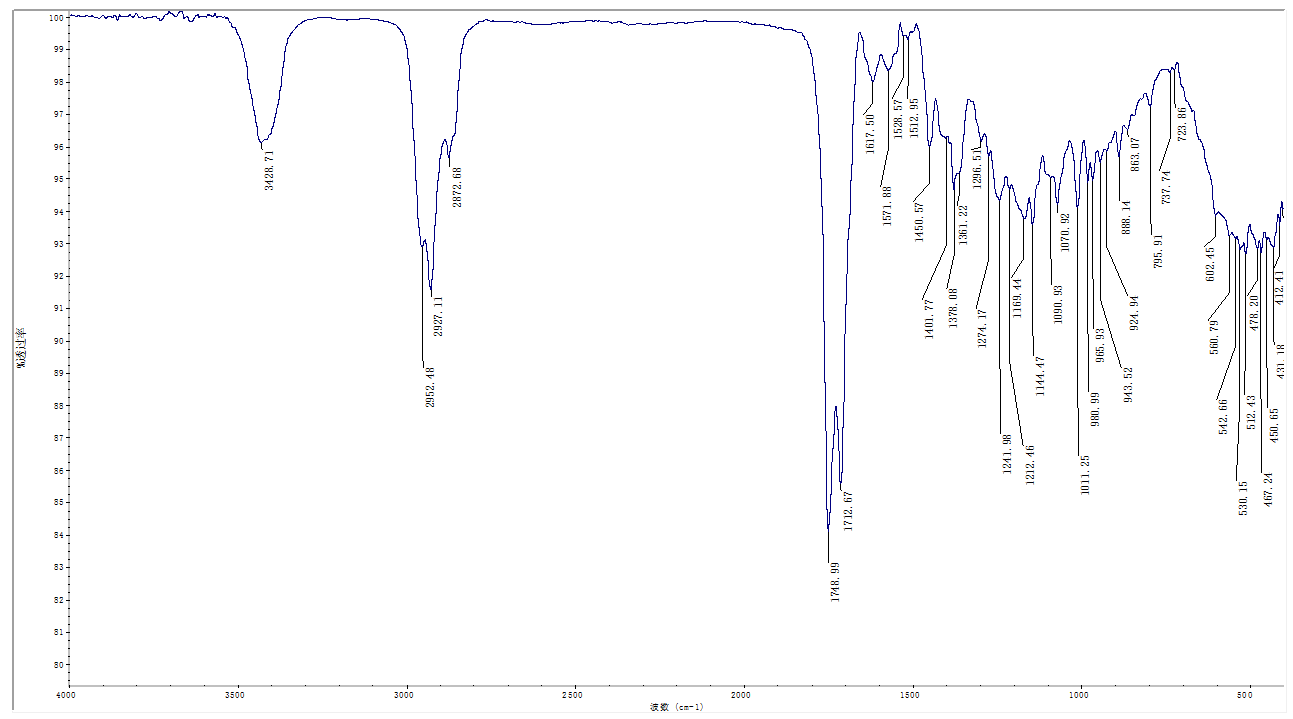


# S62. ECD of compound 7


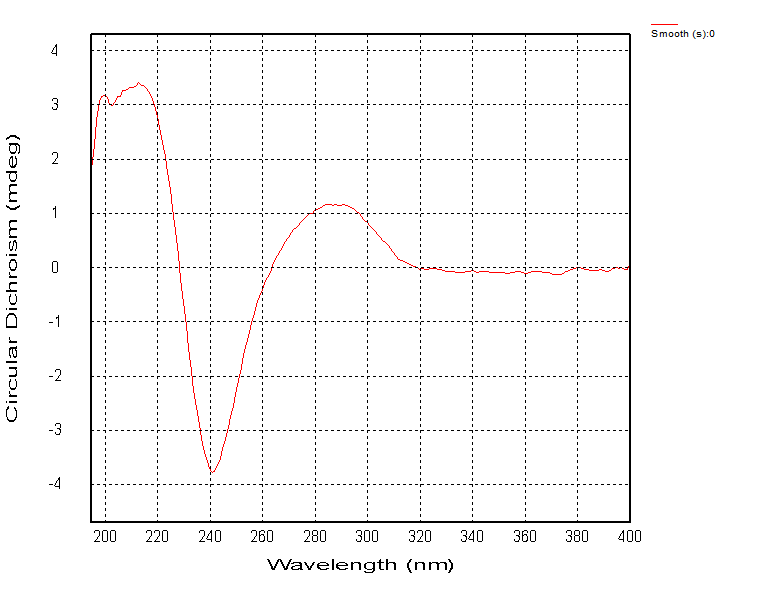


# S63. HRESIMS of compound 7


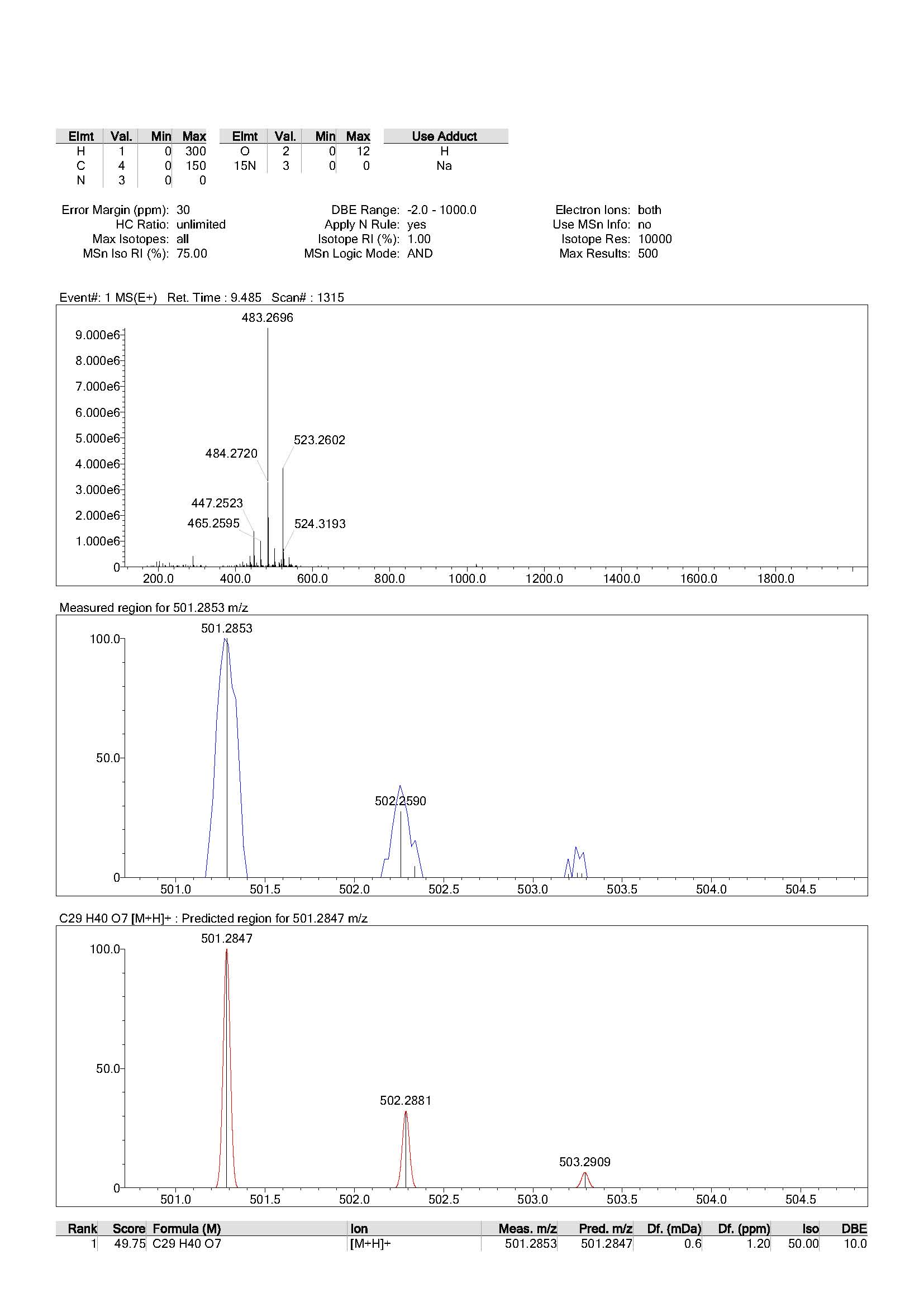


S64. ^1^H NMR (600 MHz, CDCl_3_) of compound **8**


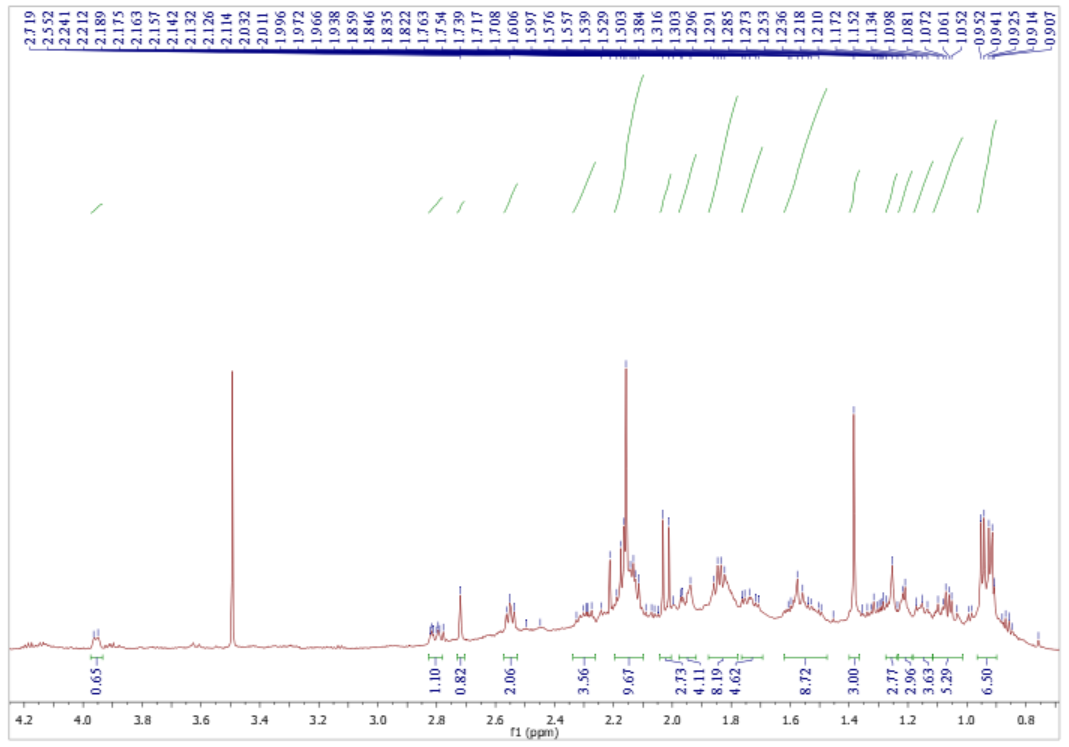


# S65. ^13^C NMR (DEPT) (150 MHz, CDCl_3_) of compound 8


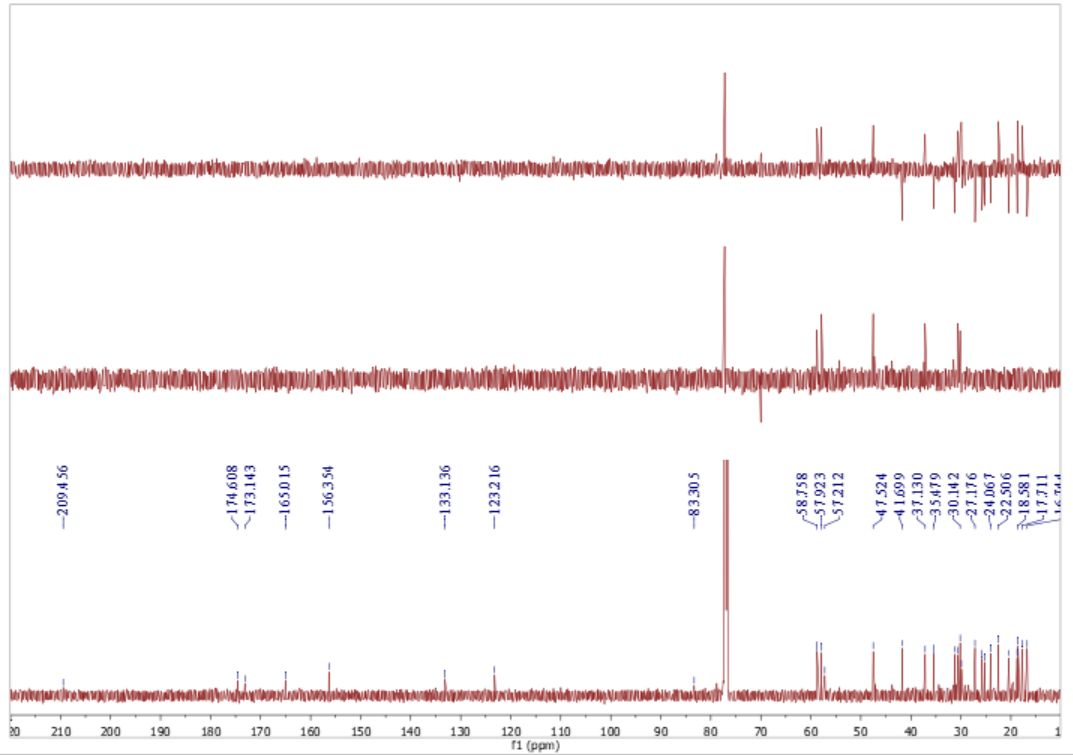


# S66. ^1^H-^1^H COSY (600 MHz, CDCl_3_) of compound 8


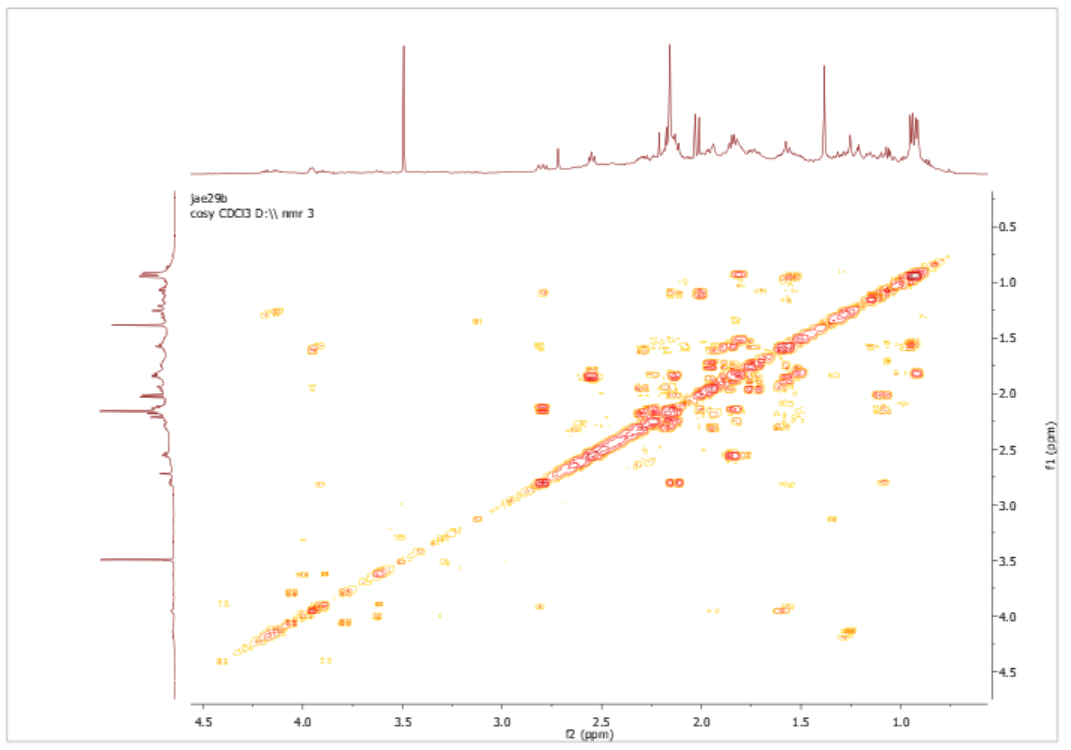


# S67. HSQC (600 MHz, CDCl_3_) of compound 8


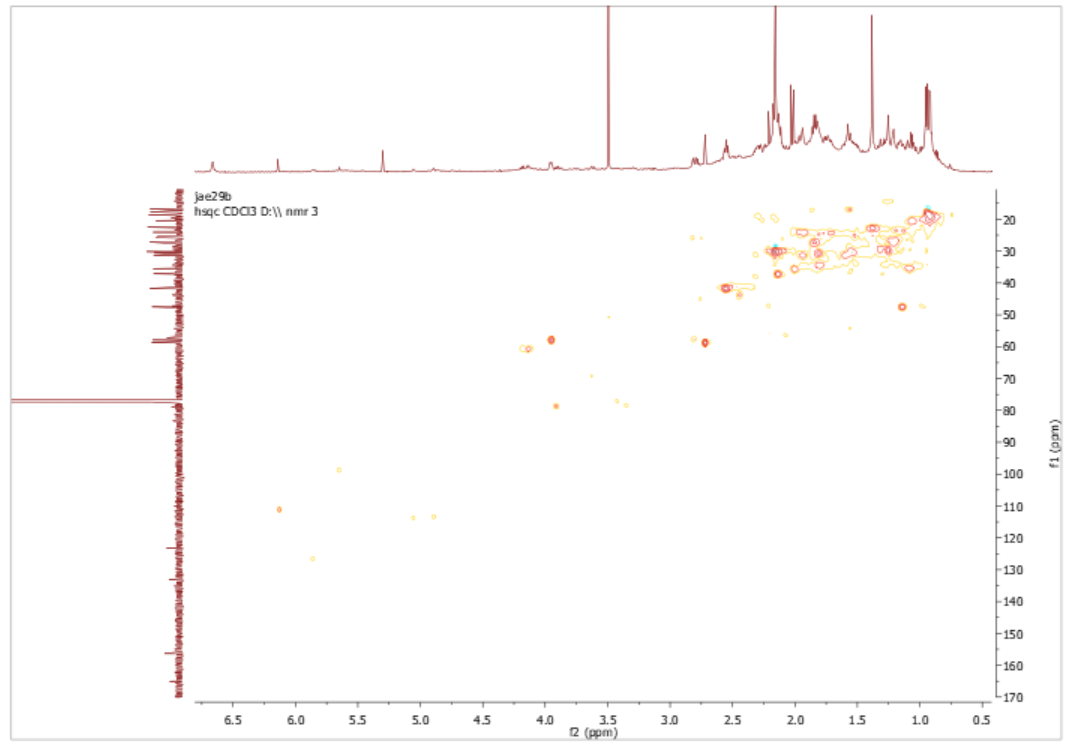


# S68. HMBC (600 MHz, CDCl_3_) of compound 8

# S69. ROESY (600 MHz, CDCl_3_) of compound 8

# S70. IR of compound 8

# S71. ECD of compound 8

# S72. HRESIMS of compound 8

# S73. ^1^H NMR (600 MHz, CDCl_3_) of compound 9

# S74. ^13^C NMR (DEPT) (150 MHz, CDCl_3_) of compound 9

# S75. ^1^H-^1^H COSY (600 MHz, CDCl_3_) of compound 9

# S76. HSQC (600 MHz, CDCl_3_) of compound 9

# S77. HMBC (600 MHz, CDCl_3_) of compound 9

# S78. ROESY (600 MHz, CDCl_3_) of compound 9

# S79. IR of compound 9

# S80. ECD of compound 9

# S81. HRESIMS of compound 9

# S82. ^1^H NMR (600 MHz, CDCl_3_) of compound 10

# S83. ^13^C NMR (DEPT) (150 MHz, CDCl_3_) of compound 10

# S84. ^1^H-^1^H COSY (600 MHz, CDCl_3_) of compound 10

# S85. HSQC (600 MHz, CDCl_3_) of compound 10

# S86. HMBC (600 MHz, CDCl_3_) of compound 10

# S87. ROESY (600 MHz, CDCl_3_) of compound 10

# S88. IR of compound 10

# S89. ECD of compound 10

# S90. HRESIMS of compound 10

# S91. ^1^H NMR (600 MHz, CDCl_3_) of compound 11

# S92. ^13^C NMR (DEPT) (150 MHz, CDCl_3_) of compound 11

# S93. ^1^H-^1^H COSY (600 MHz, CDCl_3_) of compound 11

# S94. HSQC (600 MHz, CDCl_3_) of compound 11

# S95. HMBC (600 MHz, CDCl_3_) of compound 11

# S96. ROESY (600 MHz, CDCl_3_) of compound 11

# S97. IR of compound 11

# S98. ECD of compound 11

# S99. HRESIMS of compound 11

# S100. ^1^H NMR (600 MHz, CDCl_3_) of compound 12

# S101. ^13^C NMR (DEPT) (150 MHz, CDCl_3_) of compound 12

# S102. ^1^H-^1^H COSY (600 MHz, CDCl_3_) of compound 12

# S103. HSQC (600 MHz, CDCl_3_) of compound 12

# S104. HMBC (600 MHz, CDCl_3_) of compound 12

# S105. ROESY (600 MHz, CDCl_3_) of compound 12

# S106. IR of compound 12

# S107. ECD of compound 12

# S108. HRESIMS of compound 12

# S109. ^1^H NMR (600 MHz, CDCl_3_) of compound 13

# S110. ^13^C NMR (DEPT) (150 MHz, CDCl_3_) of compound 13

# S111. ^1^H-^1^H COSY (600 MHz, CDCl_3_) of compound 13

# S112. HSQC (600 MHz, CDCl_3_) of compound 13

# S113. HMBC (600 MHz, CDCl_3_) of compound 13

# S114. ROESY (600 MHz, CDCl_3_) of compound 13

# S115. IR of compound 13

# S116. ECD of compound 13

# S117. HRESIMS of compound 13

# S118. ^1^H NMR (600 MHz, CDCl_3_) of compound 14

# S119. ^13^C NMR (DEPT) (150 MHz, CDCl_3_) of compound 14

# S120. ^1^H-^1^H COSY (600 MHz, CDCl_3_) of compound 14

# S121. HSQC (600 MHz, CDCl_3_) of compound 14

# S122. HMBC (600 MHz, CDCl_3_) of compound 14

# S123. ROESY (600 MHz, CDCl_3_) of compound 14

# S124. IR of compound 14

# S125. ECD of compound 14

# S126. HRESIMS of compound 14

# S127. ^1^H NMR (600 MHz, CD_3_OD) of compound 15

# S128. ^13^C NMR (DEPT) (150 MHz, CD_3_OD) of compound 15

# S129. ^1^H-^1^H COSY (600 MHz, CD_3_OD) of compound 15

# S130. HSQC (600 MHz, CD_3_OD) of compound 15

# S131. HMBC (600 MHz, CD_3_OD) of compound 15

# S132. ROESY (600 MHz, CD_3_OD) of compound 15

# S133. IR of compound 15

# S134. ECD of compound 15

# S135 HRESIMS of compound 15

# S136. ^1^H NMR (600 MHz, CDCl_3_) of compound 16

# S137. ^13^C NMR (DEPT) (150 MHz, CDCl_3_) of compound 16

# S138. ^1^H-^1^H COSY (600 MHz, CDCl_3_) of compound 16

# S139. HSQC (600 MHz, CDCl_3_) of compound 16

# S140. HMBC (600 MHz, CDCl_3_) of compound 16

# S141. ROESY (600 MHz, CDCl_3_) of compound 16

# S142. IR of compound 16

# S143. ECD of compound 16

# S144 HRESIMS of compound 16

# S145. ^1^H NMR (600 MHz, CDCl_3_) of compound 17

# S146. ^13^C NMR (DEPT) (150 MHz, CDCl_3_) of compound 17

# S147. ^1^H-^1^H COSY (600 MHz, CDCl_3_) of compound 17

# S148. HSQC (600 MHz, CDCl_3_) of compound 17

# S149. HMBC (600 MHz, CDCl_3_) of compound 17

# S150. ROESY (600 MHz, CDCl_3_) of compound 17

# S151. IR of compound 17

# S152. ECD of compound 17

# S153 HRESIMS of compound 17

# S154. ^1^H NMR (600 MHz, CDCl_3_) of compound 18

# S155. ^13^C NMR (DEPT) (150 MHz, CDCl_3_) of compound 18

# S156. ^1^H-^1^H COSY (600 MHz, CDCl_3_) of compound 18

# S157. HSQC (600 MHz, CDCl_3_) of compound 18

# S158. HMBC (600 MHz, CDCl_3_) of compound 18

# S159. ROESY (600 MHz, CDCl_3_) of compound 18

# S160. IR of compound 18

# S161. ECD of compound 18

# S162 HRESIMS of compound 18

# S163. ^1^H NMR (600 MHz, CD_3_OD) of compound 19

# S164. ^13^C NMR (DEPT) (150 MHz, CD_3_OD) of compound 19

# S165. ^1^H-^1^H COSY (600 MHz, CD_3_OD) of compound 19

# S166. HSQC (600 MHz, CD_3_OD) of compound 19

# S167. HMBC (600 MHz, CD_3_OD) of compound 19

# S168. ROESY (600 MHz, CD_3_OD) of compound 19

# S169. IR of compound 19

# S170. ECD of compound 19

# S171 HRESIMS of compound 19

# S172. ^1^H NMR (600 MHz, CDCl_3_) of compound 20

# S173. ^13^C NMR (DEPT) (150 MHz, CDCl_3_) of compound 20

# S174. ^1^H-^1^H COSY (600 MHz, CDCl_3_) of compound 20

# S175. HSQC (600 MHz, CDCl_3_) of compound 20

# S176. HMBC (600 MHz, CDCl_3_) of compound 20

# S177. ROESY (600 MHz, CDCl_3_) of compound 20

# S178. IR of compound 20

# S179. ECD of compound 20

# S180 HRESIMS of compound 20

# S181. ^1^H NMR (600 MHz, CD_3_OD) of compound 21

# S182. ^13^C NMR (DEPT) (150 MHz, CD_3_OD) of compound 21

# S183. ^1^H-^1^H COSY (600 MHz, CD_3_OD) of compound 21

# S184. HSQC (600 MHz, CD_3_OD) of compound 21

# S185. HMBC (600 MHz, CD_3_OD) of compound 21

# S186. ROESY (600 MHz, CD_3_OD) of compound 21

# S187. IR of compound 21

# S188. ECD of compound 21

# S189 HRESIMS of compound 21

# S190. ^1^H NMR (600 MHz, CDCl_3_) of compound 22

# S191. ^13^C NMR (DEPT) (150 MHz, CDCl_3_) of compound 22

# S192. ^1^H-^1^H COSY (600 MHz, CDCl_3_) of compound 22

# S193. HSQC (600 MHz, CDCl_3_) of compound 22

# S194. HMBC (600 MHz, CDCl_3_) of compound 22

# S195. ROESY (600 MHz, CDCl_3_) of compound 22

# S196. IR of compound 22

# S197. ECD of compound 22

# S198 HRESIMS of compound 22

# S199. ^1^H NMR (600 MHz, CD_3_OD) of compound 23

# S200. ^13^C NMR (DEPT) (150 MHz, CD_3_OD) of compound 23

# S201. ^1^H-^1^H COSY (600 MHz, CD_3_OD) of compound 23

# S202. HSQC (600 MHz, CD_3_OD) of compound 23

# S203. HMBC (600 MHz, CD_3_OD) of compound 23

# S204. ROESY (600 MHz, CD_3_OD) of compound 23

# S205. IR of compound 23

# S206. ECD of compound 23

# S207 HRESIMS of compound 23

# S208. ^1^H NMR (600 MHz, CD_3_OD) of compound 24

# S209. ^13^C NMR (DEPT) (150 MHz, CD_3_OD) of compound 24

# S210. ^1^H-^1^H COSY (600 MHz, CD_3_OD) of compound 24

# S211. HSQC (600 MHz, CD_3_OD) of compound 24

# S212. HMBC (600 MHz, CD_3_OD) of compound 24

# S213. ROESY (600 MHz, CD_3_OD) of compound 24

# S214. IR of compound 24

# S215. ECD of compound 24

# S216 HRESIMS of compound 24

# S217. ^1^H NMR (600 MHz, CD_3_OD) of compound 25

# S218. ^13^C NMR (DEPT) (150 MHz, CD_3_OD) of compound 25

# S219. ^1^H-^1^H COSY (600 MHz, CD_3_OD) of compound 25

# S220. HSQC (600 MHz, CD_3_OD) of compound 25

# S221. HMBC (600 MHz, CD_3_OD) of compound 25

# S222. ROESY (600 MHz, CD_3_OD) of compound 25

# S223. IR of compound 25

# S224. ECD of compound 25

# S225 HRESIMS of compound 25

# S226. ^1^H NMR (600 MHz, CDCl_3_) of compound 26

# S227. ^13^C NMR (DEPT) (150 MHz, CDCl_3_) of compound 26

# S228. ^1^H-^1^H COSY (600 MHz, CDCl_3_) of compound 26

# S229. HSQC (600 MHz, CDCl_3_) of compound 26

# S230. HMBC (600 MHz, CDCl_3_) of compound 26

# S231. ROESY (600 MHz, CDCl_3_) of compound 26

# S232. IR of compound 26

# S233. ECD of compound 26

# S234 HRESIMS of compound 26

# S235. ^1^H NMR (600 MHz, CD_3_OD) of compound 27

# S236. ^13^C NMR (DEPT) (150 MHz, CD_3_OD) of compound 27

# S237. ^1^H-^1^H COSY (600 MHz, CD_3_OD _3_) of compound 27

# S238. HSQC (600 MHz, CD_3_OD) of compound 27

# S239. HMBC (600 MHz, CD_3_OD) of compound 27

# S240. ROESY (600 MHz, CD_3_OD) of compound 27

# S241. IR of compound 27

# S242. ECD of compound 27

# S243 HRESIMS of compound 27

# S244. ^1^H NMR (600 MHz, CD_3_OD) of compound 28

# S245. ^13^C NMR (DEPT) (150 MHz, CD_3_OD) of compound 28

# S246. ^1^H-^1^H COSY (600 MHz, CD_3_OD) of compound 28

# S247. HSQC (600 MHz, CD_3_OD) of compound 28

# S248. HMBC (600 MHz, CD_3_OD) of compound 28

# S249. ROESY (600 MHz, CD_3_OD) of compound 28

# S250. IR of compound 28

# S251. ECD of compound 28

# S252 HRESIMS of compound 28

# S253. ^1^H NMR (600 MHz, CD_3_OD) of compound 29

# S254. ^13^C NMR (DEPT) (150 MHz, CD_3_OD) of compound 29

# S255. ^1^H-^1^H COSY (600 MHz, CD_3_OD) of compound 29

# S256. HSQC (600 MHz, CD_3_OD) of compound 29

# S257. HMBC (600 MHz, CD_3_OD) of compound 29

# S258. ROESY (600 MHz, CD_3_OD) of compound 29

# S259. IR of compound 29

# S260. ECD of compound 29

# S261 HRESIMS of compound 29

# S262. ^1^H NMR (600 MHz, CDCl_3_) of compound 30

# S263. ^13^C NMR (DEPT) (150 MHz, CDCl_3_) of compound 30

# S264. ^1^H-^1^H COSY (600 MHz, CDCl_3_) of compound 30

# S265. HSQC (600 MHz, CDCl_3_) of compound 30

# S266. HMBC (600 MHz, CDCl_3_) of compound 30

# S267. ROESY (600 MHz, CDCl_3_) of compound 30

# S268. IR of compound 30

# S269. ECD of compound 30

# S270 HRESIMS of compound 30

# S271. ^1^H NMR (600 MHz, CDCl_3_) of compound 31

# S272. ^13^C NMR (DEPT) (150 MHz, CDCl_3_) of compound 31

# S273. ^1^H-^1^H COSY (600 MHz, CDCl_3_) of compound 31

# S274. HSQC (600 MHz, CDCl_3_) of compound 31

# S275. HMBC (600 MHz, CDCl_3_) of compound 31

# S276. ROESY (600 MHz, CDCl_3_) of compound 31

# S277. IR of compound 31

# S278. ECD of compound 31

# S279 HRESIMS of compound 31

# S280. ^1^H NMR (600 MHz, CDCl_3_) of compound 32

# S281. ^13^C NMR (DEPT) (150 MHz, CDCl_3_) of compound 32

# S282. ^1^H-^1^H COSY (600 MHz, CDCl_3_) of compound 32

# S283. HSQC (600 MHz, CDCl_3_) of compound 32

# S284. HMBC (600 MHz, CDCl_3_) of compound 32

# S285. ROESY (600 MHz, CDCl_3_) of compound 32

# S286. IR of compound 32

# S287. ECD of compound 32

# S288 HRESIMS of compound 32

S289. ^1^H NMR (600 MHz, CD_3_OD) of compound **33**

# S290. ^13^C NMR (DEPT) (150 MHz, CD_3_OD) of compound 33

# S291. ^1^H-^1^H COSY (600 MHz, CD_3_OD) of compound 33

# S292. HSQC (600 MHz, CD_3_OD) of compound 33

# S293. HMBC (600 MHz, CD_3_OD) of compound 33

# S294. ROESY (600 MHz, CD_3_OD) of compound 33

# S295. IR of compound 33

# S296. ECD of compound 33

# S297 HRESIMS of compound 33

# S298. ^1^H NMR (600 MHz, CDCl_3_) of compound 34

# S299. ^13^C NMR (DEPT) (150 MHz, CDCl_3_) of compound 34

# S300. ^1^H-^1^H COSY (600 MHz, CDCl_3_) of compound 34

# S301. HSQC (600 MHz, CDCl_3_) of compound 34

# S302. HMBC (600 MHz, CDCl_3_) of compound 34

# S303. ROESY (600 MHz, CDCl_3_) of compound 34

# S304. IR of compound 34

# S305. ECD of compound 34

# S306 HRESIMS of compound 34

# S307 ^1^H NMR (600 MHz, CD_3_OD) of compound 35

# S308. ^13^C NMR (DEPT) (150 MHz, CD_3_OD) of compound 35

# S309. ^1^H-^1^H COSY (600 MHz, CD_3_OD) of compound 35

# S310. HSQC (600 MHz, CD_3_OD) of compound 35

# S311. HMBC (600 MHz, CD_3_OD) of compound 35

# S312. ROESY (600 MHz, CD_3_OD) of compound 35

# S313. IR of compound 35

# S314. ECD of compound 35

# S315 HRESIMS of compound 35

# S316 ^1^H NMR (600 MHz, CDCl_3_) of compound 36

# S317. ^13^C NMR (DEPT) (150 MHz, CDCl_3_) of compound 36

# S318. ^1^H-^1^H COSY (600 MHz, CDCl_3_) of compound 36

# S319. HSQC (600 MHz, CDCl_3_) of compound 36

# S320. HMBC (600 MHz, CDCl_3_) of compound 36

# S321. ROESY (600 MHz, CDCl_3_) of compound 36

# S322. IR of compound 36

# S323. ECD of compound 36

# S324 HRESIMS of compound 36
